# Silicon-oriented regio- and enantioselective rhodium-catalyzed hydroformylation

**DOI:** 10.1038/s41467-018-04277-7

**Published:** 2018-05-23

**Authors:** Cai You, Xiuxiu Li, Yuhong Yang, Yu-Sheng Yang, Xuefeng Tan, Shuailong Li, Biao Wei, Hui Lv, Lung-Wa Chung, Xumu Zhang

**Affiliations:** 1 Key Laboratory of Biomedical Polymers of Ministry of Education & College of Chemistry and Molecular Sciences, Wuhan University, Wuhan, 430072 Hubei People’s Republic of China; 2 Department of Chemistry, Southern University of Science and Technology, Shenzhen, 518055 Guangdong, People’s Republic of China; 3 Engineering Research Center of Organosilicon Compounds & Materials, Ministry of Education, College of Chemistry and Molecular Sciences, Wuhan University, Wuhan, 430072 Hubei People’s Republic of China

## Abstract

Hydroformylation of 1,2-disubstituted alkenes usually occurs at the α position of the directing heteroatom such as oxygen atom and nitrogen atom. By contrast, to achieve hydroformylation on the β position of the heteroatom is a tough task. Herein, we report the asymmetric rhodium-catalyzed hydroformylation of 1,2-disubstituted alkenylsilanes with excellent regioselectivity at the β position (relative to the silicon heteroatom) and enantioselectivity. In a synthetic sense, we achieve the asymmetric hydroformylation on the β position of the oxygen atom indirectly by using the silicon group as a surrogate for the hydroxyl. Density functional theory (DFT) calculations are carried out to examine energetics of the whole reaction path for Rh/YanPhos-catalyzed asymmetric hydroformylation and understand its regioselectivity and enantioselectivity. Our computational study suggests that the silicon group can activate the substrate and is critical for the regioselectivity.

## Abstract (web summary)

Hydroformylation of 1,2-disubstituted alkenes usually occurs at the α position of the directing heteroatom. Here, the authors report the asymmetric rhodium-catalyzed hydroformylation of 1,2-disubstituted alkenylsilanes with excellent regioselectivity at the β position (relative to the silicon heteroatom) and enantioselectivity.

## Introduction

**Fig. 1:**
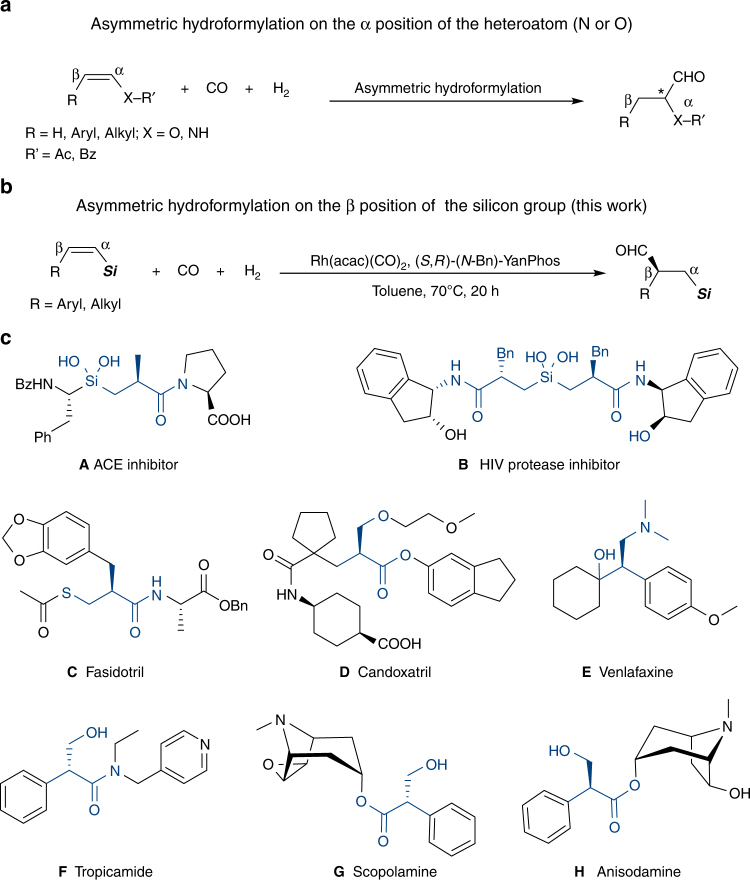
Design of a asymmetric hydroformylation on the β position of the heteroatom. **a** The α regioselectivity is controlled by electron-withdrawing inductive effects. **b** To achieve β regioselectivity by a hindered silicon group. **c** Structures of biologically active compounds and natural products containing derivatives of β-aldehydesilanes

Owing to the high atom economy, asymmetric hydroformylation (AHF) of alkenes provides an efficient way for the synthesis of enantiomerically pure aldehydes, which are versatile chiral intermediates for pharmaceuticals, agrochemicals, and other fine chemicals^1–5^. In the past decades, intensive research efforts have been made in this area, and a range of chiral phosphorus ligand systems have been developed for AHF reactions, including BINAPHOS^6–8^, bis(diazaphospholane) (BDP)^9–12^, Chiraphite^13^, Ph-BPE^14^, YanPhos^15–19^, and other phosphorus ligands^20–30^. Many simple terminal alkenes have been converted into chiral aldehydes with practical levels of regio- and enantioselectivity by AHF. However, for 1,2-disubstituted alkenes, the control of regioselectivity is a preeminent challenge because of the less steric difference between two substituent groups than in monosubstituted or 1,1-disubstituted alkenes. To date, only very limited examples have been reported in AHF of 1,2-disubstituted alkenes. To address this issue, Tan et al. designed the scaffolding catalysis for allyl amines and alcohols^20^, Reek et al. used the supramolecular catalysis in AHF of unactivated disubstituted olefins^27^, and in terms of the substrate design, Landis et al. successfully achieved AHF of 1,2-disubstituted alkenes comprising (*Z*)-enol esters and enamides with Rh-BDP catalysts^11^. Due to electron-withdrawing groups, high regioselectivities were given (up to >99:1) for AHF of (*Z*)-enol esters and enamides. The results suggest that the electronic and steric differentiation of the substituent groups is a key factor for the regioselectivity of this transformation. In these examples, CO was mainly incorporated at the α position of the oxygen atom or nitrogen atom (Fig. 1a)^9–11,31–34^. By contrast, to achieve the AHF on the β position of the heteroatom (O or N) is a tough task. Herein, we attempted to use the silicon group as a surrogate for the hydroxyl (via Fleming-Tamao oxidation^35^) in a synthetic sense, and the β aldehyde product is more favorable due to the steric hindrance of the silicon group (Fig. 1b)^36,37^, which achieves the AHF on the β position of the oxygen atom indirectly. Based on these ideas, a series of 1,2-disubstituted alkenylsilanes were designed and *Z*-alkenes were chosen instead of *E*-alkenes because of higher regio- and enantioselectivities and faster rates^7,11^.

Organosilicon compounds are essential synthetic reagents and intermediates in modern organic synthesis, and the Si atom itself is a key element in many functional materials and bioactive molecules. Particularly, chiral β-aldehydesilanes are valuable intermediates, which can be readily converted to other versatile building blocks occurring in a variety of small molecule pharmaceuticals and natural products, such as drugs A (angiotensin-converting enzyme inhibitor), B (HIV protease inhibitor)^38^, fasidotril^39^, candoxatril^40^, venlafaxine^41^, tropicamide^42^, and natural products scopolamine^43,44^ and anisodamine^45^ (Fig. 1c).

Herein, we report a rhodium-catalyzed regioselective and stereospecific hydroformylation of 1,2-disubstituted alkenylsilanes: CO can be mainly incorporated at the β position (up to >99/1 regioselectivity), respectively, and the corresponding β-aldehydesilanes are obtained with excellent enantioselectivity (up to 97% enantiomeric excesses (ee)). The products can be useful synthetic platforms based on the various transformations of the aldehyde group and silicon group (transformations of the silicon include Fleming-Tamao oxidation^35^, Hiyama coupling^46^, Brook and *retro*-Brook rearrangements^47,48^).

## Results

### Investigation of chiral ligands

Our initial studies focused on the AHF of (*Z*)-trimethyl(styryl)silane (**1a**) to give the desired chiral aldehyde product **2a**, with the expectations of achieving a highly regioselective and enantioselective transformation. To our delight, when (*S*,*R*)-(*N*-Bn)-YanPhos was employed, the reaction almost exclusively took place in the β position (the ratio of **2a**/**3a** is >99) to produce **2a** with 92% ee and 96% yield (Table 1, entry 1). As summarized in Table 1, an evaluation of ligands revealed (*S*,*R*)-(*N*-Bn)-YanPhos to be superior to all others tested (Table 1, entry 1 vs. entries 2–6). Rh catalysts based on other ligands, including XuPhos, (*S*,*S*)-Ph-BPE, (*R*c,*S*p)-DuanPhos, and (*R*)-QuinoxP*, exhibited high activity and excellent regioselectivity with low to good ee values, and almost no isomerization product was detected (Table 1, entries 2–5). (*S*)-BINAP, having axial chirality, was an unsatisfactory ligand for this reaction (Table 1, entry 6).

**Table 1:** Evaluation of chiral ligands


Entry	Ligand	Conv. (%)	Yield (%)		2a/3a	ee (%)
			**2a**+**3a**	**4a**		
1	**L1**	98	96	2	>99	92
2	**L2**	>99	>99	0	>99	42
3	**L3**	99	99	0	>99	48
4	**L4**	>99	>99	0	>99	80
5	**L5**	94	94	0	>99	60
6	**L6**	44	29	15	>99	−9

Reaction conditions: **1a** (0.5 mmol), Rh(acac)(CO)_2_ (0.5 mol%), ligand (1.0 mol%), CO (10 bar), H_2_ (10 bar), toluene (2 ml), 70 °C, 20 h. Conversions and yields were determined by ^1^H NMR analysis. Enantiomeric excesses (ee) were determined by HPLC analysis using a chiral stationary phase after NaBH_4_ reduction

### Investigation of reaction conditions

In order to achieve good regioselectivity and enantioselectivity and minimize the isomerization product **4a**, which was produced via the olefin insertion to the Rh-H bond followed by β-hydride elimination, we sought to obtain optimal reaction conditions, as summarized in Table 2. We found that different solvents have an influence on the chemoselectivity, but a very small effect on the regioselectivity and enantioselectivity (Table 2, entries 1 and 3–5). Interestingly, when tetrahydrofuran was used, the olefin isomerization product **4a** was obtained as the main product, which may be due to the solvent coordination effect (Table 2, entry 2). Next, the influence of the **L1**/Rh ratio was investigated. Lowering of the **L1**/Rh ratio gave higher conversion, but the yield of the desired product **2a** dropped significantly (Table 2, entry 6). When the **L1**/Rh ratio rose to 3, higher enantioselectivity (94% ee) with slightly lower yield (90% yield) was achieved (Table 2, entry 7). Under this ratio (**L1**/Rh = 3), the syngas pressure and reaction temperature were screened. The results indicated that lower syngas pressure and high temperature is beneficial to the conversion, which is consistent with the conclusion we have obtained for the YanPhos/Rh system (Table 2, entries 7–12)^15–19^. The complete conversion was achieved in 20 h under 10 bar of CO/H_2_ at 70 °C, affording near quantitative yield of the desired product 2a with 94% ee (Table 2, entry 8). Lower pressure (CO/H_2_ = 2/2 bar) was also tested, but no significant improvement was observed (Table 2, entry 9 vs. entry 8).

**Table 2:** Optimization of the asymmetric hydroformylation of 1a


Entry	Solvent	*x*	*T* (°C)	CO/H_2_ (bar)	Conv. (%)	Yield (%)		ee (%)
						**2a**+**3a**	**4a**	
1	Toluene	1.0	70	10/10	98	96	2	92
2	THF	1.0	70	10/10	>99	35	65	–
3	CH_2_Cl_2_	1.0	70	10/10	89	80	9	93
4	EtOAc	1.0	70	10/10	>99	95	5	91
5	DCE	1.0	70	10/10	>99	96	4	90
6^a^	Toluene	0.6	70	10/10	>99	25	75	–
7	Toluene	1.5	70	10/10	98	90	8	94
8	Toluene	1.5	70	5/5	>99	99	1	94
9	Toluene	1.5	70	2/2	>99	99	1	94
10	Toluene	1.5	70	20/20	99	91	8	94
11	Toluene	1.5	60	5/5	93	82	11	96
12	Toluene	1.5	80	5/5	>99	85	15	91

Reaction conditions: **1a** (0.5 mmol), Rh(acac)(CO)_2_ (0.5 mol%), toluene (2 ml), 20 h. Conversions and yields were determined by ^1^H NMR analysis. **2a**/**3a** = >99:1. Enantiomeric excesses (ee) were determined by HPLC analysis using a chiral stationary phase after NaBH_4_ reduction

^a^**2a**/**3a** = 18:1

### The study of isomerization reaction

**Fig. 2:**
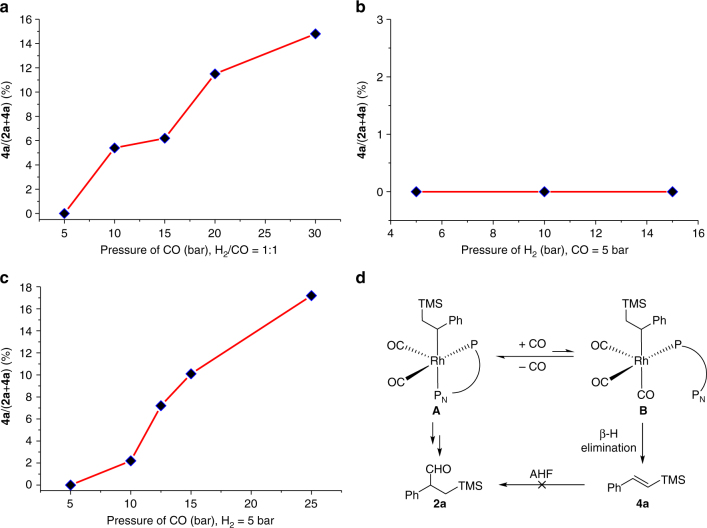
Exploration of the isomerization reaction. **a** The relation between the isomerization and total pressure. **b** The effect of H_2_ partial pressure on the isomerization. **c** The effect of CO partial pressure on the isomerization. **d** A proposal for the isomerization reaction

To gain a deeper understanding of the relationship between syngas pressure and isomerization, the effects of syngas (CO/H_2_ = 1:1), H_2_, and CO partial pressure on the isomerization were investigated systematically (Supplementary Table 1). Firstly, **4a** was used as the substrate directly and very low conversion (only 1%) was detected when the reactions were carried out in 20 h under 10 bar of syngas (CO/H_2_ = 1:1) at 70 °C (Supplementary Table 1, entry 1), which indicates that it is almost impossible to transform **4a** under current catalytic system. We also stopped the reaction at about 50% conversion (**2a** was used as the substrate, CO/H_2_ = 5:5 bar, 70 °C, 3.5 h), but only trace (<1%) **4a** was detected (Supplementary Table 1, entry 2), which means that the isomerization is very slow at low pressure. As shown in Fig. 2a, the ratio of **4a** shows a strong dependence on the total syngas pressure (CO/H_2_ = 1:1). To independently measure the effect of H_2_ partial pressure on the isomerization, one set of experiments used 5 bar CO pressure while varying H_2_ pressure from 5 to 15 bar. As shown in Fig. 2b, the ratio of **4a** was almost unchanged as the H_2_ pressure varied. The independent measure of the effect of CO partial pressure was also carried out, the H_2_ pressure was held at constant 5 bar, and the CO partial pressure was varied from 5 to 25 bar. As shown in Fig. 2c, raising the CO partial pressure could promote the isomerization, which is opposite to the results in previous papers^49^. We attempt to explain the problem from the mechanism, and proposed a proper path of the isomerization (Fig. 2d). Because of the competition between CO and the P_N_ part of YanPhos for the rhodium center, **A** could convert to **B**, which is critical for the isomerization. The coordination of CO to the rhodium species makes the Rh center of **B** electron deficient, which leads the CO on the equatorial position more weakly coordinated, while the CO on the axial position could not achieve the CO insertion. These properties of **B** may promote the β-hydride elimination to form **4a**^50^. With the CO partial pressure increasing, **B** could be formed more easily, which leads to more isomerization.

### Substrate scope

**Fig. 3:**
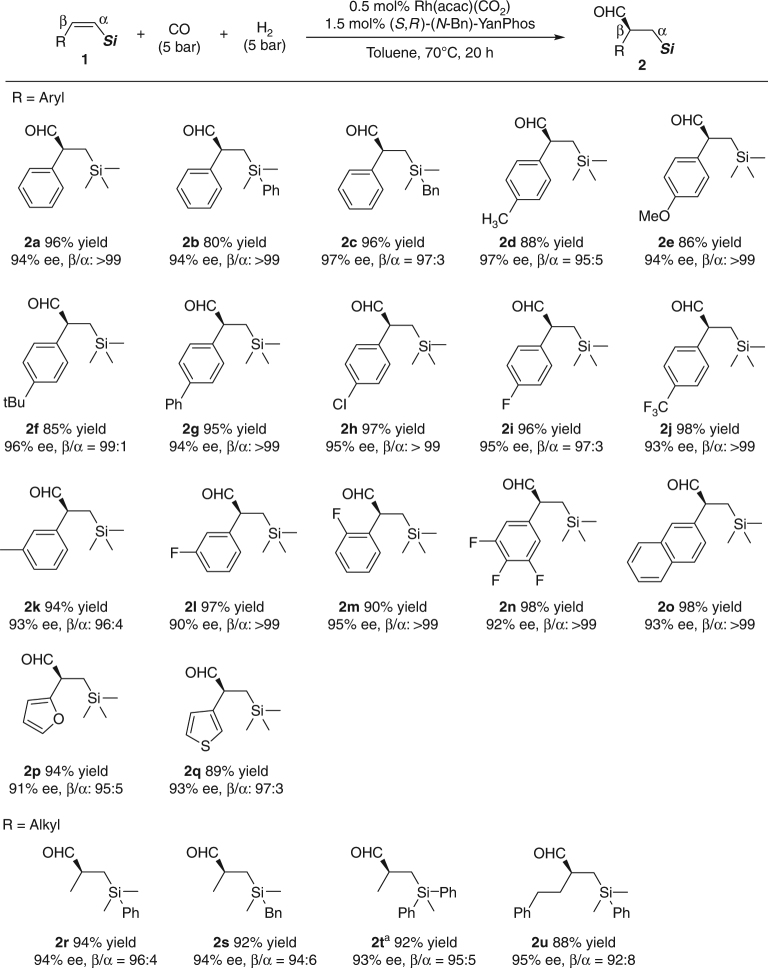
Asymmetric hydroformylation of *Z*-alkenylsilanes. Reaction conditions: **1** (0.5 mmol), Rh(acac)(CO)_2_ (0.5 mol%), (*S*,*R*)-(*N*-Bn)-YanPhos (1.5 mol%), CO (5 bar), H_2_ (5 bar), toluene (2 ml), 70 °C, 20 h. Isolated yields. Product ratios were determined by ^1^H NMR analysis of the unpurified reaction mixture. Enantiomeric excesses (ee) were determined by HPLC analysis using a chiral stationary phase after NaBH_4_ reduction. ^a^1.0 mol% Rh(acac)(CO)_2_ and 3.0 mol% (*S*,*R*)-(*N*-Bn)-YanPhos were used

With the optimized conditions in hand, we explored the substrate scope and generality of this asymmetric transformation (Fig. 3). First, in order to investigate the influence of substituents on the silicon atom, we changed trimethylsilyl (**1a**) to dimethyl(phenyl)silyl (**1b**) and benzyldimethylsilyl (**1c**). We found that when **1b** was employed as the substrate, the desired product **2b** was obtained with excellent regioselectivity and enantioselectivity albeit with 80% yield. While, **2c** was achieved in 96% yield with 97% ee and slightly lower regioselectivity (β/α = 97:3). Because of the simple preparation, trimethylsilyl was chosen as one substituent of the alkene, and a series of arylolefins were synthesized and investigated. Many functional groups, such as methyl (**2d**), methoxyl (**2e**), tertiary butyl (**2f**), phenyl (**2g**), trifluoromethyl (**2j**), and halides (**2h** and **2i**), are compatible with this transformation. It was found that, with electron-rich aryl alkenes, aldehyde products are formed in lower yields than that with electron-neutral and electron-deficient aryl alkenes. On the other hand, the regioselectivity and enantioselectivity are not obviously affected by the electrical properties of the benzene ring. Moreover, substrates with *meta*- or *ortho*- substitution on the phenyl group were readily accommodated (**2k**–**2m**). The highly electron-deficient substrate **1n** was also well tolerated. Furthermore, good yields, regioselectivities, and enantioselectivities were obtained with a range of substrates containing other aromatic fragments, including napthalenes, furans, and thiophenes (**2o**–**2q**). Then, more challenging substrates containing alkyl substituents were tested; to our delight, **2r** and **2s** were obtained with commendable results. And the compound **1t** with larger steric hindrance could also be readily used albeit with 1 mol% catalyst loading. By prolonging the chain length of the substituent, almost no effect on the reactivity was observed and excellent enantioselectivity (95% ee) and good regioselectivity (β/α = 92:8) remained (**2u**). It should be pointed out that silyl enol ethers were not detected in the present system^36,37^.

**Fig. 4:**
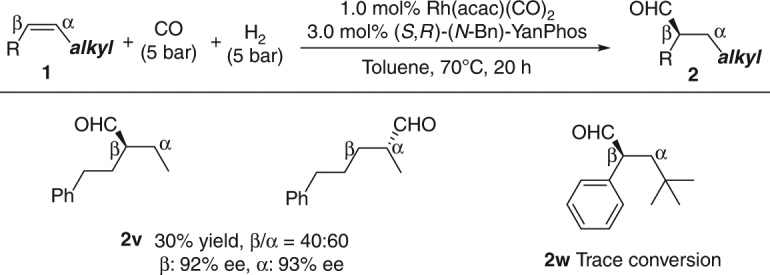
Substrate effect. Reaction conditions: **1** (0.5 mmol), Rh(acac)(CO)_2_ (1.0 mol%), (*S*,*R*)-(*N*-Bn)-YanPhos (3.0 mol%), CO (5 bar), H_2_ (5 bar), toluene (2 ml), 70 °C, 20 h. Yields and product ratios were determined by ^1^H NMR analysis of the unpurified reaction mixture. Enantiomeric excesses (ee) were determined by HPLC analysis using a chiral stationary phase after NaBH_4_ reduction

To illustrate the critical role of the silane group, *Z*-alkenes bearing an alkyl group were tested (Fig. 4). The poor regioselectivity of **2v** demonstrated that the hindered silicon groups, which facilitate the rhodium addition to the β position in the olefin insertion step, are very critical for the regiocontrol. Moreover, when **1w** was employed, almost no conversion was given. The low reaction activity of **1v** and **1w** implies an activating effect of the silicon groups in this transformation.

### Mechanistic studies

**Fig. 5:**
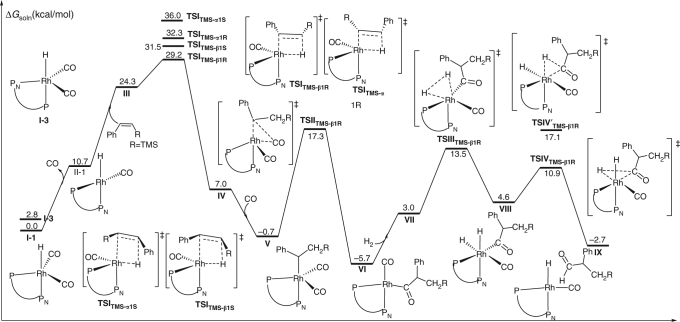
Computation studies on the mechanism. The migratory insertion of Rh-H to alkene was computed to be the rate-determining step and determines the regioselectivity and enantioselectivity. TS, transition state

**Fig. 6:**
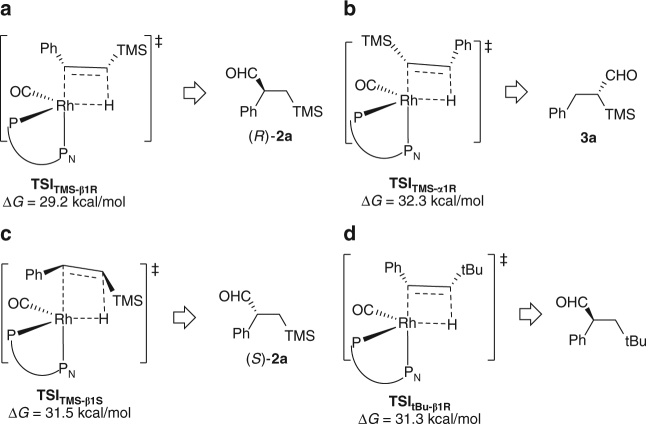
DFT calculations. Gibbs free energies of the Rh-H insertion step. **a** Gibbs free energies of **TSI**_**TMS-β1R**_. **b** Gibbs free energies of **TSI**_**TMS-α1R**_. **c** Gibbs free energies of **TSI**_**TMS-β1S**_. **d** Gibbs free energies of **TSI**_**tBu-β1R**_

**Fig. 7:**
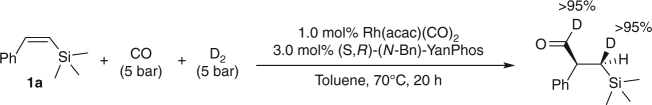
Deuteration experiments. The D incorporation in the products are >95% according to ^1^H NMR

On the basis of the previous computational and experimental results on other Rh-catalyzed hydroformylation^8,51–57^, we also carried out DFT calculations (using (*S*,*R*)-(*N*-Bn)-YanPhos and **1a**) to examine energetics of the whole reaction path for this Rh-catalyzed AHF and understand its regioselectivity and enantioselectivity to gain more insightful understanding (Figs. 5 and 6). As shown in Fig. 5, the alkene insertion step was computed to be the rate-determining step, which is in agreement with the previous computational studies^51–56^ and no observation of the H/D scrambling from the product (Fig. 7). In addition, as to the rate-determining step, **TSI**_**TMS-β1R**_ was computed to be lower in free energy than **TSI**_**TMS-α1R**_ by 3.1 kcal/mol in solution (i.e., calculated β/α > 99:1, Fig. 6a and b), which is consistent with the experimental result. On the other hand, **TSI**_**TMS-β1S**_ is computed to be higher in free energy than **TSI**_**TMS-β1R**_ by 2.3 kcal/mol in solution (i.e., calculated 98% ee, Fig. 6a and c), which is very close to the experimental result (94% ee). As shown in Fig. 6d, when **1w** was used as the substrate, this transformation suffers from a higher barrier in the alkene insertion step, which qualitatively explains poor reactivity. Both the experimental results and computational results reveal that the silane group plays a critical role in this reaction, especially in the control of the regioselectivity and the activation of the substrate.

### Applications of the transformation

**Fig. 8:**
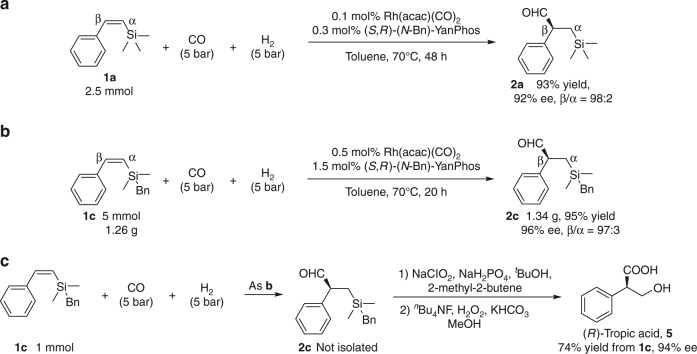
Synthetic applications of the asymmetric hydroformylation of *Z*-alkenylsilanes. **a** Asymmetric hydroformylation of **1a** with 0.1 mol% Rh(acac)(CO)_2_. **b** Gram scale synthesis of 2c. **c** Synthesis of (*R*)-tropic acid

To demonstrate the synthetic utility of the current methodology, the reaction was conducted with lower catalyst loading (S/C = 1000), affording the desired aldehyde **2a** in 93% yield with 92% ee and excellent regioselectivity (Fig. 8a). Gram scale reaction of **1c** could also proceed smoothly, and high yield, excellent regio- and enantioselectivity were remained (Fig. 8b). Furthermore, a creative synthetic route for chiral tropic acid which can be readily converted to tropicamide, hyoscyamine, scopolamine and anisodamine was developed^42,44^. As shown in Fig. 8c, the AHF product **2c** was subjected to sequential oxidation of the aldehyde group and Fleming-Tamao oxidation to afford the desired enantiomerically enriched tropic acid **5** (94% ee).

## Discussion

In conclusion, we develop an efficient approach for synthesizing valuable chiral β-aldehydesilanes by catalytic AHF of *Z*-alkenylsilanes. Because of the hindered silicon groups, this transformation exhibits excellent regioselectivities (β/α up to >99), and respectively, the corresponding β-aldehydesilanes are obtained with excellent enatioselectivities (up to 97% ee) under mild reaction conditions with low catalyst loading. By using the transformations of aldehyde groups and silicon groups, the products can be useful synthetic intermediates for bioactive molecules and natural products. Moreover, experiment results and DFT calculations indicate that the silicon group are primary factors of the regiocontrol and substrates’ reactivity in this asymmetric transformation.

## Methods

### General procedure for the AHF of alkenylsilanes

In a glovebox filled with nitrogen, to a 5 ml vial equipped with a magnetic bar was added ligand **L1** (0.0075 mmol) and Rh(acac)(CO)_2_ (0.0025 mmol in 0.5 mL solvent). After stirring for 10 min, substrate (0.5 mmol) and additional solvent were charged to bring the total volume of the reaction mixture to 2.0 mL. The vial was transferred into an autoclave and taken out of the glovebox. Carbon monoxide (5 bar) and hydrogen (5 bar) were charged in sequence. The reaction mixture was stirred at 70 °C (oil bath) for 20 h. The reaction was cooled and the pressure was carefully released in a well-ventilated hood. The conversion and β/α ratio were determined by ^1^H NMR spectroscopy from the crude reaction mixture. Enantiomeric excesses (ee) were determined by HPLC analysis using a chiral stationary phase after NaBH_4_ reduction.

### Data availability

The authors declare that the data supporting the conclusions of this study are available within the article and its Supplementary Information file or from the corresponding author upon reasonable request.

## Electronic supplementary material

Supplementary Information

Description of Additional Supplementary Files

Supplementary Data 1
