# Supplementary material for: Silicon-oriented regio- and enantioselective rhodium-catalyzed hydroformylation
Source: Nat Commun. 2018 May 23;9:2045. doi: 10.1038/s41467-018-04277-7 (PMC5966446; doi:10.1038/s41467-018-04277-7)
Supplement: Supplementary file 1 — Supplementary Information [file 41467_2018_4277_MOESM1_ESM.pdf]

Silicon-Oriented Regio- and Enantioselective Rhodium-Catalyzed Hydroformylation

You et al.

Supplementary Methods

All reactions and manipulations that were sensitive to moisture or air were performed in a nitrogen-filled glovebox or using standard Schlenk techniques, unless otherwise noted. Solvents were dried with standard procedures, degassed with N_2 and transferred by syringe. NMR spectra were recorded on Bruker ADVANCE III (400 MHz) spectrometers for ^{1}H NMR and ^{13}C NMR. $CDCl_3$ was the solvent used for the NMR analysis, with tetramethylsilane as the internal standard. Chemical shifts were reported up field to TMS (0.00 ppm) for ^{1}H NMR and relative to $CDCl_3$ (77.3 ppm) for ^{13}C NMR. Optical rotation was determined using a Perkin Elmer 343 polarimeter. HPLC analysis was conducted on an Agilent 1260 Series instrument. Thin layer chromatography (TLC) was performed on EM reagents 0.25 mm silica 60-F plates. All new products were further characterized by HRMS. A positive ion mass spectrum of sample was acquired on a Thermo LTQ-FT mass spectrometer with an electrospray ionization source.

Procedures for the preparation of substrates

All the substrates were prepared according to the literature[1-4].

(*Z*)-trimethyl(styryl)silane (1a)

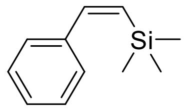

colorless oil; ^{1}H NMR (400 MHz, $CDCl_3$) δ: 7.44 (d, *J* = 15.1 Hz, 1H), 7.39-7.32 (m, 5H), 5.91 (d, *J* = 15.1 Hz, 1H), 0.15 (s, 9H). ^{13}C NMR (100 MHz, $CDCl_3$) δ: 146.6, 140.1, 132.8, 128.1, 127.9, 127.3, 0.2 ppm.

(*Z*)-dimethyl(phenyl)(styryl)silane (1b)

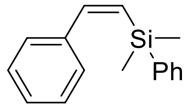

colorless oil; ^{1}H NMR (400 MHz, $CDCl_3$) δ: 7.57-7.52 (m, 2H), 7.49 (d, *J* = 15.1 Hz, 1H), 7.36-7.31 (m, 3H), 7.24-7.18 (m, 5H), 6.00 (d, *J* = 15.1 Hz, 1H), 0.26 (s, 6H). ^{13}C NMR (100 MHz, $CDCl_3$) δ: 148.3, 139.9, 139.8, 134.0, 130.4, 129.1, 128.5, 128.1, 127.8, -0.8 ppm.

(*Z*)-benzyldimethyl(styryl)silane (1c)

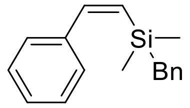

colorless oil; ^{1}H NMR (400 MHz, $CDCl_3$) δ: 7.42 (d, *J* = 15.1 Hz, 1H), 7.29-7.26 (m, 2H), 7.23-7.18 (m, 4H), 7.07 (t, *J* = 7.4 Hz, 1H), 7.02-6.94 (m, 2H), 5.80 (d, *J* = 15.1 Hz, 1H), 2.15 (s, 2H), 0.00 (s, 6H). ^{13}C NMR (100 MHz, $CDCl_3$) δ: 147.9, 140.3, 140.2, 130.9, 128.5, 128.4, 128.3, 128.2, 127.7, 124.3, 26.9, -1.5 ppm.

(*Z*)-trimethyl(4-methylstyryl)silane (1d)

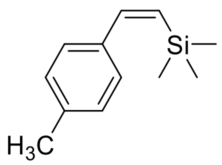

colorless oil; ^{1}H NMR (400 MHz, $CDCl_3$) δ: 7.32 (d, *J* = 15.0 Hz, 1H), 7.17 (d, *J* = 8.0 Hz, 2H), 7.11 (d, *J* = 8.0 Hz, 2H), 5.76 (d, *J* = 15.1 Hz, 1H), 2.33 (s, 3H), 0.06 (s, 9H). ^{13}C NMR (100 MHz, $CDCl_3$) δ: 146.8, 137.4, 137.4, 132.1, 128.9, 128.4, 21.6, 0.5 ppm.

(*Z*)-(4-methoxystyryl)trimethylsilane (1e)

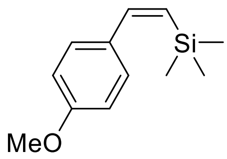

colorless oil; ^{1}H NMR (400 MHz, $CDCl_3$) δ: 7.21 (d, *J* = 15.1 Hz, 1H), 7.17-7.12 (m, 2H), 6.83-6.71 (m, 2H), 5.64 (d, *J* = 15.1 Hz, 1H), 3.73 (s, 3H), 0.00 (s, 9H). ^{13}C NMR (100 MHz, $CDCl_3$) δ: 159.3, 146.3, 132.9, 131.0, 129.7, 113.5, 55.5, 0.5 ppm.

(*Z*)-(4-(tert-butyl)styryl)trimethylsilane (1f)

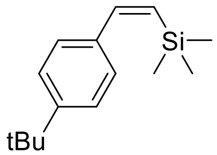

colorless oil; ^{1}H NMR (400 MHz, $CDCl_3$) δ: 7.27-7.22 (m, 3H), 7.16-7.14 (m, 2H), 5.70 (d, *J* = 15.2 Hz, 1H), 1.24 (s, 9H), 0.00 (s, 9H). ^{13}C NMR (100 MHz, $CDCl_3$) δ: 150.7, 146.7, 137.3, 132.0, 128.2, 125.1, 34.8, 31.6, 0.5 ppm. **HRMS** calculated $[M+H]^+$ for $C_{15}H_{25}Si$ = 233.1720, found: 233.1715.

(*Z*)-(2-([1,1'-biphenyl]-4-yl)vinyl)trimethylsilane (1g)

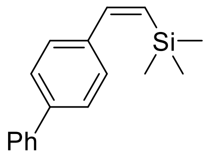

white solid; ^{1}H NMR (400 MHz, $CDCl_3$) δ: 7.53-7.45 (m, 4H), 7.43-7.29 (m, 3H), 7.27-7.23 (m, 3H), 5.76 (d, *J* = 15.2 Hz, 1H), 0.00 (s, 9H). ^{13}C NMR (100 MHz, $CDCl_3$) δ 146.3, 141.0, 140.3, 139.3, 133.2, 129.0, 128.9, 127.6, 127.2, 126.9, 0.5 ppm. **HRMS** calculated $[M+H]^+$ for $C_{17}H_{21}Si$ = 253.1407, found: 253.1400.

(*Z*)-(4-chlorostyryl)trimethylsilane (1h)

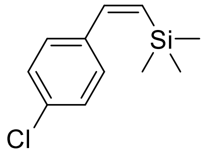

colorless oil; ^{1}H NMR (400 MHz, $CDCl_3$) δ: 7.26-7.20 (m, 3H), 7.18-7.11 (m, 2H), 5.81 (d, *J* = 15.2 Hz, 1H), 0.00 (s, 9H). ^{13}C NMR (100 MHz, $CDCl_3$) δ: 145.4, 138.8, 134.1, 133.4, 129.7, 128.4, 0.4

ppm.

(*Z*)-(4-fluorostyryl)trimethylsilane (1i)

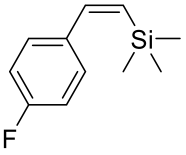

colorless oil; ^{1}H NMR (400 MHz, $CDCl_3$) δ: 7.26 (d, *J* = 15.1 Hz, 1H), 7.21-7.16 (m, 2H), 6.99-6.91 (m, 2H), 5.77 (d, *J* = 15.1 Hz, 1H), 0.00 (s, 9H). ^{13}C NMR (100 MHz, $CDCl_3$) δ 162.4 (d, *J* = 246.4 Hz), 145.6, 136.5 (d, *J* = 3.3 Hz), 133.2 (d, *J* = 1.0 Hz), 130.0 (d, *J* = 8.0 Hz), 115.1 (d, *J* = 21.4 Hz), 0.4 ppm. **HRMS** calculated $[M+H]^+$ for $C_{11}H_{16}FSi$ = 195.1000, found: 195.0995.

(*Z*)-(4-fluorostyryl)trimethylsilane (1i)

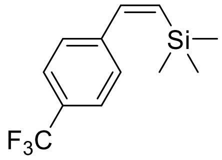

colorless oil; ^{1}H NMR (400 MHz, $CDCl_3$) δ: 7.52 (d, *J* = 8.1 Hz, 2H), 7.33-7.31 (m, 3H), 5.92 (d, *J* = 15.2 Hz, 1H), 0.00 (s, 9H). ^{13}C NMR (100 MHz, $CDCl_3$) δ: 145.2, 143.9 (d, *J* = 1.2 Hz), 135.8, 129.6 (q, *J* = 32.4 Hz), 128.6, 125.2 (q, *J* = 3.8 Hz), 124.5 (d, *J* = 271.9 Hz), 0.4 ppm.

(*Z*)-trimethyl(3-methylstyryl)silane (1k)

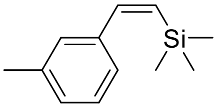

colorless oil; ^{1}H NMR (400 MHz, $CDCl_3$) δ: 7.28 (d, *J* = 15.1 Hz, 1H), 7.14 (t, *J* = 7.5 Hz, 1H), 7.06-6.98 (m, 3H), 5.74 (d, *J* = 15.1 Hz, 1H), 2.29 (s, 3H), 0.00 (s, 9H). ^{13}C NMR (100 MHz, $CDCl_3$) δ: 147.0, 140.2, 137.6, 132.8, 129.2, 128.3, 128.1, 125.4, 21.6, 0.5 ppm. **HRMS** calculated $[M+H]^+$ for $C_{12}H_{19}Si$ = 191.1251, found: 191.1245.

(*Z*)-(3-fluorostyryl)trimethylsilane (1l)

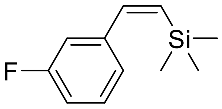

colorless oil; ^{1}H NMR (400 MHz, $CDCl_3$) δ: 7.26-7.18 (m, 2H), 6.98 (d, *J* = 7.7 Hz, 1H), 6.93-6.86 (m, 2H), 5.83 (d, *J* = 15.1 Hz, 1H), 0.00 (s, 9H). ^{13}C NMR (100 MHz, $CDCl_3$) δ: 162.8 (d, *J* = 245.5 Hz), 145.4 (d, *J* = 2.2 Hz), 142.7 (d, *J* = 7.4 Hz), 134.6, 129.7 (d, *J* = 8.3 Hz), 124.1 (d, *J* = 2.8 Hz), 115.1 (d, *J* = 21.3 Hz), 114.4 (d, *J* = 21.2 Hz), 0.4 ppm. **HRMS** calculated $[M+H]^+$ for $C_{11}H_{16}FSi$ = 195.1000, found: 195.0995.

(*Z*)-(2-fluorostyryl)trimethylsilane (1m)

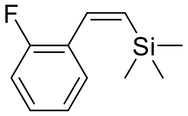

colorless oil; ^{1}H NMR (400 MHz, $CDCl_3$) δ: 7.32 (d, J = 15.2 Hz, 1H), 7.26-7.20 (m, 2H), 7.09-6.96 (m, 2H), 5.98 (d, J = 15.2 Hz, 1H), 0.00 (s, 9H). ^{13}C NMR (100 MHz, $CDCl_3$) δ: 160.3 (d, J = 246.6 Hz), 139.3 (d, J = 2.9 Hz), 135.9 (d, J = 0.9 Hz), 130.5 (d, J = 3.6 Hz), 129.4 (d, J = 8.1 Hz), 128.2 (d, J = 15.1 Hz), 123.7 (d, J = 3.6 Hz), 115.4 (d, J = 21.9 Hz), 0.13 ppm. **HRMS** calculated $[M+H]^+$ for $C_{11}H_{16}FSi$ = 195.1000, found: 195.0997.

(*Z*)-trimethyl(3,4,5-trifluorostyryl)silane (1n)

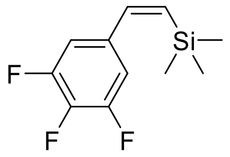

colorless oil; ^{1}H NMR (400 MHz, $CDCl_3$) δ :7.09 (d, J = 15.1 Hz, 1H), 6.85-6.74 (m, 2H), 5.85 (d, J = 15.1 Hz, 1H), 0.00 (s, 9H). ^{13}C NMR (100 MHz, $CDCl_3$) δ: 151.0 (ddd, J = 249.7, 10.1, 4.1 Hz), 143.5 (d, J = 1.8 Hz), 139.3 (dt, J = 251.7, 15.4 Hz), 136.4 (dd, J = 7.4, 2.7 Hz), 136.1 (d, J = 1.2 Hz), 112.3 (dd, J = 15.6, 5.7 Hz), 0.3 ppm. **HRMS** calculated $[M+H]^+$ for $C_{11}H_{12}F_3Si$ = 229.0666, found: 229.0659.

(*Z*)-trimethyl(2-(naphthalen-2-yl)vinyl)silane (1o)

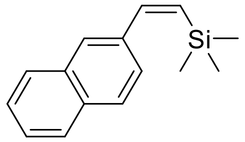

colorless oil; ^{1}H NMR (400 MHz, $CDCl_3$) δ: 7.73-7.64 (m, 4H), 7.41 (d, J = 15.2 Hz, 1H), 7.38-7.31 (m, 3H), 5.83 (d, J = 15.2 Hz, 1H), 0.00 (s, 9H). ^{13}C NMR (100 MHz, $CDCl_3$) δ: 146.8, 137.8, 133.6, 133.4, 133.0, 128.3, 127.9, 127.7, 127.4, 126.6, 126.4, 126.2, 0.6 ppm.

(*Z*)-(2-(furan-2-yl)vinyl)trimethylsilane (1p)

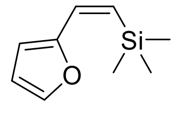

colorless oil; ^{1}H NMR (400 MHz, $CDCl_3$) δ: 7.26-7.18 (m, 1H), 6.75 (d, J = 15.5 Hz, 1H), 6.22 (dd, J = 3.3, 1.8 Hz, 1H), 6.08 (d, J = 3.3 Hz, 1H), 5.52 (d, J = 15.5 Hz, 1H), 0.00 (s, 9H). ^{13}C NMR (100 MHz, $CDCl_3$) δ: 154.8, 142.2, 132.2, 129.6, 111.8, 110.6, 0.4 ppm. **HRMS** calculated $[M+H]^+$ for $C_9H_{15}OSi$ = 167.0887, found: 167.0884.

(*Z*)-trimethyl(2-(thiophen-3-yl)vinyl)silane (1q)

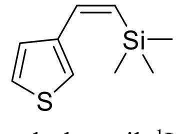

colorless oil; ^{1}H NMR (400 MHz, $CDCl_3$) δ: 7.16-7.12 (m, 2H), 7.04 (dd, J = 1.9, 1.0 Hz, 1H), 6.97 (dd, J = 4.9, 1.0 Hz, 1H), 5.68 (d, J = 15.2 Hz, 1H), 0.00 (s, 9H). ^{13}C NMR (100 MHz, $CDCl_3$) δ: 142.0, 140.5, 132.4, 128.2, 125.4, 123.6, 0.3 ppm. **HRMS** calculated $[M+H]^+$ for $C_9H_{15}SSi$ = 183.0658, found: 183.0656.

(*Z*)-dimethyl(phenyl)(prop-1-en-1-yl)silane (1r)

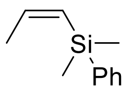

colorless oil; ^{1}H NMR (400 MHz, $CDCl_3$) δ: 7.58-7.53 (m, 2H), 7.36-7.33 (m, 3H), 6.53 (dq, *J* = 13.7, 6.8 Hz, 1H), 5.66 (dd, *J* = 14.0, 1.5 Hz, 1H), 1.72 (dd, *J* = 6.8, 1.5 Hz, 3H), 0.38 (s, 6H). ^{13}C NMR (100 MHz, $CDCl_3$) δ: 145.4, 139.9, 134.0, 129.0, 128.0, 127.9, 19.7, -0.6 ppm.

(*Z*)-benzyldimethyl(prop-1-en-1-yl)silane (1s)

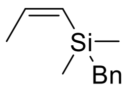

colorless oil; ^{1}H NMR (400 MHz, $CDCl_3$) δ: 7.12-7.05 (m, 2H), 6.99-6.89 (m, 3H), 6.32 (dq, *J* = 13.7, 6.8 Hz, 1H), 5.36 (dq, *J* = 14.0, 1.4 Hz, 1H), 2.07 (s, 2H), 1.60 (dd, *J* = 6.8, 1.5 Hz, 3H), 0.00 (s, 6H). ^{13}C NMR (100 MHz, $CDCl_3$) δ: 144.7, 140.4, 128.5, 128.4, 128.0, 124.2, 26.9, 19.5, -1.5 ppm. **HRMS** calculated $[M+H]^+$ for $C_{12}H_{19}Si$ = 191.1251, found: 191.1245.

(*Z*)-methyldiphenyl(prop-1-en-1-yl)silane (1t)

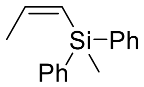

colorless oil; ^{1}H NMR (400 MHz, $CDCl_3$) δ: 7.58-7.53 (m, 4H), 7.36-7.32 (m, 6H), 6.68 (dq, *J* = 13.7, 6.9 Hz, 1H), 5.85 (ddd, *J* = 14.0, 2.9, 1.4 Hz, 1H), 1.65 (dd, *J* = 6.9, 1.5 Hz, 3H), 0.66 (s, 3H). ^{13}C NMR (100 MHz, $CDCl_3$) δ: 147.1, 137.8, 134.9, 129.4, 128.1, 126.0, 20.1, -1.7 ppm. **HRMS** calculated $[M+H]^+$ for $C_{16}H_{19}Si$ = 239.1251, found: 239.1253.

(*Z*)-dimethyl(phenyl)(4-phenylbut-1-en-1-yl)silane (1u)

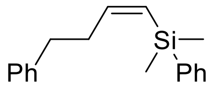

colorless oil; ^{1}H NMR (400 MHz, $CDCl_3$) δ: 7.55-7.50 (m, 2H), 7.35-7.33 (m, 3H), 7.27-7.11 (m, 4H), 7.01 (d, *J* = 7.1 Hz, 2H), 6.46 (dt, *J* = 14.4, 7.4 Hz, 1H), 5.68 (d, *J* = 14.0 Hz, 1H), 2.57 (dd, *J* = 9.0, 6.7 Hz, 2H), 2.34 (dd, *J* = 15.5, 7.5 Hz, 2H), 0.34 (s, 6H). ^{13}C NMR (100 MHz, $CDCl_3$) δ: 149.9, 141.9, 139.9, 134.0, 129.1, 128.7, 128.5, 128.1, 127.8, 126.1, 36.0, -0.6 ppm.

General procedure for asymmetric hydroformylation

In a glovebox filled with nitrogen, to a 5 ml vial equipped with a magnetic bar was added ligand **L1** (0.0075 mmol) and $Rh(acac)(CO)_2$ (0.0025 mmol in 0.5 mL solvent). After stirring for 10 min, substrate (0.5 mmol) and additional solvent was charged to bring the total volume of the reaction mixture to 2.0 mL. The vial was transferred into an autoclave and taken out of the glovebox. Carbon monoxide (5 atm) and hydrogen (5 atm) were charged in sequence. The reaction mixture was stirred at 70 ℃ (oil bath) for 20 h. The reaction was cooled and the pressure was carefully released in a well-ventilated hood. The conversion and β/α ratio were determined by ^{1}H NMR spectroscopy from

the crude reaction mixture. The enantiomeric excesses were determined by GC analysis or by HPLC analysis.

NMR, optical rotation and HRMS Data of 2

The enantiomeric excesses of **2** were determined HPLC after $NaBH_4$ reduction. Compounds **2** and the corresponding alcohols were isolated by column chromatography (**2**: AcOEt/hexane 1:30 to 1:20; the alcohol: AcOEt/hexane 1:20 to 1:10). Because of the racemization of the chiral aldehydes (**2a-2q**) after purified by column chromatography, at least two batches of aldehydes were obtained, one of them (the crude aldehydes) was reduced by $NaBH_4$ immediately for the e.e. determination, and the other one was purified for the data of **2**, including yields, NMR etc. The optical rotation is the data of the **corresponding alcohol**. The **absolute configuration** was assigned by comparing the sign of the optical rotation of the derivative, ***(R)*-tropic acid 5**, with that reported in the literature, see ref 3.

(*S*)-2-phenyl-3-(trimethylsilyl)propanal (2a)

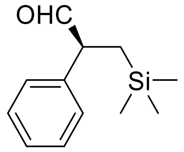

colorless oil; Isolated yield: 96%; 96% ee; $[\alpha]_D^{20}$ = 11.4 (c = 1.0, $CHCl_3$); The enantiomeric excess was determined by HPLC on Chiralpak OD-H column, hexane: isopropanol = 99:1; flow rate = 1.0 mL/min; UV detection at 220 nm; t_R = 15.2 min (major), 17.0 min (minor). ^{1}H NMR (400 MHz, $CDCl_3$) δ: 9.73 (d, *J* = 2.2 Hz, 1H), 7.50-7.46 (m, 2H), 7.43-7.37 (m, 1H), 7.35-7.32 (m, 2H), 3.68 (ddd, *J* = 9.8, 5.6, 2.2 Hz, 1H), 1.41 (dd, *J* = 14.8, 5.6 Hz, 1H), 1.16 (dd, *J* = 14.8, 9.8 Hz, 1H), 0.00 (s, 9H). ^{13}C NMR (100 MHz, $CDCl_3$) δ: 201.2, 138.0, 129.2, 129.0, 127.81, 55.4, 17.2, -0.1 ppm. **HRMS** calculated $[M+H]^+$ for $C_{12}H_{19}OSi$ = 207.1200, found: 207.1195.

(*S*)-3-(dimethyl(phenyl)silyl)-2-phenylpropanal (2b)

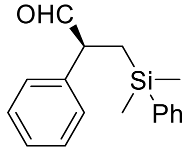

colorless oil; Isolated yield: 80%; 94% ee; $[\alpha]_D^{20}$ = 29.8 (c = 1.0, $CHCl_3$); The enantiomeric excess was determined by HPLC on Chiralpak OD-H column, hexane: isopropanol = 97:3; flow rate = 1.0 mL/min; UV detection at 220 nm; t_R = 20.0 min (minor), 24.5 min (major). ^{1}H NMR (400 MHz, $CDCl_3$) δ: 9.44 (d, *J* = 1.9 Hz, 1H), 7.34-7.28 (m, 2H), 7.24-7.10 (m, 6H), 7.01-6.99 (m, 2H), 3.38 (ddd, *J* = 9.6, 5.6, 1.9 Hz, 1H), 1.45 (dd, *J* = 14.9, 5.6 Hz, 1H), 1.12 (dd, *J* = 14.9, 9.6 Hz, 1H), 0.01 (s, 3H), -0.01 (s, 3H). ^{13}C NMR (100 MHz, $CDCl_3$) δ: 200.8, 138.4, 137.7, 133.8, 129.3, 129.2, 129.1, 128.0, 127.8, 55.2, 16.4, -2.1, -2.7 ppm. **HRMS** calculated $[M+H]^+$ for $C_{17}H_{21}OSi$ = 269.1356, found: 269.1352.

(*S*)-3-(benzyldimethylsilyl)-2-phenylpropanal (2c)

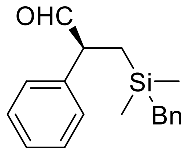

colorless oil; Isolated yield: 96%; 97% ee; $[\alpha]_D^{20}$ = 14.6 (c = 1.0, $CHCl_3$); The enantiomeric excess was determined by HPLC on Chiralpak OD-H column, hexane: isopropanol = 99:1; flow rate = 1.0 mL/min; UV detection at 210 nm; t_R = 33.5 min (major), 49.9 min (minor). ^{1}H NMR (400 MHz, $CDCl_3$) δ: 9.69 (d, J = 2.0 Hz, 1H), 7.48-7.44 (m, 2H), 7.42-7.36 (m, 1H), 7.34-7.25 (m, 4H), 7.22-7.16 (m, 1H), 7.06-7.04 (m, 2H), 3.62 (ddd, J = 9.6, 5.6, 2.0 Hz, 1H), 2.15-2.05 (m, 2H), 1.45 (dd, J = 14.9, 5.6 Hz, 1H), 1.16 (dd, J = 14.9, 9.6 Hz, 1H), 0.00 (s, 3H), -0.08 (s, 3H). ^{13}C NMR (100 MHz, $CDCl_3$) δ: 200.8, 139.9, 137.8, 129.2, 129.0, 128.5, 128.3, 127.9, 124.3, 55.1, 26.0, 15.5, -2.8, -3.0 ppm. **HRMS** calculated $[M+H]^+$ for $C_{18}H_{23}OSi$ = 283.1513, found: 283.1505.

(*S*)-2-(p-tolyl)-3-(trimethylsilyl)propanal (2d)

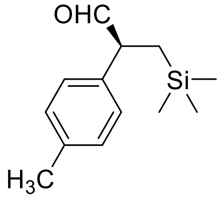

colorless oil; Isolated yield: 88%; 97% ee; $[\alpha]_D^{20}$ = 22.1 (c = 1.0, $CHCl_3$); The enantiomeric excess was determined by HPLC on Chiralpak OD-H column, hexane: isopropanol = 99:1; flow rate = 1.0 mL/min; UV detection at 220 nm; t_R = 10.5 min (minor), 11.2 min (major). ^{1}H NMR (400 MHz, $CDCl_3$) δ: 9.70 (d, J = 2.2 Hz, 1H), 7.28 (d, J = 8.0 Hz, 2H), 7.21 (d, J = 8.0 Hz, 2H), 3.63 (ddd, J = 9.8, 5.6, 2.2 Hz, 1H), 2.45 (s, 3H), 1.38 (dd, J = 14.8, 5.6 Hz, 1H), 1.14 (dd, J = 14.8, 9.8 Hz, 1H), 0.00 (s, 9H). ^{13}C NMR (100 MHz, $CDCl_3$) δ: 201.2, 137.5, 134.8, 129.9, 128.9, 54.9, 21.3, 17.1, -0.9 ppm. **HRMS** calculated $[M+H]^+$ for $C_{13}H_{21}OSi$ = 221.1356, found: 221.1352.

(*S*)-2-(4-methoxyphenyl)-3-(trimethylsilyl)propanal (2e)

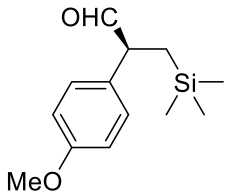

colorless oil; Isolated yield: 86%; 94% ee; $[\alpha]_D^{20}$ = 15.5 (c = 1.0, $CHCl_3$); The enantiomeric excess was determined by HPLC on Chiralpak AD-H column, hexane: isopropanol = 97:3; flow rate = 0.8 mL/min; UV detection at 210 nm; t_R = 15.0 min (minor), 17.7 min (major). ^{1}H NMR (400 MHz, $CDCl_3$) δ: 9.68 (d, J = 2.2 Hz, 1H), 7.28-7.20 (m, 2H), 7.06-6.95 (m, 2H), 3.91 (s, 3H), 3.62 (ddd, J = 10.0, 5.5, 2.2 Hz, 1H), 1.36 (dd, J = 14.7, 5.5 Hz, 1H), 1.13 (dd, J = 14.7, 10.0 Hz, 1H), 0.00 (s, 9H). ^{13}C NMR (100 MHz, $CDCl_3$) δ: 201.2, 159.2, 130.0, 129.7, 114.6, 55.5, 54.4, 17.1, -0.9 ppm. **HRMS** calculated $[M+H]^+$ for $C_{13}H_{21}O_2Si$ = 237.1305, found: 237.1301.

(*S*)-2-(4-(tert-butyl)phenyl)-3-(trimethylsilyl)propanal (2f)

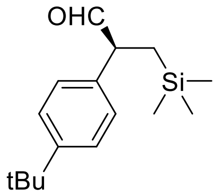

colorless oil; Isolated yield: 85%; 96% ee; $[\alpha]_D^{20}$ = 11.6 (c = 1.0, $CHCl_3$); The enantiomeric excess was determined by HPLC on Chiralpak OD-H column, hexane: isopropanol = 99:1; flow rate = 0.5 mL/min; UV detection at 220 nm; t_R = 10.6 min (minor), 11.1 min (major). ^{1}H NMR (400 MHz, $CDCl_3$) δ: 9.71 (d, J = 2.3 Hz, 1H), 7.51-7.46 (m, 2H), 7.28-7.23 (m, 2H), 3.69-3.62 (m, 1H), 1.42 (s, 9H), 1.41-1.37 (m, 1H), 1.13 (dd, J = 14.7, 9.2 Hz, 1H), 0.00 (s, 9H). ^{13}C NMR (100 MHz, $CDCl_3$) δ: 201.3, 150.8, 134.9, 128.6, 126.1, 54.8, 34.7, 31.6, 17.2, -1.0 ppm. **HRMS** calculated $[M+H]^+$ for $C_{16}H_{27}OSi$ = 263.1826, found: 263.1820.

(*S*)-2-([1,1'-biphenyl]-4-yl)-3-(trimethylsilyl)propanal (2g)

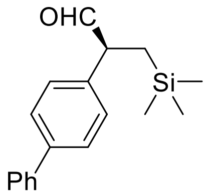

white solid; Isolated yield: 95%; 94% ee; $[\alpha]_D^{20}$ = 9.4 (c = 1.0, $CHCl_3$); The enantiomeric excess was determined by HPLC on Chiralpak AD-H column, hexane: isopropanol = 97:3; flow rate = 0.8 mL/min; UV detection at 210 nm; t_R = 15.4 min (minor), 18.2 min (major). ^{1}H NMR (400 MHz, $CDCl_3$) δ: 9.72 (d, J = 2.2 Hz, 1H), 7.67 (d, J = 8.2 Hz, 4H), 7.55-7.48 (m, 2H), 7.45-7.39 (m, 1H), 7.39-7.33 (m, 2H), 3.69 (ddd, J = 9.6, 5.8, 2.2 Hz, 1H), 1.41 (dd, J = 14.8, 5.8 Hz, 1H), 1.16 (dd, J = 14.8, 9.6 Hz, 1H), 0.00 (s, 9H). ^{13}C NMR (100 MHz, $CDCl_3$) δ: 200.9, 140.7, 140.6, 137.0, 129.4, 129.0, 127.8, 127.6, 127.2, 55.0, 17.2, -0.9 ppm. **HRMS** calculated $[M+H]^+$ for $C_{18}H_{23}OSi$ = 283.1513, found: 283.1505.

(*S*)-2-(4-chlorophenyl)-3-(trimethylsilyl)propanal (2h)

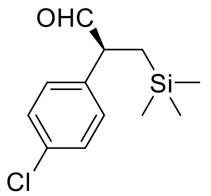

colorless oil; Isolated yield: 97%; 95% ee; $[\alpha]_D^{20}$ = 18.7 (c = 1.0, $CHCl_3$); The enantiomeric excess was determined by HPLC on Chiralpak AD-H column, hexane: isopropanol = 95:5; flow rate = 0.3 mL/min; UV detection at 210 nm; t_R = 21.1 min (minor), 24.3 min (major). ^{1}H NMR (400 MHz, $CDCl_3$) δ: 9.68 (d, J = 2.1 Hz, 1H), 7.47-7.41 (m, 2H), 7.29-7.24 (m, 2H), 3.65 (ddd, J = 9.9, 5.6, 2.1 Hz, 1H), 1.39 (dd, J = 14.8, 5.6 Hz, 1H), 1.11 (dd, J = 14.8, 9.9 Hz, 1H), 0.00 (s, 9H). ^{13}C NMR (100 MHz, $CDCl_3$) δ: 200.5, 136.5, 133.7, 130.3, 129.3, 54.6, 17.2, -0.9 ppm. **HRMS** calculated $[M-H]^+$ for $C_{12}H_{16}OClSi$ = 239.0664, found: 239.0662.

(*S*)-2-(4-fluorophenyl)-3-(trimethylsilyl)propanal (2i)

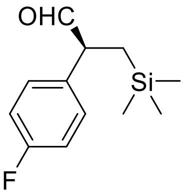

yellow oil; Isolated yield: 96%; 95% ee; $[\alpha]_D^{20}$ = 17.6 (c = 1.0, $CHCl_3$); The enantiomeric excess was determined by HPLC on Chiralpak AD-H column, hexane: isopropanol = 97:3; flow rate = 0.5 mL/min; UV detection at 210 nm; t_R = 16.6 min (minor), 19.4 min (major). ^{1}H NMR (400 MHz, $CDCl_3$) δ: 9.70 (d, J = 2.2 Hz, 1H), 7.33-7.26 (m, 2H), 7.20-7.12 (m, 2H), 3.67 (ddd, J = 10.0, 5.5, 2.1 Hz, 1H), 1.39 (dd, J = 14.8, 5.5 Hz, 1H), 1.12 (dd, J = 14.8, 10.0 Hz, 1H), 0.00 (s, 9H). ^{13}C NMR (100 MHz, $CDCl_3$) δ: 200.8 (d, J = 1.1 Hz), 162.4 (d, J = 246.3 Hz), 133.7 (d, J = 3.2 Hz), 130.5 (d, J = 8.1 Hz), 116.1 (d, J = 21.4 Hz), 54.5, 17.4, -1.0 ppm. **HRMS** calculated $[M-H]^+$ for $C_{12}H_{16}OFSi$ = 223.0960, found: 223.0955.

(*S*)-2-(4-(trifluoromethyl)phenyl)-3-(trimethylsilyl)propanal (2j)

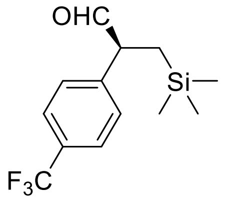

colorless oil; Isolated yield: 98%; 93% ee; $[\alpha]_D^{20}$ = 9.9 (c = 1.0, $CHCl_3$); The enantiomeric excess was determined by HPLC on Chiralpak AD-H column, hexane: isopropanol = 95:5; flow rate = 0.3 mL/min; UV detection at 210 nm; t_R = 18.1 min (minor), 21.4 min (major). ^{1}H NMR (400 MHz, $CDCl_3$) δ: 9.72 (d, J = 2.1 Hz, 1H), 7.72 (d, J = 8.2 Hz, 2H), 7.45 (d, J = 8.2 Hz, 2H), 3.75 (ddd, J = 9.6, 5.8, 2.0 Hz, 1H), 1.44 (dd, J = 14.8, 5.8 Hz, 1H), 1.15 (dd, J = 14.8, 9.6 Hz, 1H), 0.00 (s, 9H). ^{13}C NMR (100 MHz, $CDCl_3$) δ: 200.2, 142.3, 130.1 (q, J = 32.5 Hz), 129.4 (s), 126.1 (q, J = 3.8 Hz), 124.3 (q, J = 272.0 Hz), 55.2, 17.4, -1.0 ppm. **HRMS** calculated $[M+H]^+$ for $C_{13}H_{18}OF_3Si$ = 275.1074, found: 275.1066.

(*S*)-2-(m-tolyl)-3-(trimethylsilyl)propanal (2k)

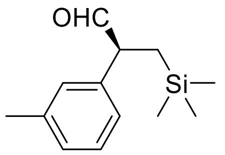

colorless oil; Isolated yield: 94%; 93% ee; $[\alpha]_D^{20}$ =16.8 (c = 1.0, $CHCl_3$); The enantiomeric excess was determined by HPLC on Chiralpak OD-H column, hexane: isopropanol = 97:;3; flow rate = 0.5 mL/min; UV detection at 210 nm; t_R = 14.1 min (major), 14.5 min (minor). ^{1}H NMR (400 MHz, $CDCl_3$) δ: 9.70 (d, J = 2.2 Hz, 1H), 7.37-7.32 (m, 1H), 7.21-7.19 (m, 1H), 7.13-7.11 (m, 2H), 3.63 (ddd, J = 9.4, 5.8, 2.2 Hz, 1H), 2.45 (s, 3H), 1.39 (dd, J = 14.8, 5.8 Hz, 1H), 1.14 (dd, J = 14.8, 9.4 Hz, 1H), 0.00 (s, 9H). ^{13}C NMR (100 MHz, $CDCl_3$) δ: 201.2, 138.9, 137.9, 129.7, 129.1, 128.5, 126.1, 55.3, 21.6, 17.1, -1.0 ppm. **HRMS** calculated $[M+H]^+$ for $C_{13}H_{21}OSi$ = 221.1356, found: 221.1352.

(*S*)-2-(3-fluorophenyl)-3-(trimethylsilyl)propanal (2l)

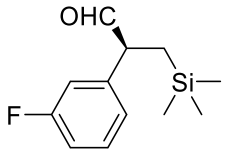

colorless oil; Isolated yield: 97%; 90% ee; $[\alpha]_D^{20}$ = 13.0 (c = 2.0, $CHCl_3$); The enantiomeric excess was determined by HPLC on Chiralpak AD-H column, hexane: isopropanol = 97:3; flow rate = 0.5 mL/min; UV detection at 210 nm; t_R = 16.6 min (minor), 17.8 min (major). ^{1}H NMR (400 MHz, $CDCl_3$) δ: 9.69 (d, *J* = 2.2 Hz, 1H), 7.42 (td, *J* = 8.0, 6.0 Hz, 1H), 7.14-7.00 (m, 3H), 3.66 (ddd, *J* = 9.7, 5.7, 2.2 Hz, 1H), 1.39 (dd, *J* = 14.8, 5.7 Hz, 1H), 1.12 (dd, *J* = 14.8, 9.7 Hz, 1H), 0.00 (s, 9H). ^{13}C NMR (100 MHz, $CDCl_3$) δ: 200.4, 163.3 (d, *J* = 246.9 Hz). 140.6 (d, *J* = 7.1 Hz), 130.7 (d, *J* = 8.3 Hz), 124.7 (d, *J* = 2.9 Hz), 115.8 (d, *J* = 21.6 Hz), 114.8 (d, *J* = 21.0 Hz), 55.0 (d, *J* = 1.5 Hz), 17.2, -1.0 ppm. **HRMS** calculated $[M-H]^+$ for $C_{12}H_{16}OFSi$ = 223.0960, found: 223.0956.

(*S*)-2-(2-fluorophenyl)-3-(trimethylsilyl)propanal (2m)

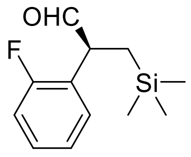

colorless oil; Isolated yield: 90%; 95% ee; $[\alpha]_D^{20}$ = 9.9 (c = 1.0, $CHCl_3$); The enantiomeric excess was determined by HPLC on Chiralpak AD-H column, hexane: isopropanol = 97:3; flow rate = 0.5 mL/min; UV detection at 210 nm; t_R = 17.8 min (minor), 20.0 min (major). ^{1}H NMR (400 MHz, $CDCl_3$) δ: 9.78 (t, *J* = 1.4 Hz, 1H), 7.42-7.35 (m, 1H), 7.31-7.22 (m, 2H), 7.22-7.16 (m, 1H), 3.97 (ddd, *J* = 10.4, 5.2, 1.2 Hz, 1H), 1.45 (dd, *J* = 14.8, 5.2 Hz, 1H), 1.16 (ddd, *J* = 14.8, 10.4, 0.8 Hz, 1H), 0.00 (s, 9H). ^{13}C NMR (100 MHz, $CDCl_3$) δ 200.3 (d, *J* = 0.8 Hz), 161.1 (d, *J* = 245.9 Hz), 130.4 (d, *J* = 4.4 Hz), 129.5 (d, *J* = 8.3 Hz), 125.5 (d, *J* = 15.1 Hz), 124.8 (d, *J* = 3.6 Hz), 116.1 (d, *J* = 22.4 Hz), 48.3, 16.3 (d, *J* = 0.9 Hz), -1.2 ppm. **HRMS** calculated $[M-H]^+$ for $C_{12}H_{16}OFSi$ = 223.0960, found: 223.0956.

(*S*)-2-(3,4,5-trifluorophenyl)-3-(trimethylsilyl)propanal (2n)

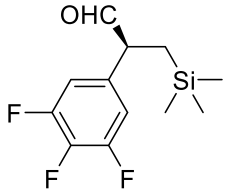

colorless oil; Isolated yield: 98%; 92% ee; $[\alpha]_D^{20}$ = 12.2 (c = 1.0, $CHCl_3$); The enantiomeric excess was determined by HPLC on Chiralpak AD-H column, hexane: isopropanol = 97:3; flow rate = 0.5 mL/min; UV detection at 210 nm; t_R = 14.7 min (minor), 15.2 min (major). ^{1}H NMR (400 MHz, $CDCl_3$) δ: 9.62 (d, *J* = 2.0 Hz, 1H), 6.93 (dd, *J* = 8.2, 6.4 Hz, 2H), 3.58 (ddd, *J* = 9.6, 5.9, 2.0 Hz, 1H), 1.35 (dd, *J* = 14.8, 5.9 Hz, 1H), 1.02 (dd, *J* = 14.8, 9.6 Hz, 1H), 0.00 (s, 9H). ^{13}C NMR (100 MHz, $CDCl_3$) δ: 199.4, 151.6 (ddd, *J* = 251.1, 10.0, 4.0 Hz), 139.3 (dt, *J* = 251.9, 15.3 Hz), 134.6 (td, *J* = 7.0, 4.8 Hz), 113.0 (dd, *J* = 15.7, 5.8 Hz), 54.4, 17.2, -1.0 ppm. **HRMS** calculated $[M-H]^+$ for $C_{12}H_{14}OF_3Si$ = 259.0771, found: 259.0770.

(*S*)-2-(naphthalen-2-yl)-3-(trimethylsilyl)propanal (2o)

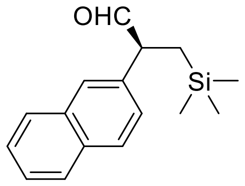

yellow oil; Isolated yield: 98%; 93% ee; $[\alpha]_D^{20}$ = 19.0 (c = 1.0, $CHCl_3$); The enantiomeric excess was determined by HPLC on Chiralpak AD-H column, hexane: isopropanol = 99:1; flow rate = 1.0 mL/min; UV detection at 210 nm; t_R = 23.5 min (minor), 33.7 min (major). ^{1}H NMR (400 MHz, $CDCl_3$) δ: 9.77 (d, J = 2.2 Hz, 1H), 7.94-7.90 (m, 3H), 7.79 (d, J = 1.1 Hz, 1H), 7.62-7.52 (m, 2H), 7.41 (dd, J = 8.6, 1.8 Hz, 1H), 3.83 (ddd, J = 9.4, 5.8, 2.2 Hz, 1H), 1.49 (dd, J = 14.8, 5.8 Hz, 1H), 1.26 (dd, J = 14.8, 9.4 Hz, 1H), -0.00 (s, 9H). ^{13}C NMR (100 MHz, $CDCl_3$) δ 201.0, 135.4, 133.8, 132.9, 129.0, 128.0, 127.9, 127.9, 126.7, 126.6, 126.3, 55.4, 17.1, -0.9 ppm. **HRMS** calculated $[M+H]^+$ for $C_{16}H_{21}OSi$ = 257.1356, found: 257.1351.

(*S*)-2-(furan-2-yl)-3-(trimethylsilyl)propanal (2p)

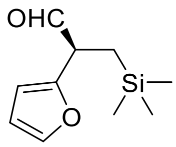

colorless oil; Isolated yield: 94%; 91% ee; $[\alpha]_D^{20}$ = 15.5 (c = 1.0, $CHCl_3$); The enantiomeric excess was determined by HPLC on Chiralpak AD-H column, hexane: isopropanol = 99:1; flow rate = 1.0 mL/min; UV detection at 210 nm; t_R = 12.0 min (minor), 13.1 min (major).

(*S*)-2-(thiophen-3-yl)-3-(trimethylsilyl)propanal (2q)

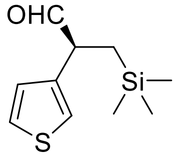

colorless oil; Isolated yield: 89%; 93% ee; $[\alpha]_D^{20}$ = 14.7 (c = 1.0, $CHCl_3$); The enantiomeric excess was determined by HPLC on Chiralpak OD-H column, hexane: isopropanol = 99:1; flow rate = 1.0 mL/min; UV detection at 230 nm; t_R = 19.3 min (major), 20.7 min (minor). ^{1}H NMR (400 MHz, $CDCl_3$) δ: 9.65 (d, J = 2.4 Hz, 1H), 7.43 (dd, J = 5.0, 2.9 Hz, 1H), 7.21 (ddd, J = 2.9, 1.2, 0.4 Hz, 1H), 7.06 (dd, J = 5.0, 1.2 Hz, 1H), 3.79 (ddd, J = 10.0, 5.5, 2.4 Hz, 1H), 1.32 (dd, J = 14.7, 5.5 Hz, 1H), 1.13 (dd, J = 14.7, 10.0 Hz, 1H), 0.00 (s, 9H). ^{13}C NMR (100 MHz, $CDCl_3$) δ: 200.4, 138.3, 127.5, 126.8, 122.9, 50.5, 17.1, -1.1 ppm. **HRMS** calculated $[M+H]^+$ for $C_{10}H_{17}OSSi$ = 213.0764, found: 213.0759.

(*S*)-3-(dimethyl(phenyl)silyl)-2-methylpropanal (2r)

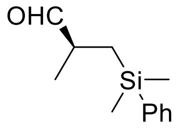

colorless oil; Isolated yield: 94%; 94% ee; $[\alpha]_D^{20}$ = 9.2 (c = 1.0, $CHCl_3$); The enantiomeric excess was determined by HPLC on Chiralpak AD-H column, hexane: isopropanol = 99:1; flow rate = 1.5

mL/min; UV detection at 210 nm; t_R = 11.4 min (minor), 12.0 min (major).^{1}H NMR (400 MHz, $CDCl_3$) δ: 9.42 (d, *J* = 1.7 Hz, 1H), 7.46-7.40 (m, 2H), 7.30-7.25 (m, 3H), 2.33-2.24 (m, 1H), 1.15 (dd, *J* = 14.9, 5.4 Hz, 1H), 0.97 (d, *J* = 7.0 Hz, 3H), 0.64 (dd, *J* = 14.9, 8.8 Hz, 1H), 0.25 (s, 6H). ^{13}C NMR (100 MHz, $CDCl_3$) δ: 204.9, 138.7, 133.7, 129.4, 128.2, 42.7, 17.0, 16.4, -2.0, -2.0 ppm. **HRMS** calculated $[M+H]^+$ for $C_{12}H_{19}OSi$ = 207.1200, found: 207.1195.

(*S*)-3-(benzyldimethylsilyl)-2-methylpropanal (2s)

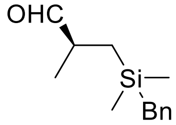

colorless oil; Isolated yield: 92%; 94% ee; $[\alpha]_D^{20}$ = 12.9 (c = 1.0, $CHCl_3$); The enantiomeric excess was determined by HPLC on Chiralpak OD-H column, hexane: isopropanol = 99:1; flow rate = 1.0 mL/min; UV detection at 210 nm; t_R = 30.8 min (major), 37.4 min (minor). ^{1}H NMR (400 MHz, $CDCl_3$) δ 9.48: (d, *J* = 1.8 Hz, 1H), 7.22-7.15 (m, 2H), 7.05 (t, *J* = 7.4 Hz, 1H), 6.96 (d, *J* = 7.0 Hz, 2H), 2.38-2.26 (m, 1H), 2.09 (s, 2H), 1.07 (d, *J* = 7.0 Hz, 3H), 0.96 (dd, *J* = 14.8, 5.6 Hz, 1H), 0.46 (dd, *J* = 14.8, 8.8 Hz, 1H), 0.00 (s, 6H). ^{13}C NMR (100 MHz, $CDCl_3$) δ: 204.9, 139.9, 128.5, 128.3, 124.4, 42.6, 26.3, 16.5, 15.9, -2.4, -2.7 ppm. **HRMS** calculated $[M+H]^+$ for $C_{13}H_{21}OSi$ = 221.1356, found: 221.1351.

(*S*)-2-methyl-3-(methyldiphenylsilyl)propanal (2t)

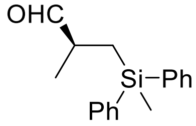

colorless oil; Isolated yield: 92%; 93% ee; $[\alpha]_D^{20}$ = 6.4 (c = 1.0, $CHCl_3$); The enantiomeric excess was determined by HPLC on Chiralpak AD-H column, hexane: isopropanol = 99:1; flow rate = 1.5 mL/min; UV detection at 210 nm; t_R = 19.7 min (minor), 22.4 min (major). ^{1}H NMR (400 MHz, $CDCl_3$) δ: 9.42 (d, *J* = 1.4 Hz, 1H), 7.45-7.40 (m, 4H), 7.31-7.23 (m, 6H), 2.41-2.27 (m, 1H), 1.50 (dd, *J* = 15.0, 4.7 Hz, 1H), 0.98-0.90 (m, 4H), 0.53 (s, 3H). ^{13}C NMR (100 MHz, $CDCl_3$) δ: 204.7, 136.8, 136.6, 134.6, 134.6, 129.7, 129.7, 128.2, 128.2, 42.6, 16.5, 15.3, -3.3 ppm. **HRMS** calculated $[M+H]^+$ for $C_{17}H_{21}OSi$ = 269.1356, found: 269.1351.

(*S*)-2-((dimethyl(phenyl)silyl)methyl)-4-phenylbutanal (2u)

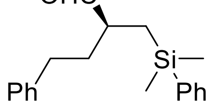

colorless oil; Isolated yield: 88%; 95% ee; $[\alpha]_D^{20}$ = 8.0 (c = 0.5, $CHCl_3$); The enantiomeric excess was determined by HPLC on Chiralpak OD-H column, hexane: isopropanol = 99:1; flow rate = 1.0 mL/min; UV detection at 220 nm; t_R = 63.5 min (minor), 66.5 min (major). ^{1}H NMR (400 MHz, $CDCl_3$) δ: 9.39 (d, *J* = 2.6 Hz, 1H), 7.44-7.35 (m, 2H), 7.32-7.23 (m, 3H), 7.20-7.12 (m, 2H), 7.11-7.05 (m, 1H), 7.01-6.91 (m, 2H), 2.56-2.34 (m, 2H), 2.31-2.24 (m, 1H), 1.90-1.73 (m, 1H), 1.66-1.54 (m, 1H), 1.04 (dd, *J* = 14.9, 7.0 Hz, 1H), 0.78 (dd, *J* = 14.9, 7.1 Hz, 1H), 0.21 (s, 6H). ^{13}C NMR

(100 MHz, $CDCl_3$) δ: 204.6, 141.5, 138.6, 133.8, 129.4, 128.6, 128.6, 128.2, 126.2, 47.5, 33.4, 33.3, 15.3, -1.9, -2.3 ppm. **HRMS** calculated $[M+H]^+$ for $C_{19}H_{25}OSi$ = 297.1669, found: 297.1663.

Procedure for the synthesis of compound 5

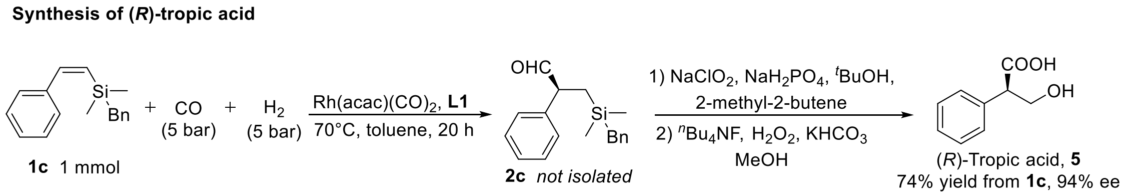

The step of the aldehyde oxidation is following the reference 5 and the Fleming-Tamao oxidation step is following the reference 4.

Procedure for the synthesis of (*R*)-tropic acid 5: In a glovebox filled with nitrogen, to a 10 ml vial equipped with a magnetic bar was added ligand **L1** (0.015 mmol) and $Rh(acac)(CO)_2$ (0.005 mmol in 1 mL solvent). After stirring for 10 min, substrate **1c** (252 mg, 1.0 mmol) and additional solvent was charged to bring the total volume of the reaction mixture to 4.0 mL. The vial was transferred into an autoclave and taken out of the glovebox. Carbon monoxide (5 bar) and hydrogen (5 bar) were charged in sequence. The reaction mixture was stirred at 70 ℃ (oil bath) for 20 h. The reaction was cooled and the pressure was carefully released in a well-ventilated hood. The reaction mixture was transferred into a 20 mL Schlenk tube, then *t*-BuOH (10 mL), 2-methyl-2-butene (2.0 M in THF, 5.5 mL, 11 mmol), and NaH_2PO_4 (276 mg, 1.77 mmol) in H_2O (2.0 mL) were added. The mixture was cooled to 0 ℃, then $NaClO_2$ (994 mg, 11.0 mmol) in 2mL of H_2O was added. After being stirred for 30 min at room temperature. The reaction mixture was poured into saturated aq NH_4Cl (5 mL), and whole was extracted with EtOAc (5 mL).The combined organic layers were washed with brine (5mL) and dried over anhydrous Na_2SO_4. Filtration and evaporation in vacuo furnished the crude product, the crude product was used directly in next step.

A THF solution of tetrabutylanmonium fluoride (1.0 M, 3.00 mL, 3.00 mmol) was added to a solution of the crude product obtained in last step in THF (5.0 mL) with stirring at room temperature. After 30 min, $KHCO_3$ (300 mg, 3.0 mmol), MeOH (6.00 mL) and 30% H_2O_2 (3.00 mL) were successively added to the reaction mixture. After 30 min, the reaction mixture was diluted with water, extracted with three times of EtOAc, dried over anhydrous Na_2SO_4. Filtration and evaporation in vacuo furnished the crude product. The crude product was purified by flash chromatography on silica gel to give the product **5** (123 mg, 74% yield from **1c**) as a white solid, 94% ee, $[\alpha]_D^{25}$ = 55.8 (c = 0.5, acetone); lit. 6 $[\alpha]_D^{25}$ = -63.3 (c = 0.3, acetone, *S*-isomer). ^1H NMR (400 MHz, DMSO) δ: 12.39 (s, 1H), 7.44-7.19 (m, 5H), 4.94 (s, 1H), 3.92 (t, *J* = 9.4 Hz, 1H), 3.64 (dd, *J* = 8.7, 5.6 Hz, 1H), 3.56 (dd, *J* = 10.0, 5.7 Hz, 1H). ^{13}C NMR (100 MHz, DMSO) δ: 173.8, 137.1, 128.5, 128.1, 127.2, 63.5, 54.4 ppm. The enantiomeric excess of **5** was determined by HPLC on Chiralpak AD-H column after esterification with CH_2N_2. Conditions: hexane: isopropanol = 95:5; flow rate = 1.0 mL/min; UV detection at 210 nm; t_R = 15.2 min (minor), 16.3 min (major).

Deuterium Labeling Studies

Asymmetric hydroformylation of **1a** in toluene solution under D_2: In a glovebox filled with nitrogen, to a 5 ml vial equipped with a magnetic bar was added ligand **L1** (0.015 mmol) and $Rh(acac)(CO)_2$ (0.005 mmol in 0.5 mL solvent). After stirring for 10 min, substrate (0.5 mmol) and additional

solvent was charged to bring the total volume of the reaction mixture to 1.0 mL. The vial was transferred into an autoclave and taken out of the glovebox. Carbon monoxide (5 bar) and D_2 (5 bar) were charged in sequence. The reaction mixture was stirred at 70 ℃ (oil bath) for 20 h. The reaction was cooled and the pressure was carefully released in a well-ventilated hood. The crude product was purified by flash chromatography on silica gel to give the product.

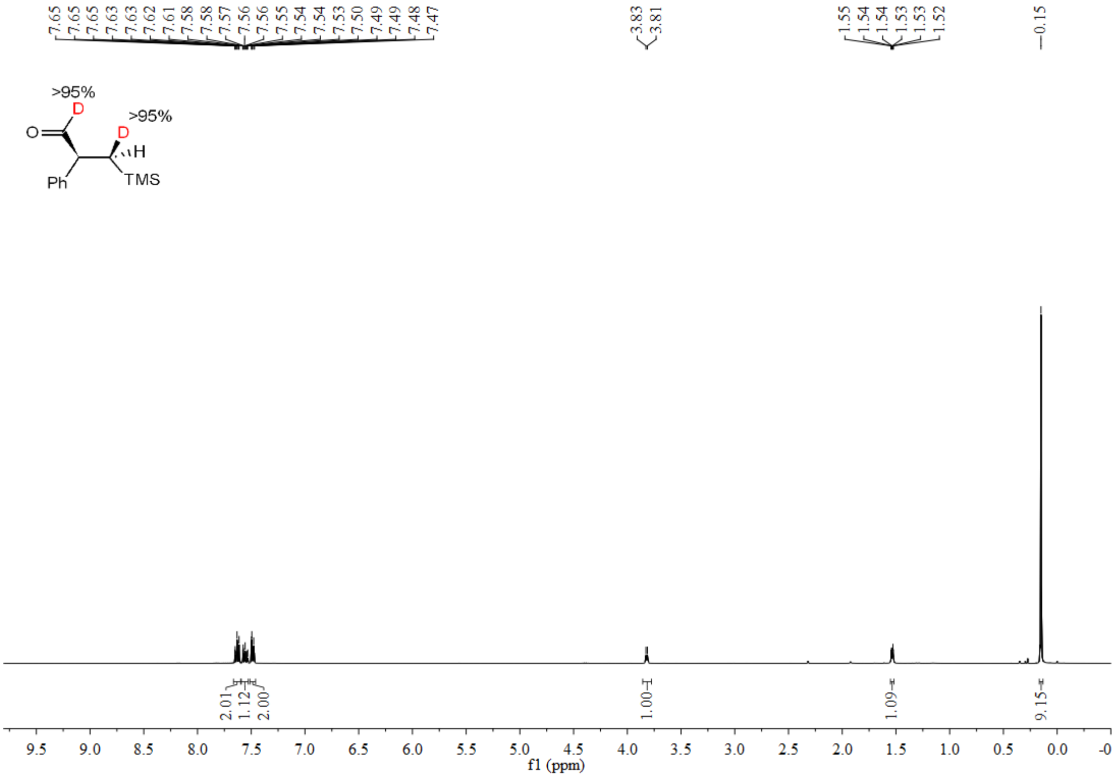

Supplementary Figure 1. ^{1}H NMR (400 MHz, $CDCl_3$) spectra for compound 2a. AHF of 1a under 5 bar CO and 5 bar D_2

Supplementary Table 1. The the effects of syngas pressure on the isomerization of 1a

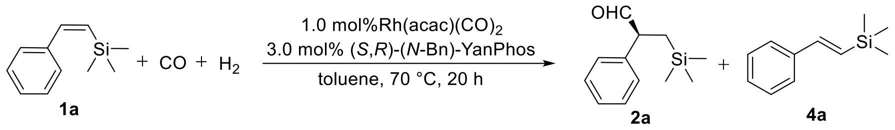

Entry	CO/H_2 (bar)	Conv. (%)	Yield (%)		**4a**/(**2a**+**4a**) (%)
			**2a**	**4a**	
1[a]	5/5	1	1	99	-
2[b]	5/5	46	46	trace	-
3	5/5	>99	>99	0	0
4	5/10	>99	>99	0	0
5	5/15	>99	>99	0	0
6	10/10	93	88	5	5.4
7	15/15	81	76	5	6.2
8	20/20	78	69	9	11.5
9	30/30	54	46	8	14.8
10	10/5	93	91	2	2.2
11	12.5/5	83	77	6	7.2
12	15/5	79	71	8	10.1
13	25/5	58	48	10	17.2

Conditions: **1a** (0.5 mmol), Rh(acac)$(CO)_2$ (1.0 mol%), (*S*,*R*)-(*N*-Bn)-YanPhos (3.0 mol%), toluene (2 ml), 70 ℃, 20 h. Conversions and yields were determined by ^{1}H NMR analysis. [a]Using **4a** instead of **1a**. [b]The reaction time was 3.5 hours.

Computational Details

Density functional theory (DFT) calculations were carried out to understand the alkene insertion step which is regioselectivity and enantioselectivity determining step in Rh-catalyzed asymmetric hydroformylation of (*S*,*R*)-(*N*-Bn)-YanPhos. M06[7] method combined with 6-31G* basis set[8,9,10]was used to fully optimize all structures in gas phase. Vibrational frequency calculations were computed on the optimized structures at the same level of theory to check whether every optimized structure is either a local minimum or a transition state. The effect of the solvent (toluene) was then included by single-point calculations with an implicit solvent model SMD[11] (by the M06 method). The most critical points were also re-optimized by B3LYP-D3/6-31G* method[12,13,14,15] along with the subsequent frequency and solvent calculations at the same level. All the computations were carried out by Gaussian 09 package[16]. All 3D images of the optimized structures were illustrated by CYLview[17].

Supplementary Table 2. The absolute (in Hartree) and relative (in kcal/mol) energies for three model substrates catalyzed by (*S*,*R*)-(*N*-Bn)-YanPhos-Rh in gas phase by M06/6-31G* method.

		E	E+ZPE	G	ΔE_{gas}	$\Delta E_{gas+ZPE}$	ΔG_{gas}
PhCH=CHTMS							
PhCH=CHTMS		-717.933362	-717.698733	-717.74031	-	-	-
CO		-113.251769	-113.246701	-113.265838	-	-	-
I-1		-3496.891558	-3496.038592	-3496.130538	0.0	0.0	0.0
I-2		-3496.88788	-3496.034124	-3496.125172	2.3	2.8	3.4
I-3		-3496.888286	-3496.035317	-3496.126826	2.1	2.1	2.3
I-4		-3496.885025	-3496.030969	-3496.122509	4.1	4.8	5.0
II-1		-3383.603811	-3382.759711	-3382.850842	22.6	20.2	8.7
II-2		-3383.606664	-3382.76185	-3382.851083	20.8	18.9	8.5
III		-4101.560532	-4100.474609	-4100.57501	7.9	10.0	18.8
$TSI_{TMS\text{-}\beta1}$	S	-4101.544806	-4100.462355	-4100.565124	17.8	17.7	25.0
	R	-4101.542983	-4100.462079	-4100.566733	18.9	17.9	24.0
$TSI_{TMS\text{-}\beta2}$	R	-4101.531333	-4100.451019	-4100.554559	26.2	24.9	31.7
$TSI_{TMS\text{-}\alpha1}$	S	-4101.538321	-4100.455872	-4100.557708	21.9	21.8	29.7
	R	-4101.543759	-4100.46054	-4100.56212	18.4	18.9	26.9
$TSI_{TMS\text{-}\alpha2}$	R	-4101.532743	-4100.4518	-4100.555296	25.4	24.4	31.2
$TSI_{TMS\text{-}\beta3}$	R	-4101.532573	-4100.452082	-4100.554986	25.5	24.2	31.4
$TSI_{TMS\text{-}\alpha3}$	R	-4101.533396	-4100.452361	-4100.55528	24.9	24.0	31.2
IV		-4101.58296	-4100.49752	-4100.6011	-6.2	-4.3	2.5
V		-4214.864463	-4213.76998	-4213.874381	-24.8	-20.5	-2.2

**TSII$_{TMS\text{-}\beta 1R}$**		-4214.831615	-4213.738429	-4213.844907	-4.2	-0.7	16.3
**VI**		-4214.869115	-4213.774269	-4213.881446	-27.7	-23.2	-6.7
**VII**		-4216.038566	-4214.925681	-4215.03416	-29.5	-19.8	3.1
**TSIII$_{TMS\text{-}\beta 1R}$**		-4216.021415	-4214.911513	-4215.018286	-18.7	-11.0	13.0
**VIII**		-4216.035484	-4214.922961	-4215.032007	-27.5	-18.1	4.4
**TSIV$_{TMS\text{-}\beta 1R}$**		-4216.027929	-4214.917032	-4215.023613	-22.8	-14.4	9.7
**TSIV'$_{TMS\text{-}\beta 1R}$**		-4216.012201	-4214.902289	-4215.010754	-12.9	-5.2	17.8
**IX**		-4216.051102	-4214.935199	-4215.042875	-37.3	-25.8	-2.4
**IX'**		-4216.024488	-4214.909591	-4215.017162	-20.6	-9.8	13.7
**TSV$_{TMS\text{-}\beta 1R}$**		-4216.009819	-4214.897792	-4215.005178	-11.4	-2.3	21.3
**PhCH=CHtBu**							
**PhCH=CHtBu**		-466.515544	-466.268982	-466.306364	0.0	0.0	0.0
**TSI$_{tBu\text{-}\beta 1}$**	**S**	-3850.121555	-3849.027979	-3849.128046	21.2	20.6	27.0
	**R**	-3850.121742	-3849.028789	-3849.12992	21.1	20.1	25.8
**IV$_{tBu\text{-}\beta 1R}$**		-3850.164067	-3849.067219	-3849.17003	-5.5	-4.0	0.6
**TSI$_{tBu\text{-}\alpha 1}$**	**S**	-3850.115239	-3849.023389	-3849.124085	25.2	23.5	29.5
	**R**	-3850.115162	-3849.019725	-3849.117138	25.2	25.8	33.8

Supplementary Table 3. The absolute (in Hartree) and relative (in kcal/mol) energies for three model substrates catalyzed by (S,R)-(N-Bn)-Yanphos-Rh in toluene solvent by SMD M06/6-31G* method.

		E_{solv}	ΔE_{solv}	ΔG_{solv}
**PhCH=CHTMS**				
**PhCH=CHTMS**		-717.942723	-	-
**CO**		-113.247314	-	-
**I-1**		-3496.941605	0.0	0.0
**I-2**		-3496.937322	2.7	3.7
**I-3**		-3496.937565	2.5	2.8
**I-4**		-3496.933821	4.9	5.8
**II-1**		-3383.657123	23.3	11.1
**II-2**		-3383.655045	24.6	10.7
**III**		-4101.615649	13.4	24.3
**TSI$_{TMS\text{-}\beta 1}$**	**S**	-4101.598307	24.3	31.5
	**R**	-4101.598615	24.1	**29.2**
**TSI$_{TMS\text{-}\alpha 1}$**	**S**	-4101.592167	28.1	36.0
	**R**	-4101.599072	23.8	**32.3**
**TSI$_{TMS\text{-}\beta 2}$**	**R**	-4101.588322	30.6	36.0
**TSI$_{TMS\text{-}\alpha 2}$**	**R**	-4101.589322	29.9	35.8
**TSI$_{TMS\text{-}\beta 3}$**	**R**	-4101.588398	30.5	36.4
**TSI$_{TMS\text{-}\alpha 3}$**	**R**	-4101.589242	30.0	36.2
**TSI$_{TMS\text{-}\beta 4}$**	**R**	?	?	?
**TSI$_{TMS\text{-}\alpha 4}$**	**R**	?	?	?
**IV**		-4101.639658	-1.7	7.0

**V**		-4214.921423	-23.3	-0.7
**TSII$_{TMS\text{-}\beta1R}$**		-4214.88939	-3.2	17.3
**VI**		-4214.926959	-26.8	-5.7
**VII**		-4216.097583	-29.6	3.0
**TSIII$_{TMS\text{-}\beta1R}$**		-4216.07947	-18.2	13.5
**VIII**		-4216.094034	-27.3	4.6
**TSIV$_{TMS\text{-}\beta1R}$**		-4216.084827	-21.6	10.9
**TSIV'$_{TMS\text{-}\beta1R}$**		-4216.072119	-13.6	17.1
**IX**		-4216.110405	-37.6	-2.7
**IX'**		-4216.085867	-22.2	12.2
**TSV$_{TMS\text{-}\beta1R}$**		-4216.069652	-12.0	20.6
**PhCH=CHtBu**				
**PhCH=CHtBu**		-466.52691		
**TSI$_{tBu\text{-}\beta1}$**	**S**	-3850.177712	27.3	33.1
	**R**	-3850.17892	26.5	**31.3**
**IV$_{tBu\text{-}\beta1R}$**		-3850.222999	-1.1	5.0
**TSI$_{tBu\text{-}\alpha1}$**	**S**	-3850.17007	32.1	**36.4**
	**R**	-3850.17134	31.3	39.9
**PhCH=CHMe**				
**PhCH=CHMe**		-348.686043		
**III$_{Me\text{-}\beta1S}$**		-3732.354628	16.1	22.9
**TSI$_{Me\text{-}\beta1}$**	**S**	-3732.334674	28.7	**30.9**
	**R**	-3732.336246	27.7	34.4
**IV$_{Me\text{-}\beta1S}$**		-3732.372009	5.2	11.6
**TSI$_{Me\text{-}\alpha1}$**	**S**	-3732.333628	29.3	**34.4**
	**R**	-3732.334963	28.5	35.6

Supplementary Table 4. The absolute (in Hartree) and relative (in kcal/mol) single-point energies for three model substrates catalyzed by (*S*,*R*)-(*N*-Bn)-YanPhos-Rh in gas phase and in toluene solvent (with SMD model) by B3LYP-GD3/6-31G* method.

		E_{gas}	ΔE_{gas}	ΔG_{gas}	E_{SMD}	ΔE_{SMD}	ΔG_{SMD}
**PhCH=CHTMS**							
**PhCH=CHTMS**		-718.341313	-	-	-718.350419	-	-
**CO**		-113.309454	-	-	-113.304988	-	-
**I-1**		-3499.08831	0.0	0.0	-3499.137786	0.0	0.0
**TSI$_{TMS\text{-}\beta1}$**	**S**	-4104.089364	19.3	26.6	-4104.142035	25.8	33.1
	**R**	-4104.085474	21.8	26.9	-4104.140317	26.9	32.0
**TSI$_{TMS\text{-}\alpha1}$**	**S**	-4104.082917	23.4	31.2	-4104.135949	29.7	37.5
	**R**	-4104.084842	22.2	30.6	-4104.139342	27.5	36.0
**TSI$_{TMS\text{-}\beta2}$**	**R**	-4104.073042	29.6	35.0	-4104.129089	34.0	39.4
**TSI$_{TMS\text{-}\alpha2}$**	**R**	-4104.076077	27.7	33.5	-4104.131685	32.3	38.2
**TSI$_{TMS\text{-}\beta3}$**	**R**	-4104.079166	25.7	31.7	-4104.134148	30.8	36.7

**TSI$_{\text{TMS-}\alpha 3}$**	**R**	-4104.078353	26.2	32.5	-4104.133351	31.3	37.6
**IV**		-4104.122123	-1.2	7.4	-4104.178036	3.3	11.9
**V**		-4217.465519	-22.5	0.1	-4217.521677	-21.0	1.6
**TSII$_{\text{TMS-}\beta\text{1R}}$**		-4217.430476	-0.5	19.9	-4217.487468	0.5	20.9
**VI**		-4217.465806	-22.7	-1.6	-4217.522815	-21.7	-0.6
**VII**		-4218.640939	-22.5	10.1	-4218.699034	-22.5	10.0
**TSIII$_{\text{TMS-}\beta\text{1R}}$**		-4218.631543	-16.6	15.1	-4218.688723	-16.1	15.7
**VIII**		-4218.651981	-29.4	2.5	-4218.709655	-29.2	2.8
**TSIV$_{\text{TMS-}\beta\text{1R}}$**		-4218.640599	-22.3	10.2	-4218.696575	-21.0	11.5
**TSIV'$_{\text{TMS-}\beta\text{1R}}$**		-4218.622394	-10.8	19.8	-4218.681438	-11.5	19.2
**IX**		-4218.656536	-32.3	2.7	-4218.71493	-32.5	2.4
**IX'**		-4218.629317	-15.2	19.2	-4218.689725	-16.7	17.7
**TSV$_{\text{TMS-}\beta\text{1R}}$**		-4218.620029	-9.4	23.3	-4218.678987	-9.9	22.7
**PhCH=CHtBu**							
**PhCH=CHtBu**		-466.921038	0.0	0.0	-466.932196	0.0	0.0
**TSI$_{\text{tBu-}\beta 1}$**	**S**	-3852.661672	24.0	29.8	-3852.717009	30.1	35.9
	**R**	-3852.661581	24.0	28.8	-3852.717973	29.5	34.2
**IV$_{\text{tBu-}\beta\text{1R}}$**		-3852.6997	0.1	6.2	-3852.75787	4.5	10.6
**TSI$_{\text{tBu-}\alpha 1}$**	**S**	-3852.656739	27.1	31.4	-3852.710791	34.0	38.3
	**R**	-3852.653474	29.1	37.8	-3852.708858	35.2	43.9

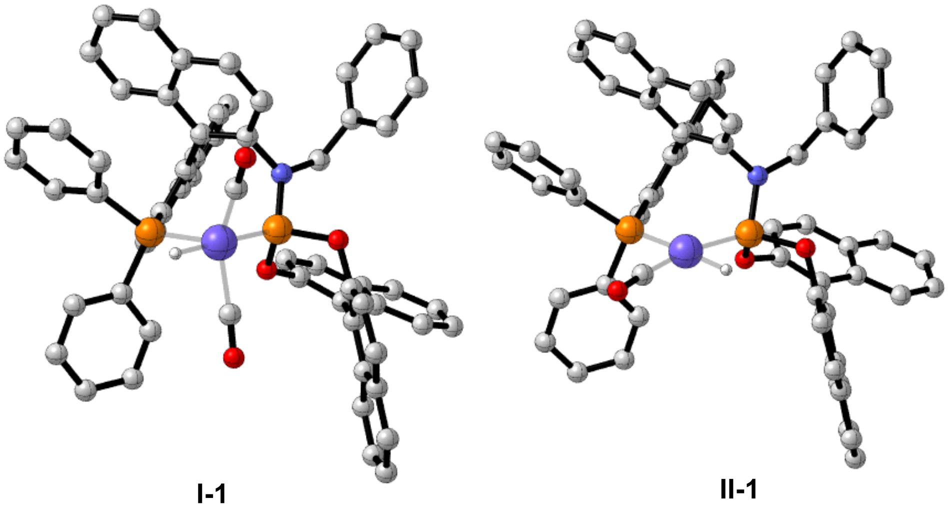

I-1
0.0

II-1
10.7

Supplementary Figure 2. Optimized geometry of (*S*,*R*)-(*N*-Bn)-Yanphos-Rh(CO)$_2$H (I-1) and (*S*,*R*)-(*N*-Bn)-Yanphos-Rh(CO)H (II-1) by M06/6-31G* method. Their relative free energies (in kcal/mol) in toluene solvent are given. Unimportant hydrogen atoms are not shown for clarity.

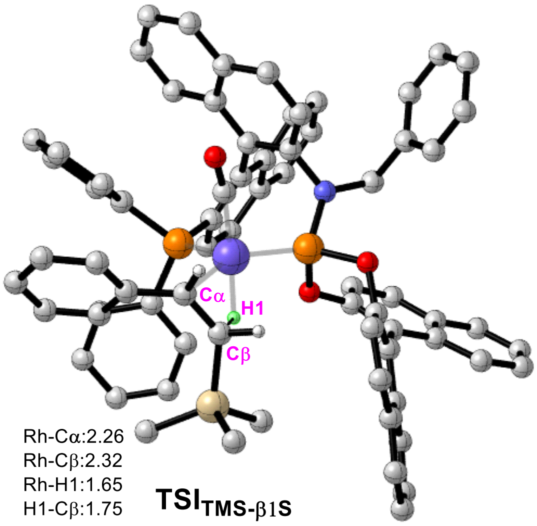

Rh-Cα:2.26
Rh-Cβ:2.32
Rh-H1:1.65
H1-Cβ:1.75

TSI$_{\mathbf{TMS\text{-}\beta 1S}}$
31.5

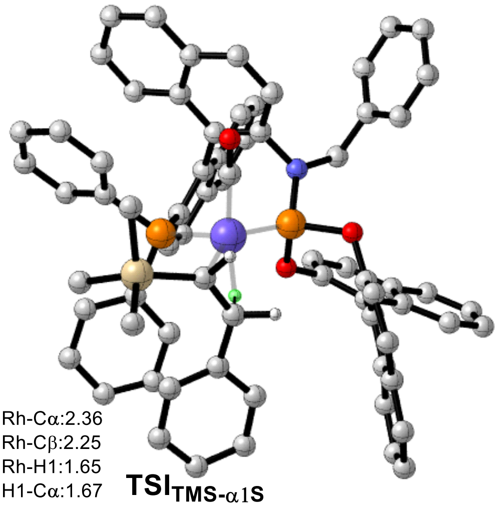

Rh-Cα:2.36
Rh-Cβ:2.25
Rh-H1:1.65
H1-Cα:1.67

TSI$_{\mathbf{TMS\text{-}\alpha 1S}}$
36.0

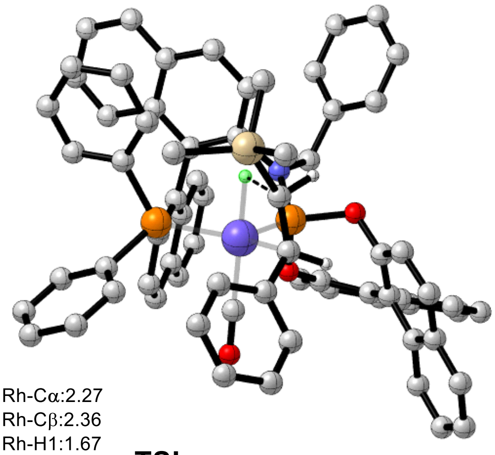

Rh-Cα:2.27
Rh-Cβ:2.36
Rh-H1:1.67
H1-Cβ:1.70

TSI$_{\mathbf{TMS\text{-}\beta 2R}}$
36.0

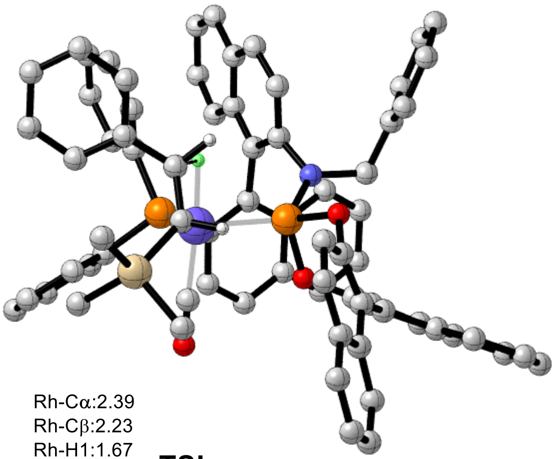

Rh-Cα:2.39
Rh-Cβ:2.23
Rh-H1:1.67
H1-Cα:1.69

TSI$_{\mathbf{TMS\text{-}\alpha 2R}}$
35.8

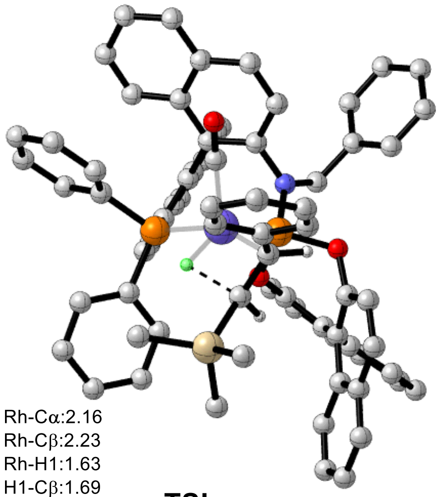

Rh-Cα:2.16
Rh-Cβ:2.23
Rh-H1:1.63
H1-Cβ:1.69

TSI$_{\mathbf{TMS\text{-}\beta 3R}}$
36.4

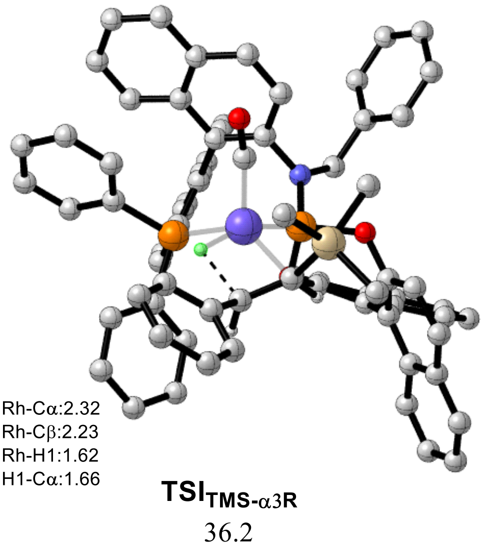

Rh-Cα:2.32
Rh-Cβ:2.23
Rh-H1:1.62
H1-Cα:1.66

TSI$_{\mathbf{TMS\text{-}\alpha 3R}}$
36.2

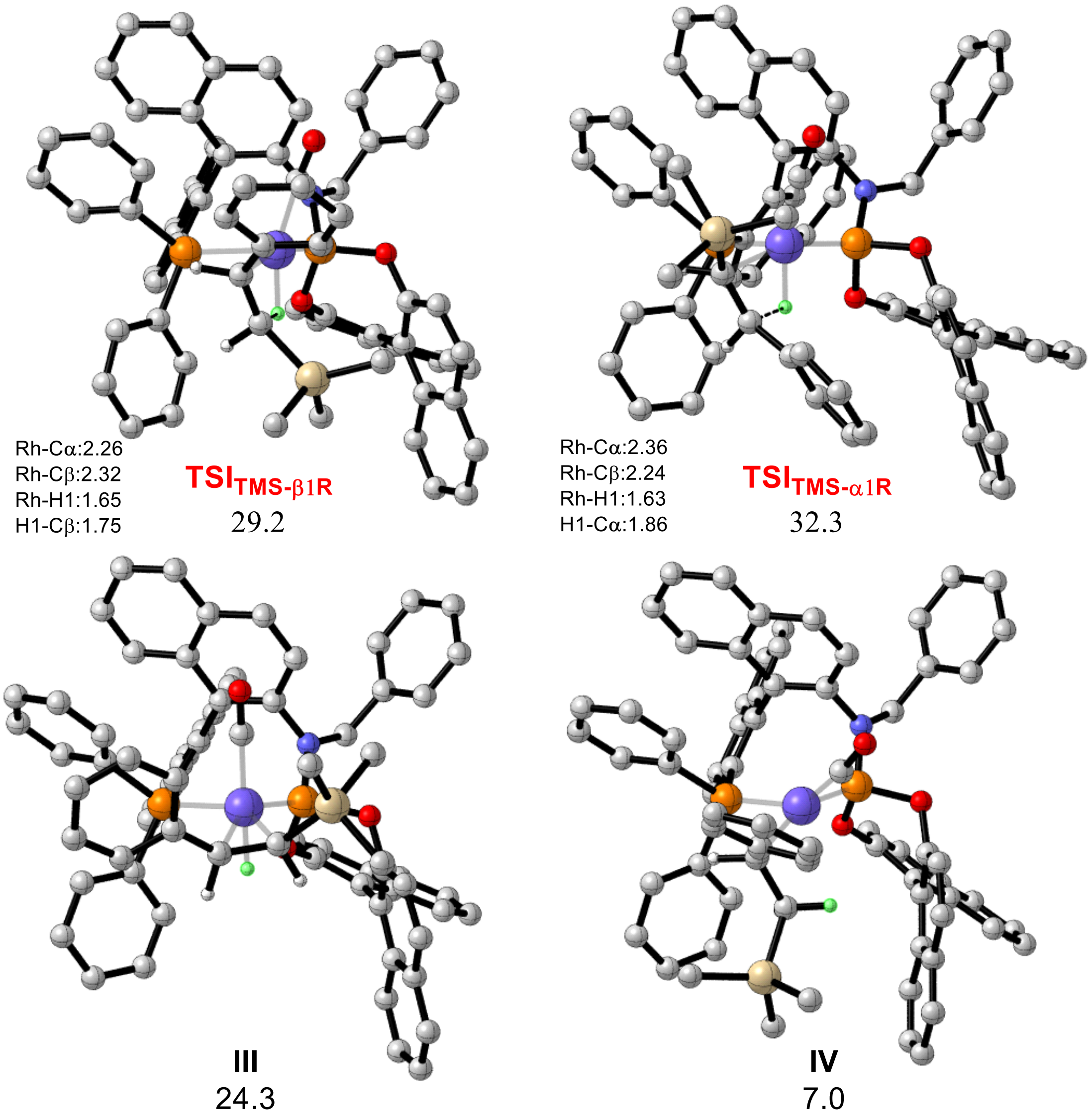

Supplementary Figure 3. Optimized transition states structures of all the alkene insertion step for hydroformylation of PhCH=CHTMS catalyzed by the catalyst I by M06/6-31G* method. The key bond lengths (in angstrom) and relative free energy (in kcal/mol) in toluene solvent are given. Unimportant hydrogen atoms are not shown for clarity.

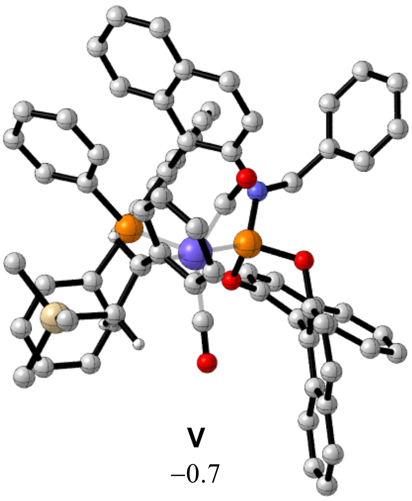

V
−0.7

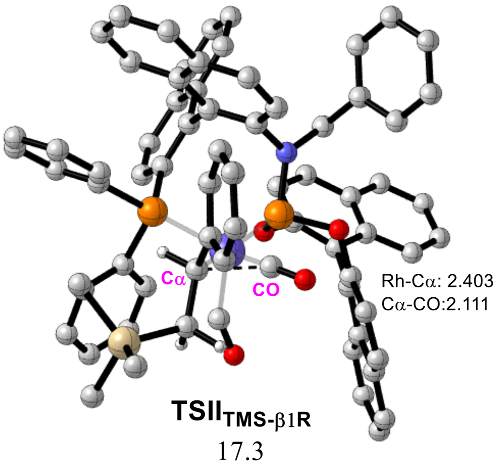

TSII$_{\mathbf{TMS\text{-}\beta 1R}}$
17.3

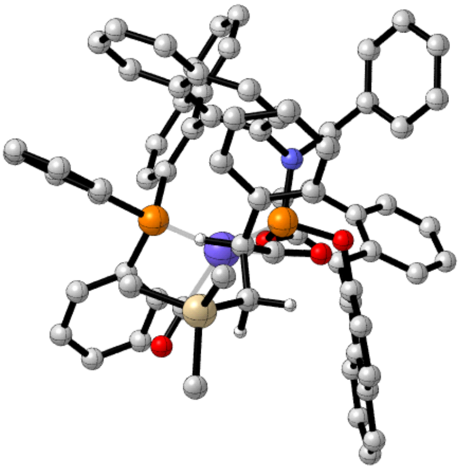

VI
−5.7

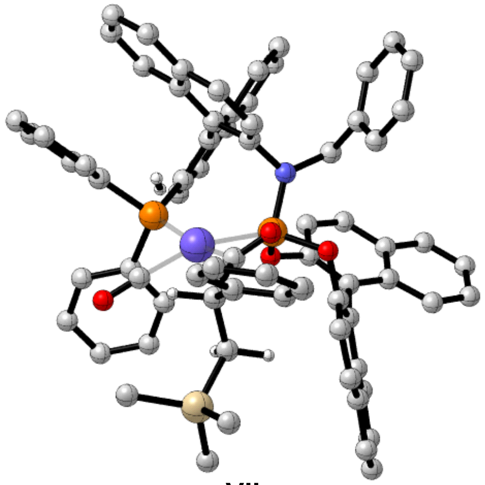

VII
3.0

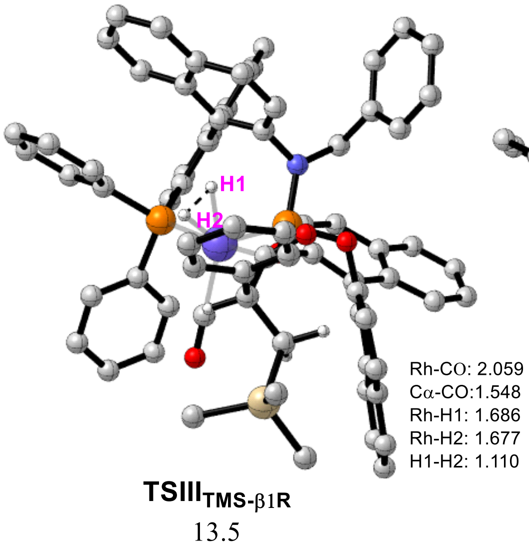

TSIII$_{\mathbf{TMS\text{-}\beta 1R}}$
13.5

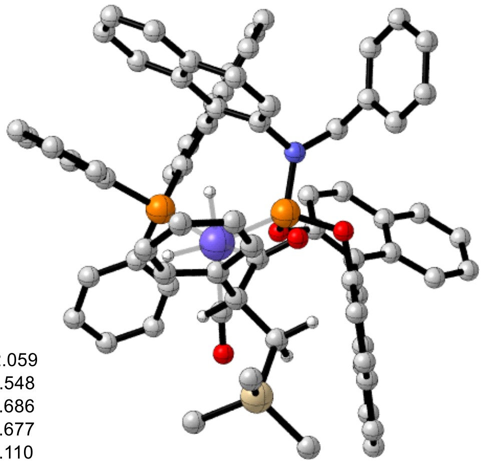

VIII
4.6

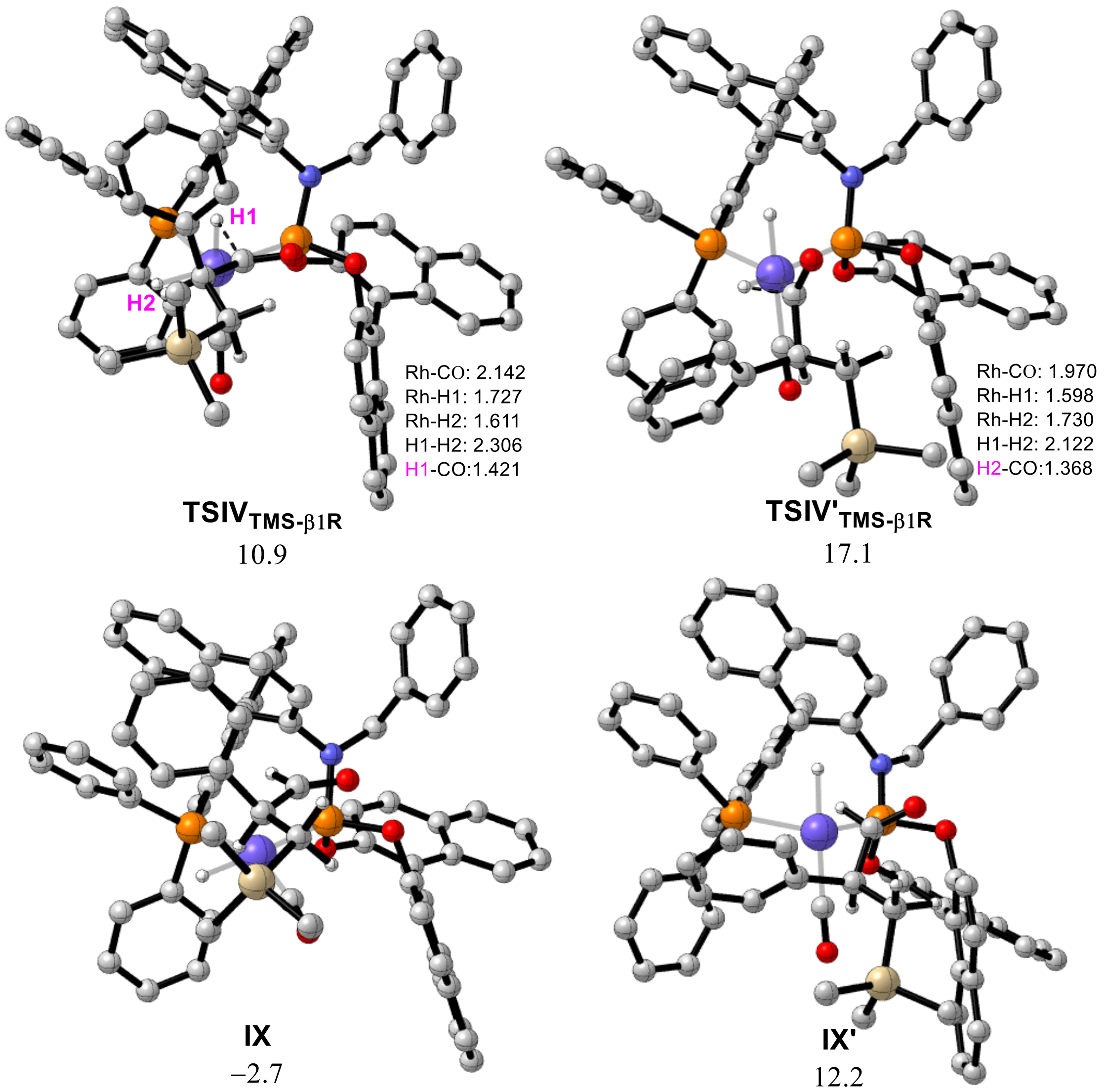

Supplementary Figure 4. Optimized structures of all the other transition states and intermediates for hydroformylation of PhCH=CHTMS catalyzed by the catalyst I by M06/6-31G* method. The key bond lengths (in angstrom) and relative free energy (in kcal/mol) in toluene solvent are given. Unimportant hydrogen atoms are not shown for clarity.

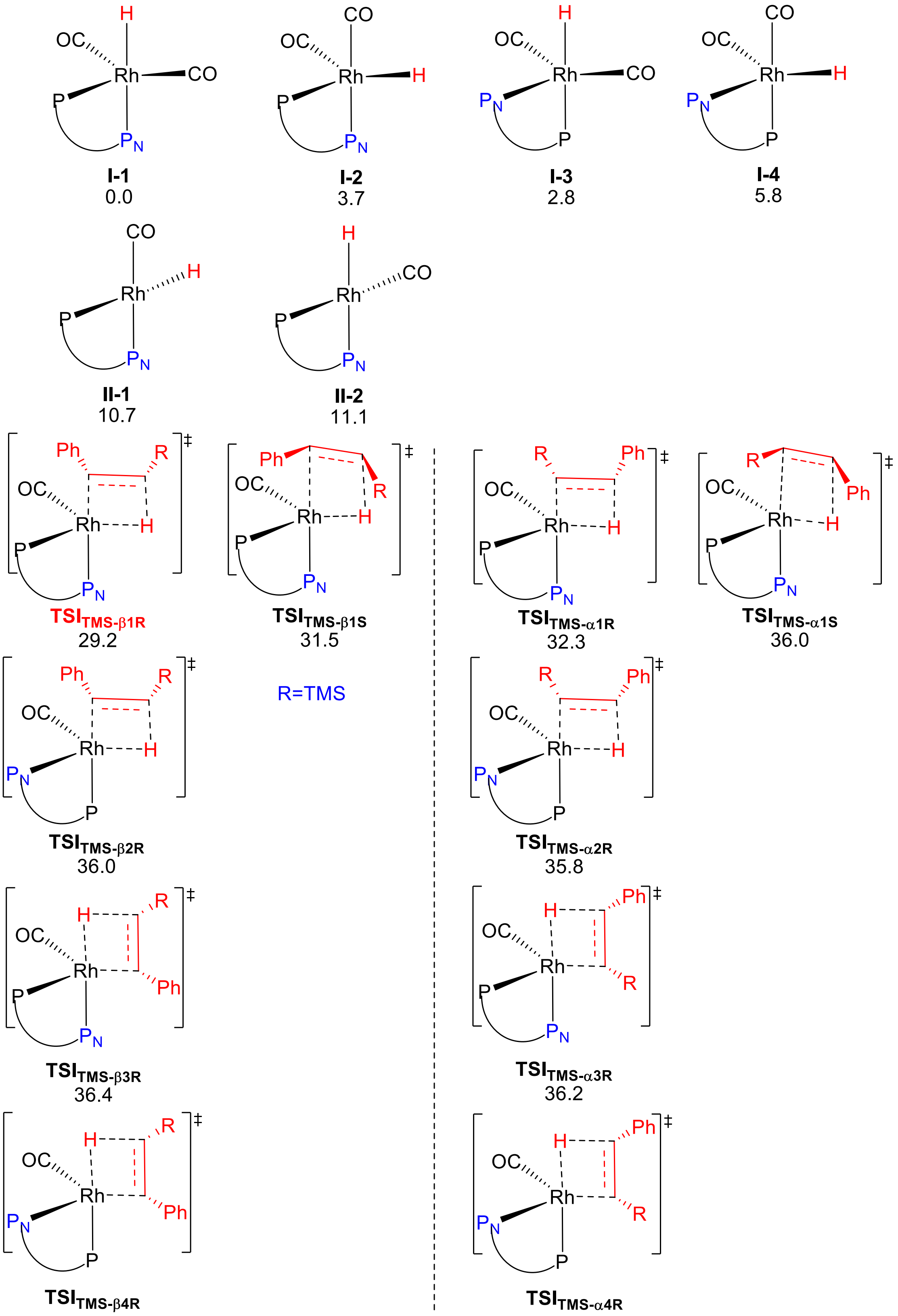

Supplementary Figure 5. Schematic structures for the catalyst I, II and the rate-determining step TSI for hydroformylation of PhCH=CHTMS. Their relative free energies (in kcal/mol) in toluene solvent are given.

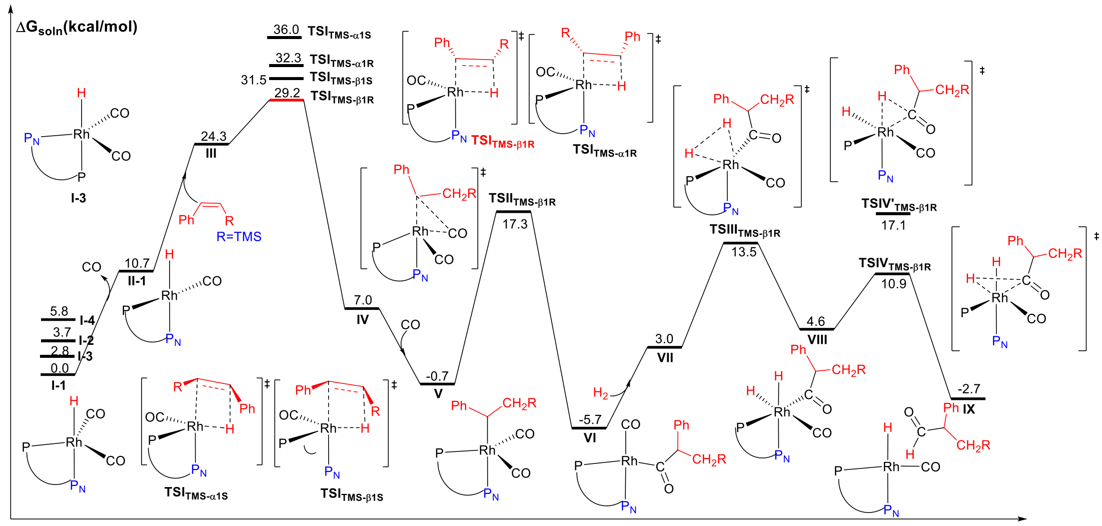

Supplementary Figure 6. Free energy profile of the favorable reaction pathway for hydroformylation of PhCH=CHTMS catalyzed by the catalyst I in toluene solvent with SMD model by M06/6-31G* method.

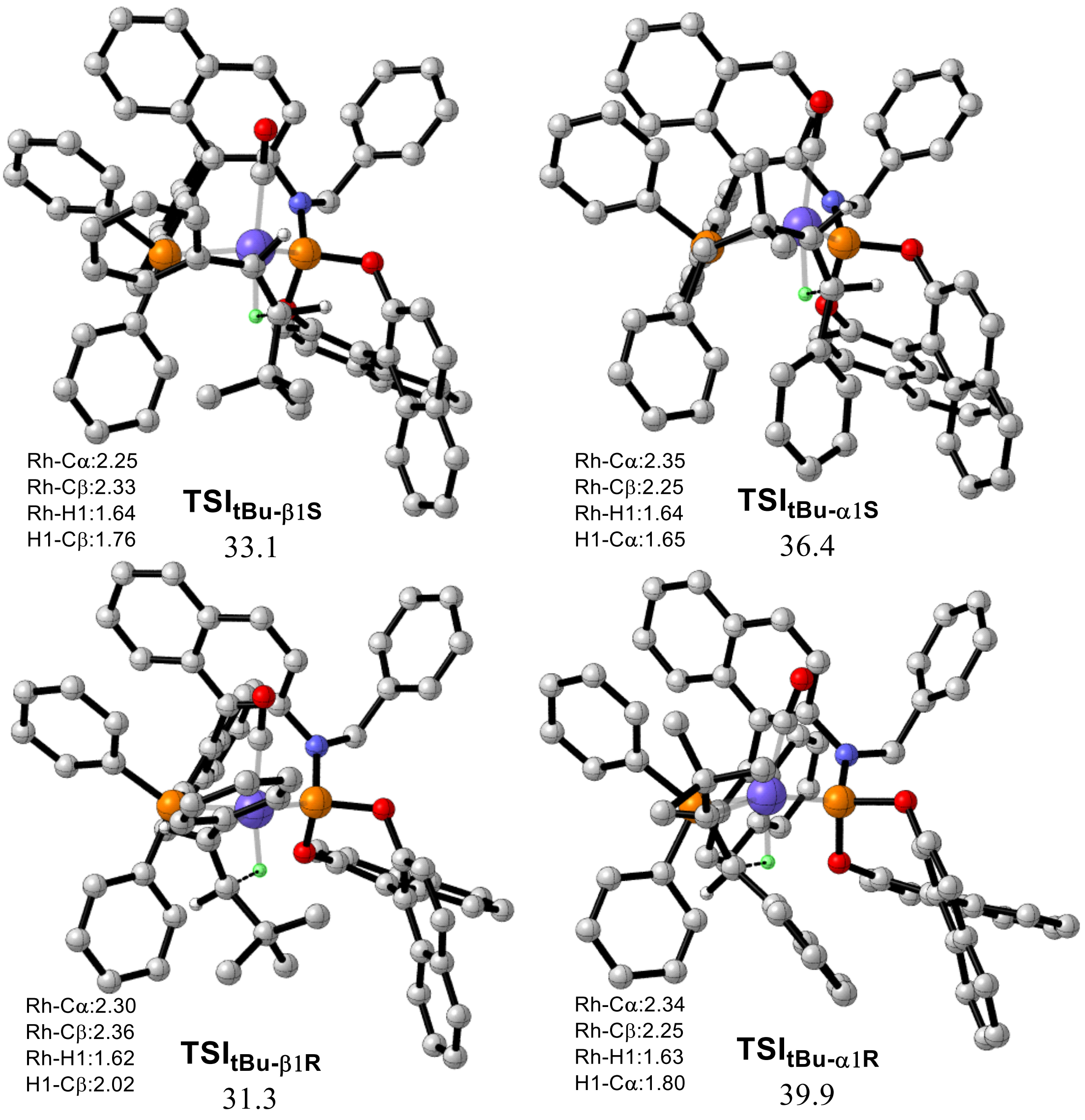

Supplementary Figure 7. Optimized transition states structures of all the alkene insertion step for hydroformylation of PhCH=CHtBu catalyzed by the catalyst I by M06/6-31G* method. The key bond lengths (in angstrom) and relative free energy (in kcal/mol) in toluene solvent are given. Unimportant hydrogen atoms are not shown for clarity.

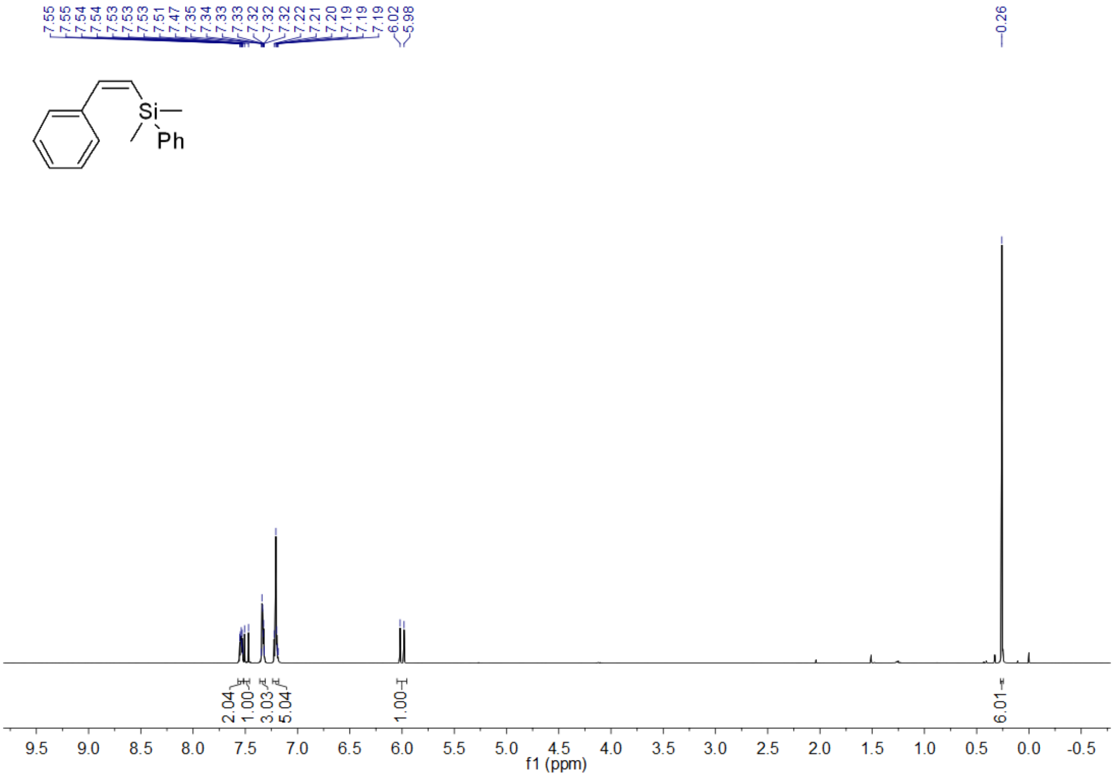

Supplementary Figure 8. ^{1}H **NMR (400 MHz,** $CDCl_3$**) spectra for compound 1b.**

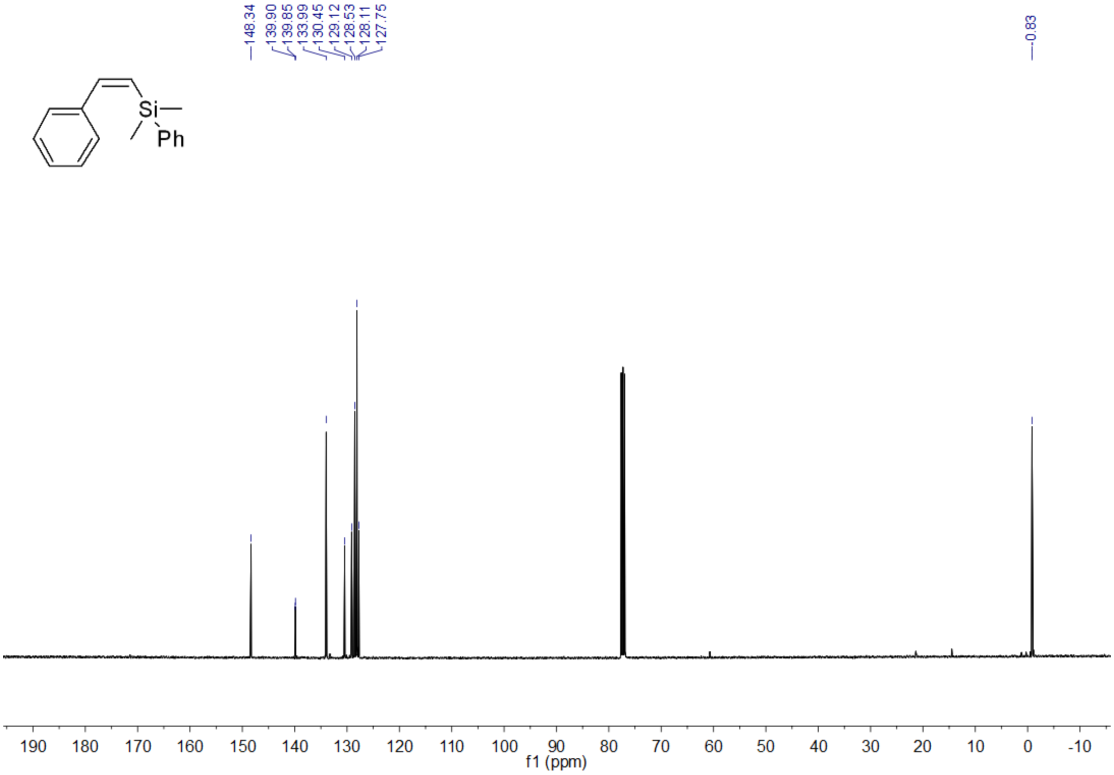

Supplementary Figure 9. ^{13}C **NMR (400 MHz,** $CDCl_3$**) spectra for compound 1b.**

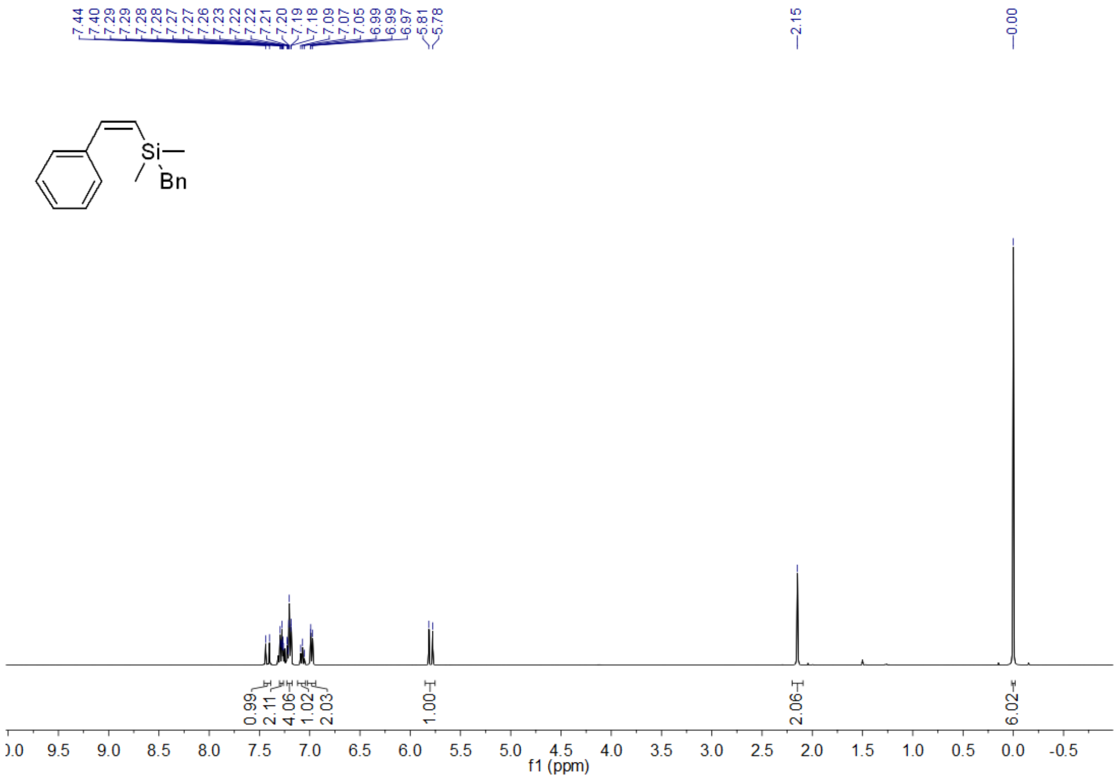

Supplementary Figure 10. ^{1}H NMR (400 MHz, $CDCl_3$) spectra for compound 1c

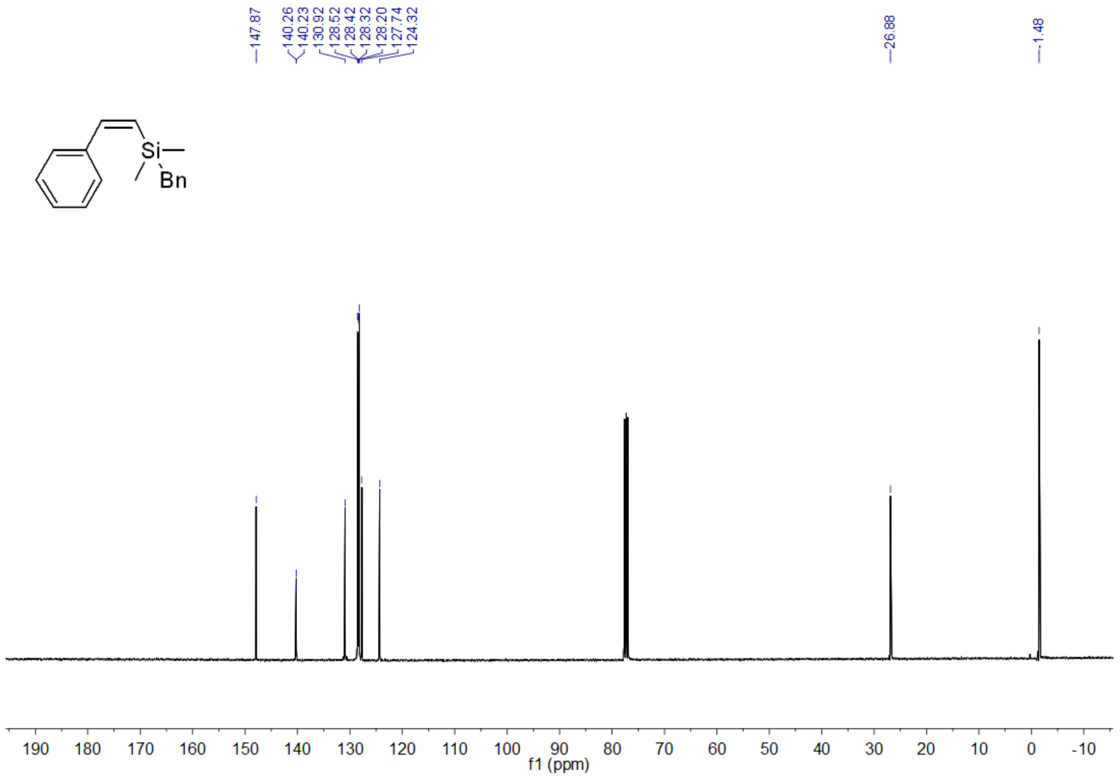

Supplementary Figure 11. ^{13}C NMR (400 MHz, $CDCl_3$) spectra for compound 1c.

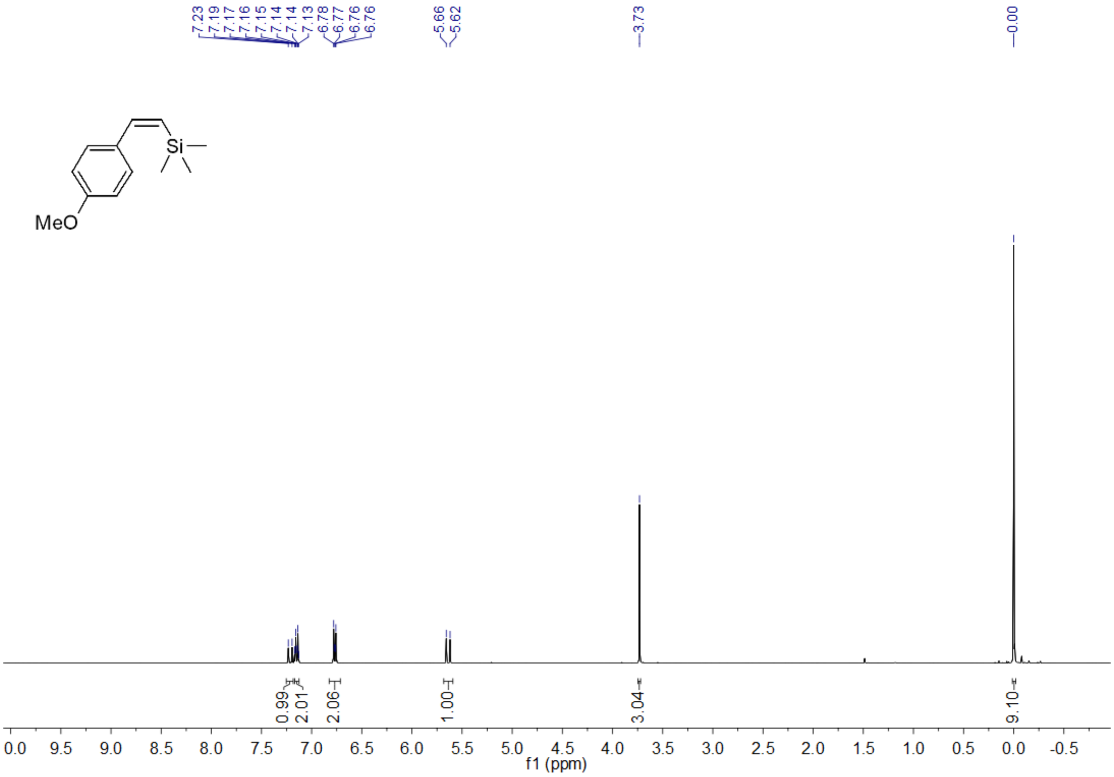

Supplementary Figure 12. ^{1}H NMR (400 MHz, $CDCl_3$) spectra for compound 1e

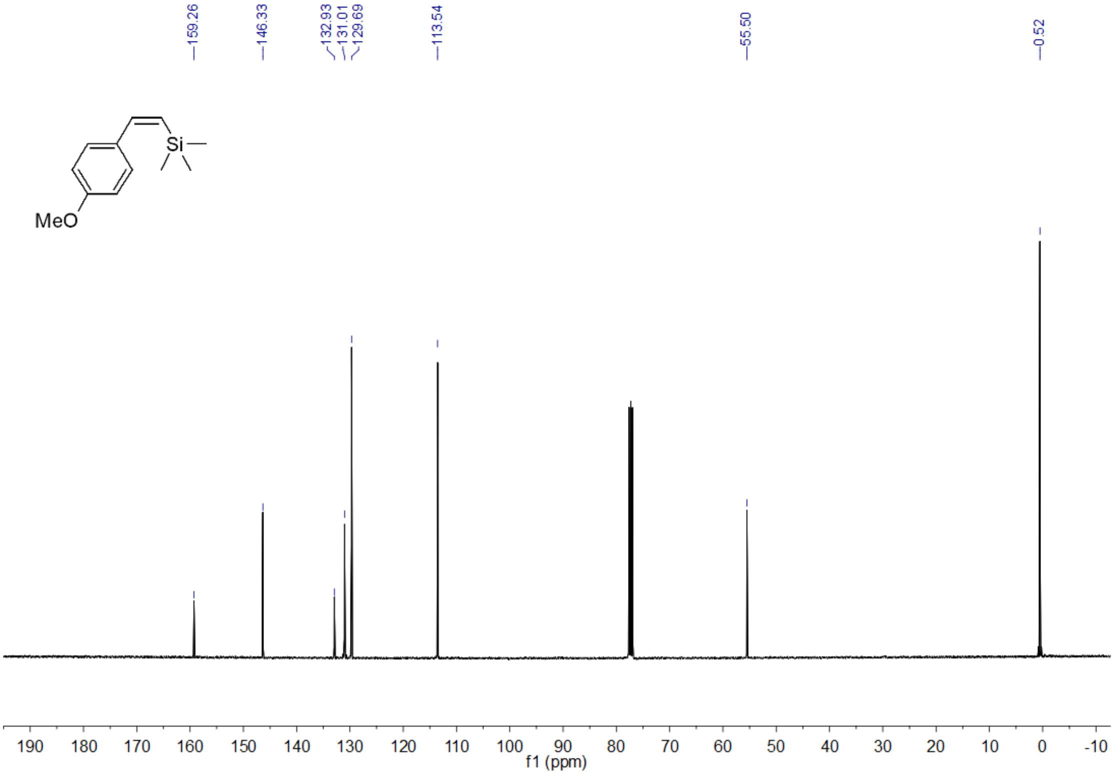

Supplementary Figure 13. ^{13}C NMR (400 MHz, $CDCl_3$) spectra for compound 1e.

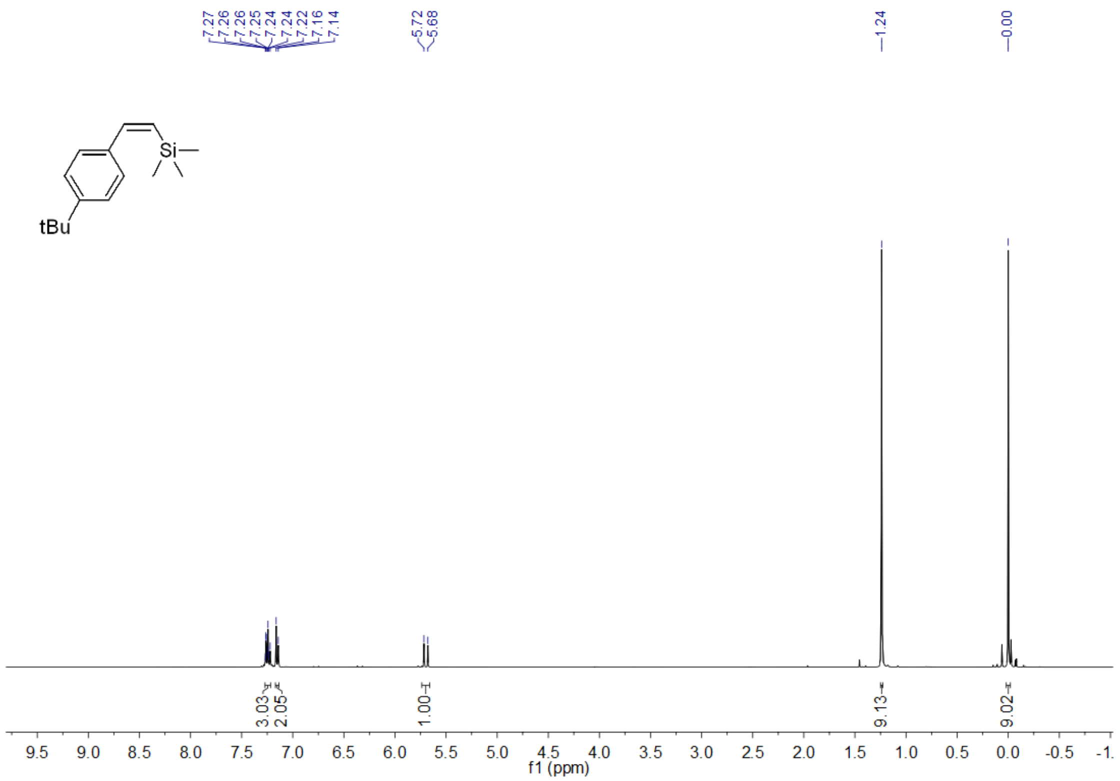

Supplementary Figure 14. ^{1}H NMR (400 MHz, $CDCl_3$) spectra for compound 1f

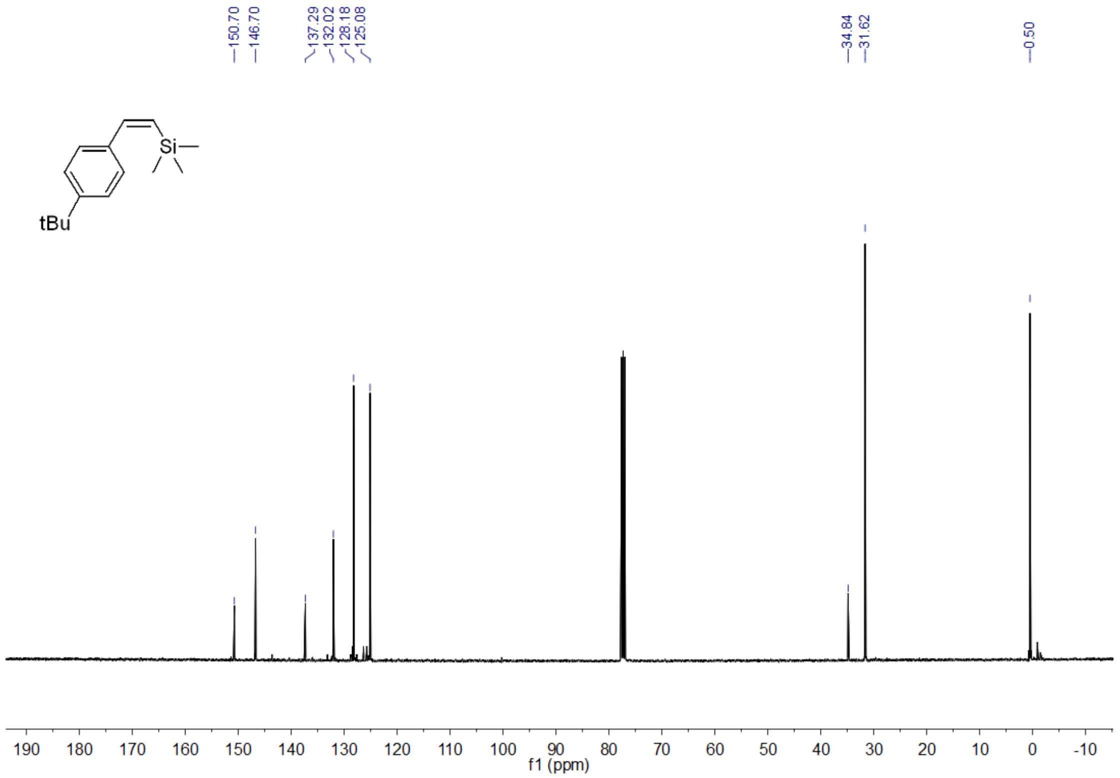

Supplementary Figure 15. ^{13}C NMR (400 MHz, $CDCl_3$) spectra for compound 1f

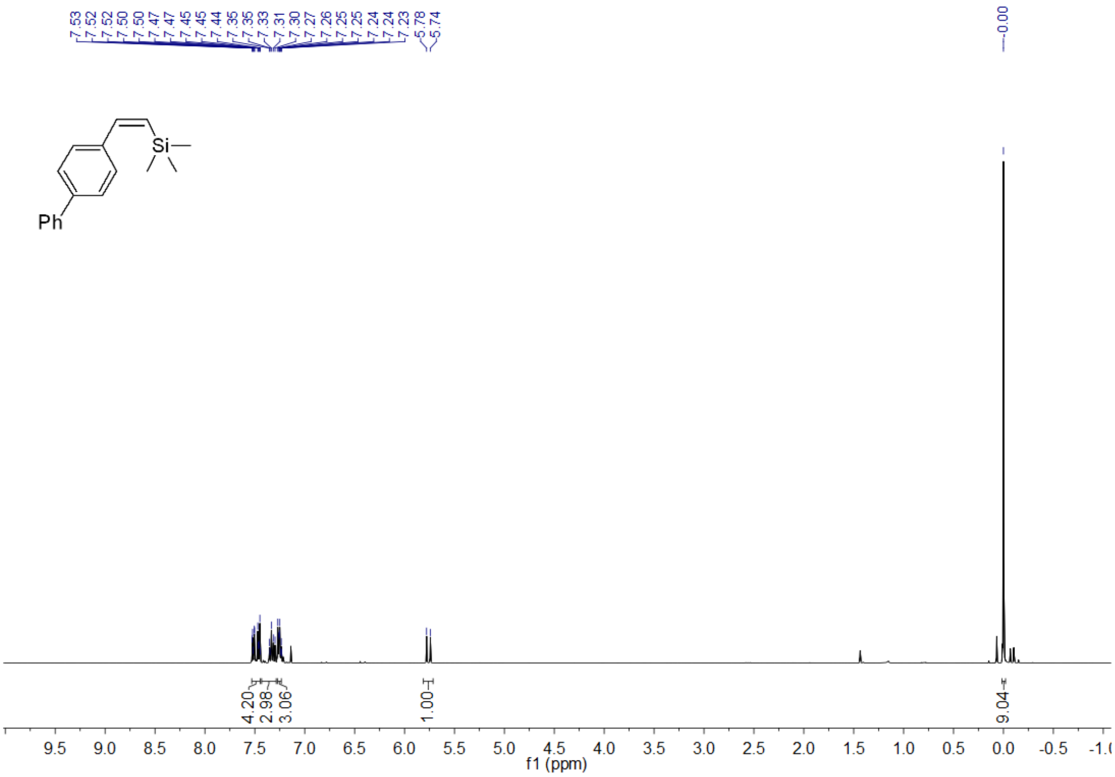

Supplementary Figure 16. ^{1}H NMR (400 MHz, $CDCl_3$) spectra for compound 1g

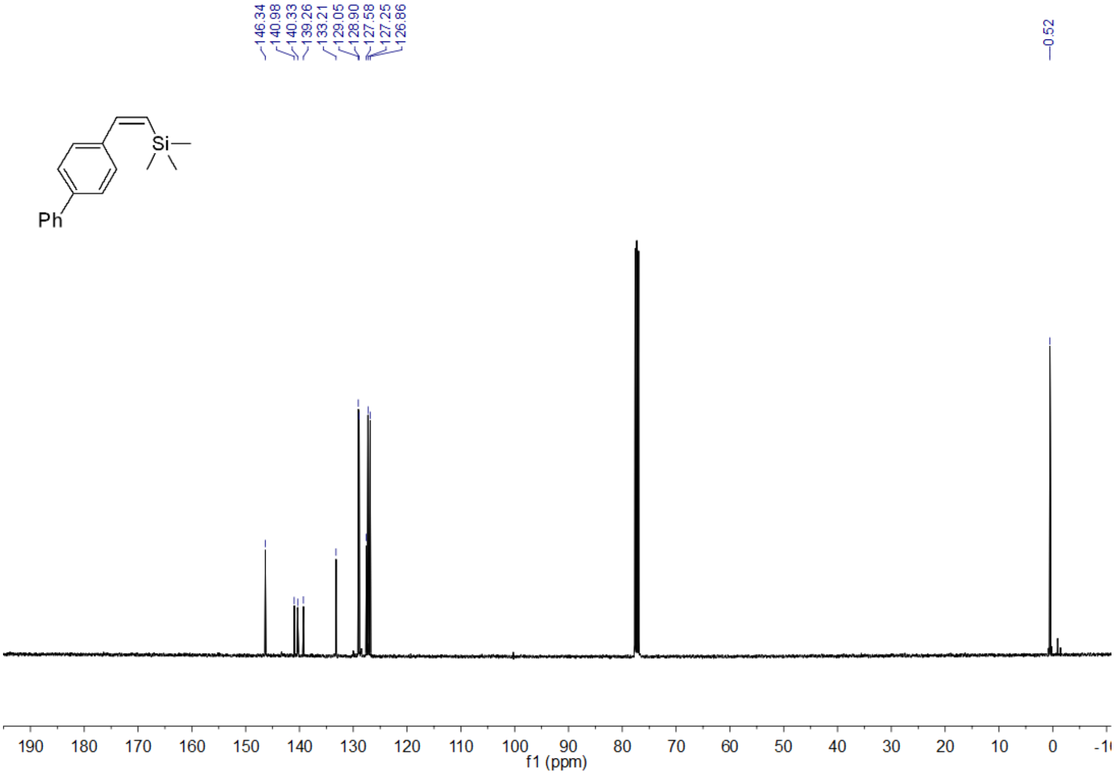

Supplementary Figure 17. ^{13}C NMR (400 MHz, $CDCl_3$) spectra for compound 1g

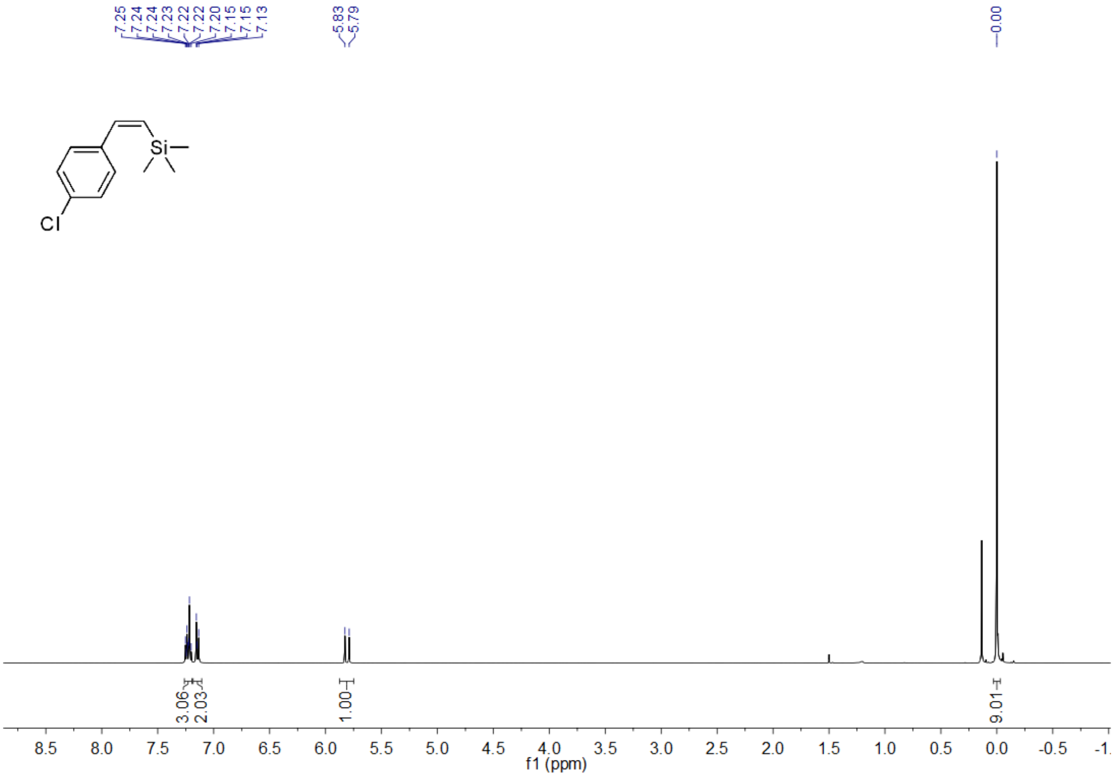

Supplementary Figure 18. ^{1}H NMR (400 MHz, $CDCl_3$) spectra for compound 1h

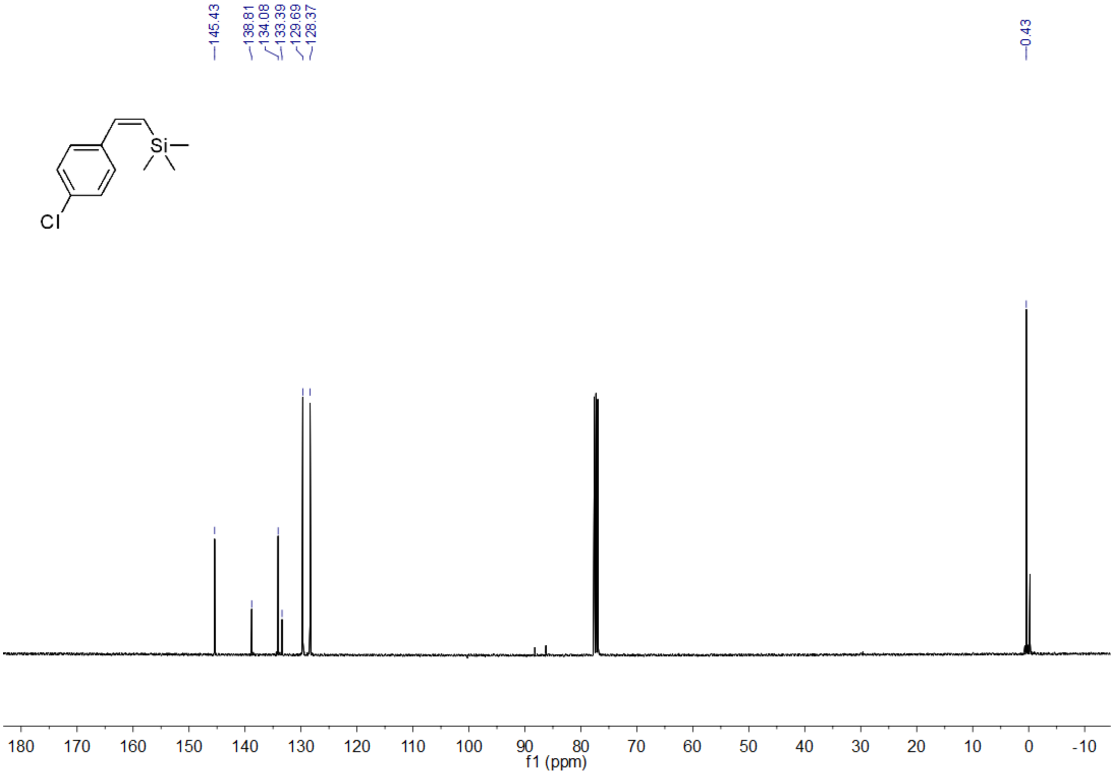

Supplementary Figure 19. ^{13}C NMR (400 MHz, $CDCl_3$) spectra for compound 1h

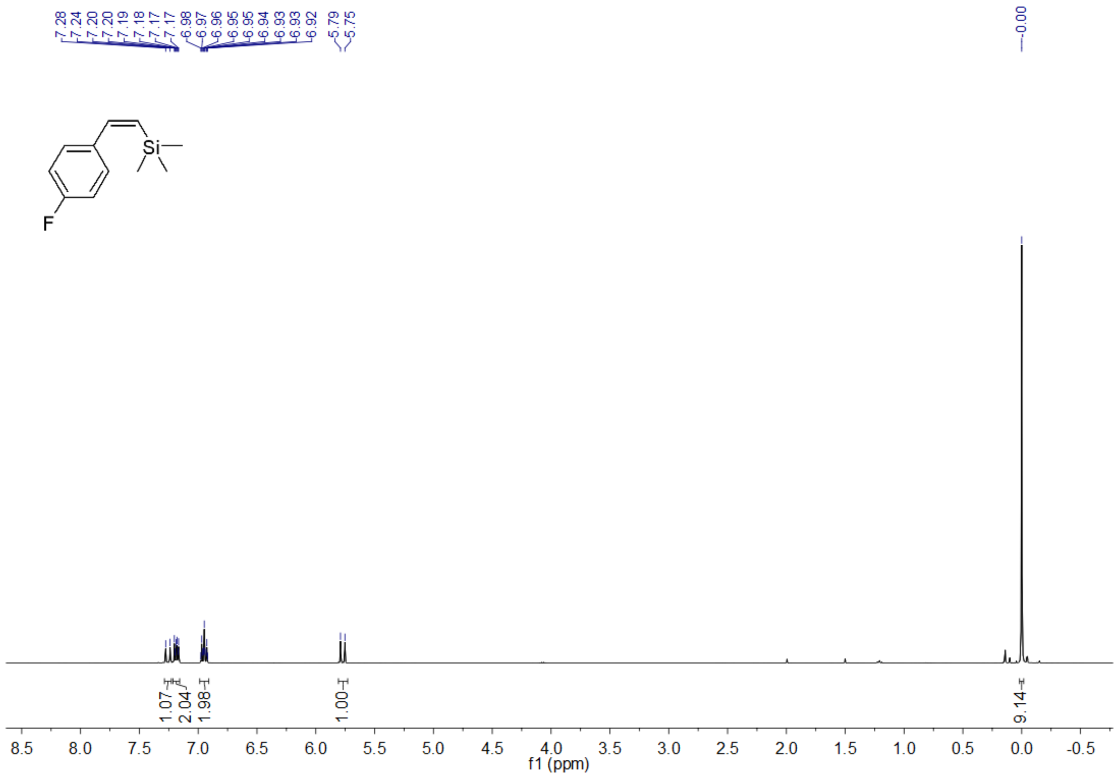

Supplementary Figure 20. **^{1}H NMR (400 MHz, $CDCl_3$) spectra for compound 1i**

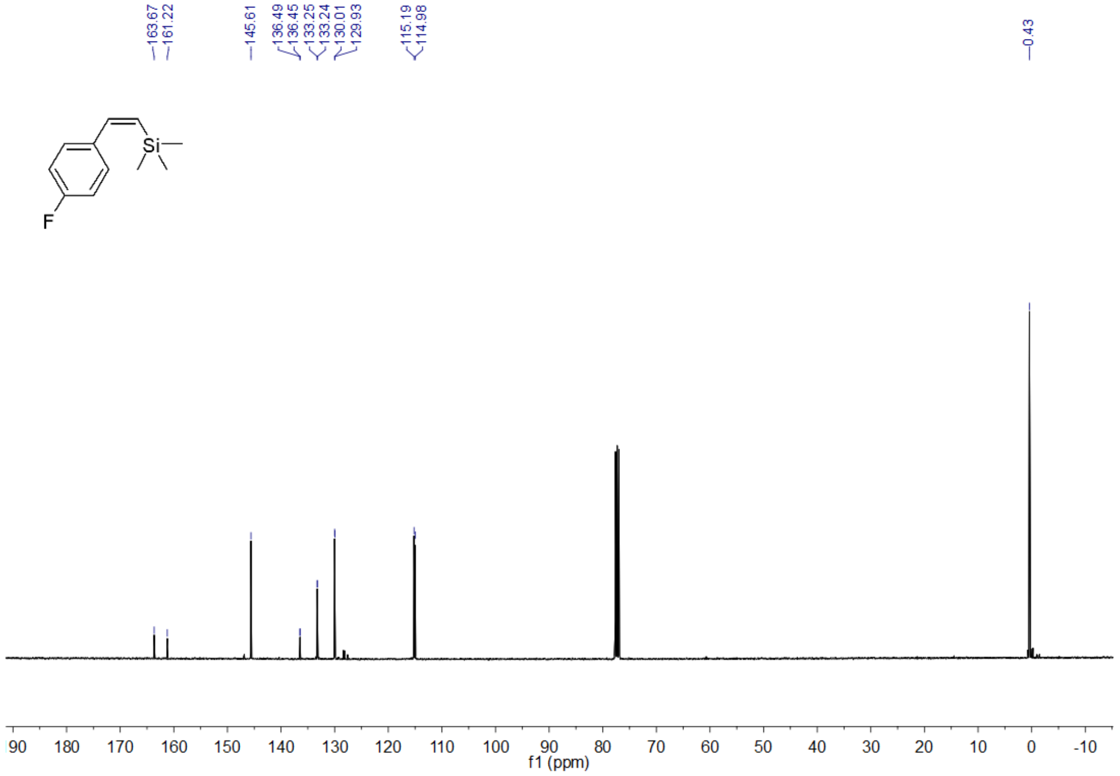

Supplementary Figure 21. **^{13}C NMR (400 MHz, $CDCl_3$) spectra for compound 1i**

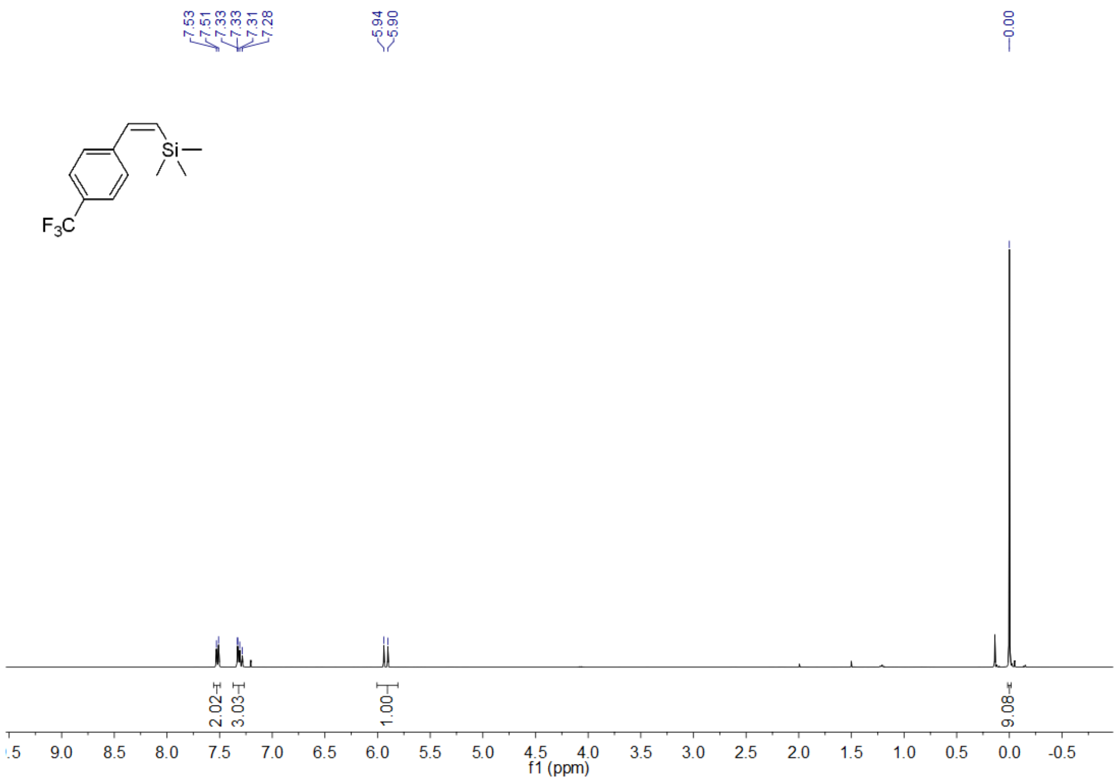

Supplementary Figure 22. ^{1}H NMR (400 MHz, $CDCl_3$) spectra for compound 1j

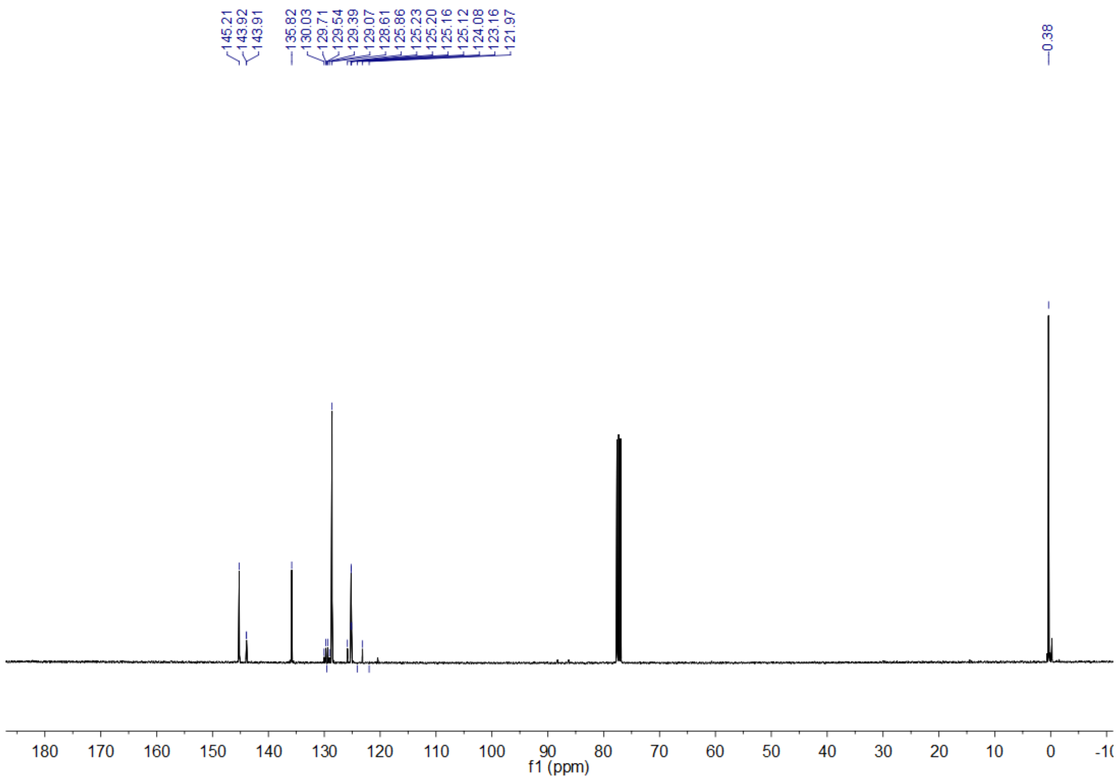

Supplementary Figure 23. ^{13}C NMR (400 MHz, $CDCl_3$) spectra for compound 1j

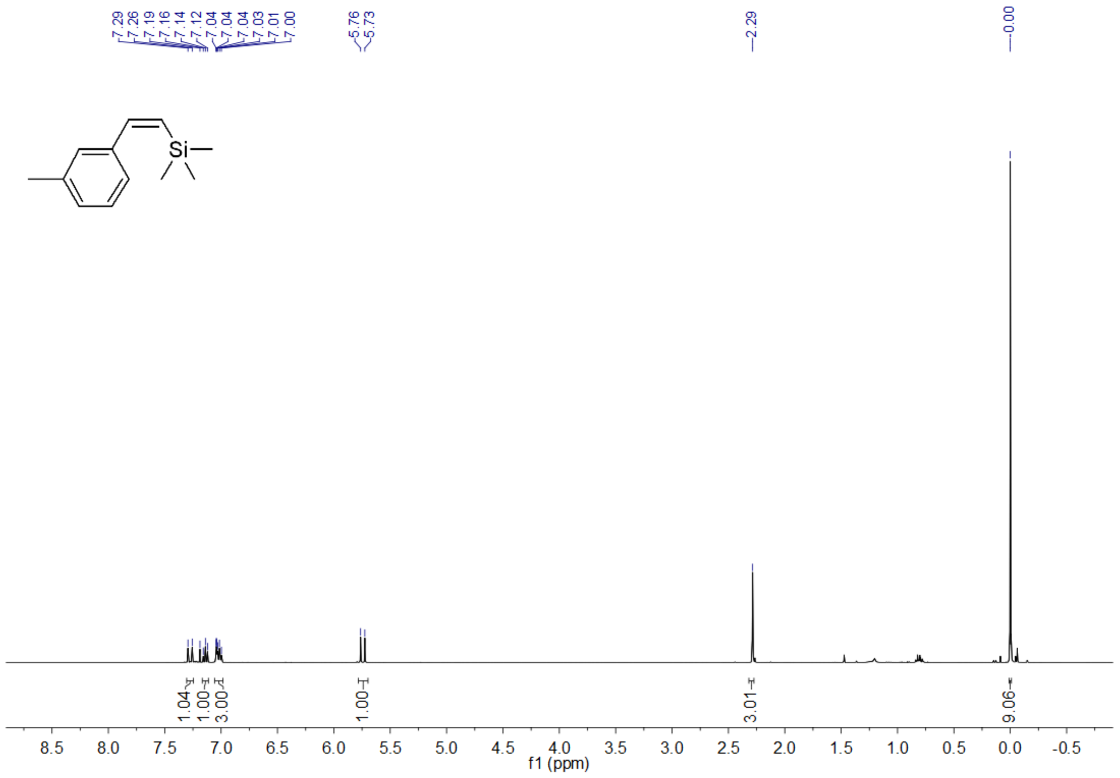

Supplementary Figure 24. **^{1}H NMR (400 MHz, $CDCl_3$) spectra for compound 1k**

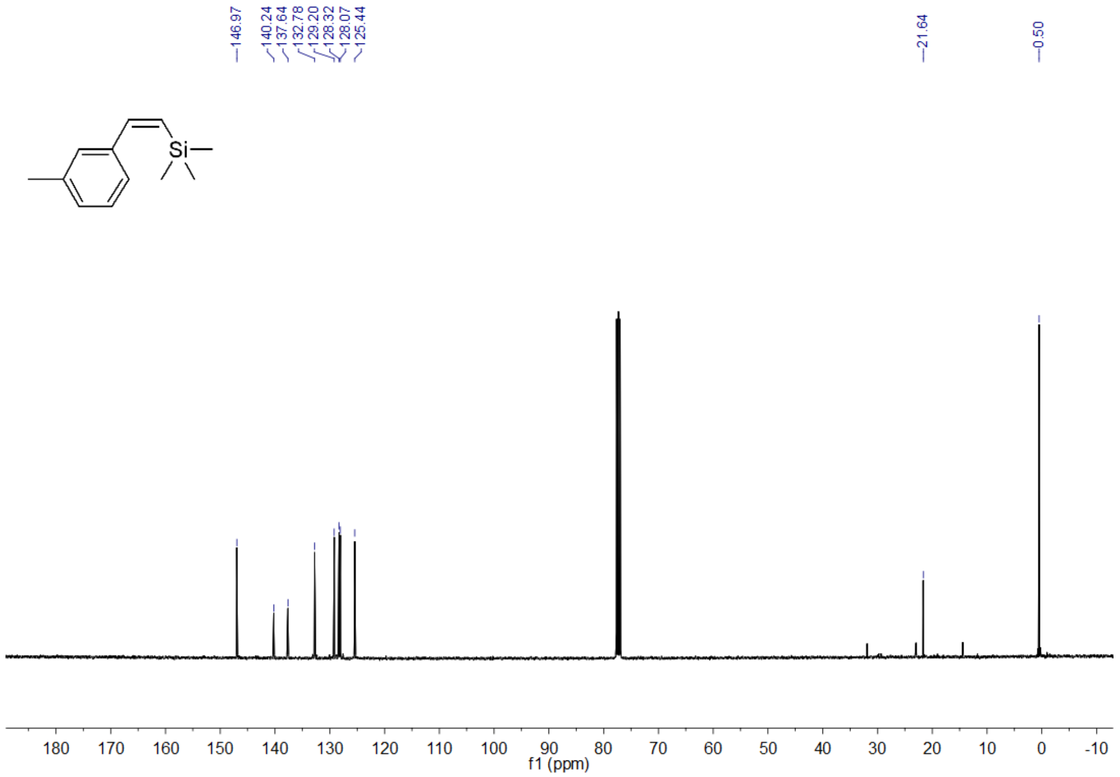

Supplementary Figure 25. **^{13}C NMR (400 MHz, $CDCl_3$) spectra for compound 1k**

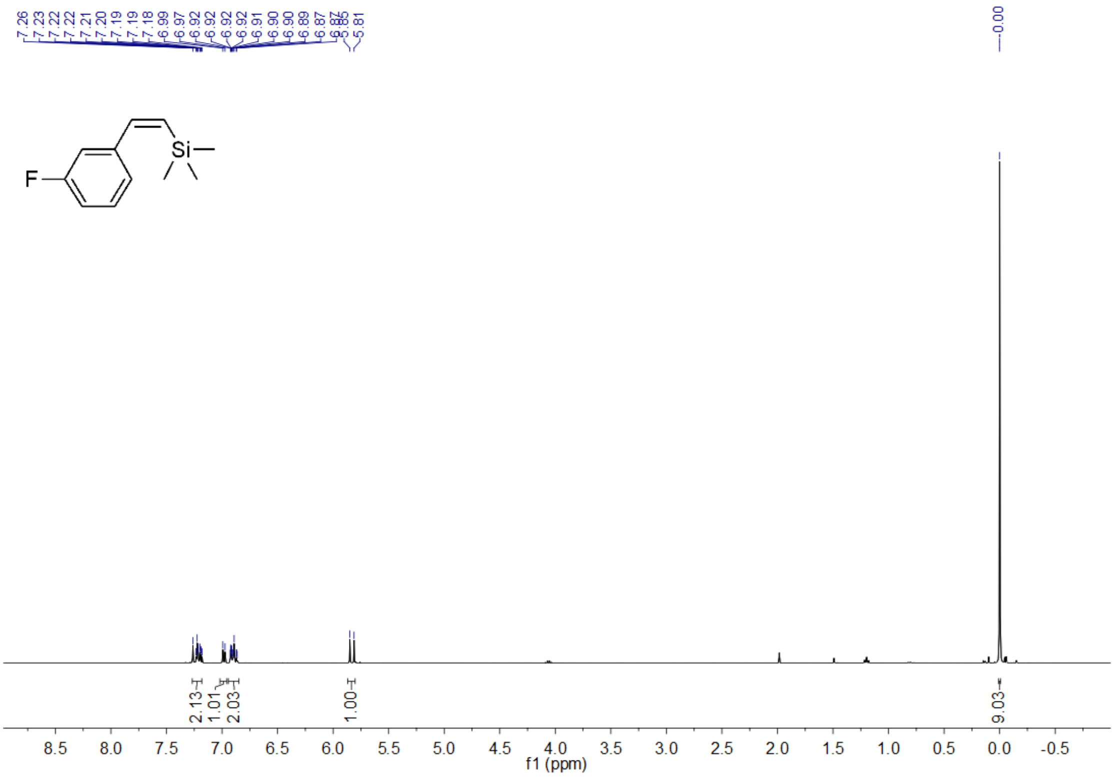

Supplementary Figure 26. ^{1}**H NMR (400 MHz,** $CDCl_3$**) spectra for compound 1l**

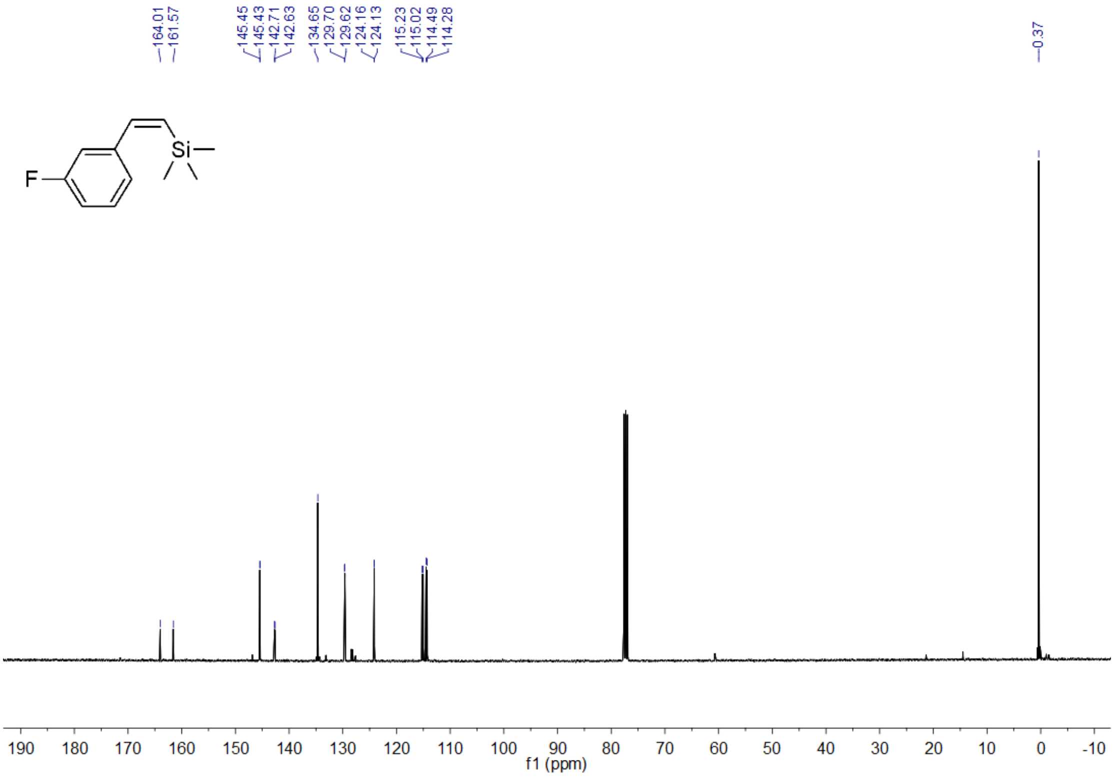

Supplementary Figure 27. ^{13}**C NMR (400 MHz,** $CDCl_3$**) spectra for compound 1l**

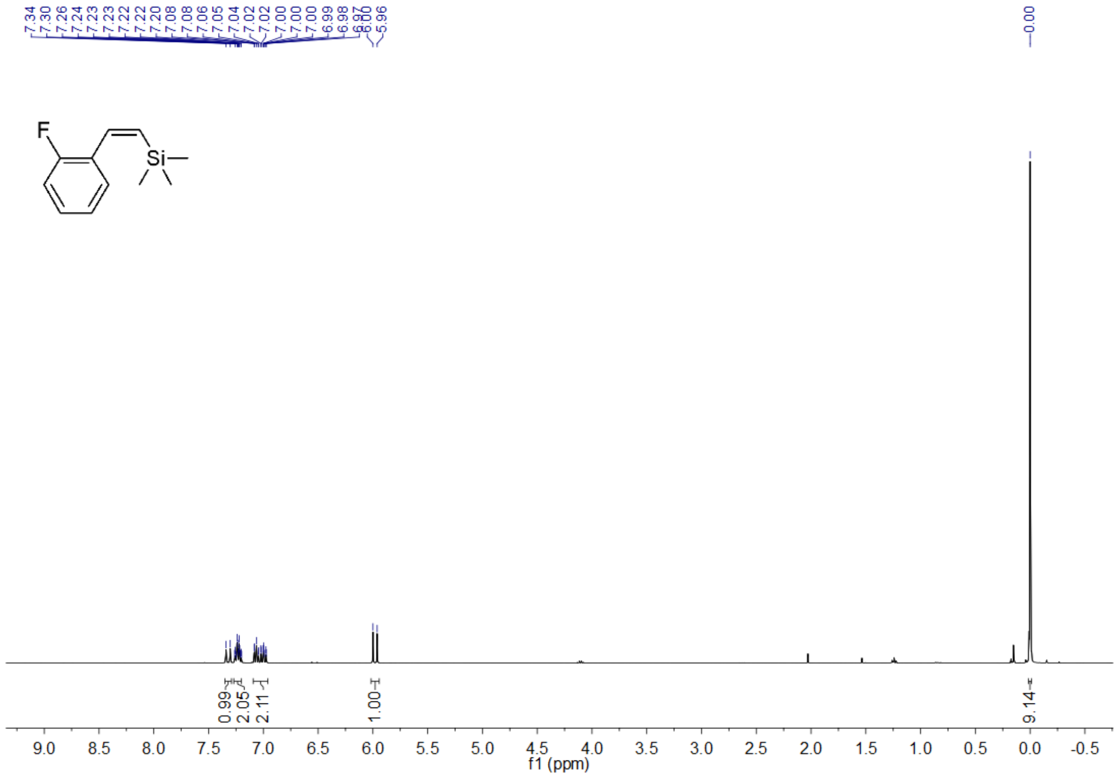

Supplementary Figure 28. ^{1}H NMR (400 MHz, $CDCl_3$) spectra for compound 1m

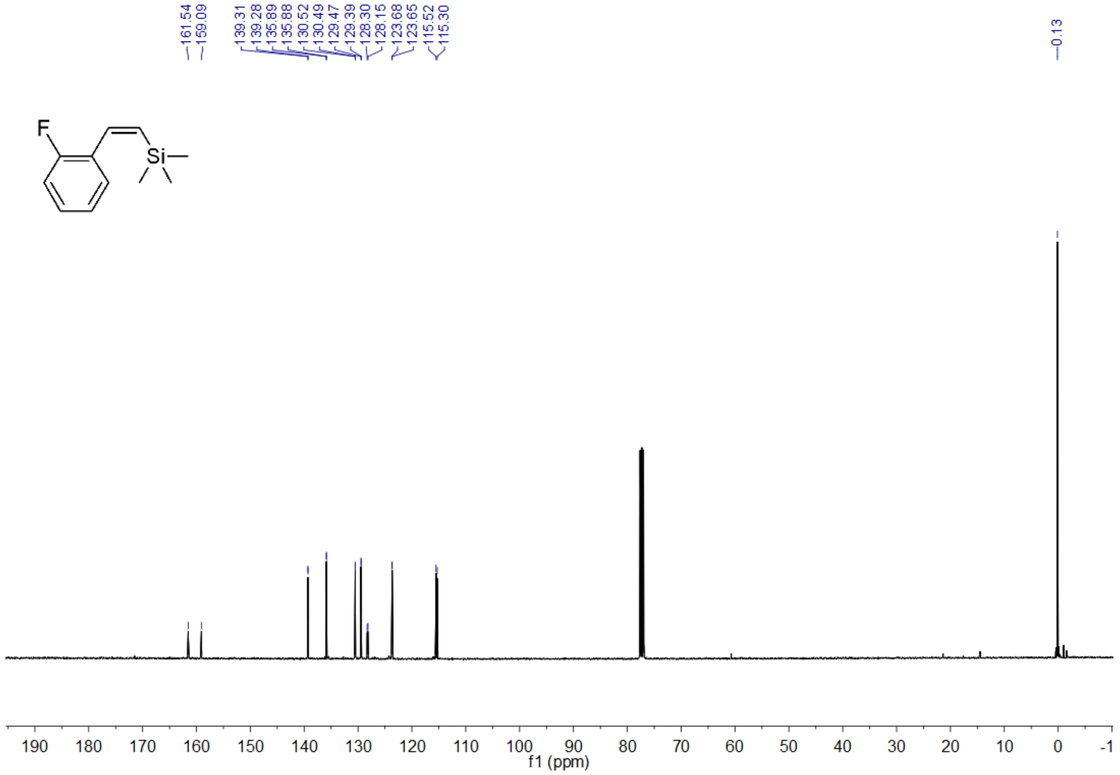

Supplementary Figure 29. ^{13}C NMR (400 MHz, $CDCl_3$) spectra for compound 1m

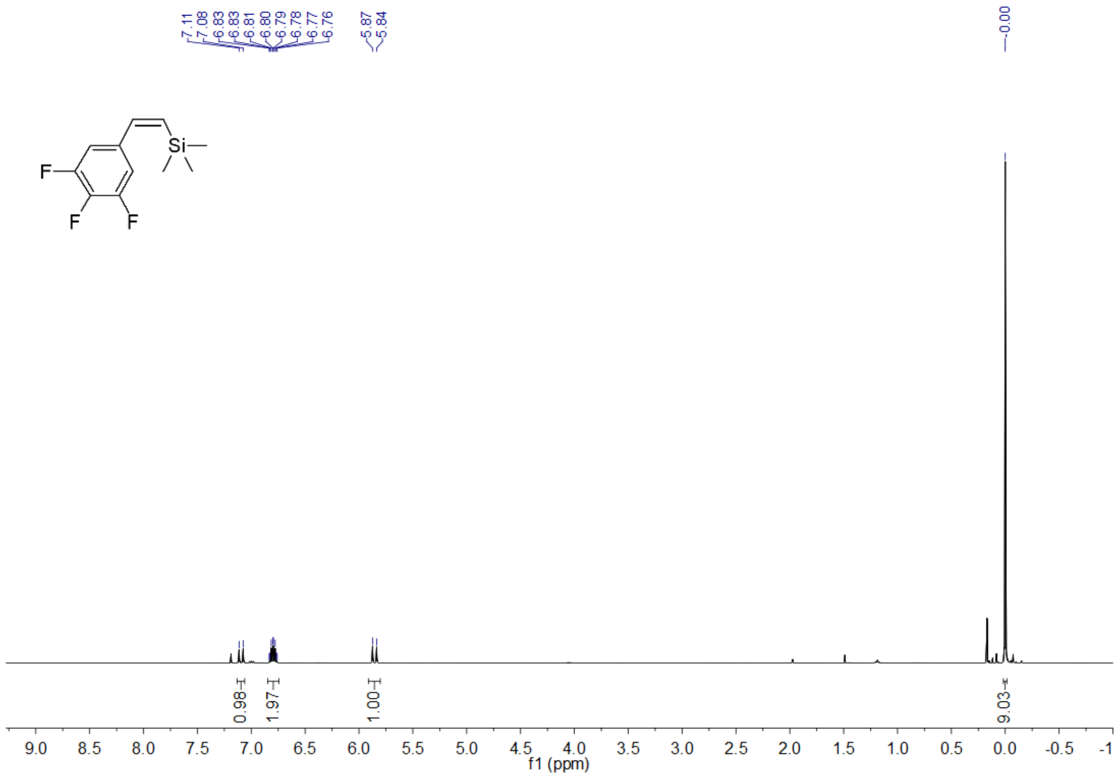

Supplementary Figure 30. ^{1}**H NMR (400 MHz,** $CDCl_3$**) spectra for compound 1n**

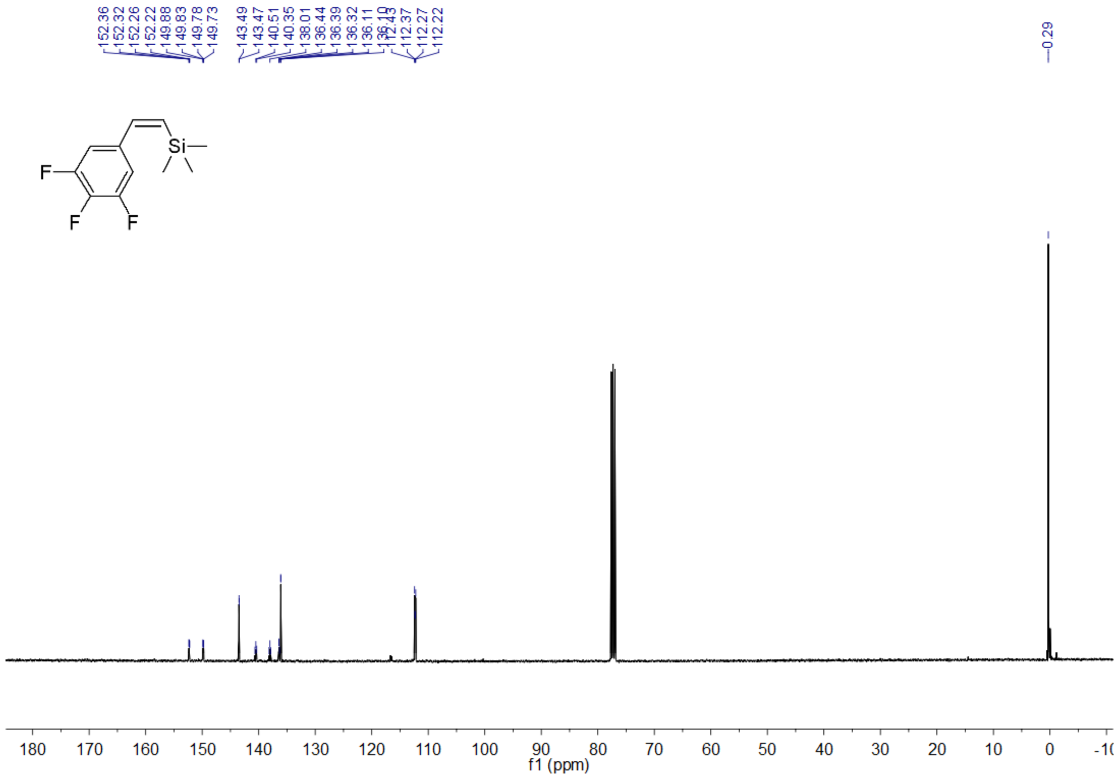

Supplementary Figure 31. ^{13}**C NMR (400 MHz,** $CDCl_3$**) spectra for compound 1n**

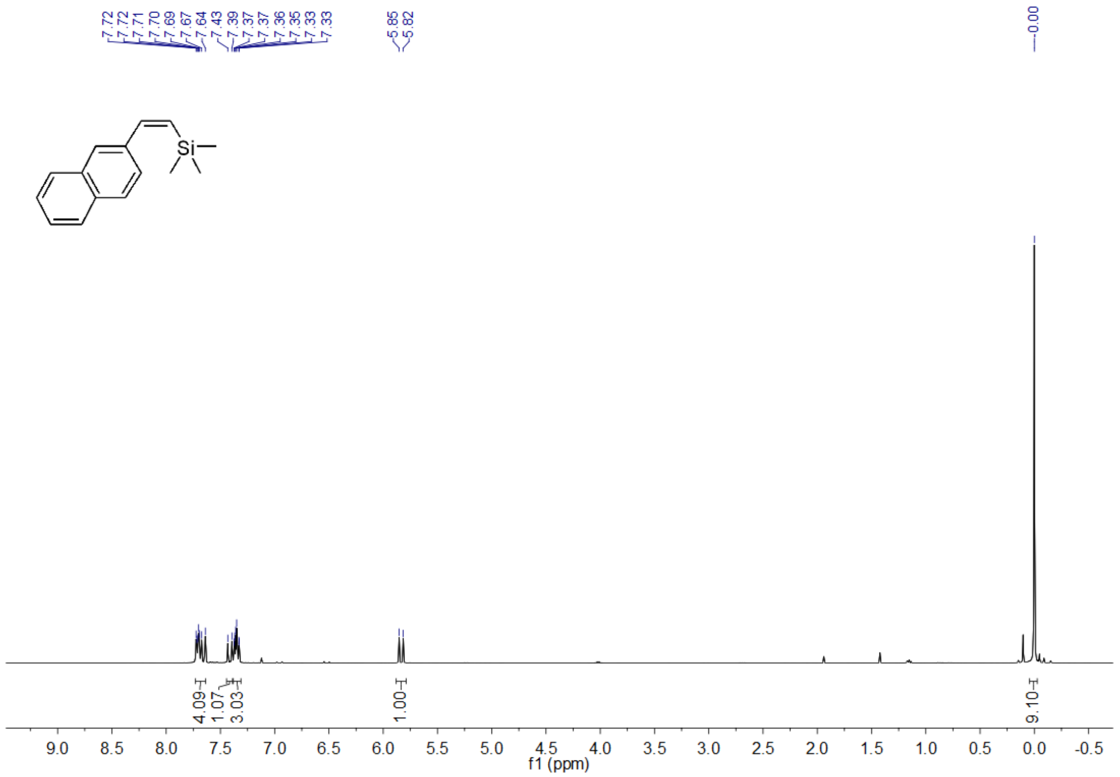

Supplementary Figure 32. ^{1}H NMR (400 MHz, $CDCl_3$) spectra for compound 1o

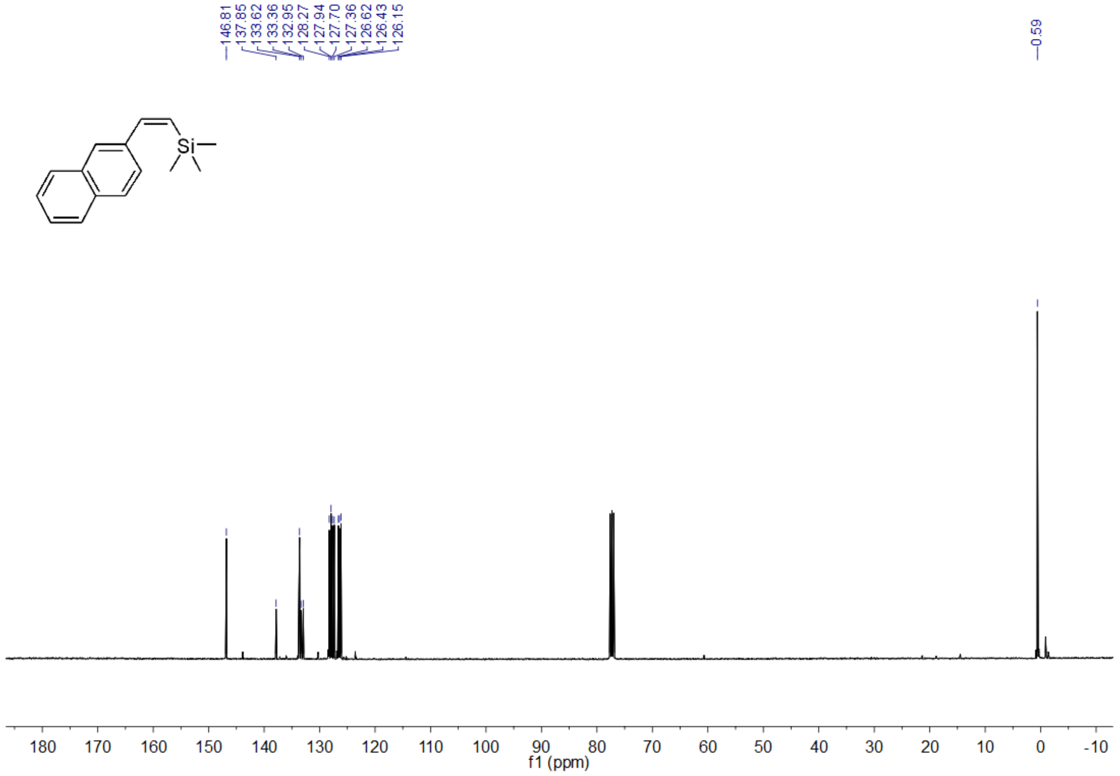

Supplementary Figure 33. ^{13}C NMR (400 MHz, $CDCl_3$) spectra for compound 1o

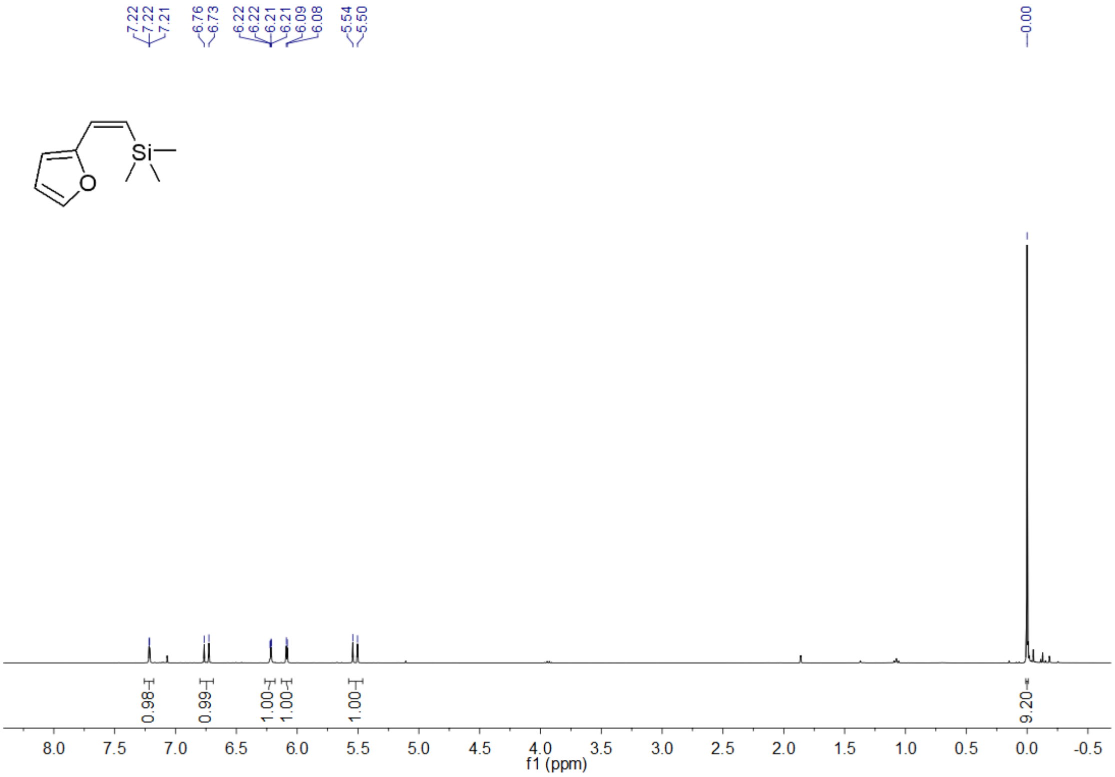

Supplementary Figure 34. ^{1}H **NMR (400 MHz,** $CDCl_3$**) spectra for compound 1p**

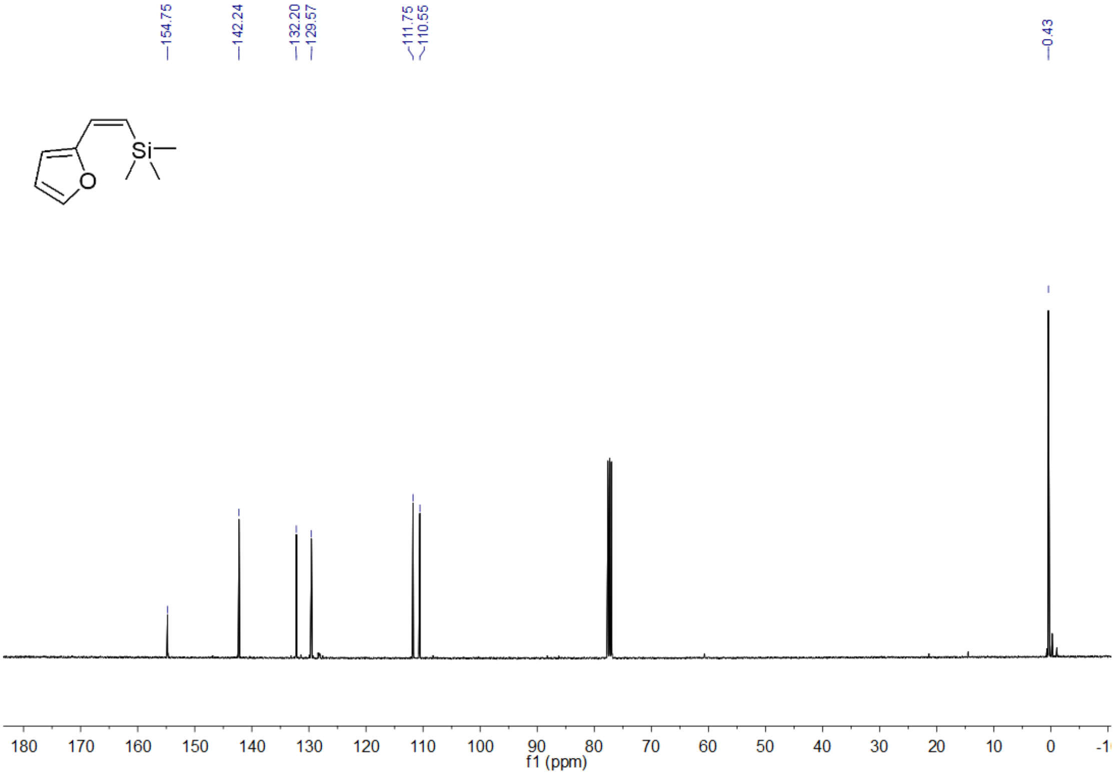

Supplementary Figure 35. ^{13}C **NMR (400 MHz,** $CDCl_3$**) spectra for compound 1p**

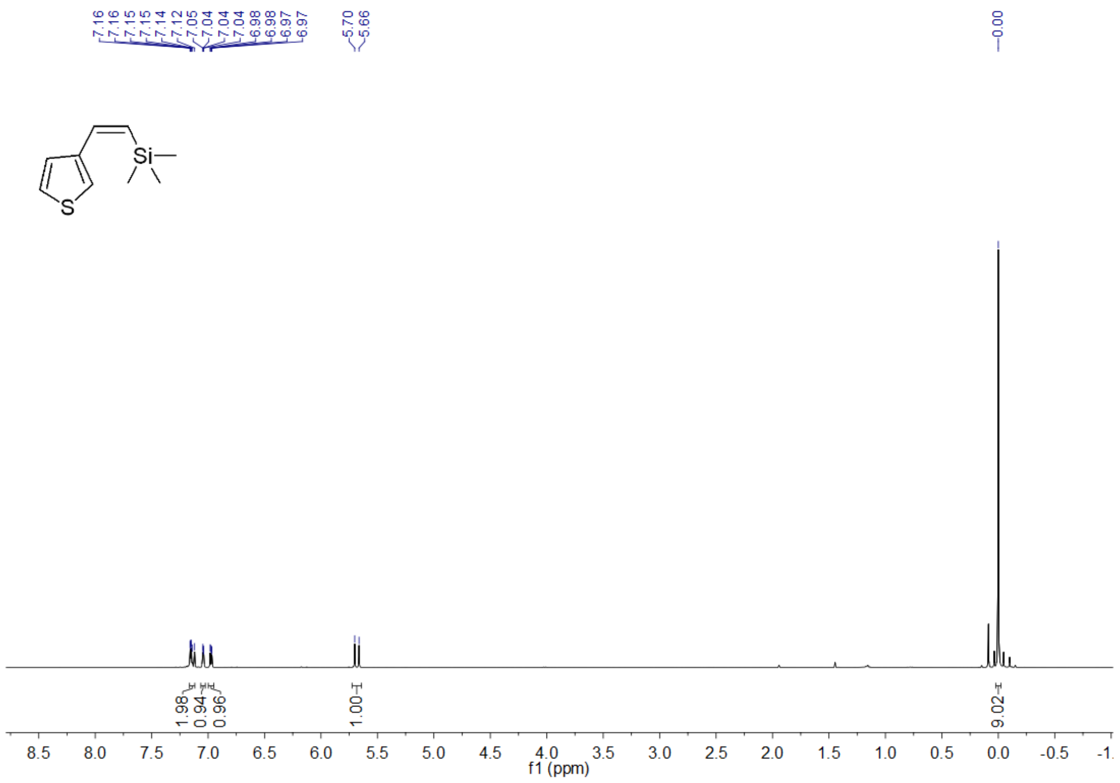

Supplementary Figure 36. ^{1}H NMR (400 MHz, $CDCl_3$) spectra for compound 1q

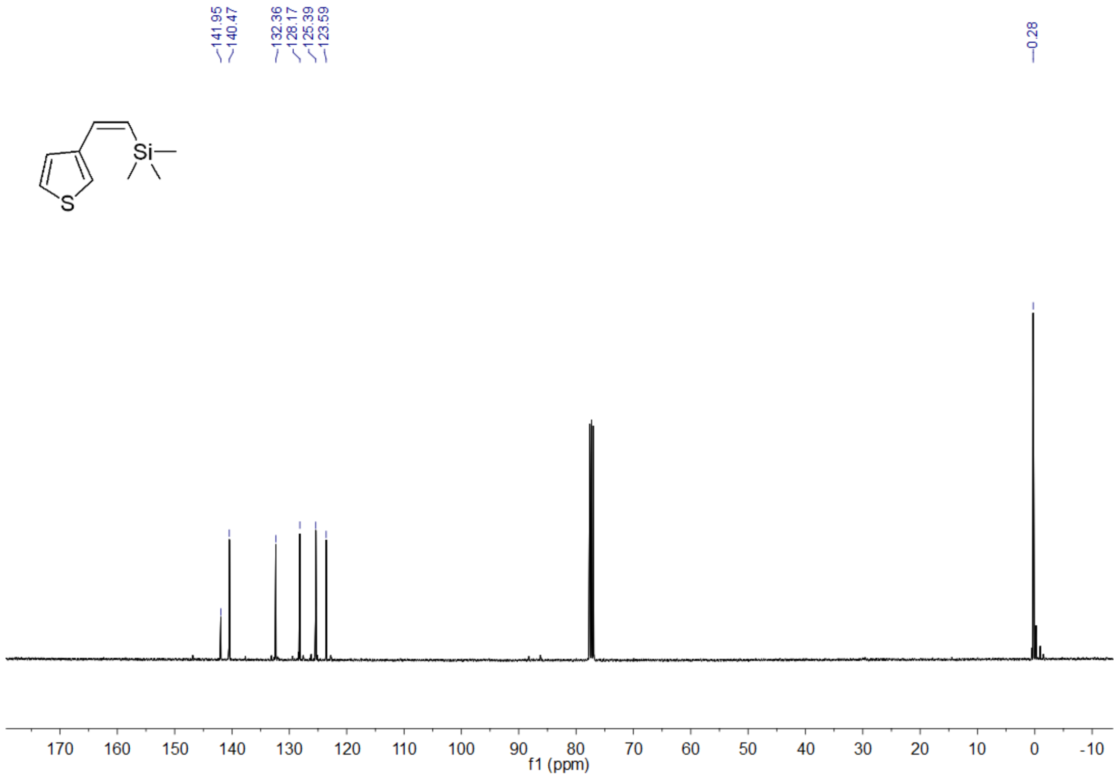

Supplementary Figure 37. ^{13}C NMR (400 MHz, $CDCl_3$) spectra for compound 1q

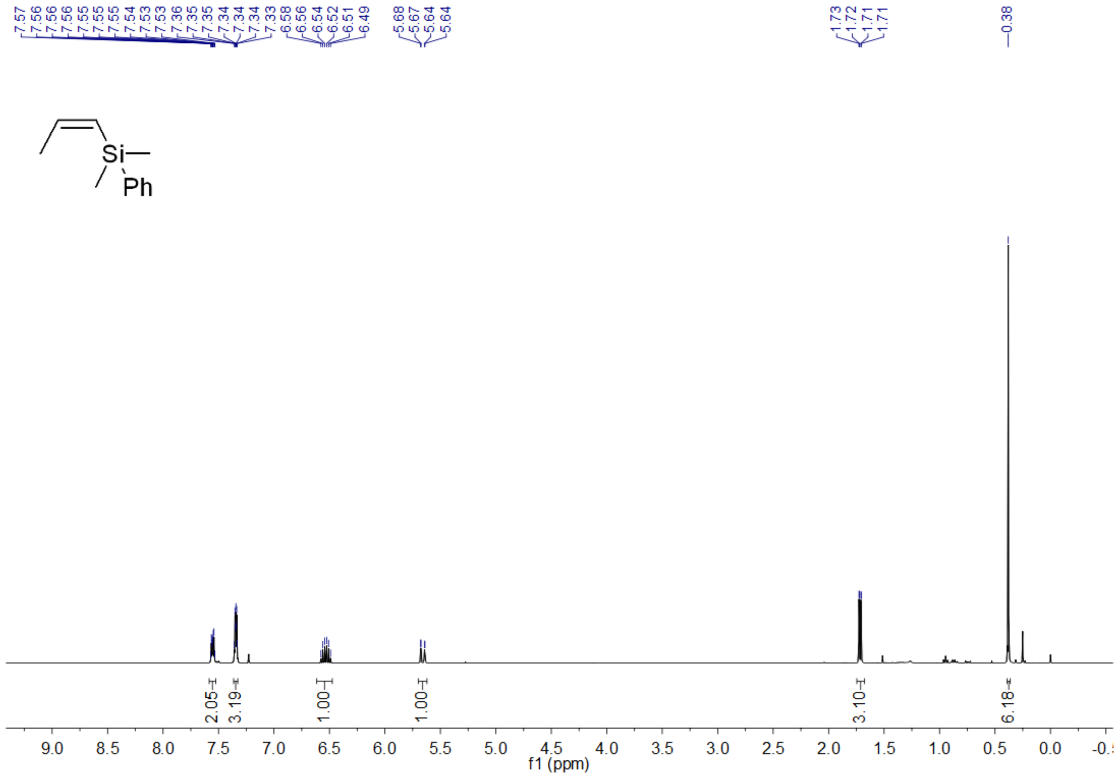

Supplementary Figure 38. ^{1}H NMR (400 MHz, $CDCl_3$) spectra for compound 1r

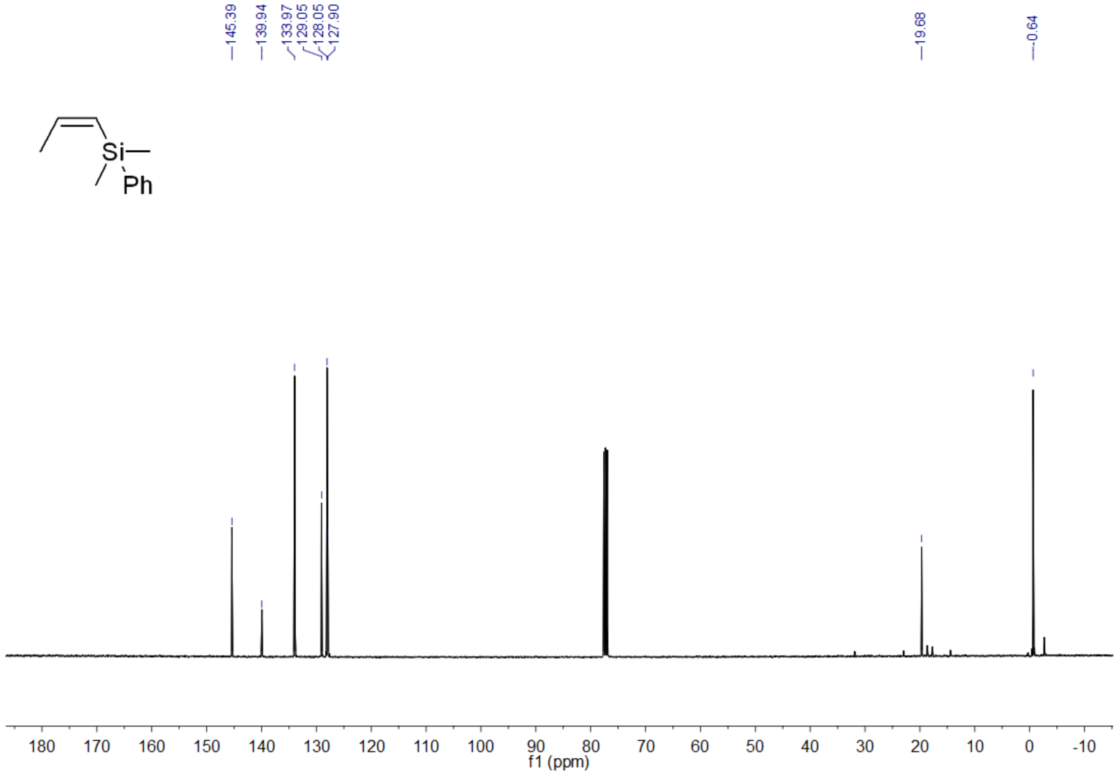

Supplementary Figure 39. ^{13}C NMR (400 MHz, $CDCl_3$) spectra for compound 1r

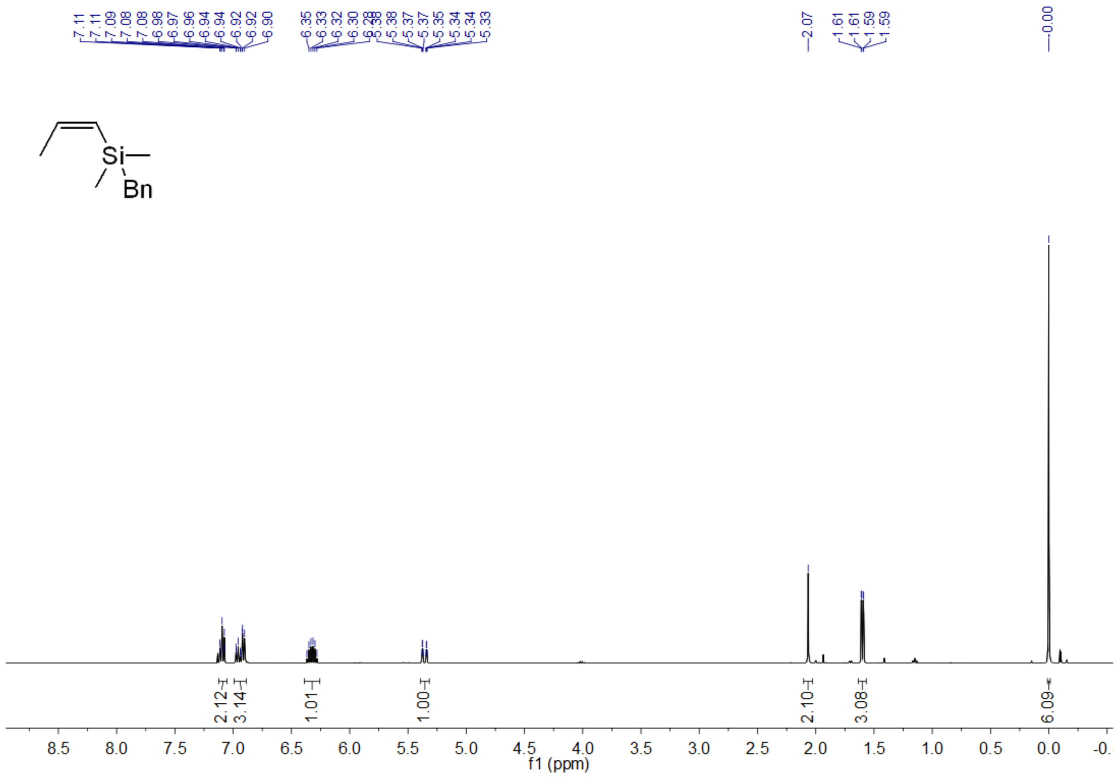

Supplementary Figure 40. ^{1}H NMR (400 MHz, $CDCl_3$) spectra for compound 1s

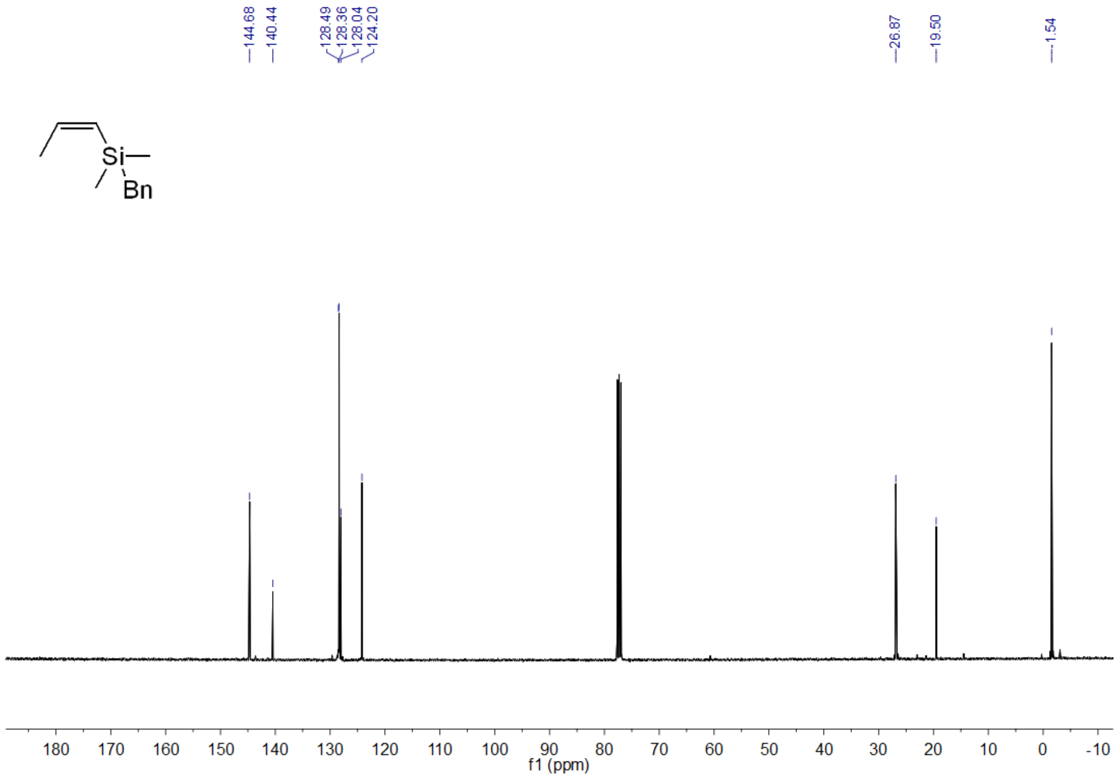

Supplementary Figure 41. ^{13}C NMR (400 MHz, $CDCl_3$) spectra for compound 1s

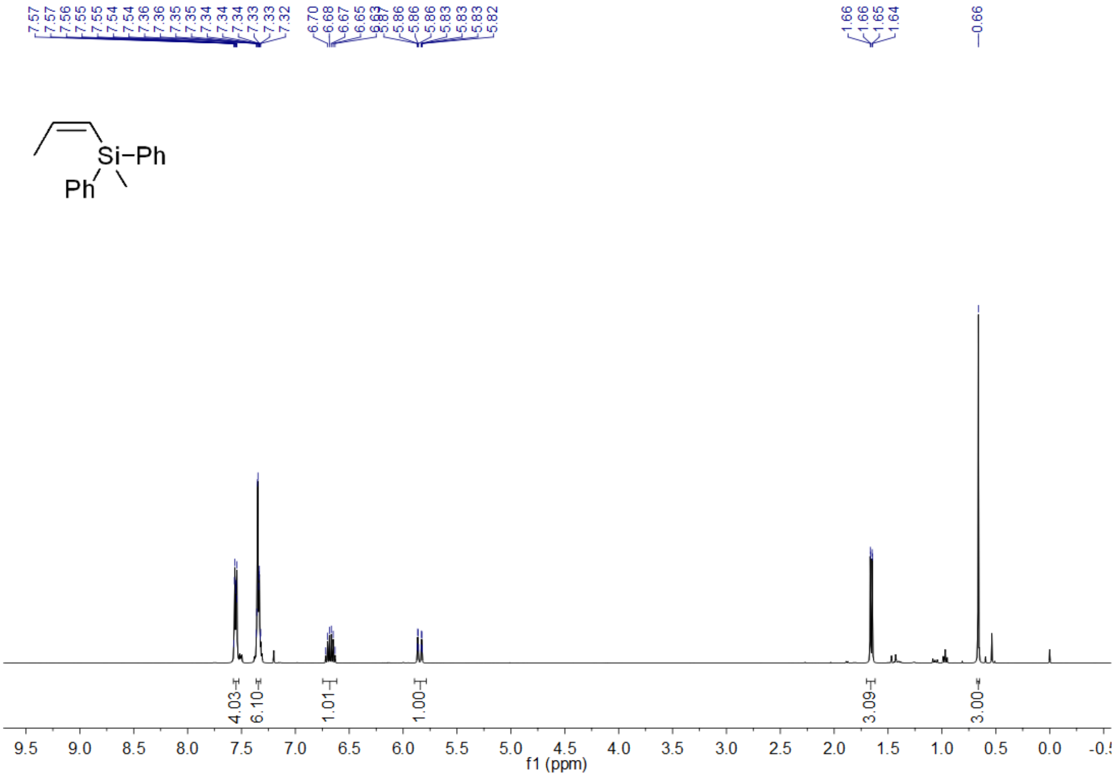

Supplementary Figure 42. ^{1}H **NMR (400 MHz,** $CDCl_3$**) spectra for compound 1t**

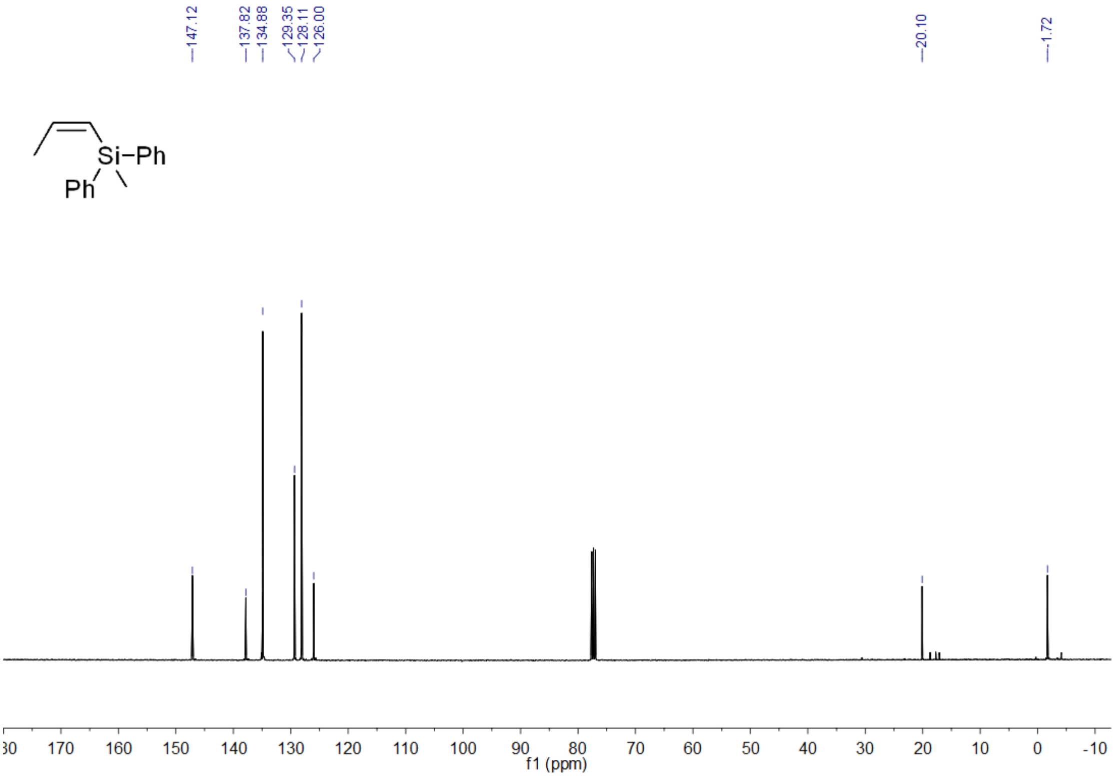

Supplementary Figure 43. ^{13}C **NMR (400 MHz,** $CDCl_3$**) spectra for compound 1t**

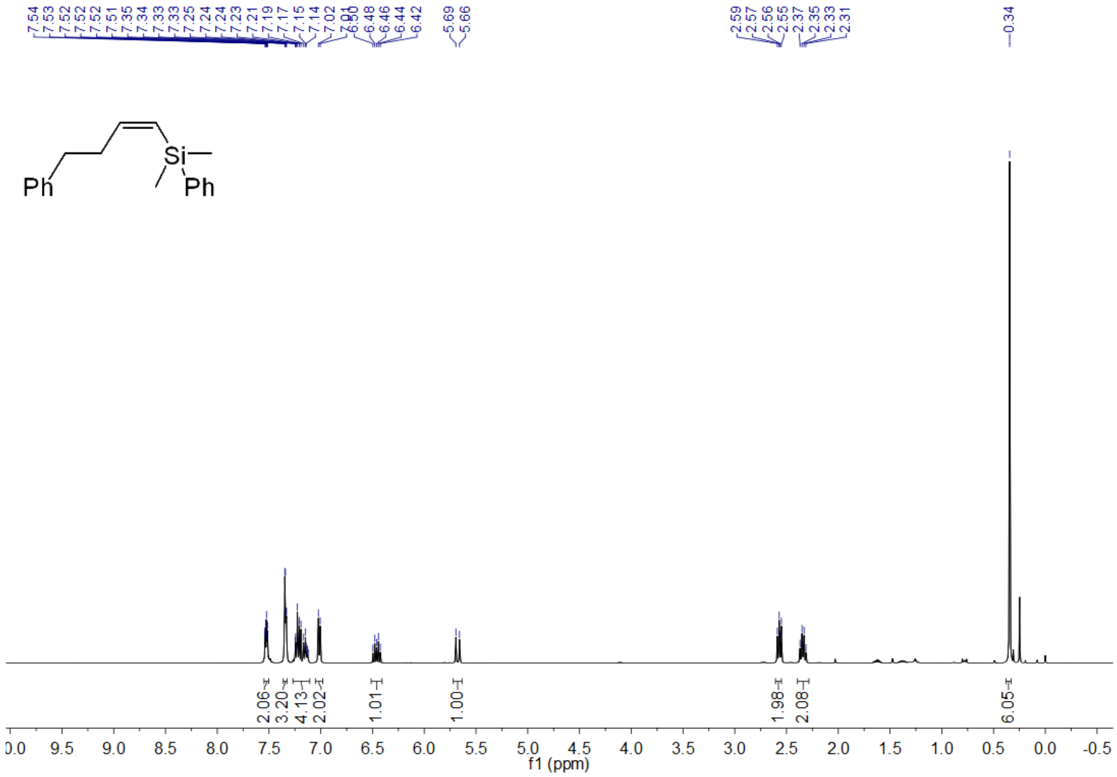

Supplementary Figure 44. ^{1}H NMR (400 MHz, $CDCl_3$) spectra for compound 1u

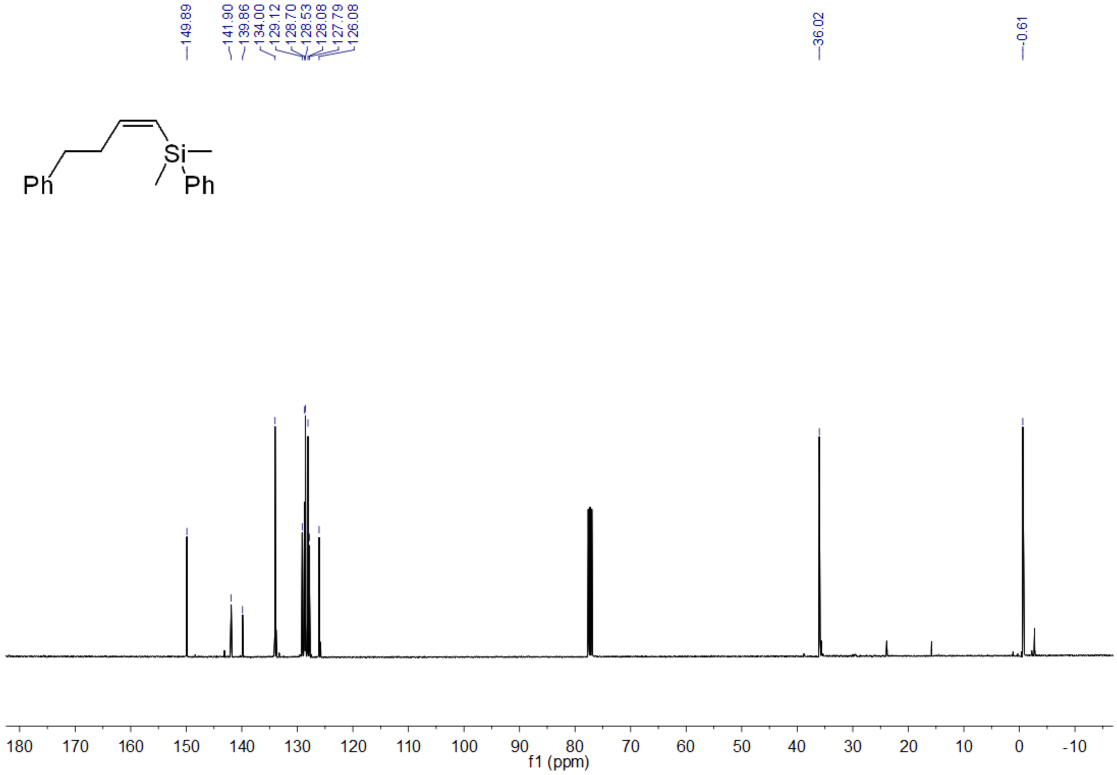

Supplementary Figure 45. ^{13}C NMR (400 MHz, $CDCl_3$) spectra for compound 1u

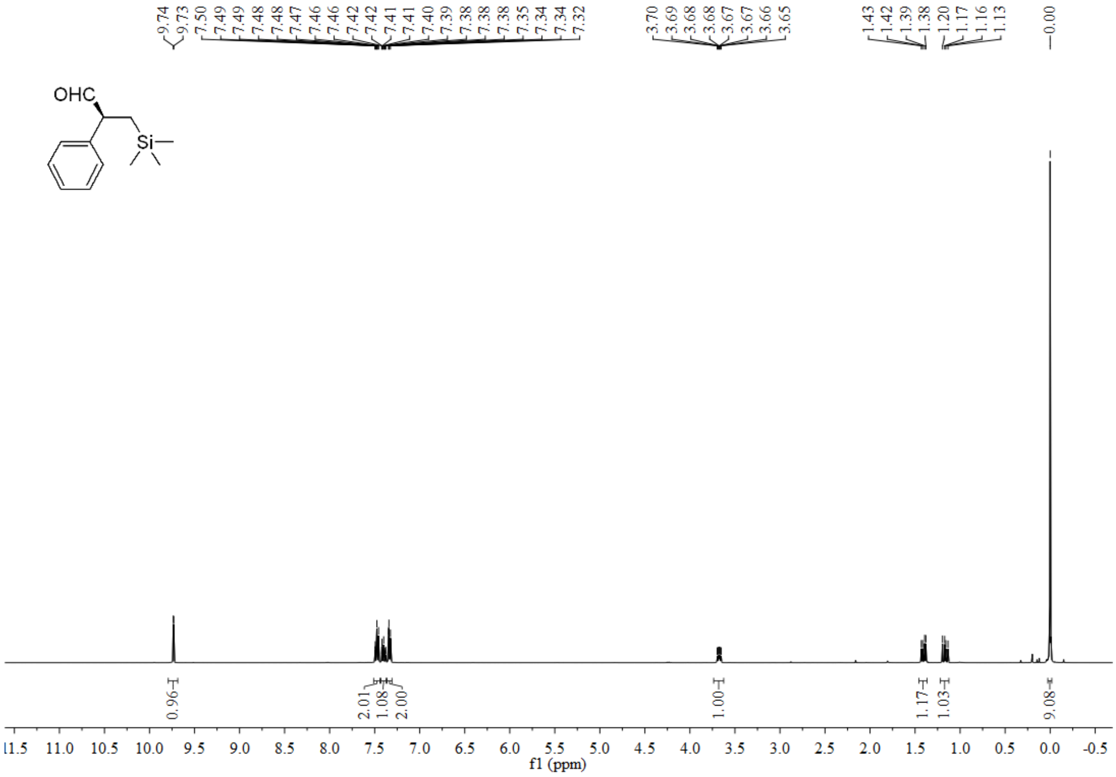

Supplementary Figure 46. ^{1}H NMR (400 MHz, $CDCl_3$) spectra for compound 2a

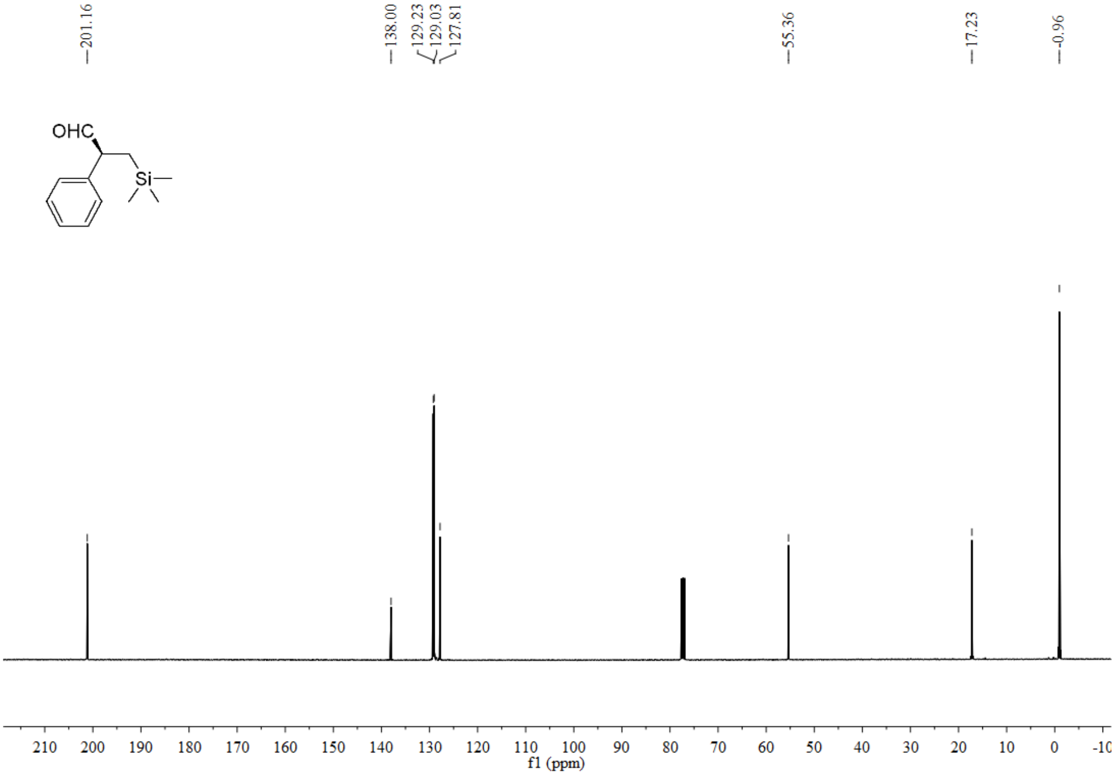

Supplementary Figure 47. ^{13}C NMR (400 MHz, $CDCl_3$) spectra for compound 2a

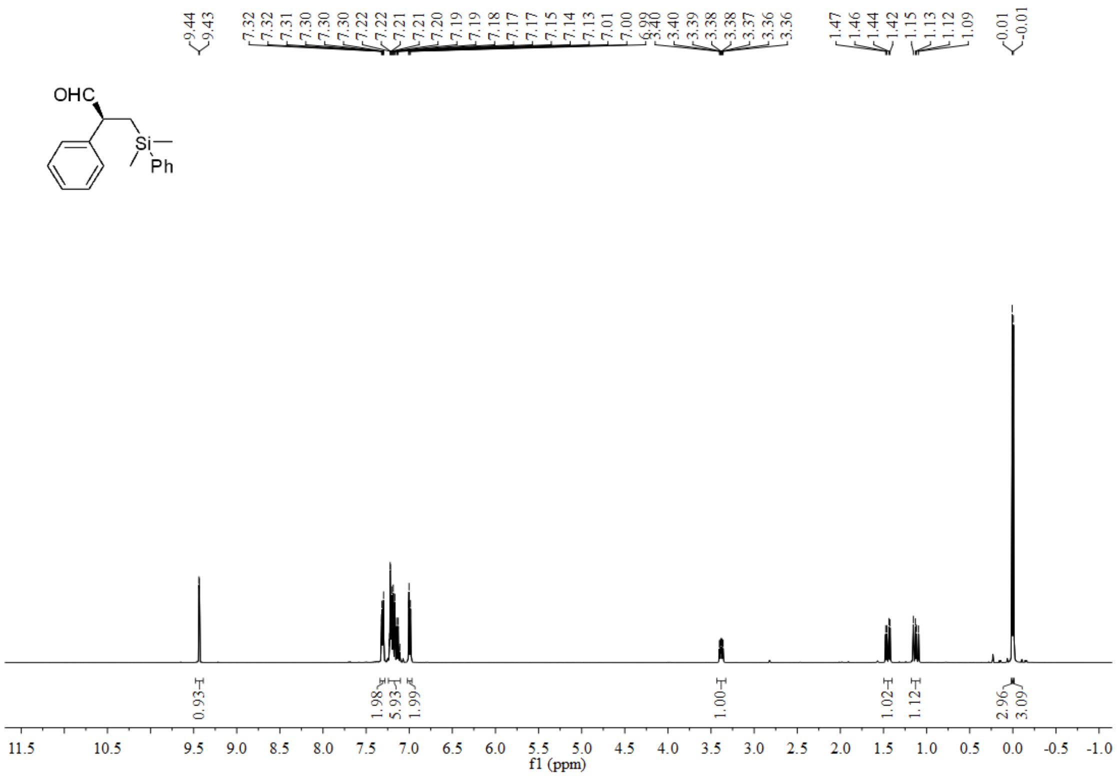

Supplementary Figure 48. ^{1}H NMR (400 MHz, $CDCl_3$) spectra for compound 2b

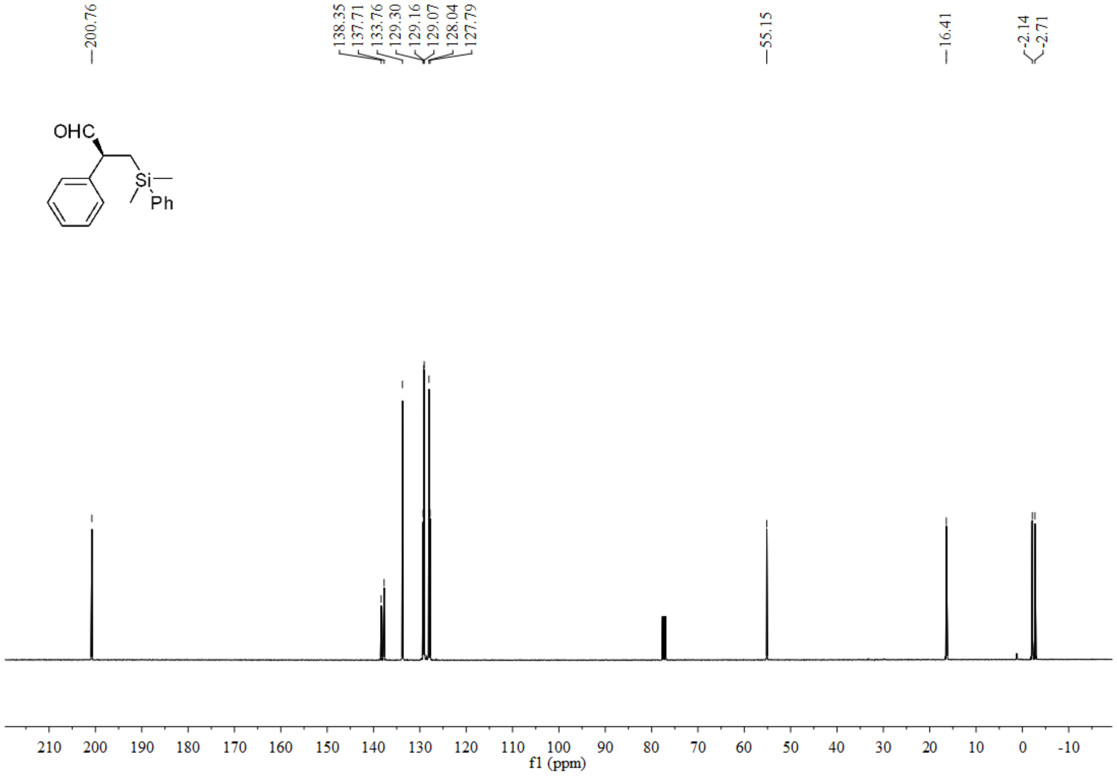

Supplementary Figure 49. ^{13}C NMR (400 MHz, $CDCl_3$) spectra for compound 2b

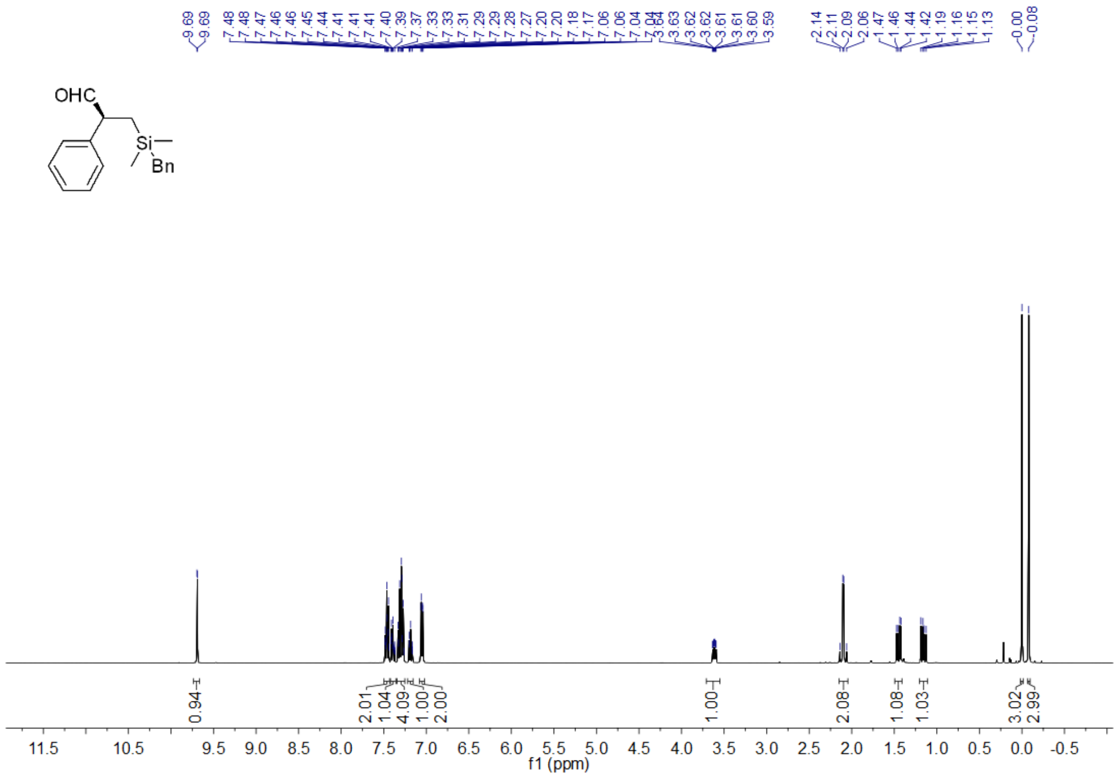

Supplementary Figure 50. ^{1}H **NMR (400 MHz, CDCl**$_{3}$**) spectra for compound 2c**

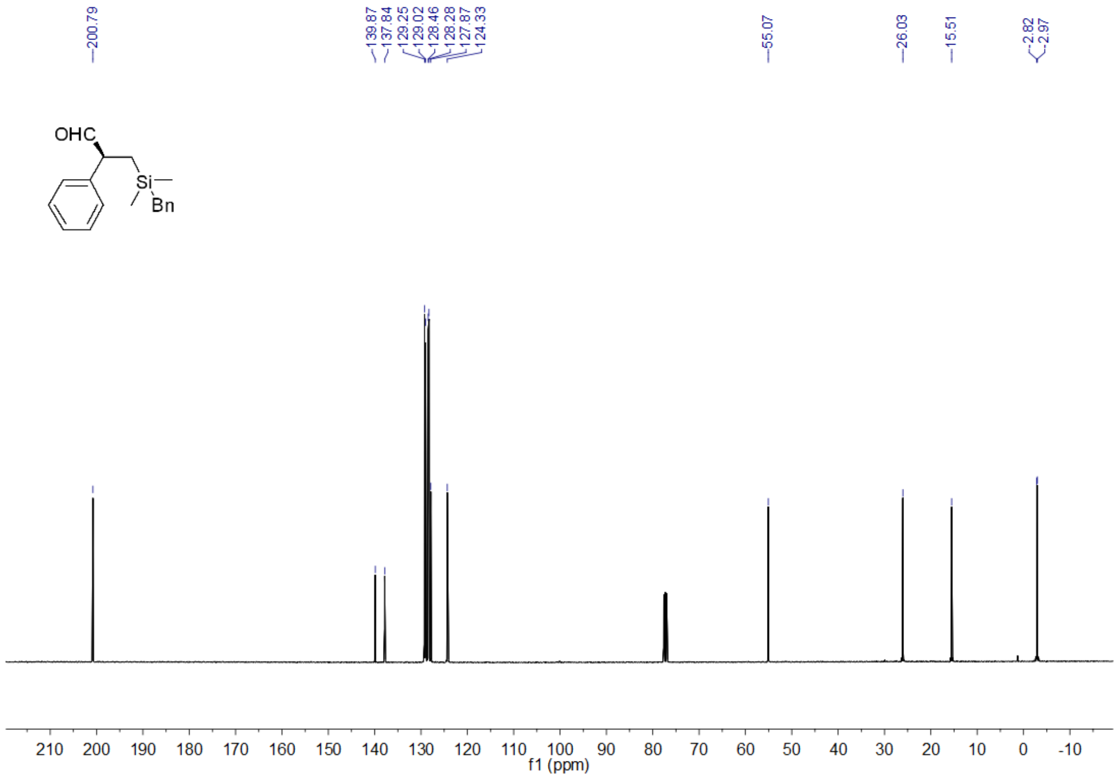

Supplementary Figure 51. ^{13}C **NMR (400 MHz, CDCl**$_{3}$**) spectra for compound 2c**

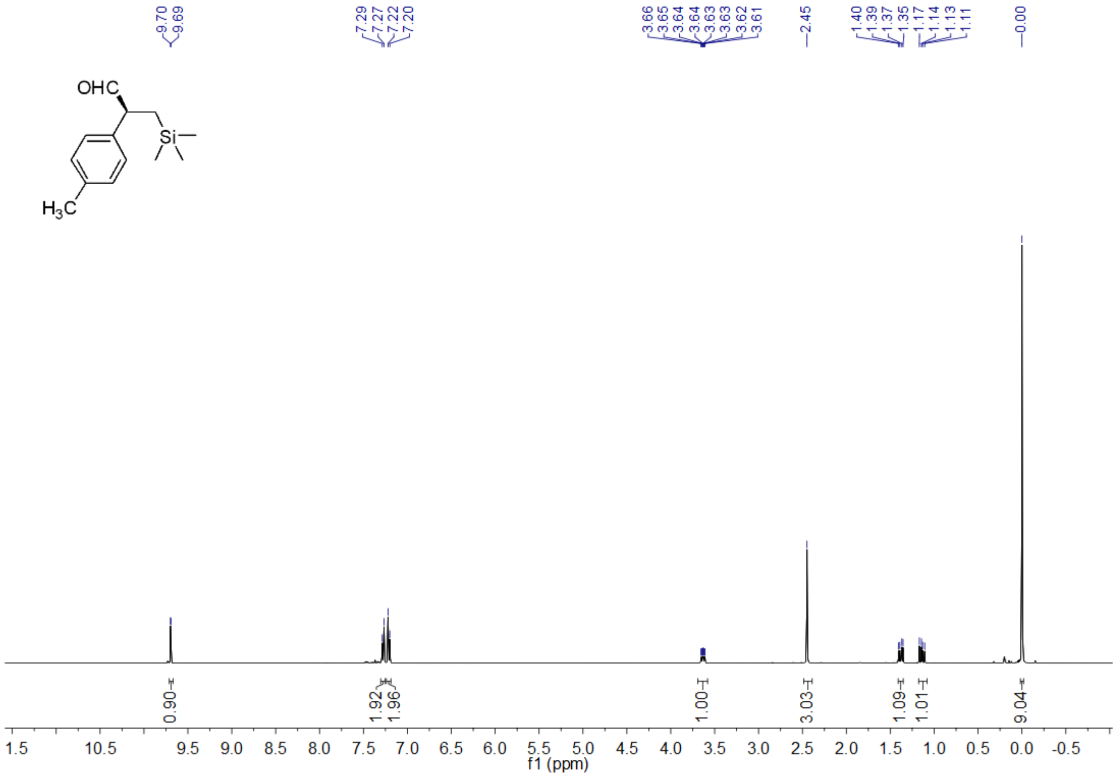

Supplementary Figure 52. ^{1}H **NMR (400 MHz,** $CDCl_3$**) spectra for compound 2d**

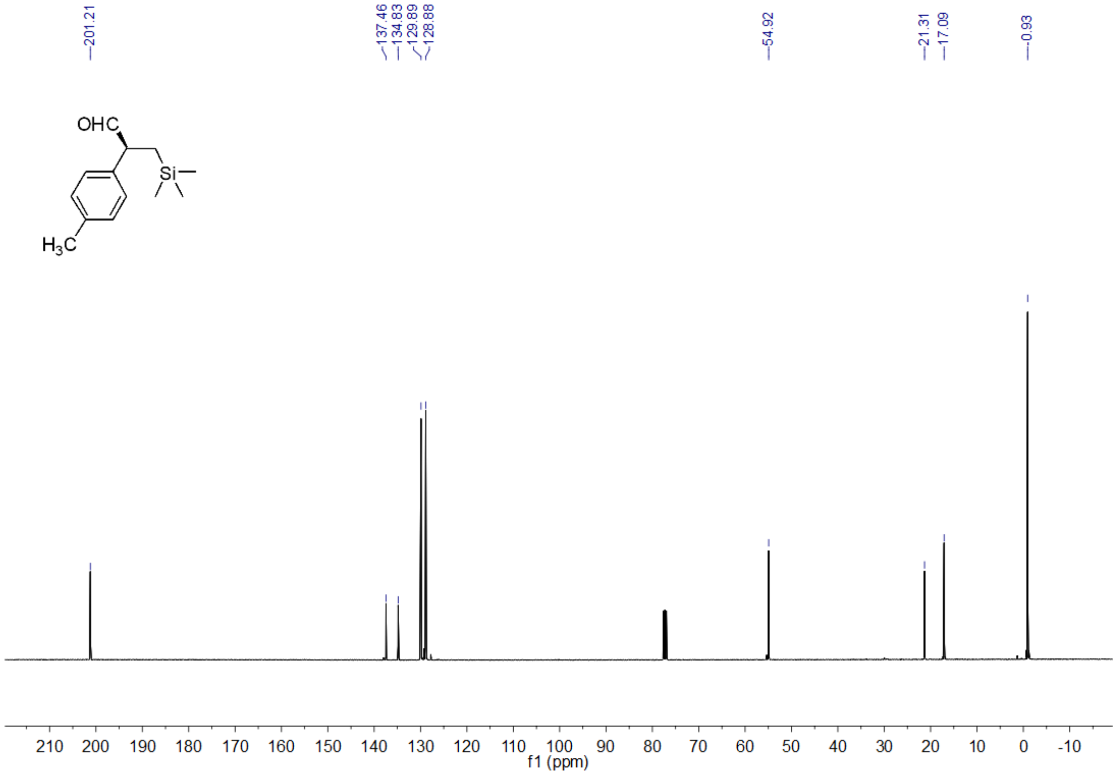

Supplementary Figure 53. ^{13}C **NMR (400 MHz,** $CDCl_3$**) spectra for compound 2d**

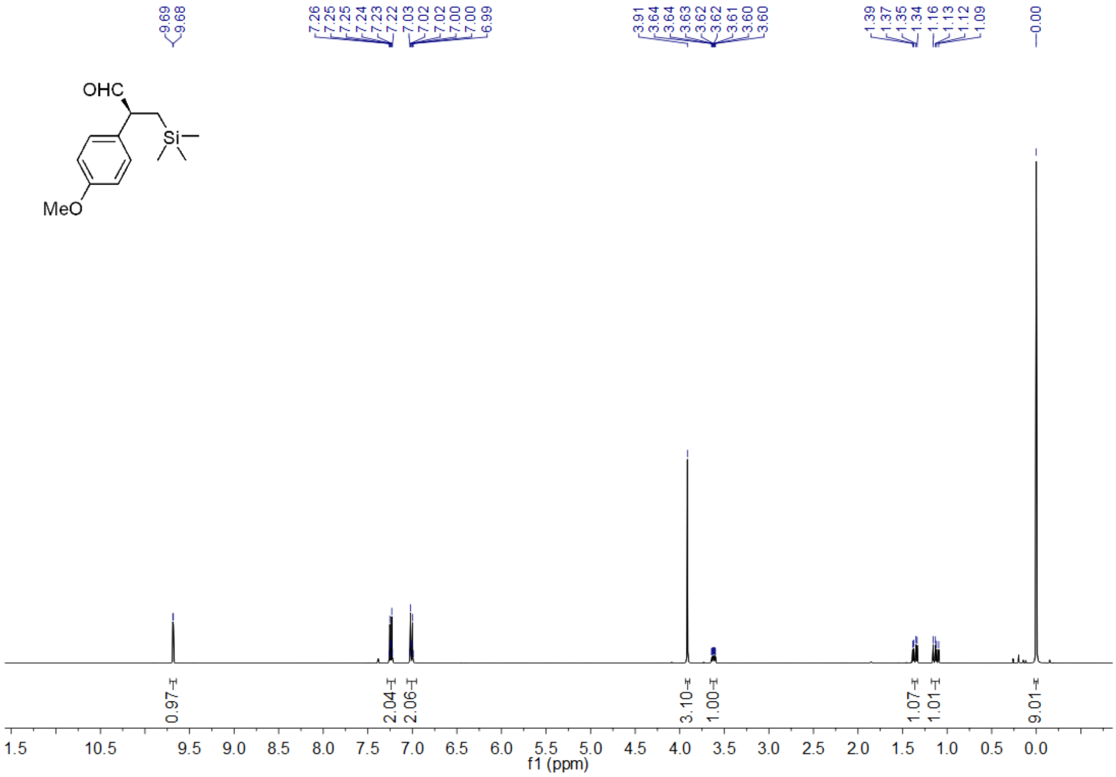

Supplementary Figure 54. ^{1}H NMR (400 MHz, $CDCl_3$) spectra for compound 2e

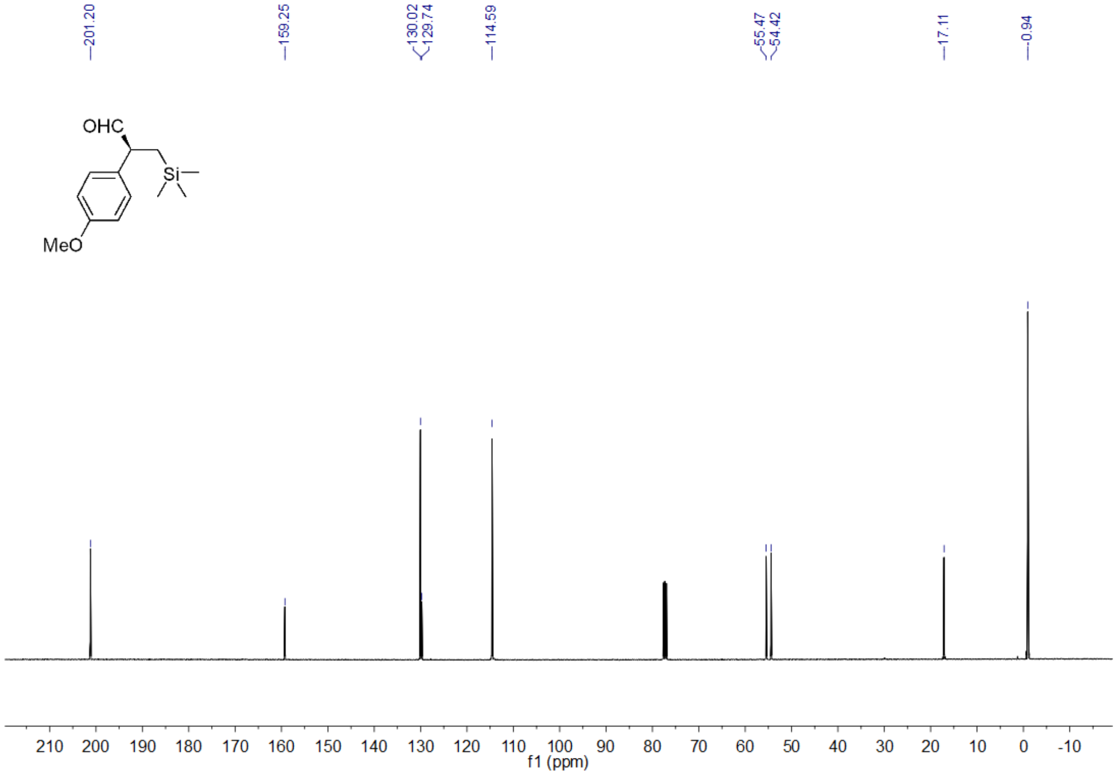

Supplementary Figure 55. ^{13}C NMR (400 MHz, $CDCl_3$) spectra for compound 2e

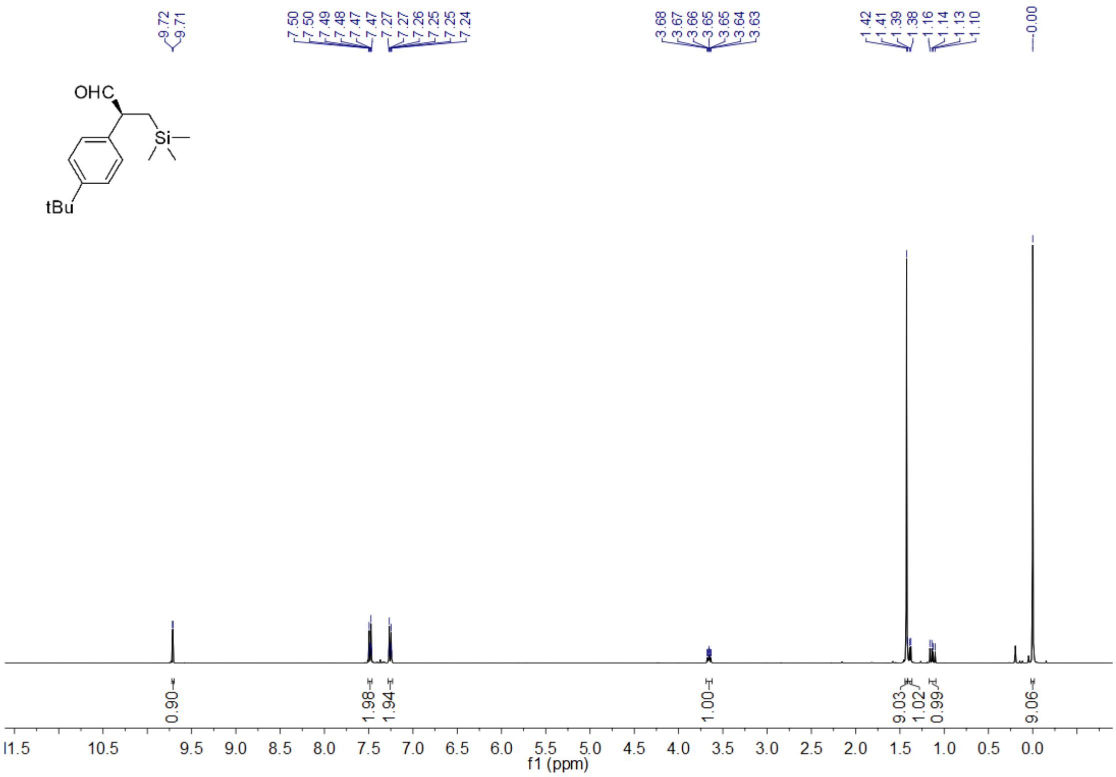

Supplementary Figure 56. ^{1}H NMR (400 MHz, CDCl$_3$) spectra for compound 2f

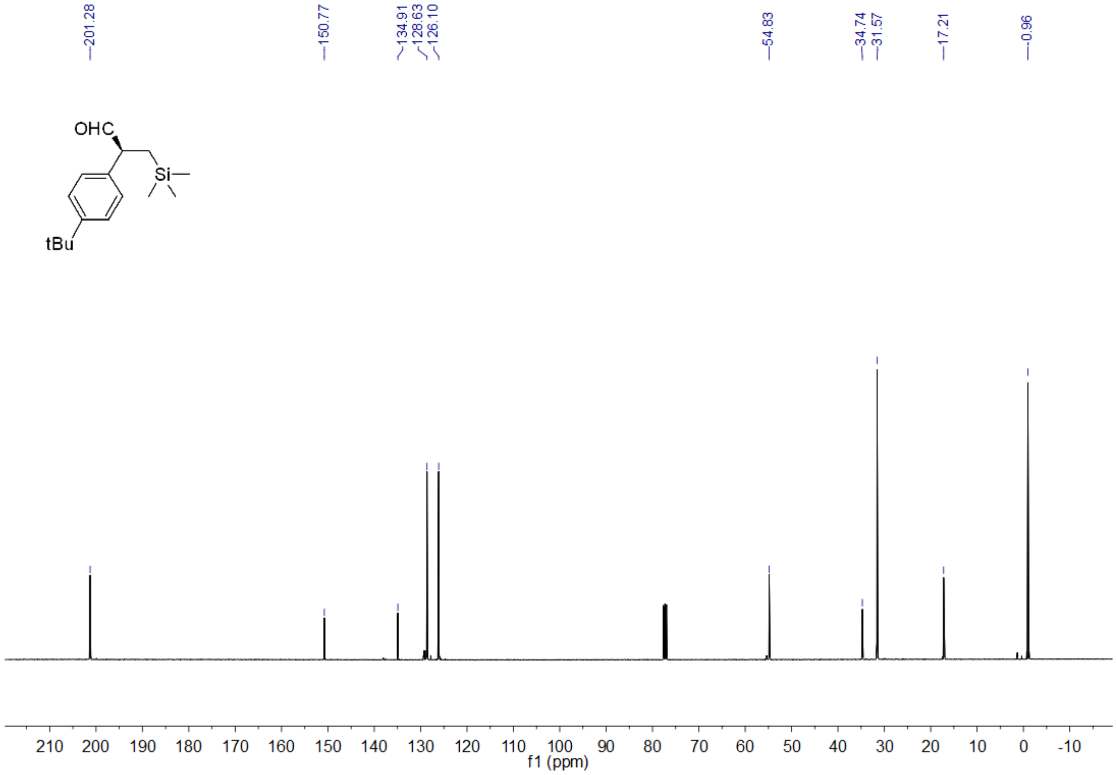

Supplementary Figure 57. ^{13}C NMR (400 MHz, CDCl$_3$) spectra for compound 2f

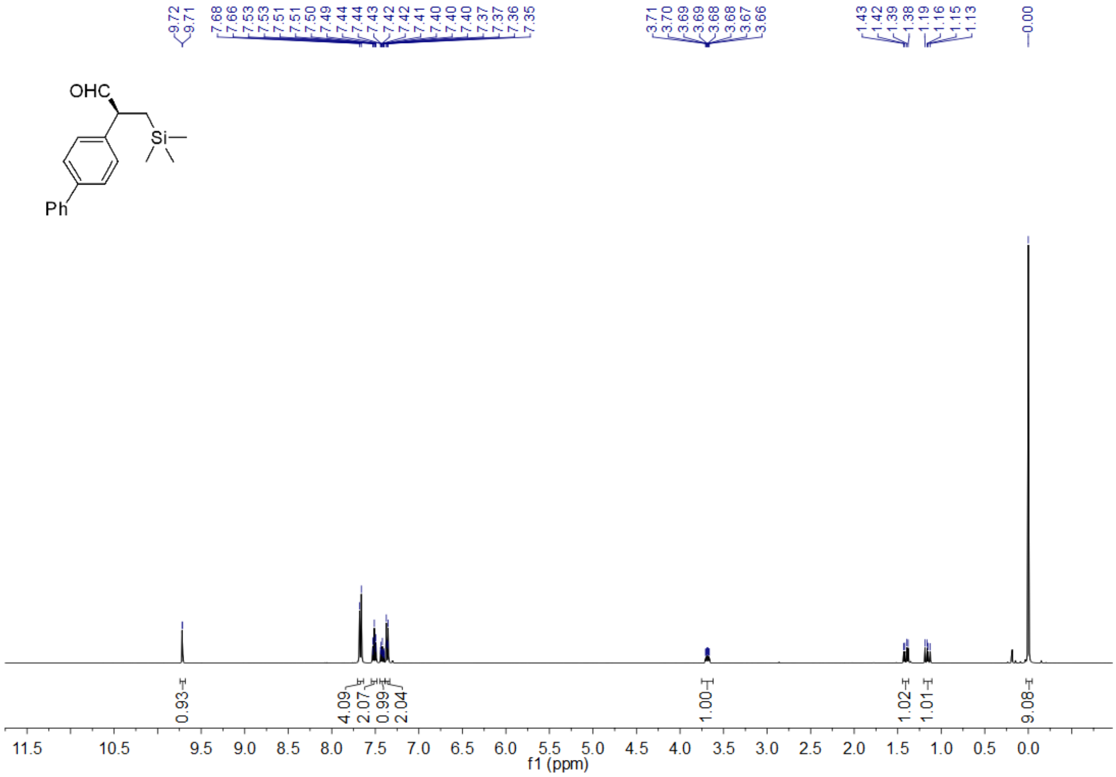

Supplementary Figure 58. ^{1}H NMR (400 MHz, $CDCl_3$) spectra for compound 2g

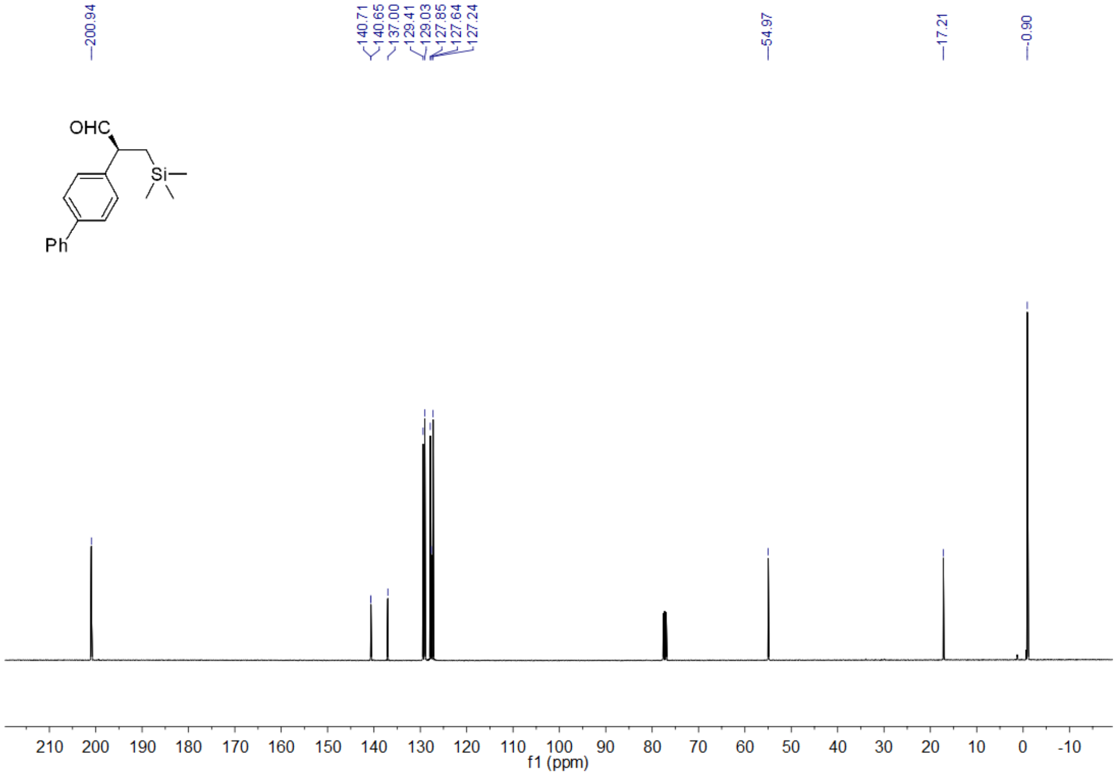

Supplementary Figure 59. ^{13}C NMR (400 MHz, $CDCl_3$) spectra for compound 2g

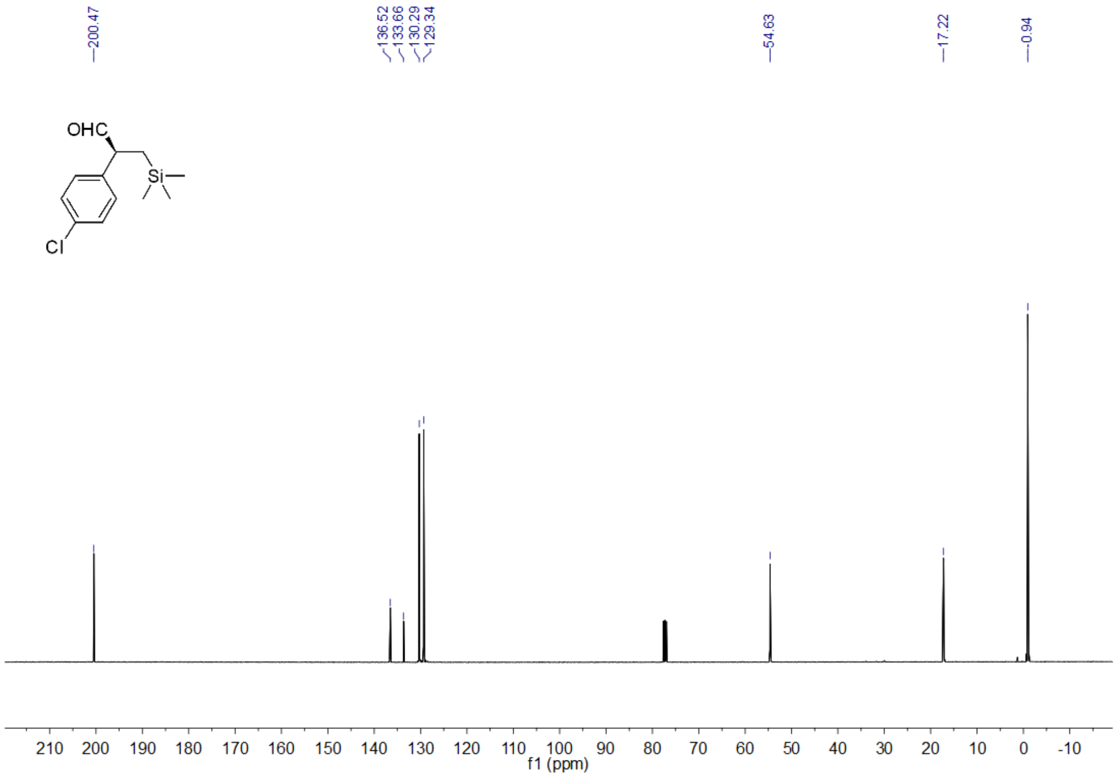

Supplementary Figure 60. ^{1}H NMR (400 MHz, $CDCl_3$) spectra for compound 2h

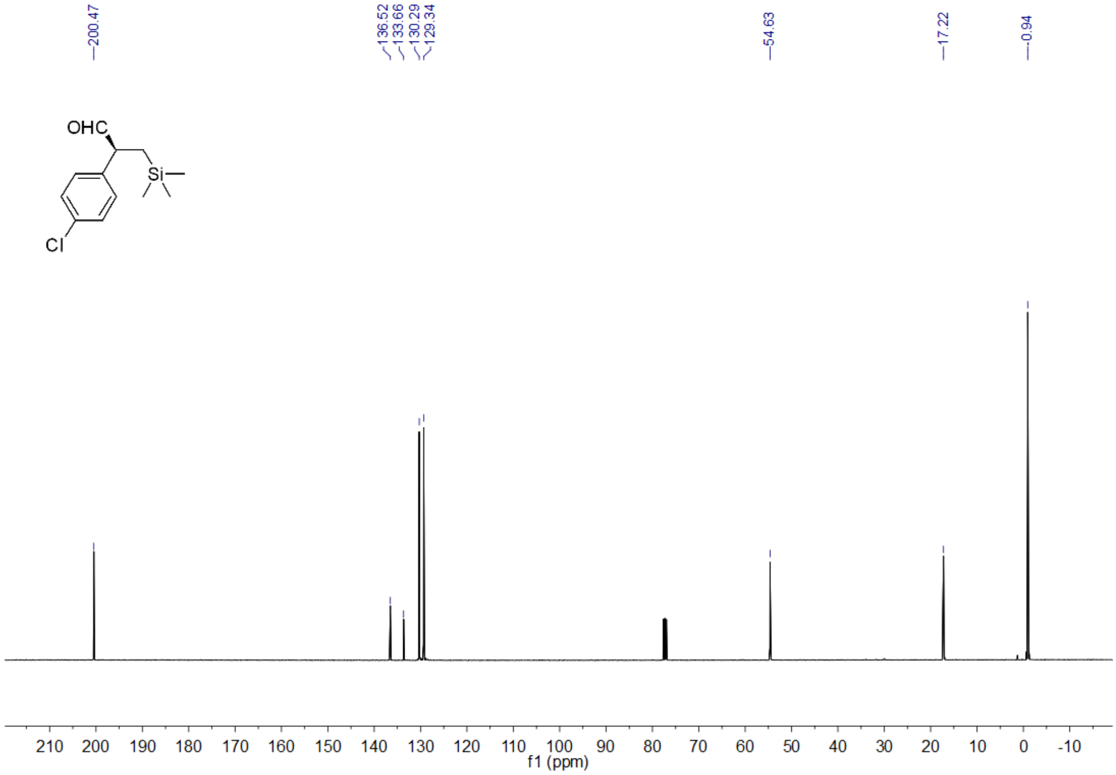

Supplementary Figure 61. ^{13}C NMR (400 MHz, $CDCl_3$) spectra for compound 2h

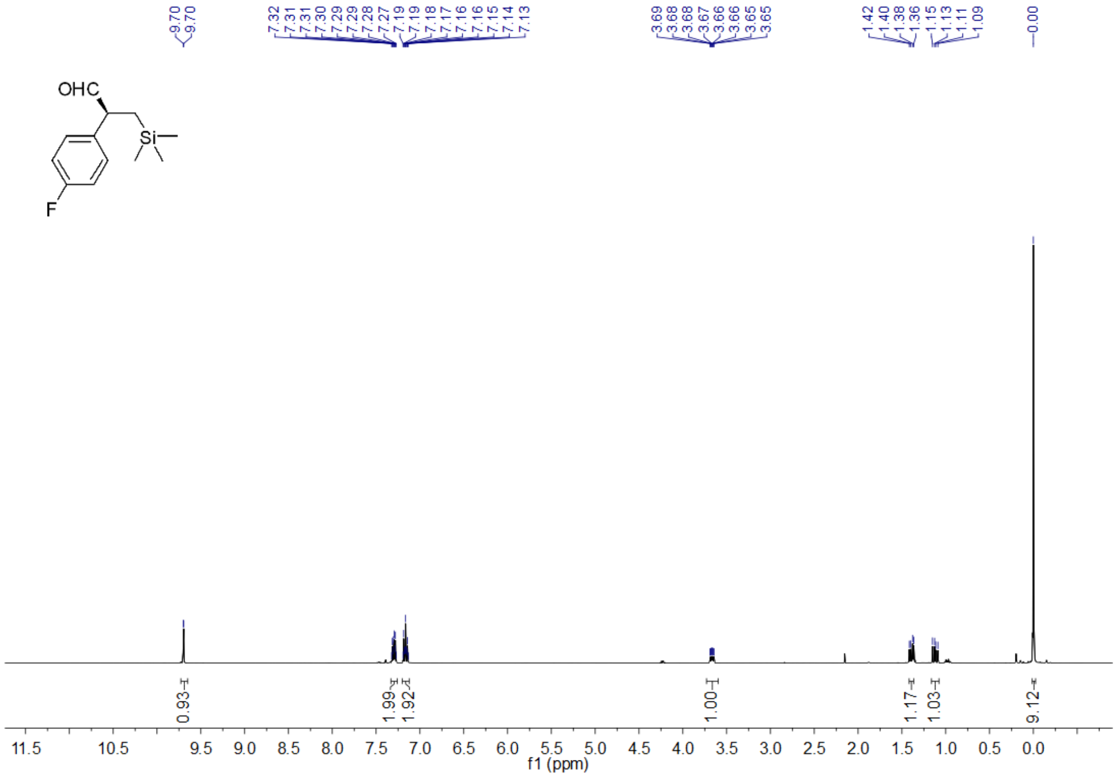

Supplementary Figure 62. ^{1}**H NMR (400 MHz, $CDCl_3$) spectra for compound 2i**

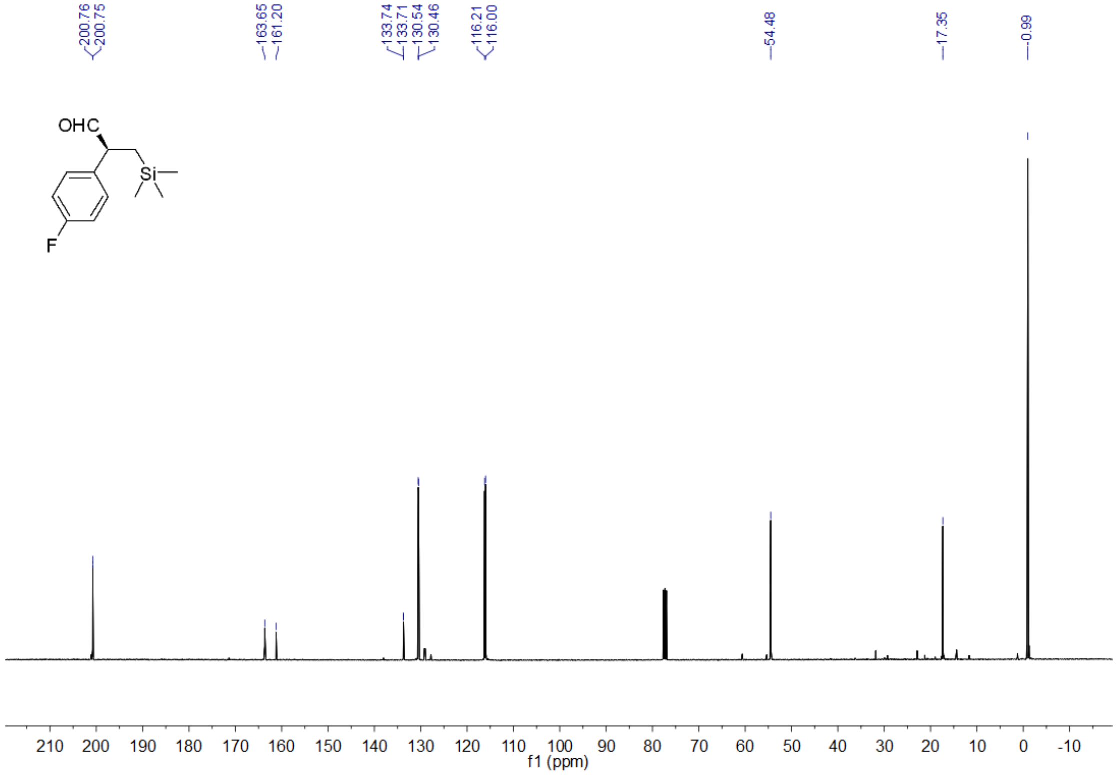

Supplementary Figure 63. ^{13}**C NMR (400 MHz, $CDCl_3$) spectra for compound 2i**

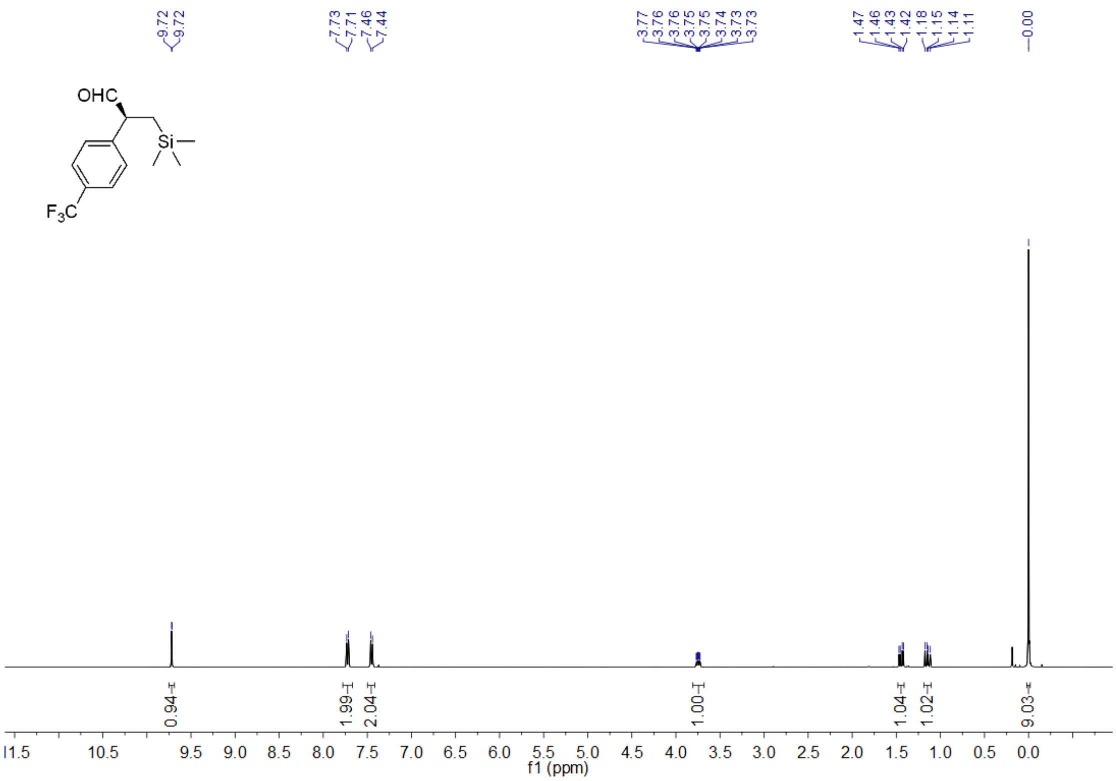

Supplementary Figure 64. ^{1}H NMR (400 MHz, $CDCl_3$) spectra for compound 2j

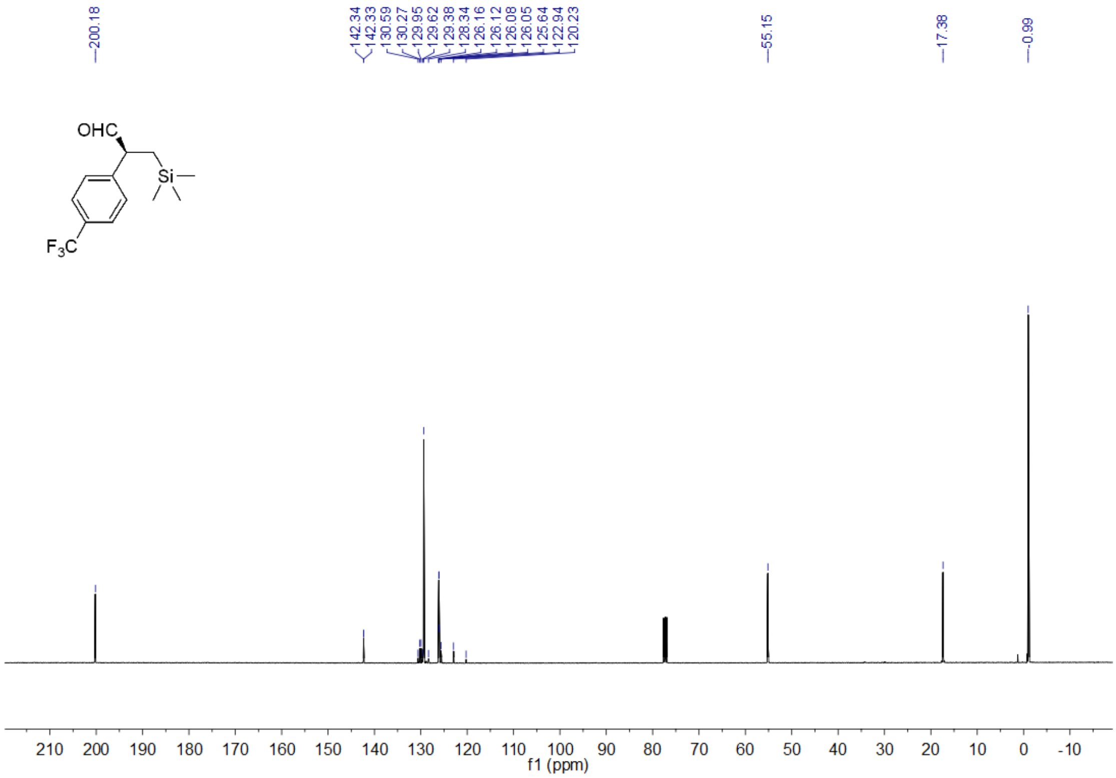

Supplementary Figure 65. ^{13}C NMR (400 MHz, $CDCl_3$) spectra for compound 2j

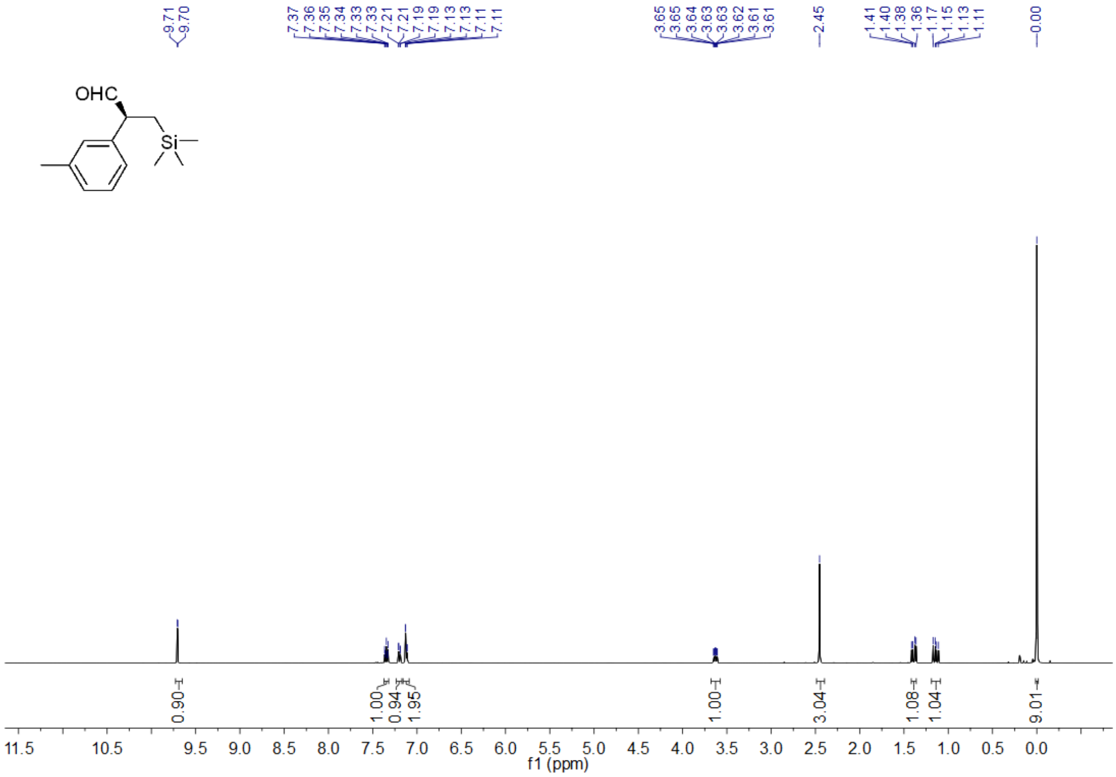

Supplementary Figure 66. ^{1}H NMR (400 MHz, $CDCl_3$) spectra for compound 2k

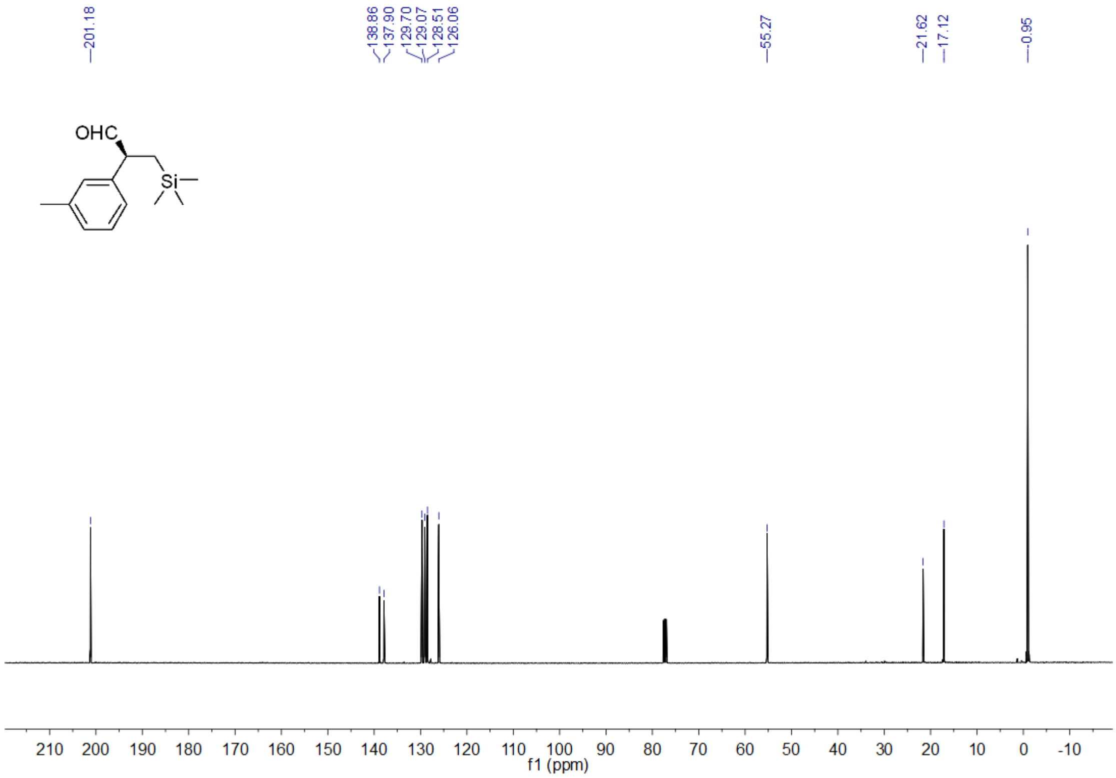

Supplementary Figure 67. ^{13}C NMR (400 MHz, $CDCl_3$) spectra for compound 2k

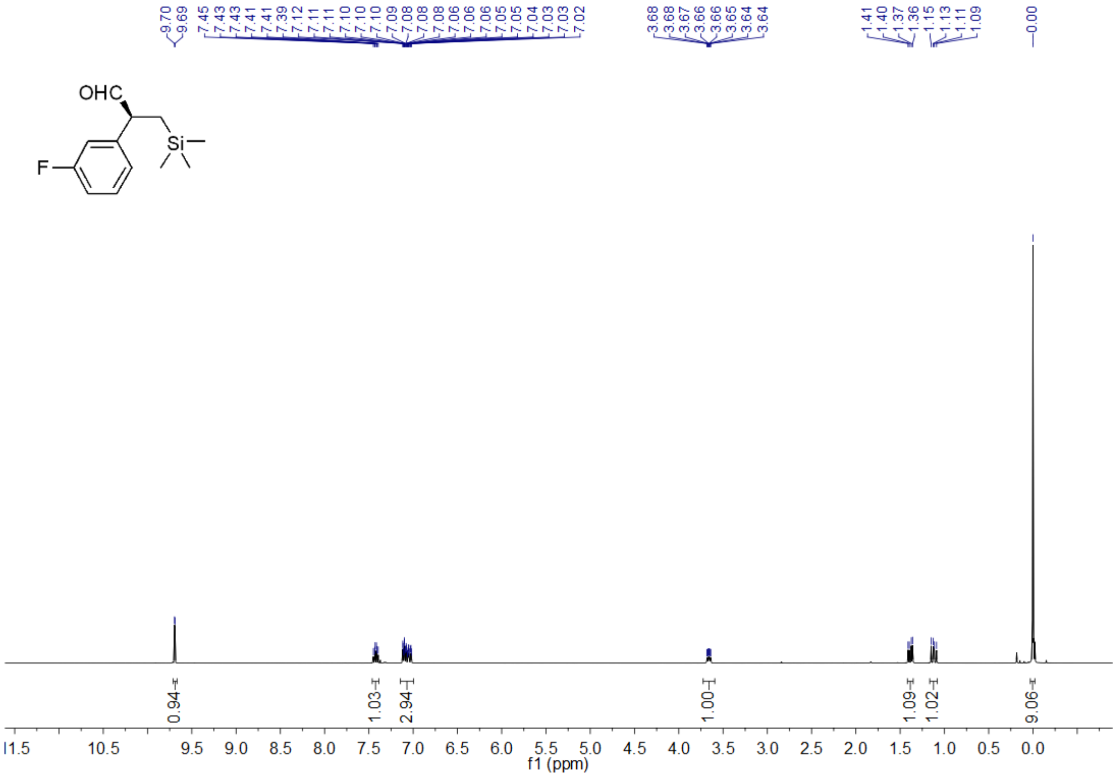

Supplementary Figure 68. ^{1}H NMR (400 MHz, $CDCl_3$) spectra for compound 2l

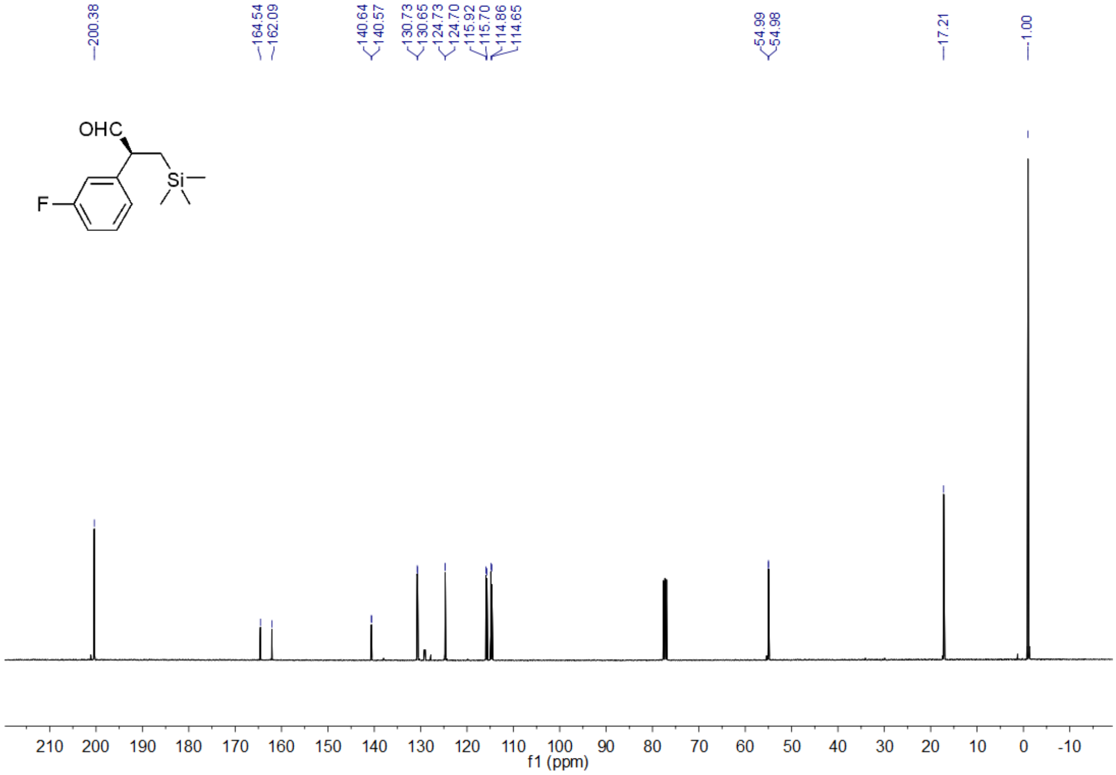

Supplementary Figure 69. ^{13}C NMR (400 MHz, $CDCl_3$) spectra for compound 2l

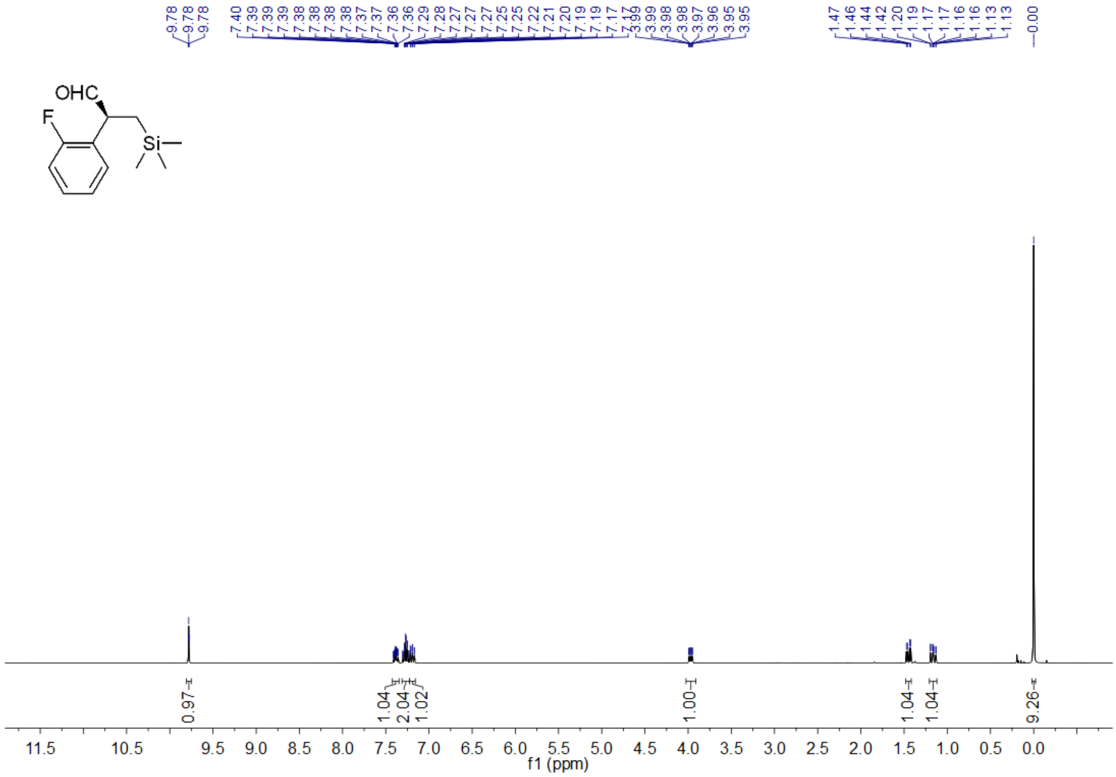

Supplementary Figure 70. ¹H NMR (400 MHz, CDCl₃) spectra for compound 2m

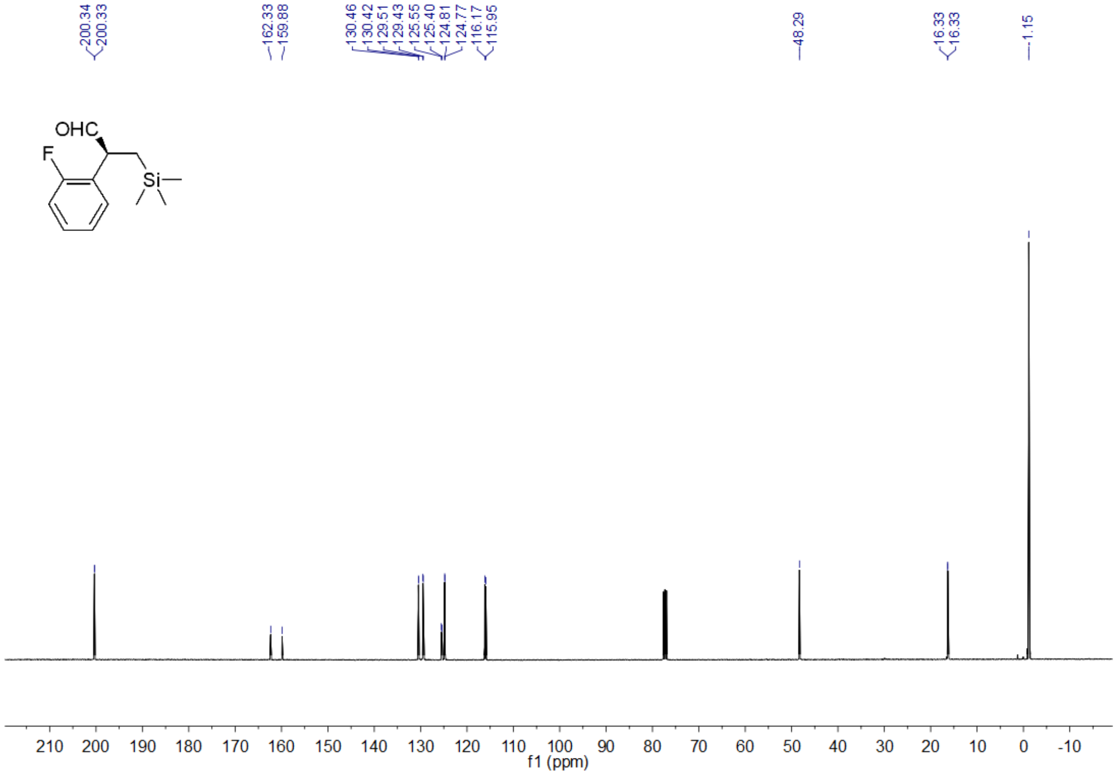

Supplementary Figure 71. ¹³C NMR (400 MHz, CDCl₃) spectra for compound 2m

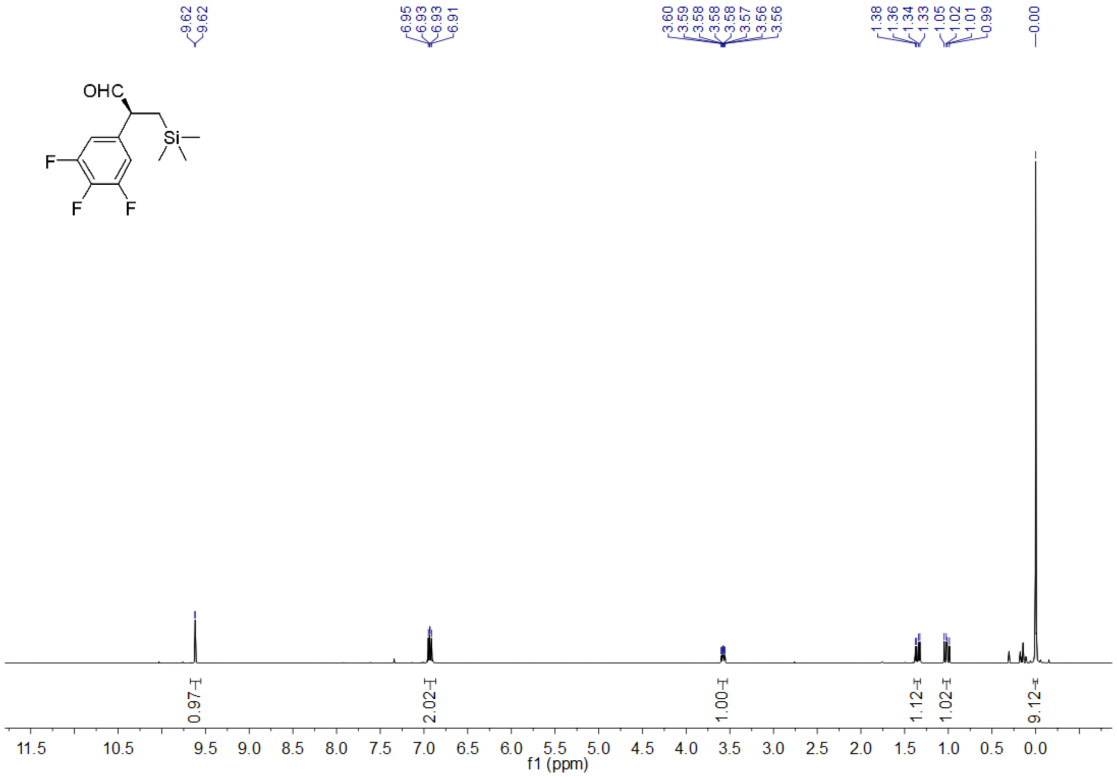

Supplementary Figure 72. ^{1}H **NMR (400 MHz,** $CDCl_3$**) spectra for compound 2n**

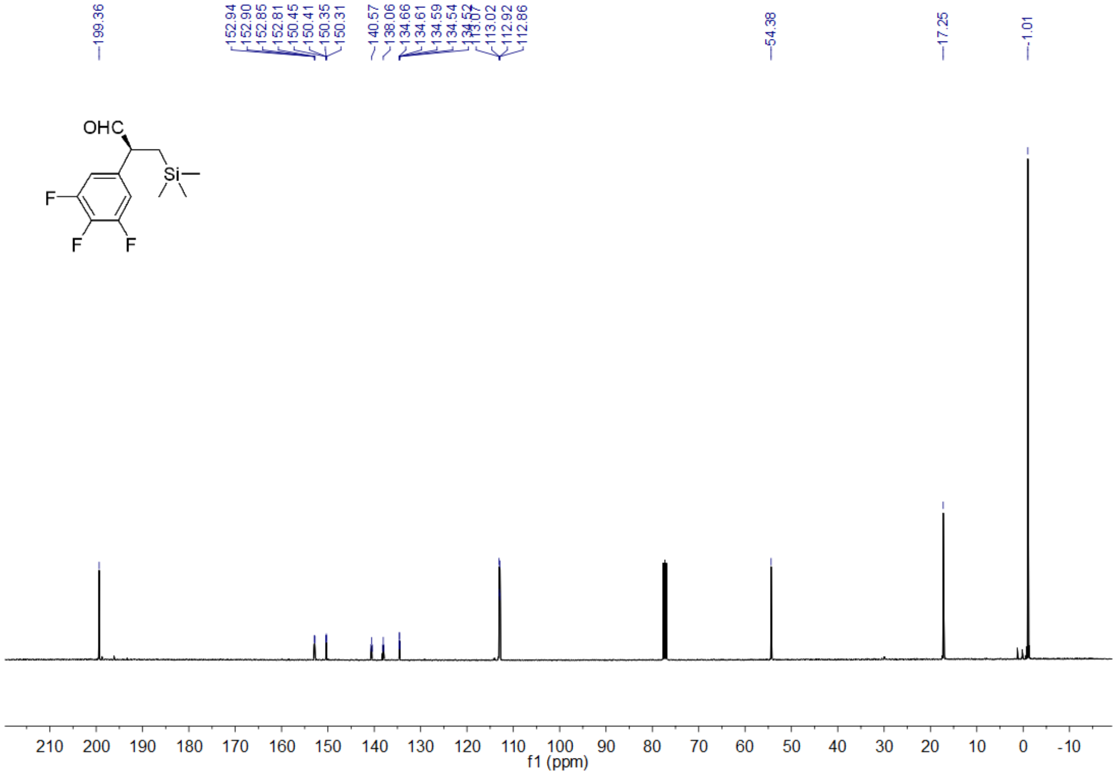

Supplementary Figure 73. ^{13}C **NMR (400 MHz,** $CDCl_3$**) spectra for compound 2n**

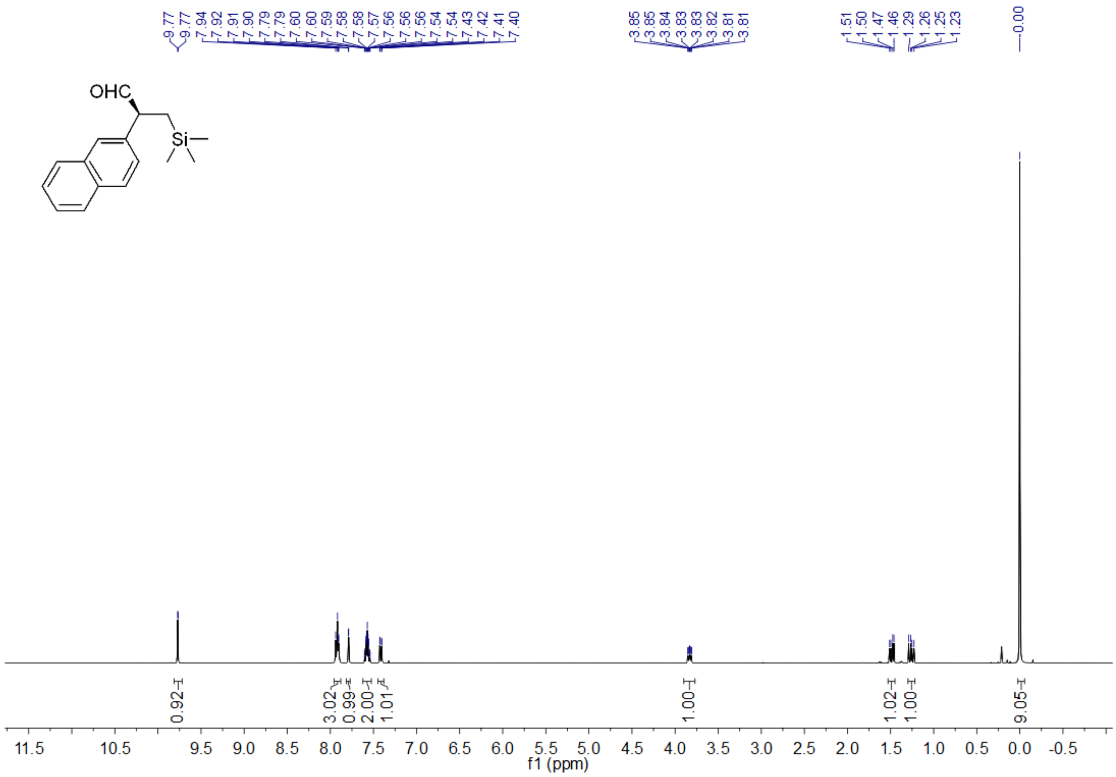

Supplementary Figure 74. ^{1}H **NMR (400 MHz,** $CDCl_3$**) spectra for compound 2o**

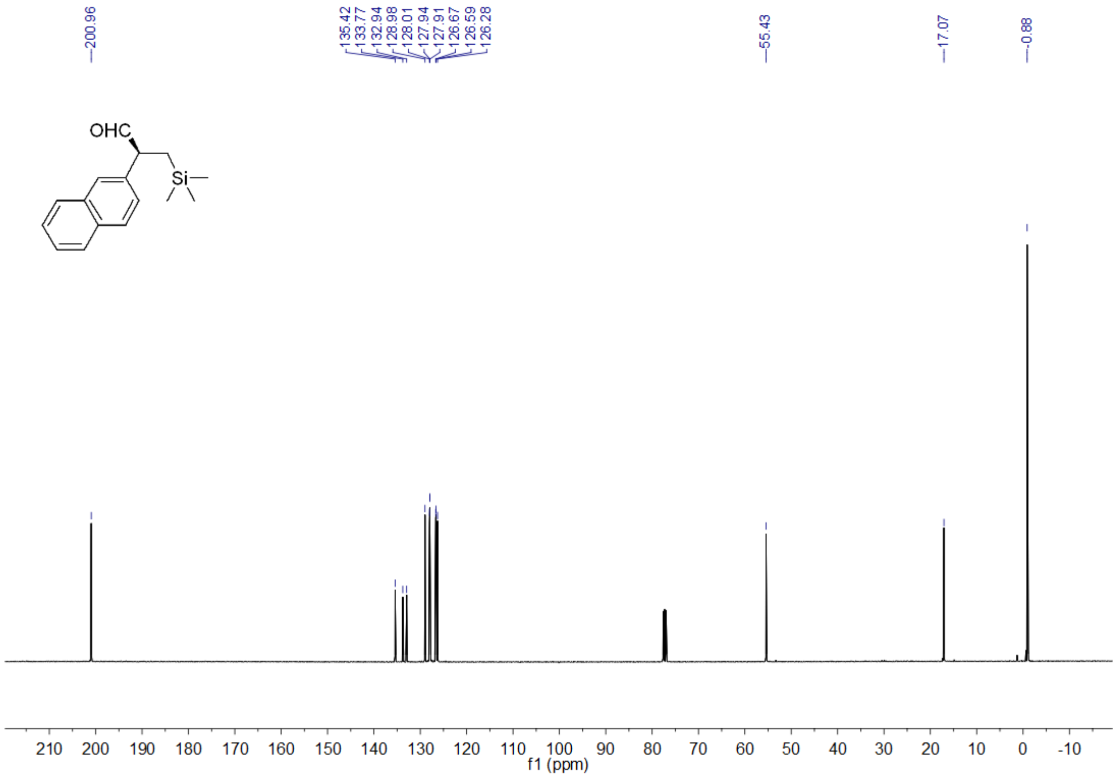

Supplementary Figure 75. ^{13}C **NMR (400 MHz,** $CDCl_3$**) spectra for compound 2o**

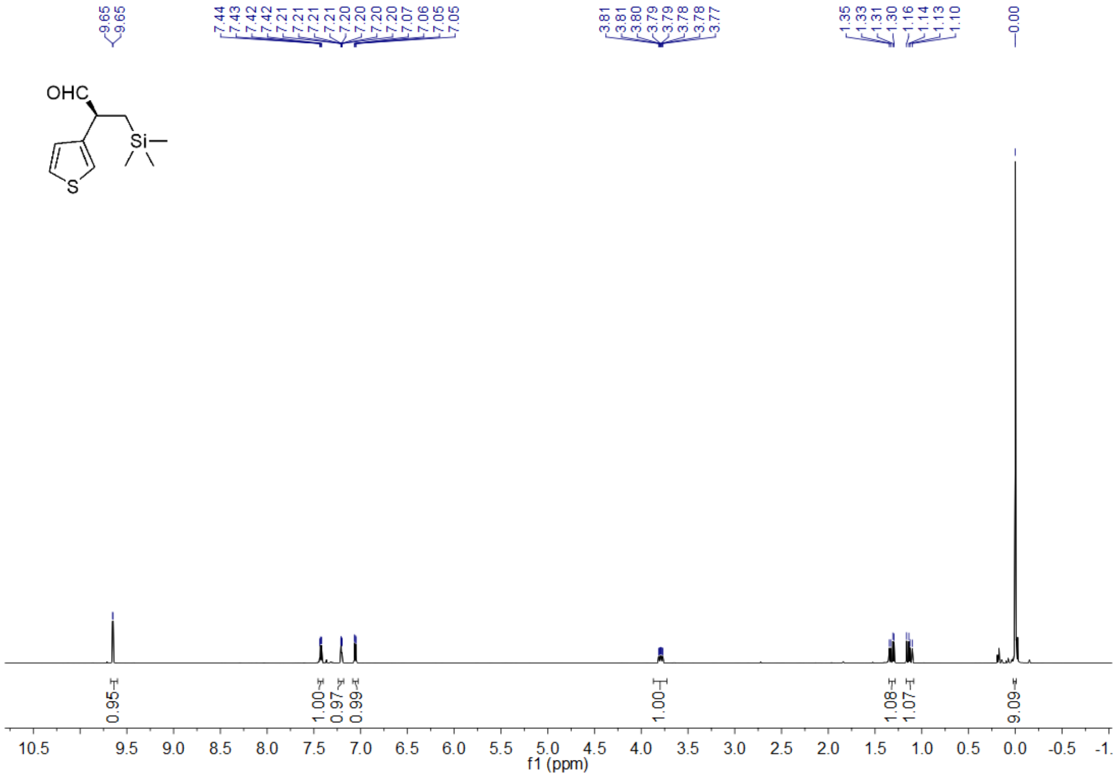

Supplementary Figure 76. ^{1}H NMR (400 MHz, CDCl$_3$) spectra for compound 2q

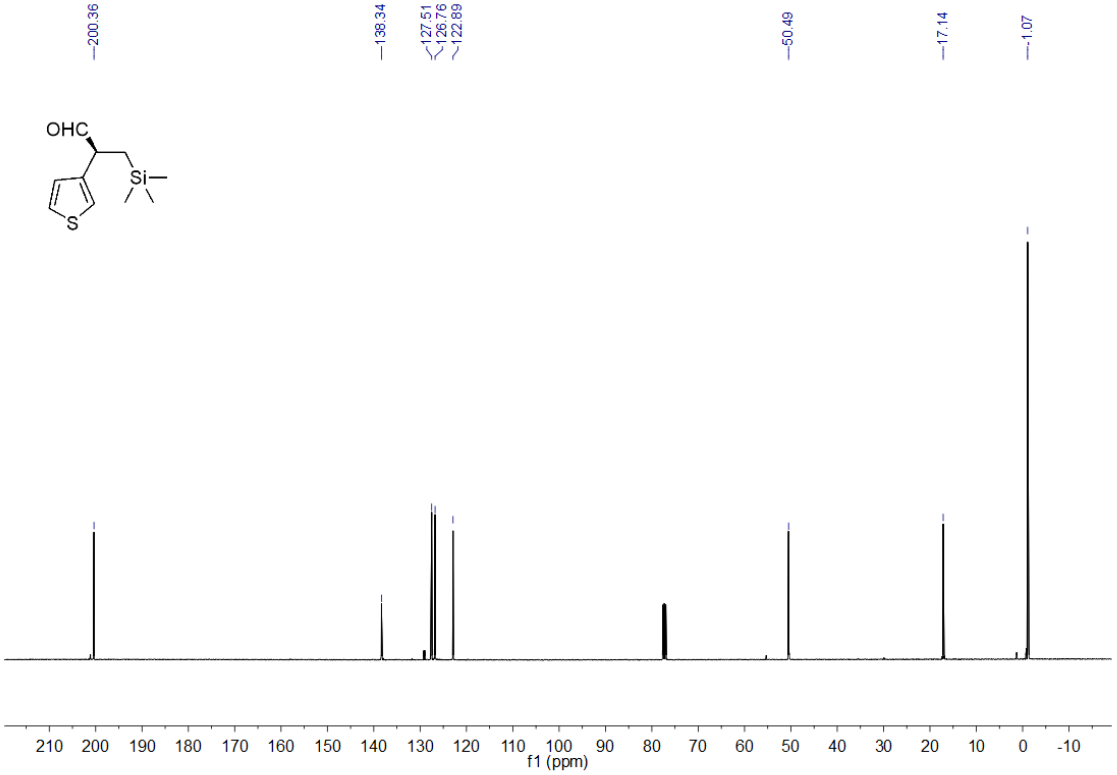

Supplementary Figure 77. ^{13}C NMR (400 MHz, CDCl$_3$) spectra for compound 2q

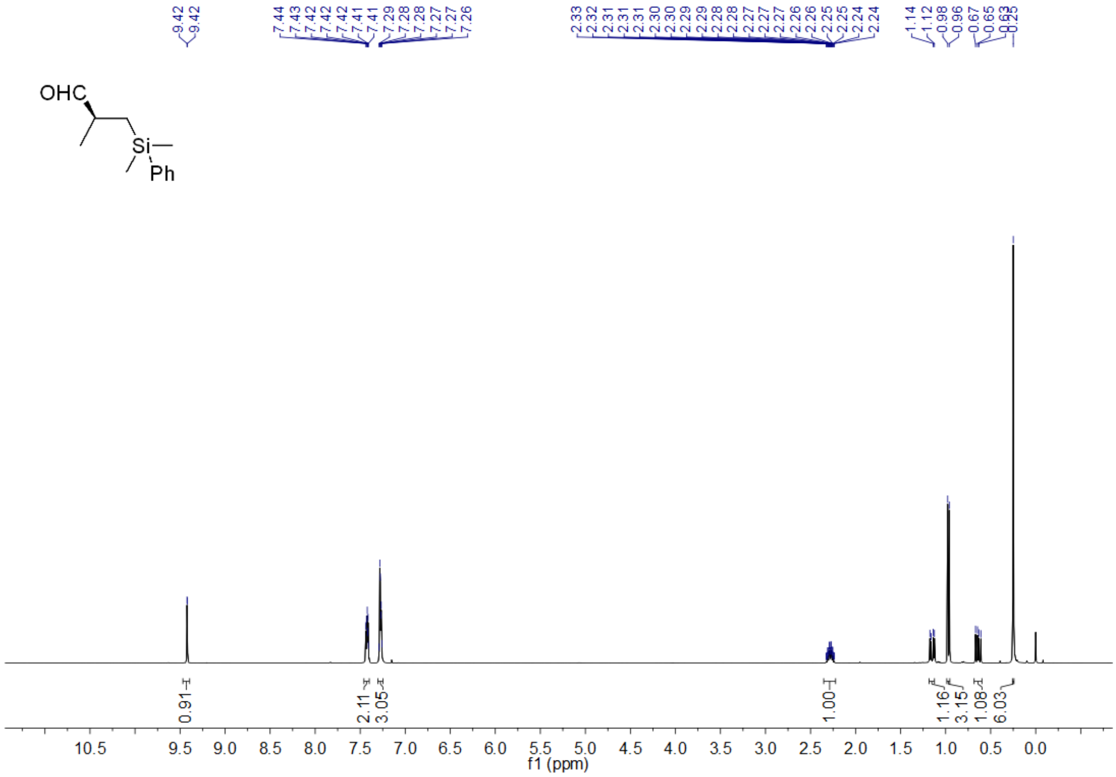

Supplementary Figure 78. ^{1}H **NMR (400 MHz,** $CDCl_3$**) spectra for compound 2r**

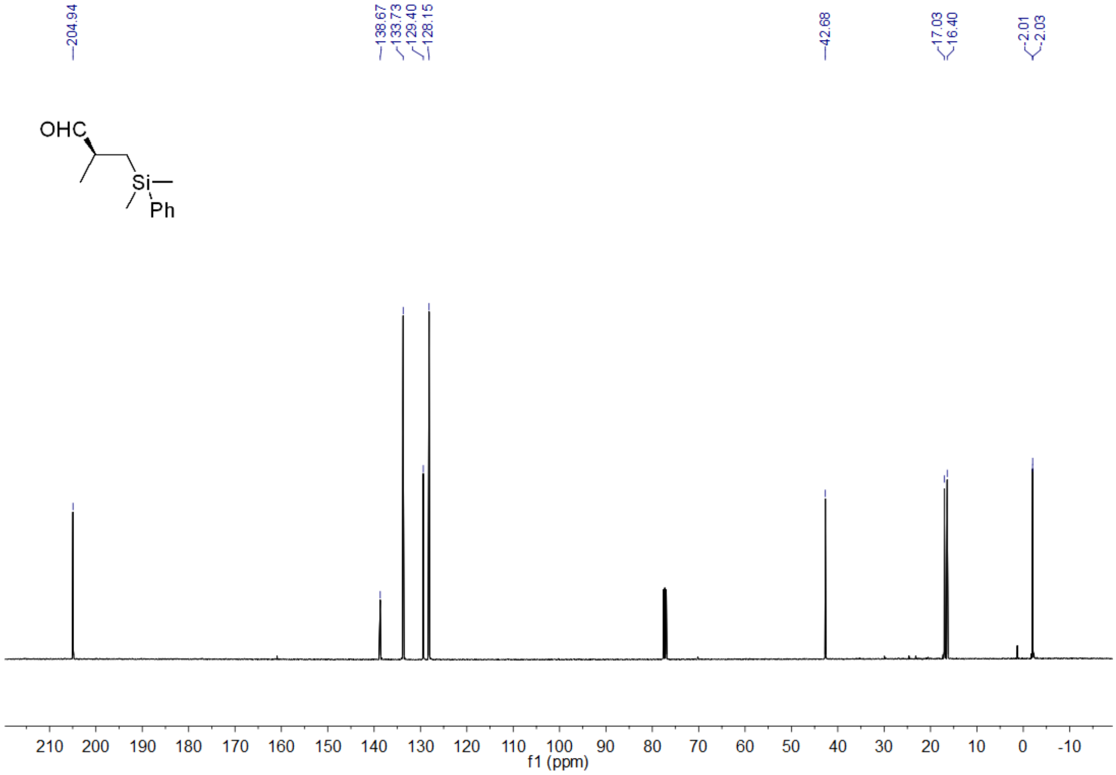

Supplementary Figure 79. ^{13}C **NMR (400 MHz,** $CDCl_3$**) spectra for compound 2r**

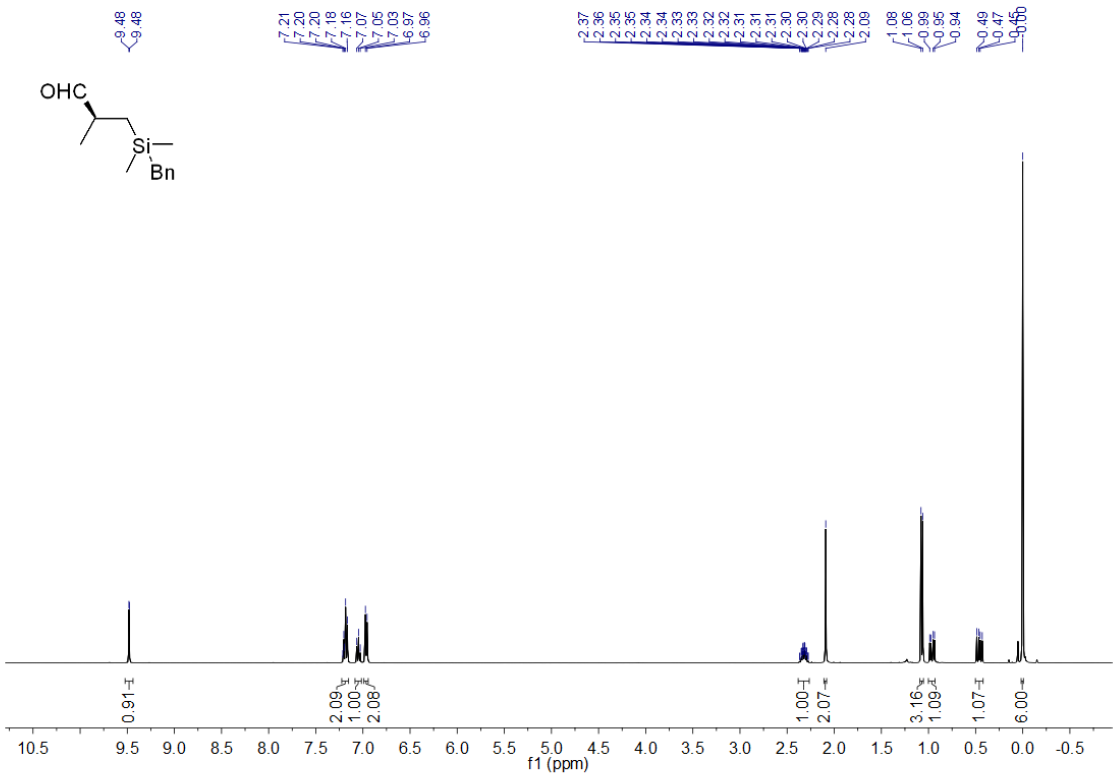

Supplementary Figure 80. ^{1}H NMR (400 MHz, $CDCl_3$) spectra for compound 2s

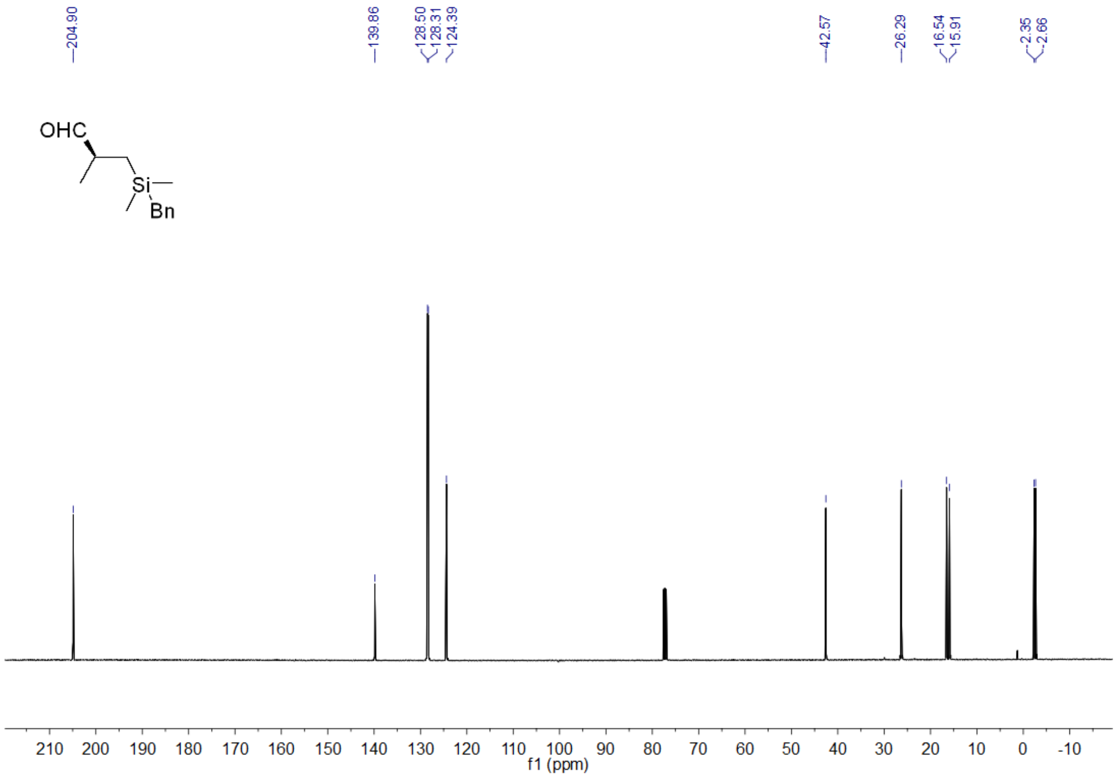

Supplementary Figure 81. ^{13}C NMR (400 MHz, $CDCl_3$) spectra for compound 2s

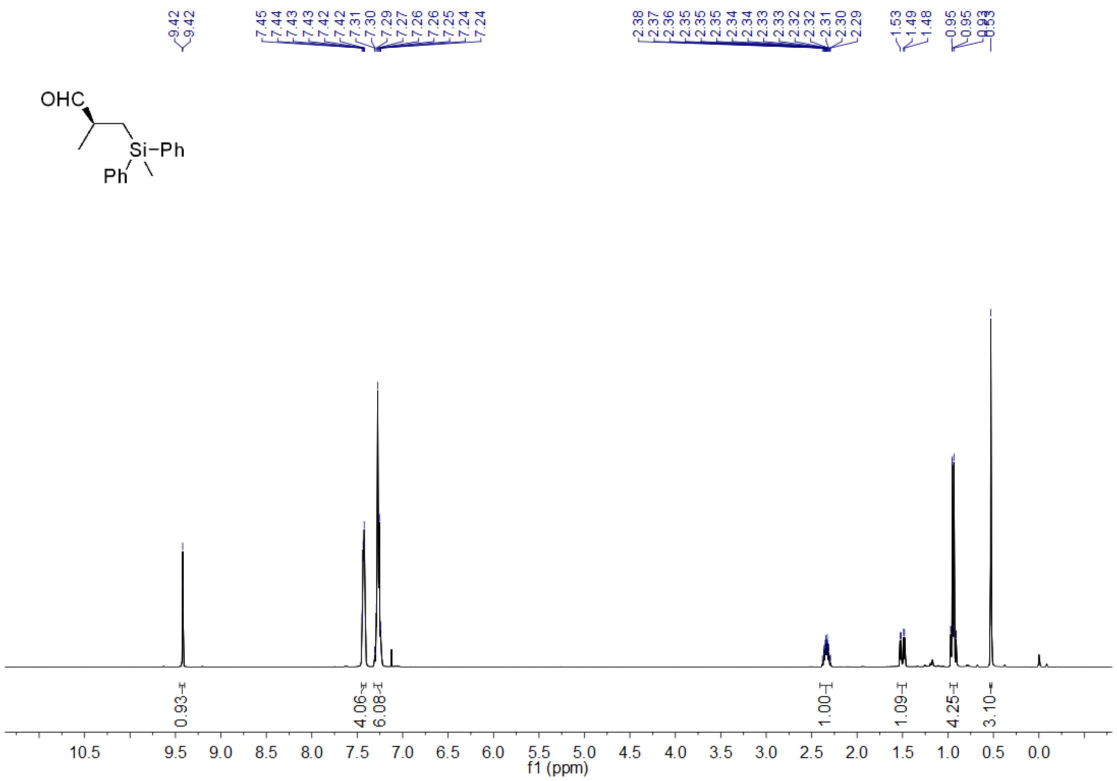

Supplementary Figure 82. ^{1}**H NMR (400 MHz, CDCl$_3$) spectra for compound 2t**

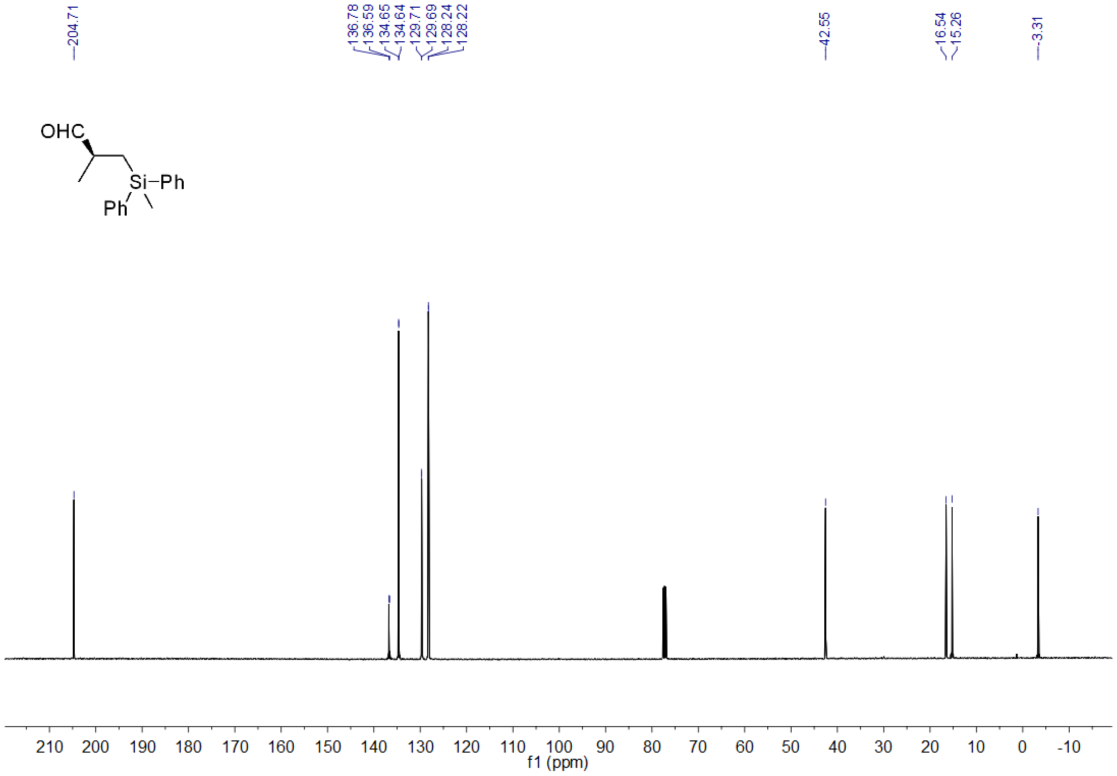

Supplementary Figure 83. ^{13}**C NMR (400 MHz, CDCl$_3$) spectra for compound 2t**

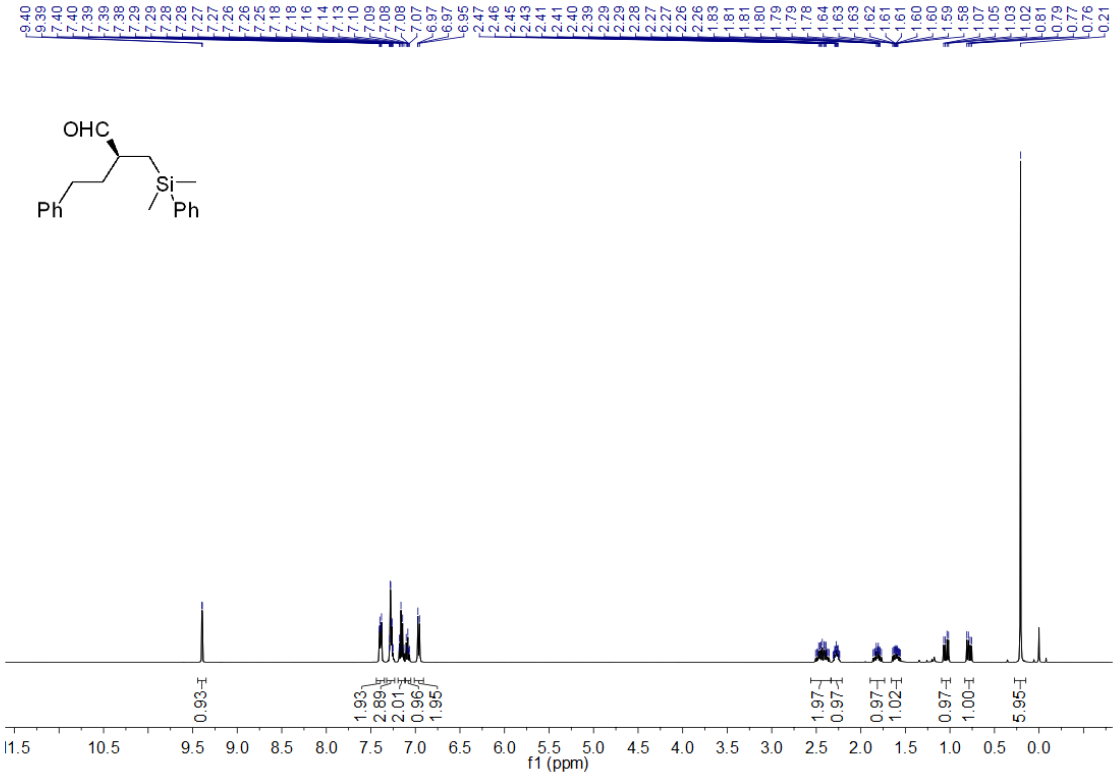

Supplementary Figure 84. ^{1}H NMR (400 MHz, $CDCl_3$) spectra for compound 2u

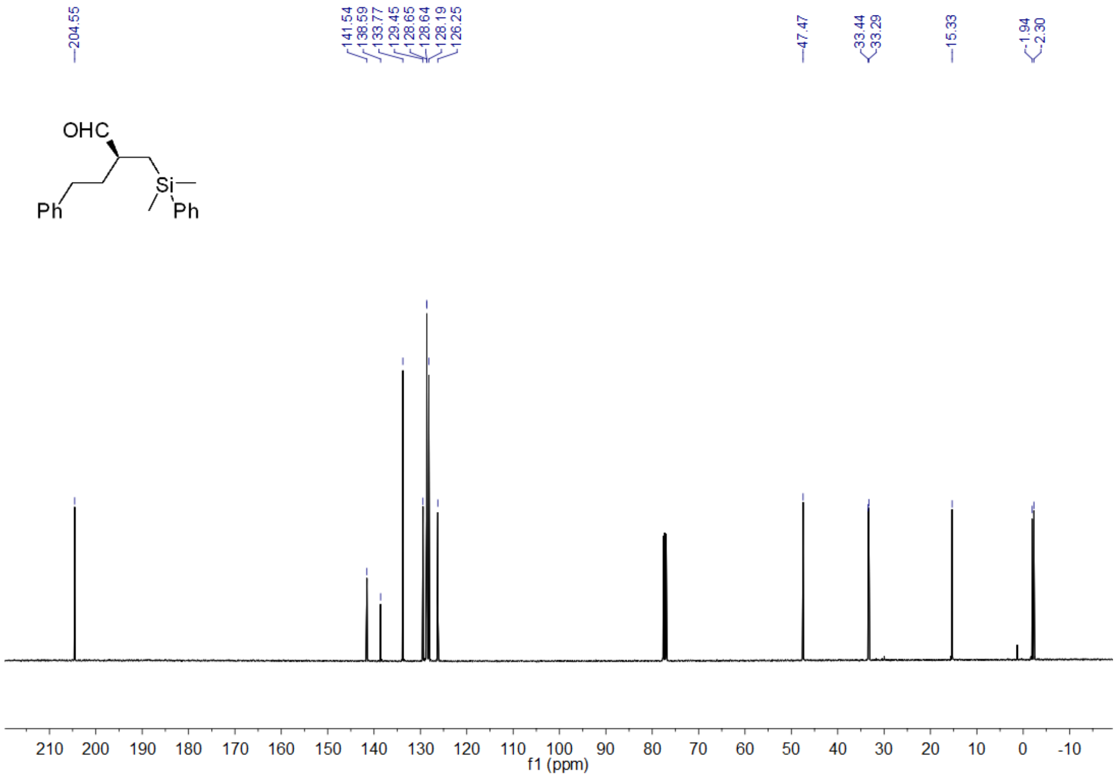

Supplementary Figure 85. ^{13}C NMR (400 MHz, $CDCl_3$) spectra for compound 2u

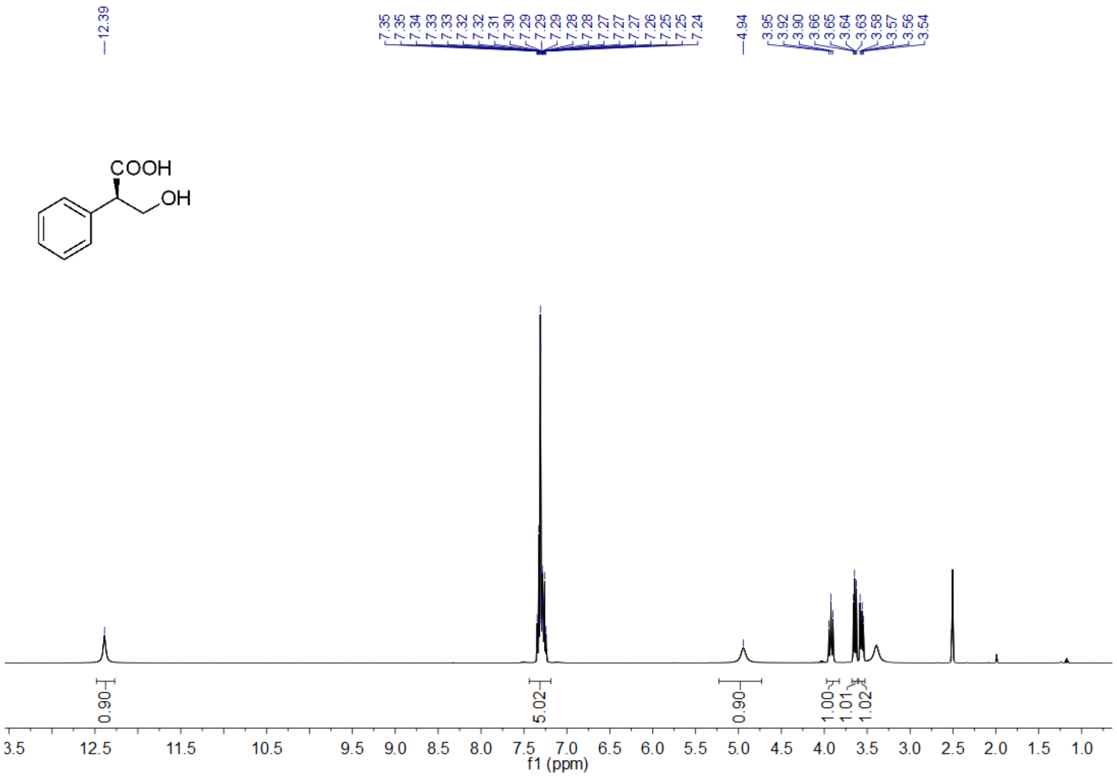

Supplementary Figure 86. **^{1}H NMR (400 MHz, $CDCl_3$) spectra for compound 5**

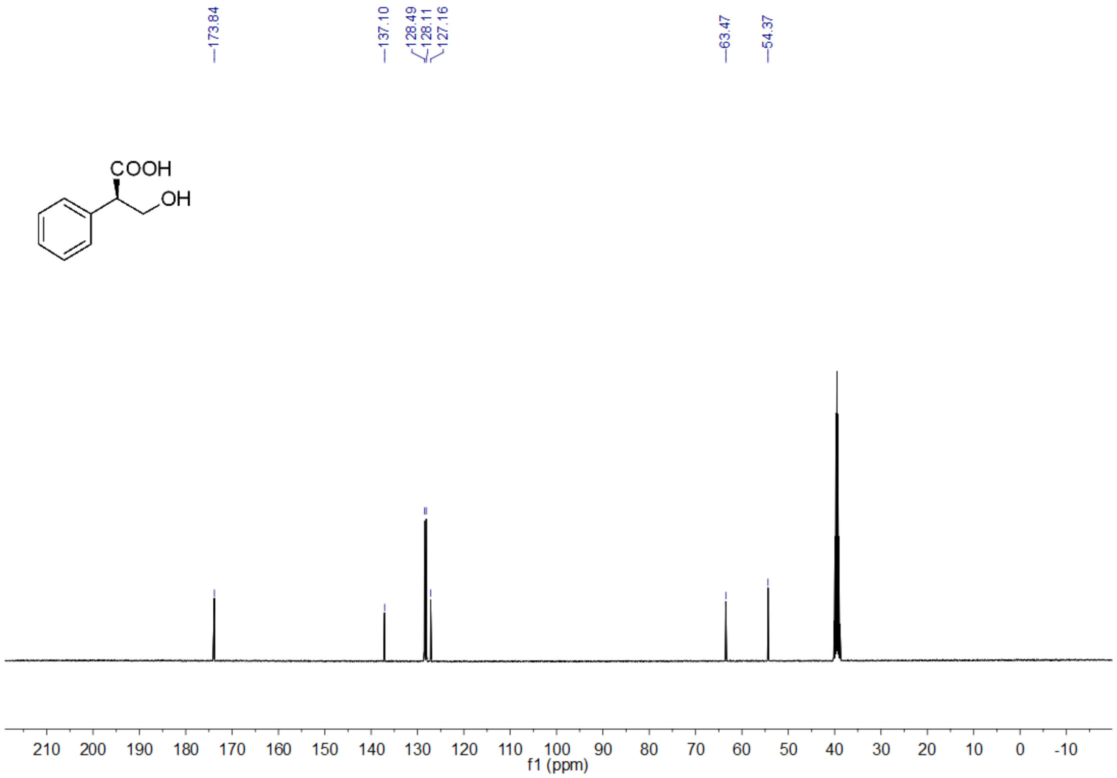

Supplementary Figure 87. **^{13}C NMR (400 MHz, $CDCl_3$) spectra for compound 5**

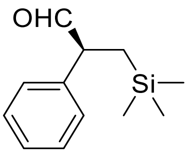

2a

Data File E:\DATA\YC\YC-4-138\YC-4-138 2016-10-17 09-33-49\021-0201.D
Sample Name: YC-4-138-RAC

===

Acq. Operator : SYSTEM Seq. Line : 2
Acq. Instrument : 1260HPLC-DAD Location : Vial 21
Injection Date : 10/17/2016 9:47:55 AM Inj : 1
Inj Volume : 3.000 µl
Acq. Method : E:\DATA\YC\YC-4-138\YC-4-138 2016-10-17 09-33-49\DAD-OD(1-2)-99-1-1ML-3UL-210NM-40MIN.M
Last changed : 10/17/2016 9:33:49 AM by SYSTEM
Analysis Method : E:\DATA\YC\YC-4-138\YC-4-138 2016-10-17 09-33-49\DAD-OD(1-2)-99-1-1ML-3UL-210NM-40MIN.M (Sequence Method)
Last changed : 10/19/2016 9:41:31 AM by SYSTEM
(modified after loading)
Additional Info : Peak(s) manually integrated

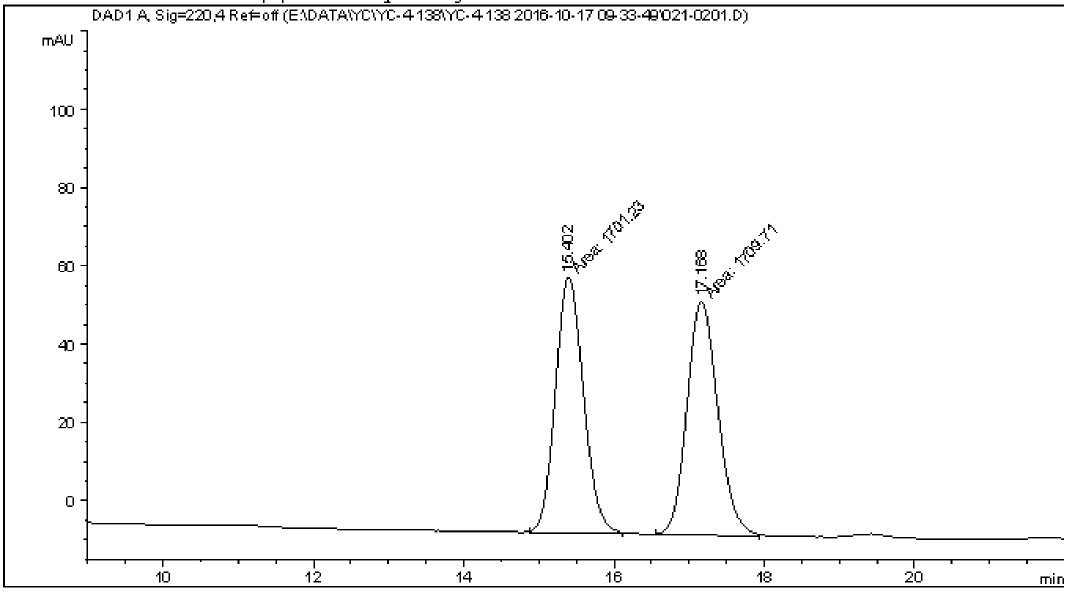

===

Area Percent Report

===

Sorted By : Signal
Multiplier : 1.0000
Dilution : 1.0000
Do not use Multiplier & Dilution Factor with ISTDs

Signal 1: DAD1 A, Sig=220,4 Ref=off

Peak #	RetTime [min]	Type	Width [min]	Area [mAU*s]	Height [mAU]	Area %
1	15.402	MM	0.4336	1701.23035	65.38848	49.8756
2	17.168	MM	0.4777	1709.71448	59.65269	50.1244
Totals :				3410.94482	125.04117	

===

*** End of Report ***

Data File E:\DATA\YC\YC-4-138\YC-4-138 2016-10-17 09-33-49\022-0301.D
Sample Name: YC-4-138

===

Acq. Operator : SYSTEM Seq. Line : 3
Acq. Instrument : 1260HPLC-DAD Location : Vial 22
Injection Date : 10/17/2016 10:28:48 AM Inj : 1
Inj Volume : 3.000 µl
Acq. Method : E:\DATA\YC\YC-4-138\YC-4-138 2016-10-17 09-33-49\DAD-OD(1-2)-99-1-1ML-3UL-210NM-40MIN.M
Last changed : 10/17/2016 9:33:49 AM by SYSTEM
Analysis Method : E:\DATA\YC\YC-4-138\YC-4-138 2016-10-17 09-33-49\DAD-OD(1-2)-99-1-1ML-3UL-210NM-40MIN.M (Sequence Method)
Last changed : 10/19/2016 9:38:52 AM by SYSTEM
(modified after loading)
Additional Info : Peak(s) manually integrated

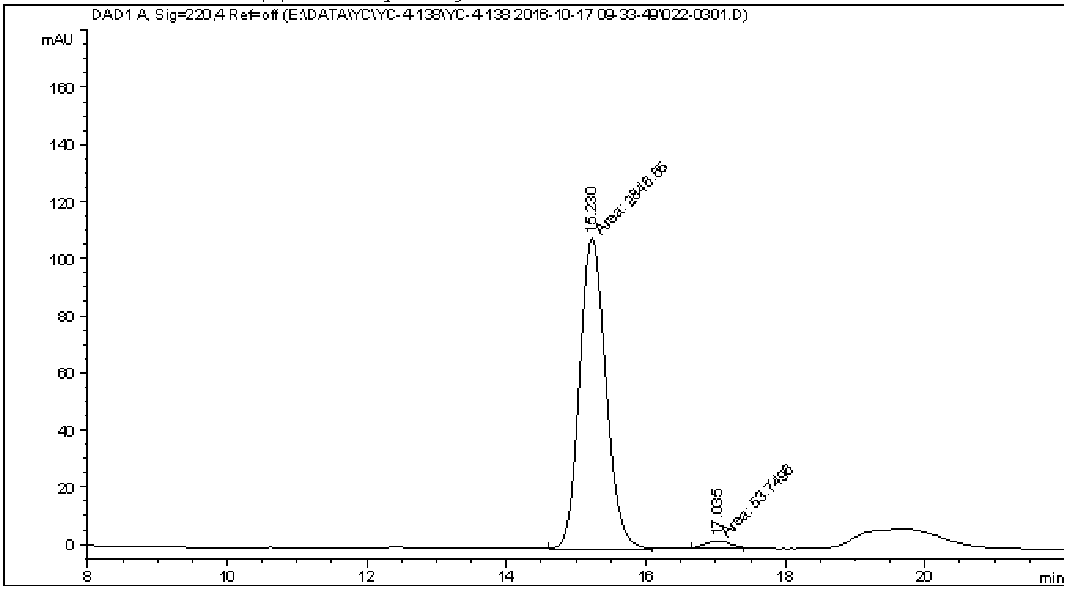

===

Area Percent Report

===

Sorted By : Signal
Multiplier : 1.0000
Dilution : 1.0000
Do not use Multiplier & Dilution Factor with ISTDs

Signal 1: DAD1 A, Sig=220,4 Ref=off

Peak #	RetTime [min]	Type	Width [min]	Area [mAU*s]	Height [mAU]	Area %
1	15.230	MM	0.4365	2846.64551	108.69571	98.1468
2	17.035	MM	0.3947	53.74965	2.26944	1.8532

Totals : 2900.39516 110.96515

===

*** End of Report ***

1260HPLC-DAD 10/19/2016 9:38:54 AM SYSTEM Page 1 of 1

Supplementary Figure 88. HPLC spectra for compound 2a

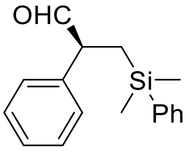

2b

Data File E:\DATA\YC\YC-4-159\YC-4-159 2016-10-23 15-49-17\051-0201.D
Sample Name: yc-4-159-1-rac

```
=====================================================================
Acq. Operator   : SYSTEM                          Seq. Line :   2
Acq. Instrument : 1260HPLC-DAD                     Location : Vial 51
Injection Date  : 10/23/2016 4:01:48 PM                 Inj :   1
                                                 Inj Volume : 3.000 µl
Acq. Method     : E:\DATA\YC\YC-4-159\YC-4-159 2016-10-23 15-49-17\DAD-OD(1-2)-97-3-1ML-
                  3UL-210-230NM-40MIN.M
Last changed    : 10/23/2016 3:49:17 PM by SYSTEM
Analysis Method : E:\DATA\YC\YC-4-159\YC-4-159 2016-10-23 15-49-17\DAD-OD(1-2)-97-3-1ML-
                  3UL-210-230NM-40MIN.M (Sequence Method)
Last changed    : 10/23/2016 6:51:57 PM by SYSTEM
                  (modified after loading)
Additional Info : Peak(s) manually integrated
```

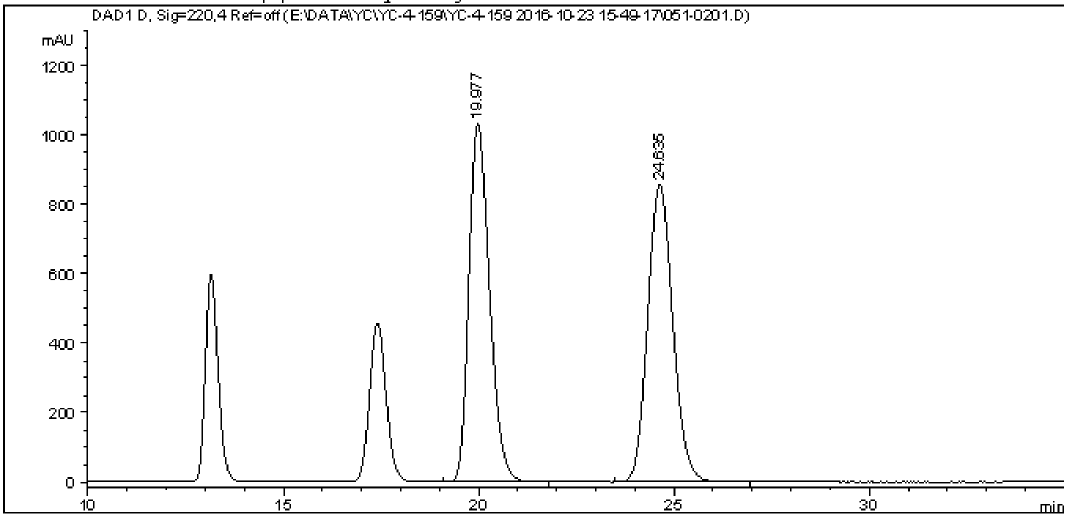


```
=====================================================================
                        Area Percent Report
=====================================================================

Sorted By             :      Signal
Multiplier            :      1.0000
Dilution              :      1.0000
Do not use Multiplier & Dilution Factor with ISTDs


Signal 1: DAD1 D, Sig=220,4 Ref=off

Peak RetTime Type  Width     Area      Height     Area
  #   [min]        [min]   [mAU*s]     [mAU]        %
----|-------|----|-------|----------|----------|--------|
   1  19.977 BB    0.5694 3.79433e4  1030.96973  49.6877
   2  24.635 BB    0.6972 3.84202e4   856.71887  50.3123

Totals :                  7.63635e4  1887.68860


=====================================================================
                         *** End of Report ***
```

Data File E:\DATA\YC\YC-4-159\YC-4-159 2016-10-23 15-49-17\052-0301.D
Sample Name: yc-4-159-1

```
=====================================================================
Acq. Operator   : SYSTEM                        Seq. Line :    3
Acq. Instrument : 1260HPLC-DAD                   Location : Vial 52
Injection Date  : 10/23/2016 4:42:44 PM               Inj :    1
                                               Inj Volume : 3.000 µl
Acq. Method     : E:\DATA\YC\YC-4-159\YC-4-159 2016-10-23 15-49-17\DAD-OD(1-2)-97-3-1ML-
                  3UL-210-230NM-40MIN.M
Last changed    : 10/23/2016 3:49:17 PM by SYSTEM
Analysis Method : E:\DATA\YC\YC-4-159\YC-4-159 2016-10-23 15-49-17\DAD-OD(1-2)-97-3-1ML-
                  3UL-210-230NM-40MIN.M (Sequence Method)
Last changed    : 10/23/2016 6:49:58 PM by SYSTEM
                  (modified after loading)
Additional Info : Peak(s) manually integrated
```

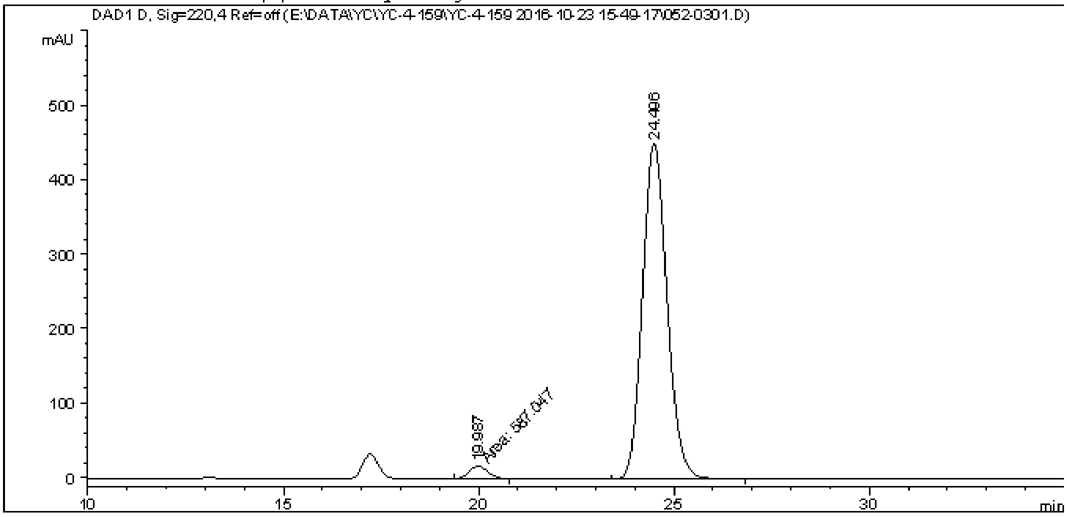


```
=====================================================================
                         Area Percent Report
=====================================================================

Sorted By             :      Signal
Multiplier            :      1.0000
Dilution              :      1.0000
Do not use Multiplier & Dilution Factor with ISTDs


Signal 1: DAD1 D, Sig=220,4 Ref=off

Peak RetTime Type  Width     Area      Height     Area
  #   [min]        [min]   [mAU*s]     [mAU]        %
----|-------|----|-------|----------|----------|--------|
   1  19.987 MM    0.5714  587.04663   17.12288   2.8945
   2  24.496 BB    0.6743 1.96943e4   450.30029  97.1055

Totals :                  2.02813e4   467.42318


=====================================================================
                          *** End of Report ***
```


Supplementary Figure 89. HPLC spectra for compound 2b

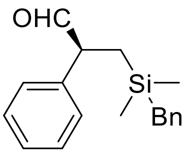

2c

Data File E:\DATA\YC\YC-4-177-OD\YC-4-177-OD 2016-11-18 17-10-52\013-0401.D
Sample Name: YC-4-177-2-RAC

===

Acq. Operator : SYSTEM Seq. Line : 4
Acq. Instrument : 1260HPLC-DAD Location : Vial 13
Injection Date : 11/18/2016 7:24:36 PM Inj : 1
Inj Volume : 5.000 µl
Acq. Method : E:\DATA\YC\YC-4-177-OD\YC-4-177-OD 2016-11-18 17-10-52\DAD-OD-(1-2)-99-1-1ML-5UL-210NM-60MIN.M
Last changed : 11/18/2016 8:01:06 PM by SYSTEM
(modified after loading)
Analysis Method : E:\DATA\YC\YC-4-177-OD\YC-4-177-OD 2016-11-18 17-10-52\DAD-OD-(1-2)-99-1-1ML-5UL-210NM-60MIN.M (Sequence Method)
Last changed : 11/18/2016 10:11:03 PM by SYSTEM
(modified after loading)
Additional Info : Peak(s) manually integrated

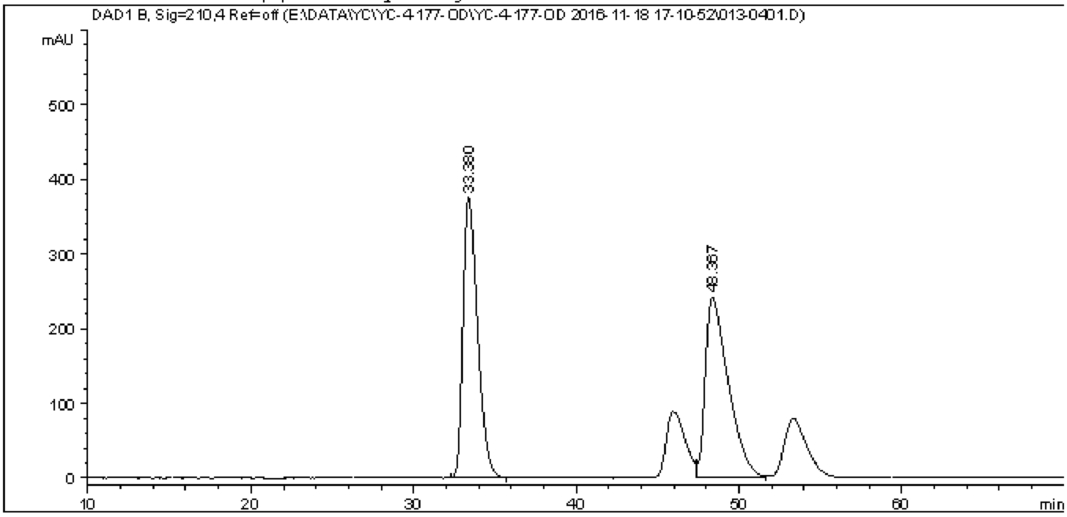

===

Area Percent Report

===

Sorted By : Signal
Multiplier : 1.0000
Dilution : 1.0000
Do not use Multiplier & Dilution Factor with ISTDs

Signal 1: DAD1 B, Sig=210,4 Ref=off

Peak #	RetTime [min]	Type	Width [min]	Area [mAU*s]	Height [mAU]	Area %
1	33.380	BV	0.8187	2.32998e4	375.09927	49.8836
2	48.367	VV	1.1494	2.34086e4	240.53174	50.1164

Totals : 4.67084e4 615.63101

===

*** End of Report ***

1260HPLC-DAD 11/18/2016 10:11:06 PM SYSTEM Page 1 of 1

```
Data File E:\DATA\YC\YC-4-177-OD\YC-4-177-OD 2016-11-18 17-10-52\014-0501.D
Sample Name: YC-4-177-2

=====================================================================
Acq. Operator   : SYSTEM                          Seq. Line :   5
Acq. Instrument : 1260HPLC-DAD                     Location : Vial 14
Injection Date  : 11/18/2016 8:35:30 PM                 Inj :   1
                                                 Inj Volume : 5.000 µl
Acq. Method     : E:\DATA\YC\YC-4-177-OD\YC-4-177-OD 2016-11-18 17-10-52\DAD-OD-(1-2)-99-1
                  -1ML-5UL-210NM-60MIN.M
Last changed    : 11/18/2016 8:01:06 PM by SYSTEM
Analysis Method : E:\DATA\YC\YC-4-177-OD\YC-4-177-OD 2016-11-18 17-10-52\DAD-OD-(1-2)-99-1
                  -1ML-5UL-210NM-60MIN.M (Sequence Method)
Last changed    : 11/18/2016 10:09:36 PM by SYSTEM
                  (modified after loading)
Additional Info : Peak(s) manually integrated
```

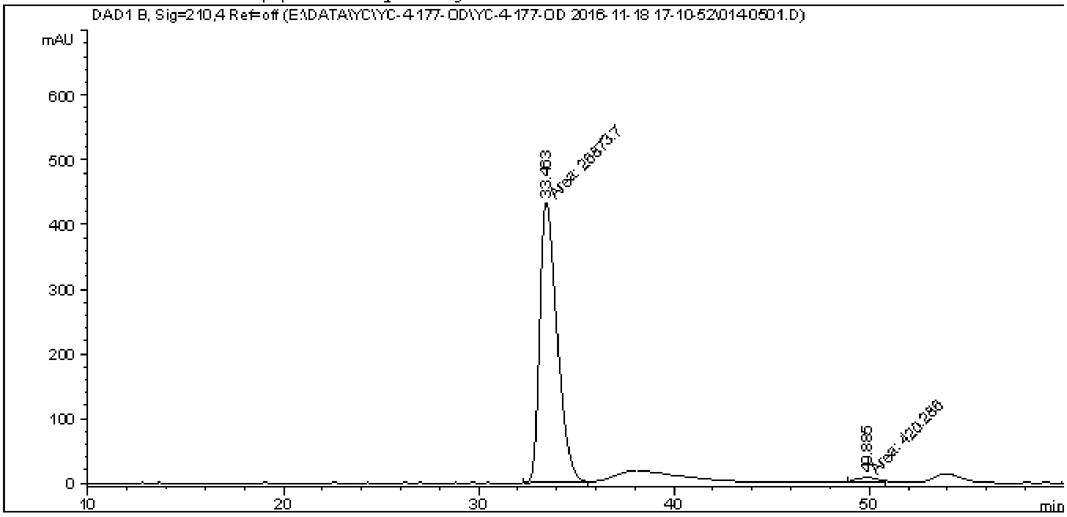


```
=====================================================================
                         Area Percent Report
=====================================================================

Sorted By             :      Signal
Multiplier            :      1.0000
Dilution              :      1.0000
Do not use Multiplier & Dilution Factor with ISTDs


Signal 1: DAD1 B, Sig=210,4 Ref=off

Peak RetTime Type  Width     Area      Height     Area
  #   [min]        [min]   [mAU*s]     [mAU]        %
----|-------|----|-------|----------|----------|--------|
   1  33.463 MM    1.0335 2.68737e4  433.35953  98.4602
   2  49.885 MM    1.1174  420.28586    6.26877   1.5398

Totals :                  2.72940e4  439.62830


=====================================================================
                          *** End of Report ***
```

1260HPLC-DAD 11/18/2016 10:09:39 PM SYSTEM   Page 1 of 1

Supplementary Figure 90. HPLC spectra for compound 2c

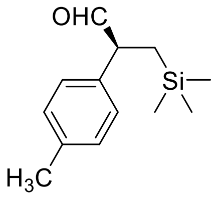

2d

Data File E:\DATA\YC\YC-4-163\YC-4-163 2016-10-26 13-30-13\051-0301.D
Sample Name: YC-4-165-1-RAC

===

Acq. Operator : SYSTEM Seq. Line : 3
Acq. Instrument : 1260HPLC-DAD Location : Vial 51
Injection Date : 10/26/2016 3:23:04 PM Inj : 1
Inj Volume : 3.000 µl
Acq. Method : E:\DATA\YC\YC-4-163\YC-4-163 2016-10-26 13-30-13\DAD-OD(1-2)-99-1-1ML-3UL-210NM-40MIN.M
Last changed : 10/26/2016 1:30:14 PM by SYSTEM
Analysis Method : E:\DATA\YC\YC-4-163\YC-4-163 2016-10-26 13-30-13\DAD-OD(1-2)-99-1-1ML-3UL-210NM-40MIN.M (Sequence Method)
Last changed : 10/26/2016 4:49:45 PM by SYSTEM
(modified after loading)
Additional Info : Peak(s) manually integrated

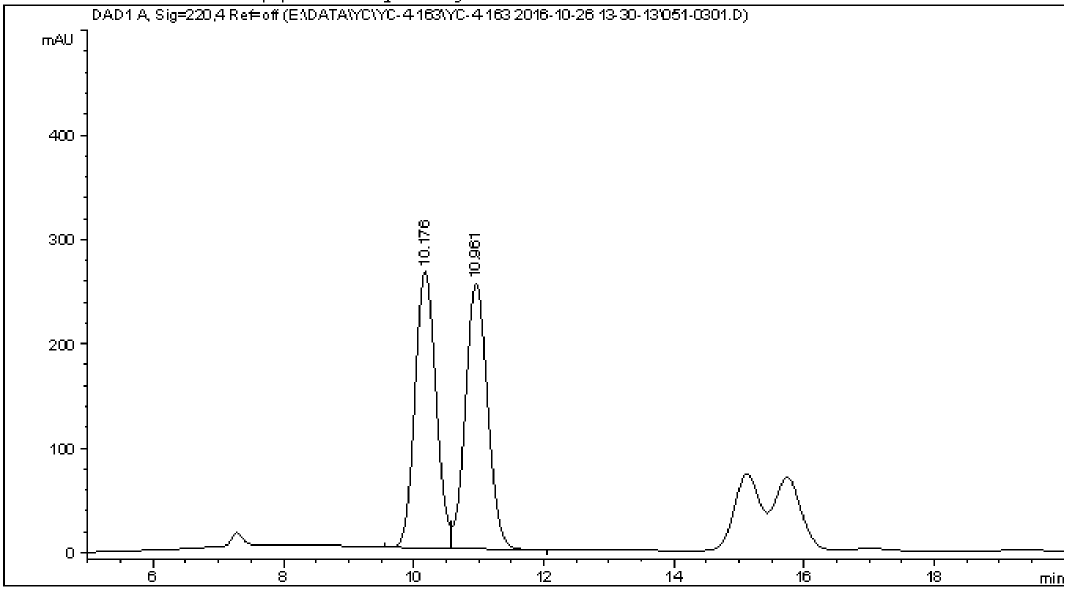

===

Area Percent Report

===

Sorted By : Signal
Multiplier : 1.0000
Dilution : 1.0000
Do not use Multiplier & Dilution Factor with ISTDs

Signal 1: DAD1 A, Sig=220,4 Ref=off

Peak #	RetTime [min]	Type	Width [min]	Area [mAU*s]	Height [mAU]	Area %
1	10.176	BV	0.3519	5944.74951	264.81735	49.7021
2	10.961	VB	0.3702	6016.01855	254.08917	50.2979

Totals : 1.19608e4 518.90652

===

*** End of Report ***

1260HPLC-DAD 10/26/2016 4:49:50 PM SYSTEM Page 1 of 1

```
Data File E:\DATA\YC\YC-4-163\YC-4-163 2016-10-26 13-30-13\052-0401.D
Sample Name: YC-4-165-1

    =====================================================================
    Acq. Operator   : SYSTEM                          Seq. Line :   4
    Acq. Instrument : 1260HPLC-DAD                     Location : Vial 52
    Injection Date  : 10/26/2016 4:03:59 PM                 Inj :   1
                                                     Inj Volume : 3.000 µl
    Acq. Method     : E:\DATA\YC\YC-4-163\YC-4-163 2016-10-26 13-30-13\DAD-OD(1-2)-99-1-1ML-
                      3UL-210NM-40MIN.M
    Last changed    : 10/26/2016 4:40:07 PM by SYSTEM
                      (modified after loading)
    Analysis Method : E:\DATA\YC\YC-4-163\YC-4-163 2016-10-26 13-30-13\DAD-OD(1-2)-99-1-1ML-
                      3UL-210NM-40MIN.M (Sequence Method)
    Last changed    : 10/26/2016 4:46:17 PM by SYSTEM
                      (modified after loading)
    Additional Info : Peak(s) manually integrated
```

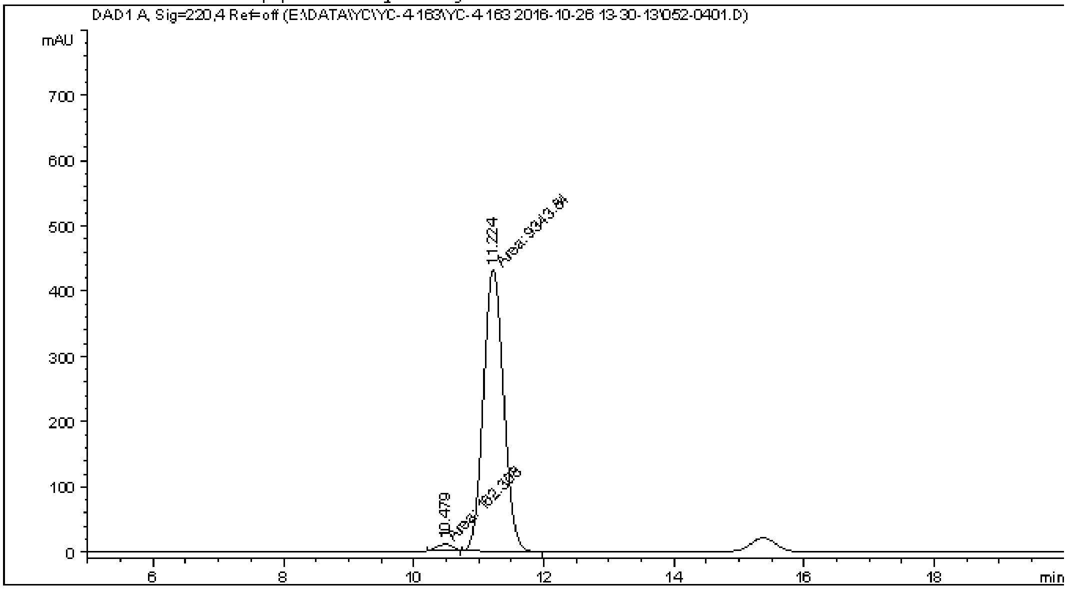


```
    =====================================================================
                             Area Percent Report
    =====================================================================

    Sorted By             :      Signal
    Multiplier            :      1.0000
    Dilution              :      1.0000
    Do not use Multiplier & Dilution Factor with ISTDs


    Signal 1: DAD1 A, Sig=220,4 Ref=off

    Peak RetTime Type  Width     Area      Height     Area
      #   [min]        [min]   [mAU*s]     [mAU]        %
    ----|-------|----|-------|----------|----------|--------|
       1  10.479 MM    0.2752  162.30769    9.82856   1.7074
       2  11.224 MM    0.3618 9343.84473  430.43066  98.2926

    Totals :                  9506.15242  440.25923


    =====================================================================
```


Supplementary Figure 91. HPLC spectra for compound 2d

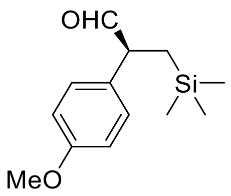

2e

Data File E:\DATA\YC\YC-4-170\YC-4-170 2016-11-05 12-07-02\013-0501.D
Sample Name: YC-4-170-2-RAC

===

Acq. Operator : SYSTEM Seq. Line : 5
Acq. Instrument : 1260HPLC-VWD Location : Vial 13
Injection Date : 11/5/2016 2:08:57 PM Inj : 1
Inj Volume : 3.000 µl
Acq. Method : E:\DATA\YC\YC-4-170\YC-4-170 2016-11-05 12-07-02\VWD-AD(1-6)-97-3-0.8-3UL-210NM-40MIN.M
Last changed : 11/5/2016 12:30:41 PM by SYSTEM
Analysis Method : E:\DATA\YC\YC-4-170\YC-4-170 2016-11-05 12-07-02\VWD-AD(1-6)-97-3-0.8-3UL-210NM-40MIN.M (Sequence Method)
Last changed : 11/5/2016 3:05:20 PM by SYSTEM
(modified after loading)
Additional Info : Peak(s) manually integrated

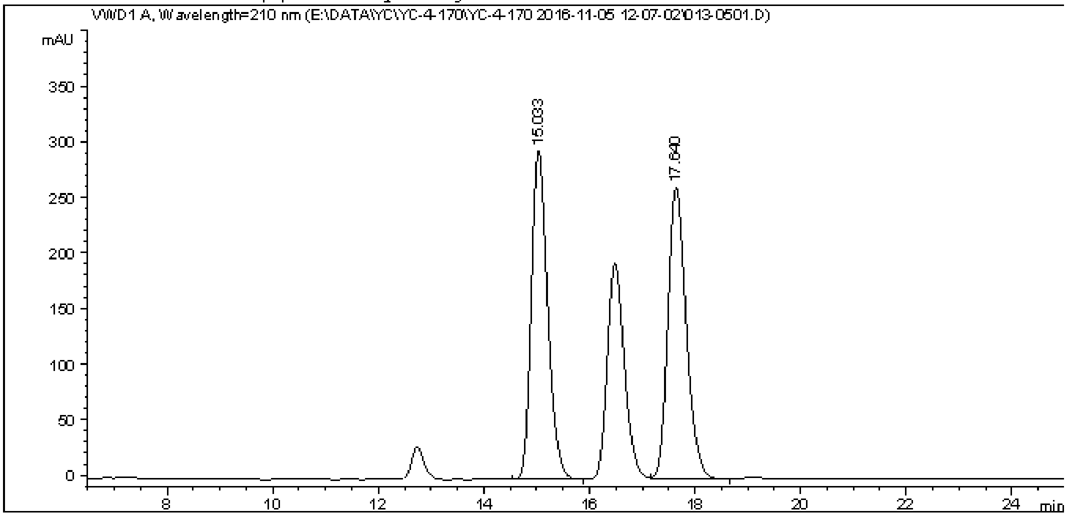

===

Area Percent Report

===

Sorted By : Signal
Multiplier : 1.0000
Dilution : 1.0000
Do not use Multiplier & Dilution Factor with ISTDs

Signal 1: VWD1 A, Wavelength=210 nm

Peak #	RetTime [min]	Type	Width [min]	Area [mAU*s]	Height [mAU]	Area %
1	15.033	BB	0.3298	6346.47705	296.37338	49.8679
2	17.640	VB	0.3719	6380.08936	263.15720	50.1321

Totals : 1.27266e4 559.53058

===

*** End of Report ***

Data File E:\DATA\YC\YC-4-170\YC-4-170 2016-11-05 12-07-02\014-0601.D
Sample Name: YC-4-170-2

===

Acq. Operator : SYSTEM Seq. Line : 6
Acq. Instrument : 1260HPLC-VWD Location : Vial 14
Injection Date : 11/5/2016 2:49:41 PM Inj : 1
Inj Volume : 3.000 µl
Acq. Method : E:\DATA\YC\YC-4-170\YC-4-170 2016-11-05 12-07-02\VWD-AD(1-6)-97-3-0.8-3UL-210NM-40MIN.M
Last changed : 11/5/2016 3:10:05 PM by SYSTEM
(modified after loading)
Analysis Method : E:\DATA\YC\YC-4-170\YC-4-170 2016-11-05 12-07-02\VWD-AD(1-6)-97-3-0.8-3UL-210NM-40MIN.M (Sequence Method)
Last changed : 11/5/2016 3:26:05 PM by SYSTEM
(modified after loading)
Additional Info : Peak(s) manually integrated

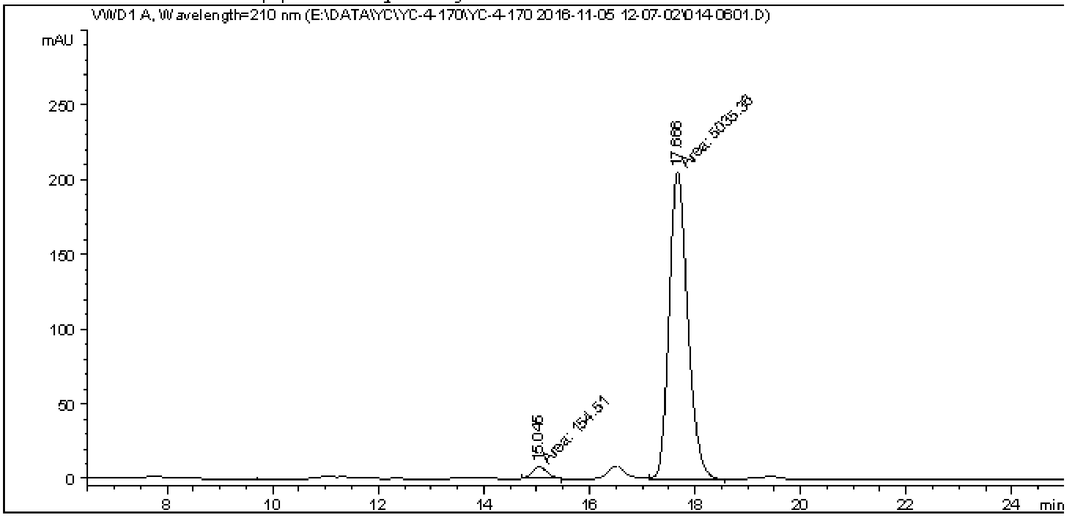

===

Area Percent Report

===

Sorted By : Signal
Multiplier : 1.0000
Dilution : 1.0000
Do not use Multiplier & Dilution Factor with ISTDs

Signal 1: VWD1 A, Wavelength=210 nm

Peak #	RetTime [min]	Type	Width [min]	Area [mAU*s]	Height [mAU]	Area %
1	15.045	MM	0.3331	154.51033	7.73085	2.9772
2	17.666	MM	0.4082	5035.36084	205.57587	97.0228

Totals : 5189.87117 213.30672

===

*** End of Report ***

1260HPLC-VWD 11/5/2016 3:26:11 PM SYSTEM Page 1 of 1

Supplementary Figure 92. HPLC spectra for compound 2e

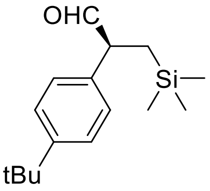

2f

Data File E:\DATA\YC\YC-4-163\YC-4-163 2016-10-26 16-53-19\053-0401.D
Sample Name: YC-4-165-3-RAC-1

```
=====================================================================
Acq. Operator   : SYSTEM                          Seq. Line :   4
Acq. Instrument : 1260HPLC-DAD                     Location : Vial 53
Injection Date  : 10/26/2016 6:17:08 PM                 Inj :   1
                                                 Inj Volume : 3.000 µl
Acq. Method     : E:\DATA\YC\YC-4-163\YC-4-163 2016-10-26 16-53-19\DAD-OD(1-2)-99-1-0.5ML-
                  3UL-210NM-40MIN.M
Last changed    : 10/26/2016 5:50:48 PM by SYSTEM
Analysis Method : E:\DATA\YC\YC-4-163\YC-4-163 2016-10-26 16-53-19\DAD-OD(1-2)-99-1-0.5ML-
                  3UL-210NM-40MIN.M (Sequence Method)
Last changed    : 10/26/2016 8:53:11 PM by SYSTEM
                  (modified after loading)
Additional Info : Peak(s) manually integrated
```

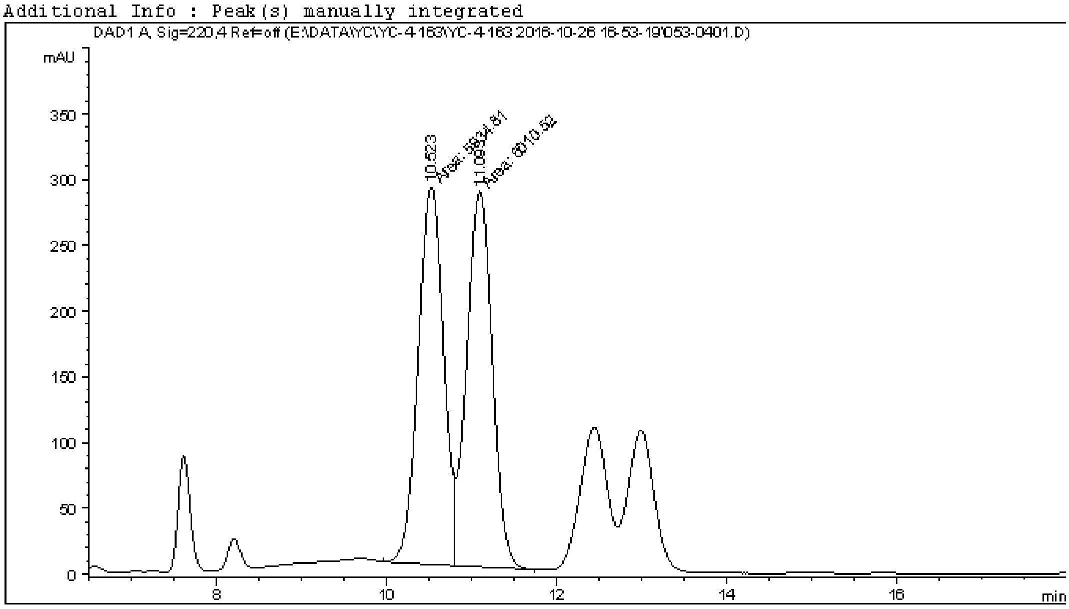


```
=====================================================================
                        Area Percent Report
=====================================================================

Sorted By             :      Signal
Multiplier            :      1.0000
Dilution              :      1.0000
Do not use Multiplier & Dilution Factor with ISTDs


Signal 1: DAD1 A, Sig=220,4 Ref=off

Peak RetTime Type  Width     Area      Height     Area
  #   [min]        [min]   [mAU*s]     [mAU]        %
----|-------|----|-------|----------|----------|--------|
   1  10.523 MF    0.3447 5934.81396  286.96530  49.6831
   2  11.093 FM    0.3511 6010.52441  285.32025  50.3169

Totals :                  1.19453e4   572.28555

=====================================================================
                         *** End of Report ***
```

Data File E:\DATA\YC\YC-4-163\YC-4-163 2016-10-26 16-53-19\054-0501.D
Sample Name: YC-4-165-3

```
=====================================================================
Acq. Operator   : SYSTEM                        Seq. Line :   5
Acq. Instrument : 1260HPLC-DAD                   Location : Vial 54
Injection Date  : 10/26/2016 6:58:02 PM               Inj :   1
                                               Inj Volume : 3.000 µl
Acq. Method     : E:\DATA\YC\YC-4-163\YC-4-163 2016-10-26 16-53-19\DAD-OD(1-2)-99-1-0.5ML-
                  3UL-210NM-40MIN.M
Last changed    : 10/26/2016 5:50:48 PM by SYSTEM
Analysis Method : E:\DATA\YC\YC-4-163\YC-4-163 2016-10-26 16-53-19\DAD-OD(1-2)-99-1-0.5ML-
                  3UL-210NM-40MIN.M (Sequence Method)
Last changed    : 10/26/2016 8:48:56 PM by SYSTEM
                  (modified after loading)
Additional Info : Peak(s) manually integrated
```

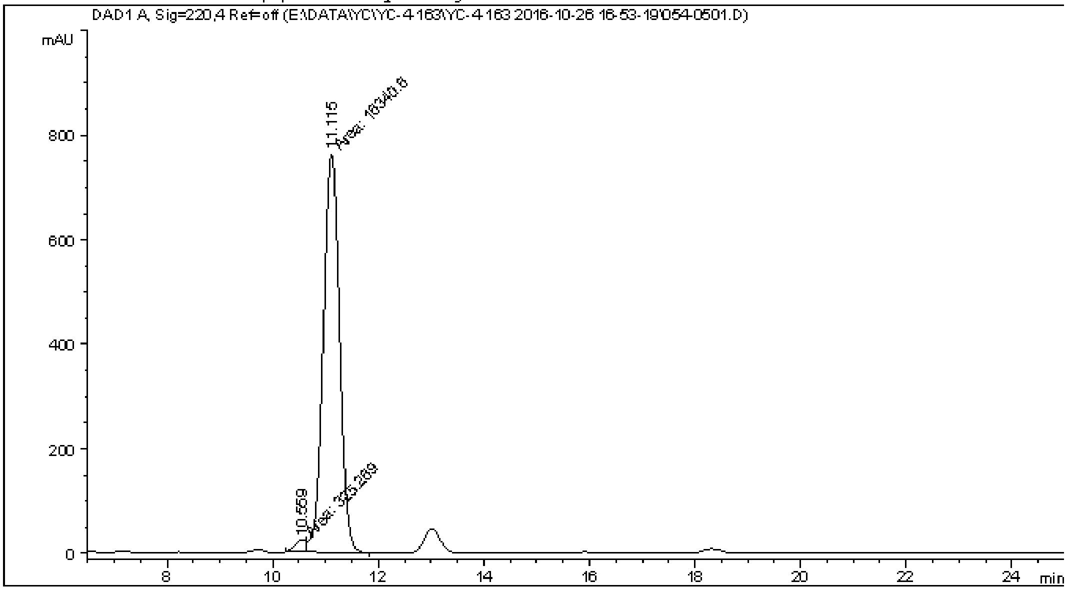


```
=====================================================================
                         Area Percent Report
=====================================================================

Sorted By             :      Signal
Multiplier            :      1.0000
Dilution              :      1.0000
Do not use Multiplier & Dilution Factor with ISTDs


Signal 1: DAD1 A, Sig=220,4 Ref=off

Peak RetTime Type  Width     Area      Height     Area
  #   [min]        [min]   [mAU*s]     [mAU]        %
----|-------|----|-------|----------|----------|--------|
   1  10.559 MF    0.2523  325.26932   21.48429   1.9517
   2  11.115 FM    0.3576 1.63406e4   761.61133  98.0483

Totals :                  1.66659e4   783.09562

=====================================================================
                          *** End of Report ***
```

1260HPLC-DAD 10/26/2016 8:49:30 PM SYSTEM Page 1 of 1

Supplementary Figure 93. HPLC spectra for compound 2f

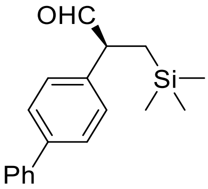

2g

Data File E:\DATA\YHL\YHL-5-204\YHL-5-204 2016-10-23 15-17-32\096-0901.D
Sample Name: YC-4-159-4-RAC

===

Acq. Operator : SYSTEM Seq. Line : 9
Acq. Instrument : 1260HPLC-VWD Location : Vial 96
Injection Date : 10/23/2016 7:29:20 PM Inj : 1
Inj Volume : 3.000 µl
Acq. Method : E:\DATA\YHL\YHL-5-204\YHL-5-204 2016-10-23 15-17-32\VWD-AD(1-6)-97-3-0.8-3UL-210NM-40MIN.M
Last changed : 10/23/2016 3:51:05 PM by SYSTEM
Analysis Method : E:\DATA\YHL\YHL-5-204\YHL-5-204 2016-10-23 15-17-32\VWD-AD(1-6)-97-3-0.8-3UL-210NM-40MIN.M (Sequence Method)
Last changed : 10/23/2016 8:45:45 PM by SYSTEM
(modified after loading)
Additional Info : Peak(s) manually integrated

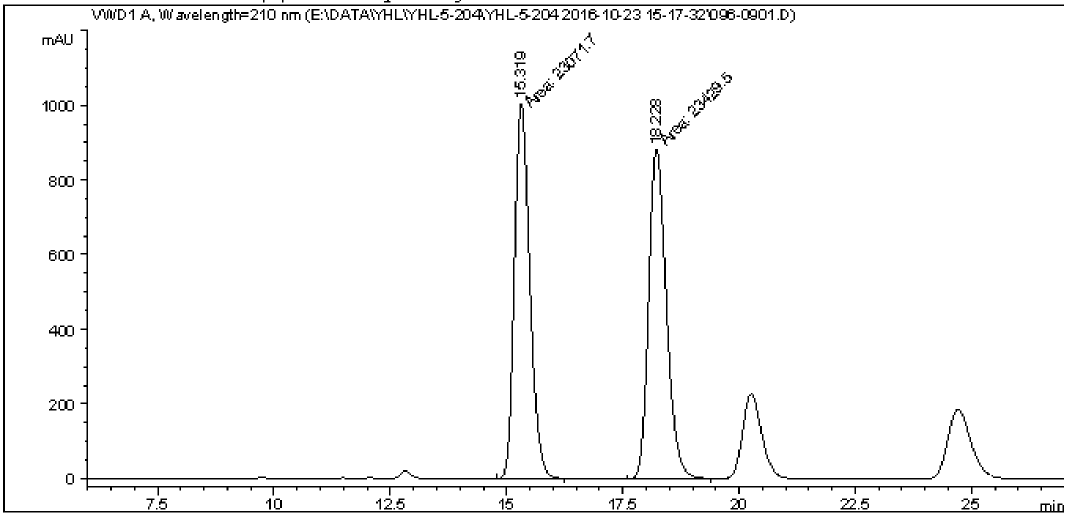

===

Area Percent Report

===

Sorted By : Signal
Multiplier : 1.0000
Dilution : 1.0000
Do not use Multiplier & Dilution Factor with ISTDs

Signal 1: VWD1 A, Wavelength=210 nm

Peak #	RetTime [min]	Type	Width [min]	Area [mAU*s]	Height [mAU]	Area %
1	15.319	MM	0.3829	2.30717e4	1004.28241	49.6153
2	18.228	MM	0.4427	2.34295e4	882.05743	50.3847
Totals :				4.65012e4	1886.33984	

===

*** End of Report ***

```
Data File E:\DATA\YHL\YHL-5-204\YHL-5-204 2016-10-23 15-17-32\097-1001.D
Sample Name: YC-4-159-4

    =====================================================================
    Acq. Operator   : SYSTEM                         Seq. Line :  10
    Acq. Instrument : 1260HPLC-VWD                    Location : Vial 97
    Injection Date  : 10/23/2016 8:10:05 PM                Inj :   1
                                                    Inj Volume : 3.000 µl
    Acq. Method     : E:\DATA\YHL\YHL-5-204\YHL-5-204 2016-10-23 15-17-32\VWD-AD(1-6)-97-3-0.8
                      -3UL-210NM-40MIN.M
    Last changed    : 10/23/2016 8:46:35 PM by SYSTEM
                      (modified after loading)
    Analysis Method : E:\DATA\YHL\YHL-5-204\YHL-5-204 2016-10-23 15-17-32\VWD-AD(1-6)-97-3-0.8
                      -3UL-210NM-40MIN.M (Sequence Method)
    Last changed    : 10/23/2016 8:48:53 PM by SYSTEM
                      (modified after loading)
    Additional Info : Peak(s) manually integrated
```

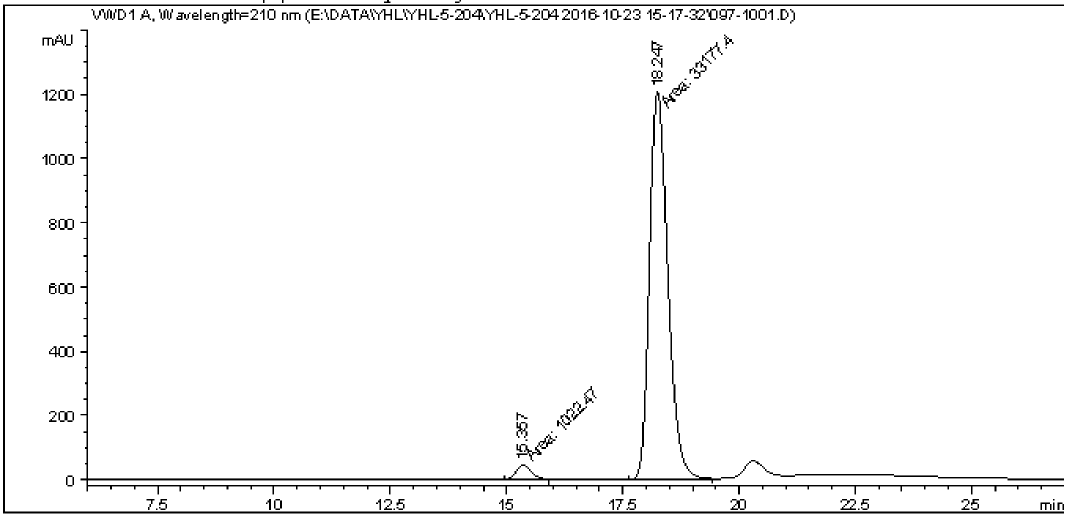


```
    =====================================================================
                             Area Percent Report
    =====================================================================

    Sorted By             :      Signal
    Multiplier            :      1.0000
    Dilution              :      1.0000
    Do not use Multiplier & Dilution Factor with ISTDs


    Signal 1: VWD1 A, Wavelength=210 nm

    Peak RetTime Type  Width     Area      Height     Area
      #   [min]        [min]   [mAU*s]     [mAU]        %
    ----|-------|----|-------|----------|----------|--------|
       1  15.357 MM    0.3675 1022.46527   46.36811   2.9897
       2  18.247 MM    0.4575 3.31774e4  1208.65466  97.0103

    Totals :                  3.41999e4  1255.02277


    =====================================================================
                             *** End of Report ***
```

1260HPLC-VWD 10/23/2016 8:49:53 PM SYSTEM Page 1 of 1

Supplementary Figure 94. HPLC spectra for compound 2g

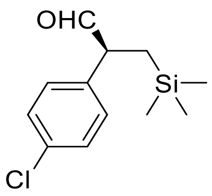

2h

Data File E:\DATA\HZY\SUANGAN\PDC-BINAP 2016-10-24 15-03-05\073-1201.D
Sample Name: YC-4-161-2-RAC

===

Acq. Operator : SYSTEM Seq. Line : 12
Acq. Instrument : 1260HPLC-VWD Location : Vial 73
Injection Date : 10/24/2016 10:00:46 PM Inj : 1
Inj Volume : 2.000 µl
Acq. Method : E:\DATA\HZY\SUANGAN\PDC-BINAP 2016-10-24 15-03-05\VWD-ADH(1-6)-95-5-0.3ML-210NM-40MIN.M
Last changed : 10/24/2016 10:02:21 PM by SYSTEM
(modified after loading)
Analysis Method : E:\DATA\HZY\SUANGAN\PDC-BINAP 2016-10-24 15-03-05\VWD-ADH(1-6)-95-5-0.3ML-210NM-40MIN.M (Sequence Method)
Last changed : 10/25/2016 8:55:49 AM by SYSTEM
(modified after loading)
Additional Info : Peak(s) manually integrated

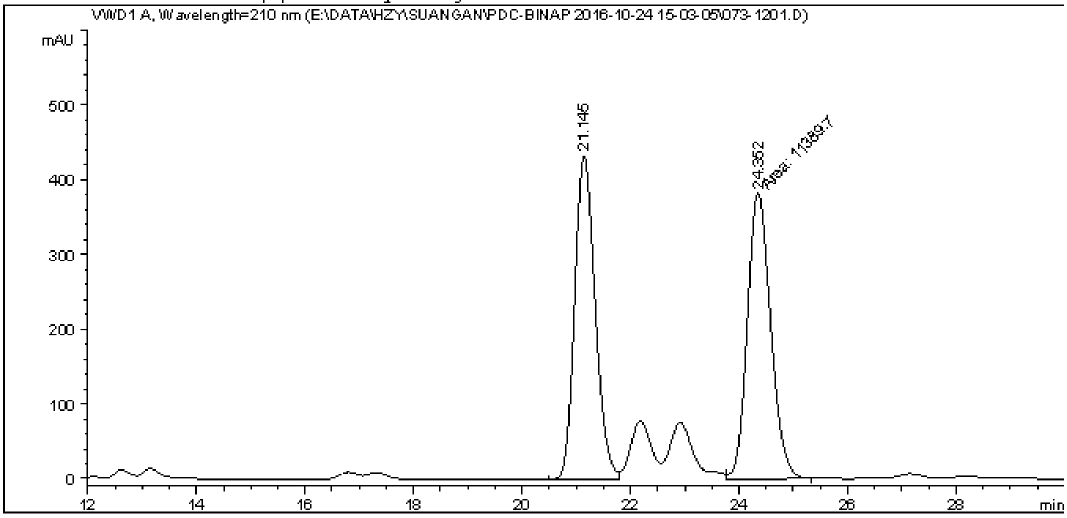

===

Area Percent Report

===

Sorted By : Signal
Multiplier : 1.0000
Dilution : 1.0000
Do not use Multiplier & Dilution Factor with ISTDs

Signal 1: VWD1 A, Wavelength=210 nm

Peak #	RetTime [min]	Type	Width [min]	Area [mAU*s]	Height [mAU]	Area %
1	21.145	BV	0.4057	1.13378e4	432.70929	49.8859
2	24.352	MM	0.4966	1.13897e4	382.25385	50.1141

Totals : 2.27275e4 814.96313

===

*** End of Report ***

Data File E:\DATA\HZY\SUANGAN\PDC-BINAP 2016-10-24 15-03-05\074-1301.D
Sample Name: YC-4-161-2

```
=====================================================================
Acq. Operator   : SYSTEM                        Seq. Line :  13
Acq. Instrument : 1260HPLC-VWD                   Location : Vial 74
Injection Date  : 10/24/2016 10:41:34 PM              Inj :   1
                                               Inj Volume : 2.000 µl
Acq. Method     : E:\DATA\HZY\SUANGAN\PDC-BINAP 2016-10-24 15-03-05\VWD-ADH(1-6)-95-5-0.
                  3ML-210NM-40MIN.M
Last changed    : 10/24/2016 10:02:21 PM by SYSTEM
Analysis Method : E:\DATA\HZY\SUANGAN\PDC-BINAP 2016-10-24 15-03-05\VWD-ADH(1-6)-95-5-0.
                  3ML-210NM-40MIN.M (Sequence Method)
Last changed    : 10/25/2016 8:58:23 AM by SYSTEM
                  (modified after loading)
Additional Info : Peak(s) manually integrated
```

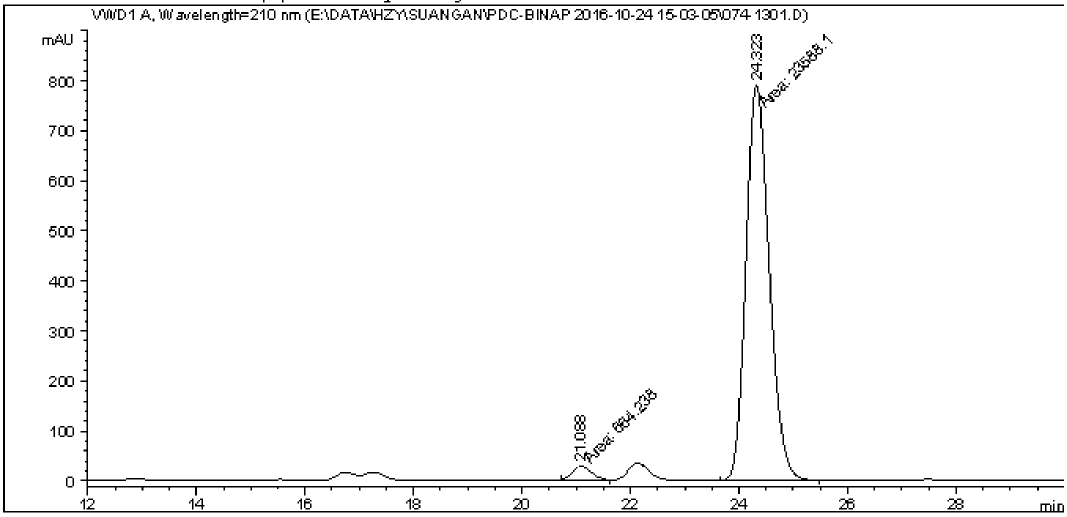


```
=====================================================================
                         Area Percent Report
=====================================================================

Sorted By             :      Signal
Multiplier            :      1.0000
Dilution              :      1.0000
Do not use Multiplier & Dilution Factor with ISTDs


Signal 1: VWD1 A, Wavelength=210 nm
```

Peak #	RetTime [min]	Type	Width [min]	Area [mAU*s]	Height [mAU]	Area %
1	21.088	MM	0.4080	664.23840	27.13119	2.7389
2	24.323	MM	0.4975	2.35881e4	790.15875	97.2611

Totals : 2.42524e4 817.28995

```
=====================================================================
                          *** End of Report ***
```


Supplementary Figure 95. HPLC spectra for compound 2h

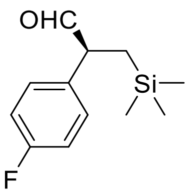

2i

```
Data File E:\DATA\YHL\YHL-5-201\YHL-5-201 2016-10-18 19-51-50\092-1301.D
Sample Name: YC-4-156-1-RAC

    =====================================================================
    Acq. Operator   : SYSTEM                        Seq. Line :  13
    Acq. Instrument : 1260HPLC-VWD                   Location : Vial 92
    Injection Date  : 10/19/2016 2:01:47 AM               Inj :   1
                                                   Inj Volume : 2.000 µl
    Acq. Method     : E:\DATA\YHL\YHL-5-201\YHL-5-201 2016-10-18 19-51-50\VWD-AD(1-6)-97-3-0.
                      5ML-2UL-210-30MIN.M
    Last changed    : 10/18/2016 9:48:40 PM by SYSTEM
    Analysis Method : E:\DATA\YHL\YHL-5-201\YHL-5-201 2016-10-18 19-51-50\VWD-AD(1-6)-97-3-0.
                      5ML-2UL-210-30MIN.M (Sequence Method)
    Last changed    : 10/19/2016 9:23:00 AM by SYSTEM
                      (modified after loading)
    Additional Info : Peak(s) manually integrated
```

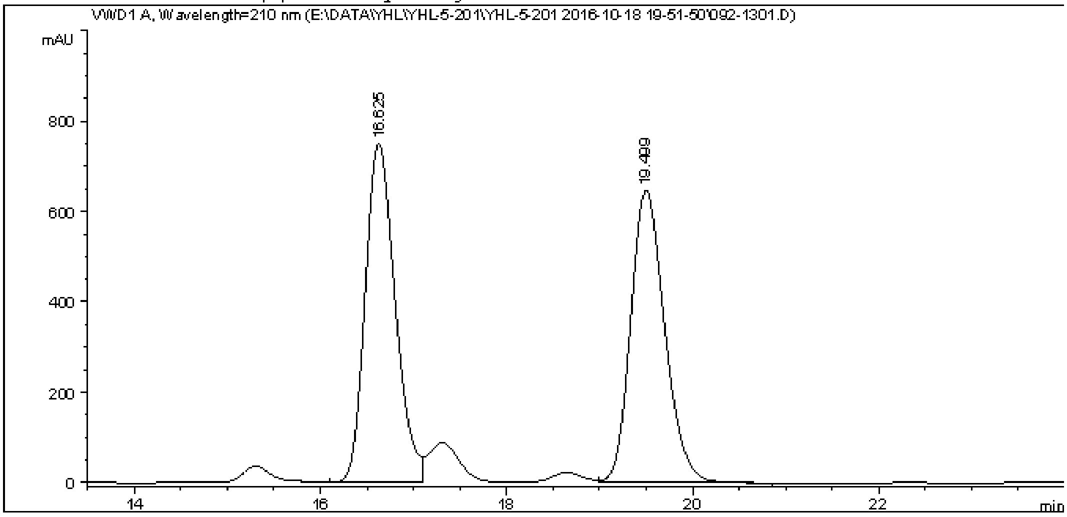


```
    =====================================================================
                            Area Percent Report
    =====================================================================

    Sorted By             :      Signal
    Multiplier            :      1.0000
    Dilution              :      1.0000
    Do not use Multiplier & Dilution Factor with ISTDs


    Signal 1: VWD1 A, Wavelength=210 nm

    Peak RetTime Type  Width     Area      Height     Area
      #   [min]        [min]   [mAU*s]     [mAU]        %
    ----|-------|----|-------|----------|----------|--------|
       1  16.625 BV    0.3452 1.67726e4  749.09235  50.5731
       2  19.499 VB    0.3919 1.63925e4  646.32410  49.4269

    Totals :                  3.31651e4  1395.41644


    =====================================================================
                            *** End of Report ***
```

Data File E:\DATA\YHL\YHL-5-201\YHL-5-201 2016-10-18 19-51-50\093-1401.D
Sample Name: YC-4-156-1

===

Acq. Operator : SYSTEM Seq. Line : 14
Acq. Instrument : 1260HPLC-VWD Location : Vial 93
Injection Date : 10/19/2016 2:32:31 AM Inj : 1
Inj Volume : 2.000 µl
Acq. Method : E:\DATA\YHL\YHL-5-201\YHL-5-201 2016-10-18 19-51-50\VWD-AD(1-6)-97-3-0.5ML-2UL-210-30MIN.M
Last changed : 10/18/2016 9:48:40 PM by SYSTEM
Analysis Method : E:\DATA\YHL\YHL-5-201\YHL-5-201 2016-10-18 19-51-50\VWD-AD(1-6)-97-3-0.5ML-2UL-210-30MIN.M (Sequence Method)
Last changed : 10/19/2016 9:27:34 AM by SYSTEM
(modified after loading)
Additional Info : Peak(s) manually integrated

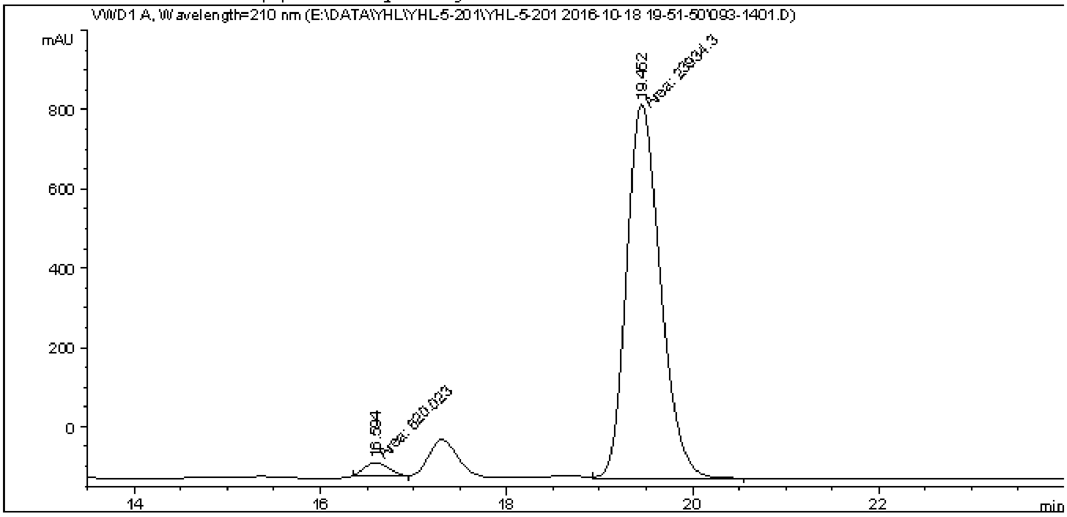

===

Area Percent Report

===

Sorted By : Signal
Multiplier : 1.0000
Dilution : 1.0000
Do not use Multiplier & Dilution Factor with ISTDs

Signal 1: VWD1 A, Wavelength=210 nm

Peak #	RetTime [min]	Type	Width [min]	Area [mAU*s]	Height [mAU]	Area %
1	16.594	MM	0.3076	620.02338	33.59301	2.5251
2	19.452	MM	0.4229	2.39343e4	943.35046	97.4749

Totals : 2.45543e4 976.94347

===

*** End of Report ***

1260HPLC-VWD 10/19/2016 9:27:39 AM SYSTEM Page 1 of 1

Supplementary Figure 96. HPLC spectra for compound 2i

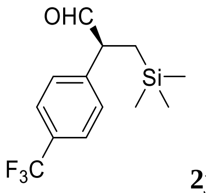

2j

Data File E:\DATA\HZY\SUANGAN\PDC-BINAP 2016-10-24 15-03-05\071-1001.D
Sample Name: YC-4-161-1-RAC

```
=====================================================================
Acq. Operator   : SYSTEM                        Seq. Line :  10
Acq. Instrument : 1260HPLC-VWD                   Location : Vial 71
Injection Date  : 10/24/2016 8:50:54 PM               Inj :   1
                                               Inj Volume : 2.000 µl
Acq. Method     : E:\DATA\HZY\SUANGAN\PDC-BINAP 2016-10-24 15-03-05\VWD-ADH(1-6)-95-5-0.
                  3ML-210NM-40MIN.M
Last changed    : 10/24/2016 8:09:59 PM by SYSTEM
Analysis Method : E:\DATA\HZY\SUANGAN\PDC-BINAP 2016-10-24 15-03-05\VWD-ADH(1-6)-95-5-0.
                  3ML-210NM-40MIN.M (Sequence Method)
Last changed    : 10/24/2016 9:58:18 PM by SYSTEM
                  (modified after loading)
Additional Info : Peak(s) manually integrated
```

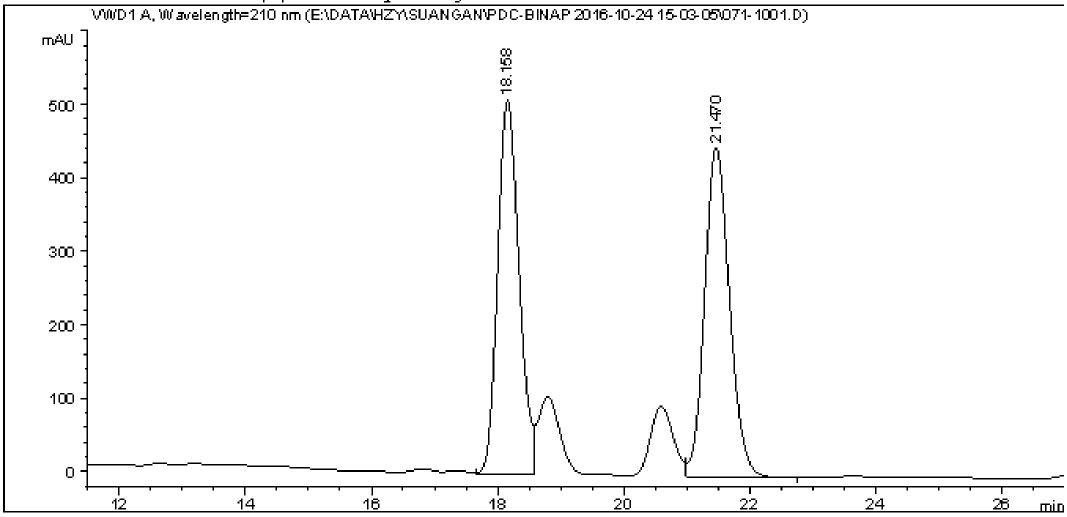


```
=====================================================================
                         Area Percent Report
=====================================================================

Sorted By             :      Signal
Multiplier            :      1.0000
Dilution              :      1.0000
Do not use Multiplier & Dilution Factor with ISTDs


Signal 1: VWD1 A, Wavelength=210 nm
```

Peak #	RetTime [min]	Type	Width [min]	Area [mAU*s]	Height [mAU]	Area %
1	18.158	BV	0.3661	1.19837e4	508.16574	49.3373
2	21.470	VB	0.4232	1.23056e4	448.39621	50.6627
Totals :				2.42893e4	956.56195	

===
*** End of Report ***

Data File E:\DATA\HZY\SUANGAN\PDC-BINAP 2016-10-24 15-03-05\072-1101.D
Sample Name: YC-4-161-1

===

Acq. Operator : SYSTEM Seq. Line : 11
Acq. Instrument : 1260HPLC-VWD Location : Vial 72
Injection Date : 10/24/2016 9:31:38 PM Inj : 1
Inj Volume : 2.000 µl
Acq. Method : E:\DATA\HZY\SUANGAN\PDC-BINAP 2016-10-24 15-03-05\VWD-ADH(1-6)-95-5-0.3ML-210NM-40MIN.M
Last changed : 10/24/2016 10:00:01 PM by SYSTEM
(modified after loading)
Analysis Method : E:\DATA\HZY\SUANGAN\PDC-BINAP 2016-10-24 15-03-05\VWD-ADH(1-6)-95-5-0.3ML-210NM-40MIN.M (Sequence Method)
Last changed : 10/24/2016 10:01:39 PM by SYSTEM
(modified after loading)
Additional Info : Peak(s) manually integrated

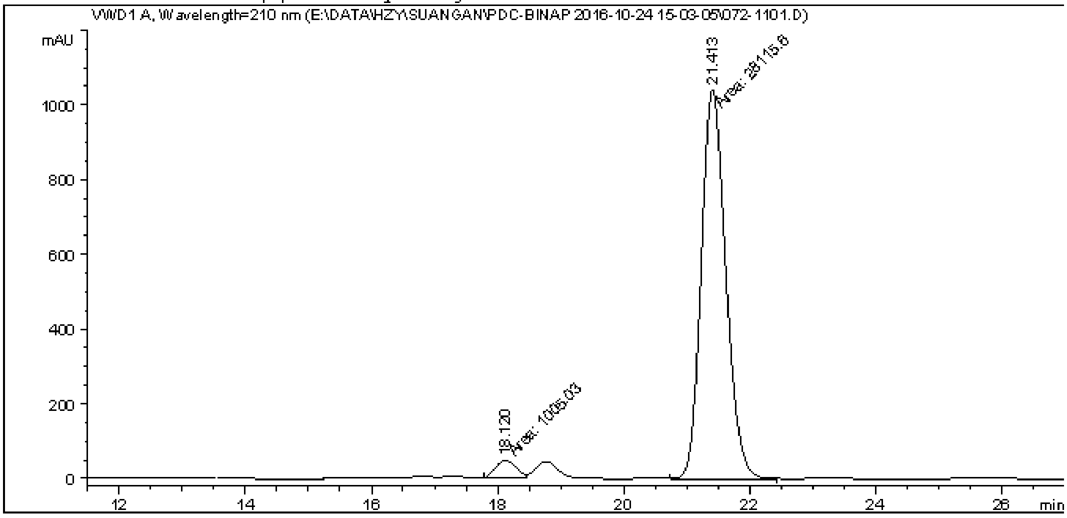

===

Area Percent Report

===

Sorted By : Signal
Multiplier : 1.0000
Dilution : 1.0000
Do not use Multiplier & Dilution Factor with ISTDs

Signal 1: VWD1 A, Wavelength=210 nm

Peak #	RetTime [min]	Type	Width [min]	Area [mAU*s]	Height [mAU]	Area %
1	18.120	MM	0.3618	1005.03363	46.29342	3.4513
2	21.413	MM	0.4498	2.81156e4	1041.75647	96.5487

Totals : 2.91206e4 1088.04988

===

*** End of Report ***

1260HPLC-VWD 10/24/2016 10:01:45 PM SYSTEM Page 1 of 1

Supplementary Figure 97. HPLC spectra for compound 2j

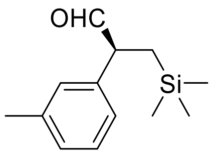

2k

Data File E:\DATA\YC\YC-4-177-OD\YC-4-177-OD 2016-11-18 17-10-52\015-0701.D
Sample Name: YC-4-177-3-RAC

===

Acq. Operator : SYSTEM Seq. Line : 7
Acq. Instrument : 1260HPLC-DAD Location : Vial 15
Injection Date : 11/18/2016 9:57:29 PM Inj : 1
Inj Volume : 3.000 µl
Acq. Method : E:\DATA\YC\YC-4-177-OD\YC-4-177-OD 2016-11-18 17-10-52\DAD-OD(1-2)-97-3-0.5ML-3UL-210-NM-40MIN.M
Last changed : 11/18/2016 5:10:53 PM by SYSTEM
Analysis Method : E:\DATA\YC\YC-4-177-OD\YC-4-177-OD 2016-11-18 17-10-52\DAD-OD(1-2)-97-3-0.5ML-3UL-210-NM-40MIN.M (Sequence Method)
Last changed : 11/19/2016 8:35:51 PM by SYSTEM
(modified after loading)
Additional Info : Peak(s) manually integrated

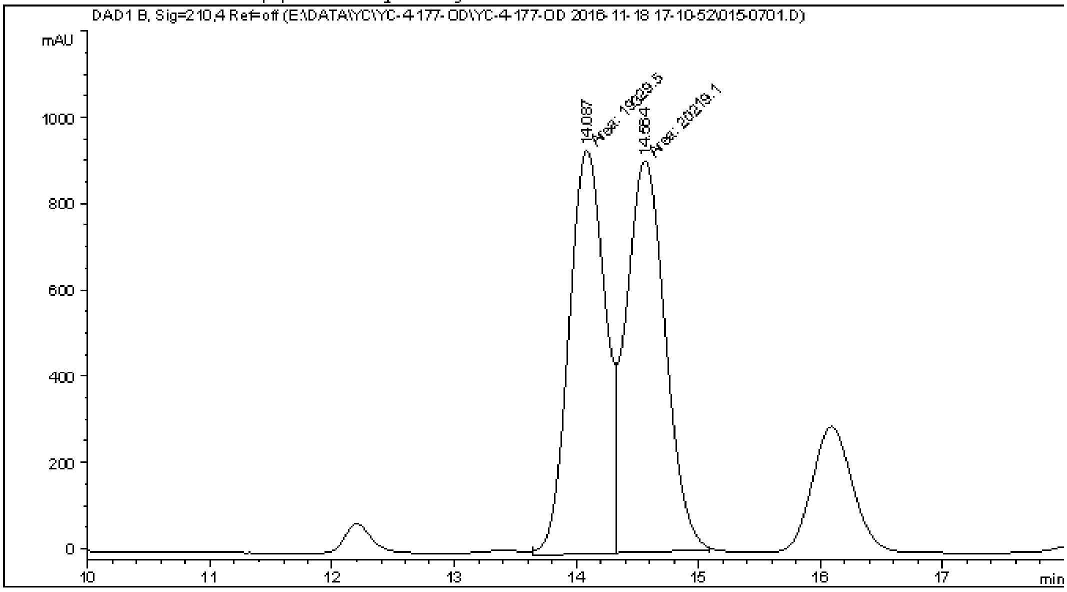

===

Area Percent Report

===

Sorted By : Signal
Multiplier : 1.0000
Dilution : 1.0000
Do not use Multiplier & Dilution Factor with ISTDs

Signal 1: DAD1 B, Sig=210,4 Ref=off

Peak #	RetTime [min]	Type	Width [min]	Area [mAU*s]	Height [mAU]	Area %
1	14.087	MF	0.3446	1.93295e4	934.85016	48.8753
2	14.564	FM	0.3716	2.02191e4	906.82324	51.1247
Totals :				3.95486e4	1841.67340	

===

*** End of Report ***

```
Data File E:\DATA\YC\YC-4-177-OD\YC-4-177-OD 2016-11-18 17-10-52\016-0801.D
Sample Name: YC-4-177-3

=====================================================================
Acq. Operator   : SYSTEM                        Seq. Line :   8
Acq. Instrument : 1260HPLC-DAD                   Location : Vial 16
Injection Date  : 11/18/2016 10:38:22 PM              Inj :   1
                                               Inj Volume : 3.000 µl
Acq. Method     : E:\DATA\YC\YC-4-177-OD\YC-4-177-OD 2016-11-18 17-10-52\DAD-OD(1-2)-97-3-
                  0.5ML-3UL-210-NM-40MIN.M
Last changed    : 11/18/2016 5:10:53 PM by SYSTEM
Analysis Method : E:\DATA\YC\YC-4-177-OD\YC-4-177-OD 2016-11-18 17-10-52\DAD-OD(1-2)-97-3-
                  0.5ML-3UL-210-NM-40MIN.M (Sequence Method)
Last changed    : 11/19/2016 8:36:57 PM by SYSTEM
                  (modified after loading)
Additional Info : Peak(s) manually integrated
```

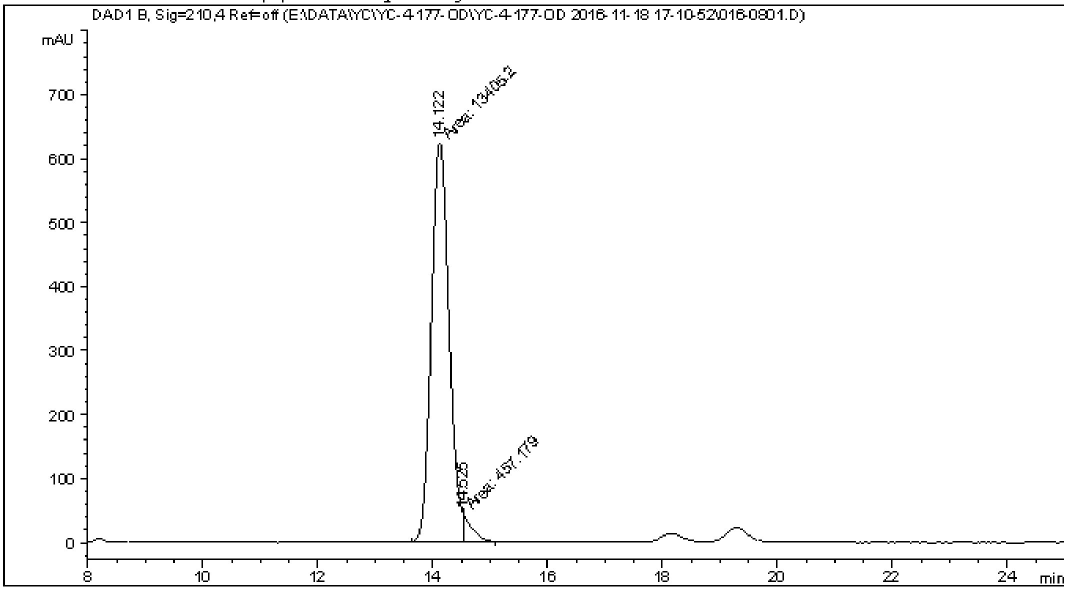


```
=====================================================================
                        Area Percent Report
=====================================================================

Sorted By             :      Signal
Multiplier            :      1.0000
Dilution              :      1.0000
Do not use Multiplier & Dilution Factor with ISTDs


Signal 1: DAD1 B, Sig=210,4 Ref=off

Peak RetTime Type  Width     Area      Height     Area
  #   [min]        [min]   [mAU*s]     [mAU]        %
----|-------|----|-------|----------|----------|--------|
   1  14.122 MF    0.3584 1.34052e4   623.45337  96.7020
   2  14.525 FM    0.1697  457.17908   44.89913   3.2980

Totals :                  1.38624e4   668.35250


=====================================================================
                         *** End of Report ***

1260HPLC-DAD 11/19/2016 8:37:04 PM SYSTEM                              Page 1 of 1
```

Supplementary Figure 98. HPLC spectra for compound 2k

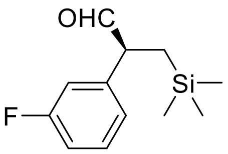

2l

Data File E:\DATA\WFY\WFY-2-MS\AD-MS-1223 2016-12-23 10-34-52\083-0401.D
Sample Name: YC-4-192-RAC

===

Acq. Operator : SYSTEM Seq. Line : 4
Acq. Instrument : 1260HPLC-VWD Location : Vial 83
Injection Date : 12/23/2016 11:32:57 AM Inj : 1
Inj Volume : 3.000 µl
Acq. Method : E:\DATA\WFY\WFY-2-MS\AD-MS-1223 2016-12-23 10-34-52\VWD-AD(1-6)-97-3-0.5ML-3UL-210NM-40MIN.M
Last changed : 12/23/2016 11:53:36 AM by SYSTEM
(modified after loading)
Analysis Method : E:\DATA\WFY\WFY-2-MS\AD-MS-1223 2016-12-23 10-34-52\VWD-AD(1-6)-97-3-0.5ML-3UL-210NM-40MIN.M (Sequence Method)
Last changed : 12/23/2016 12:44:08 PM by SYSTEM
(modified after loading)
Additional Info : Peak(s) manually integrated

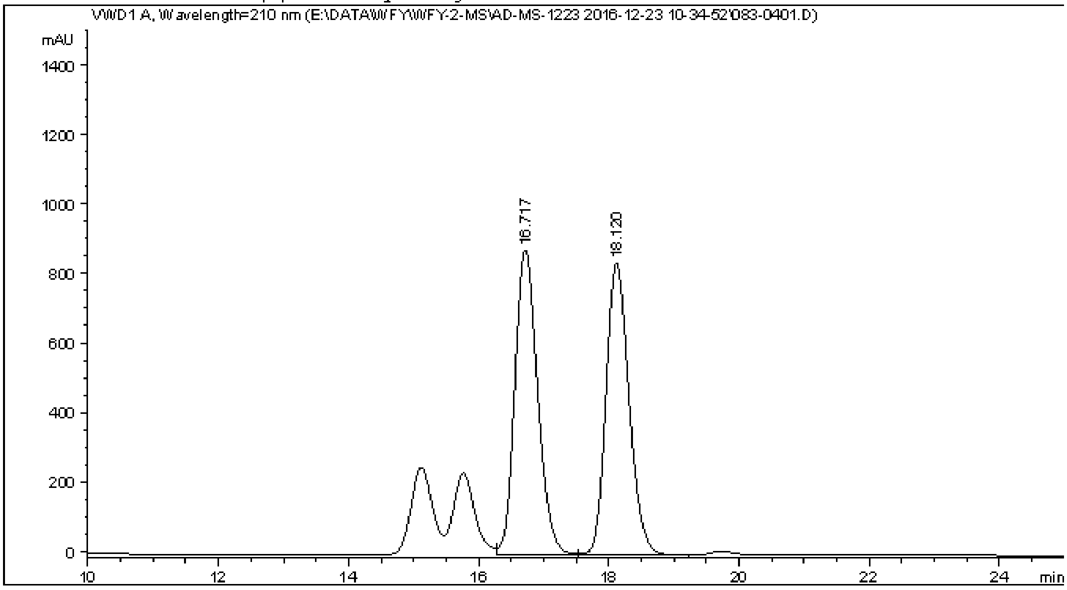

===

Area Percent Report

===

Sorted By : Signal
Multiplier : 1.0000
Dilution : 1.0000
Do not use Multiplier & Dilution Factor with ISTDs

Signal 1: VWD1 A, Wavelength=210 nm

Peak #	RetTime [min]	Type	Width [min]	Area [mAU*s]	Height [mAU]	Area %
1	16.717	VB	0.3755	2.11798e4	874.76770	50.8116
2	18.120	BB	0.3795	2.05032e4	837.80731	49.1884

Totals : 4.16830e4 1712.57501

===

1260HPLC-VWD 12/23/2016 12:45:10 PM SYSTEM Page 1 of 1

```
Data File E:\DATA\WFY\WFY-2-MS\AD-MS-1223 2016-12-23 10-34-52\084-0501.D
Sample Name: YC-4-192

=====================================================================
Acq. Operator   : SYSTEM                          Seq. Line :   5
Acq. Instrument : 1260HPLC-VWD                     Location : Vial 84
Injection Date  : 12/23/2016 12:03:42 PM                Inj :   1
                                                 Inj Volume : 3.000 µl
Acq. Method     : E:\DATA\WFY\WFY-2-MS\AD-MS-1223 2016-12-23 10-34-52\VWD-AD(1-6)-97-3-0.
                  5ML-3UL-210NM-40MIN.M
Last changed    : 12/23/2016 11:53:36 AM by SYSTEM
Analysis Method : E:\DATA\WFY\WFY-2-MS\AD-MS-1223 2016-12-23 10-34-52\VWD-AD(1-6)-97-3-0.
                  5ML-3UL-210NM-40MIN.M (Sequence Method)
Last changed    : 12/23/2016 12:44:08 PM by SYSTEM
                  (modified after loading)
Additional Info : Peak(s) manually integrated
```

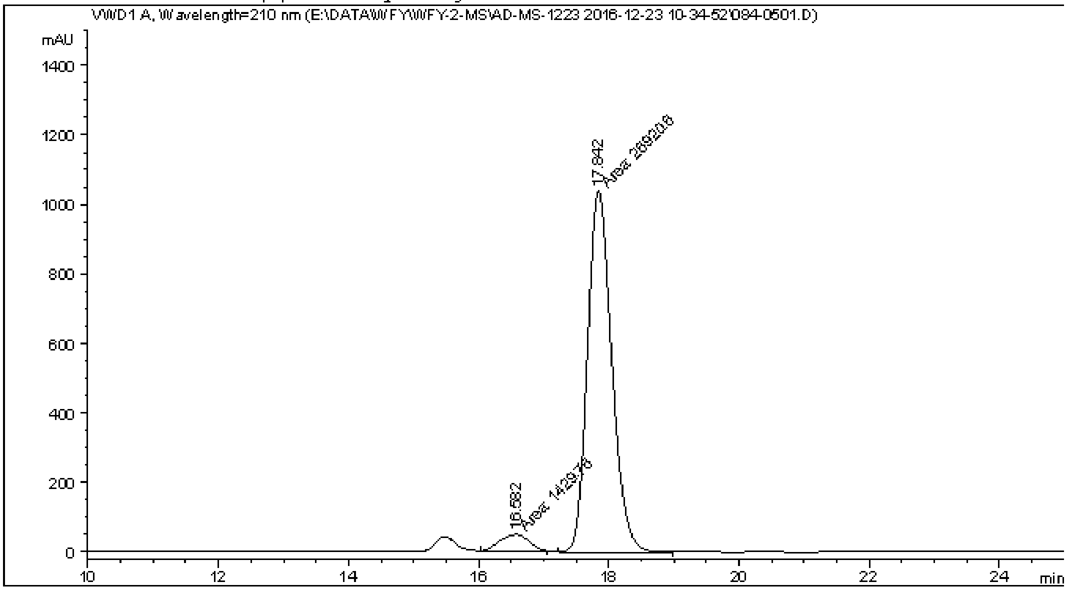


```
=====================================================================
                         Area Percent Report
=====================================================================

Sorted By             :      Signal
Multiplier            :      1.0000
Dilution              :      1.0000
Do not use Multiplier & Dilution Factor with ISTDs


Signal 1: VWD1 A, Wavelength=210 nm

Peak RetTime Type  Width     Area      Height     Area
  #   [min]        [min]   [mAU*s]     [mAU]        %
----|-------|----|-------|----------|----------|--------|
   1  16.582 MM    0.5145 1429.76477   46.31187   5.0432
   2  17.842 MM    0.4324 2.69206e4  1037.56006  94.9568

Totals :                  2.83503e4  1083.87193

=====================================================================
                          *** End of Report ***
```

1260HPLC-VWD 12/23/2016 12:44:13 PM SYSTEM   Page 1 of 1

Supplementary Figure 99. HPLC spectra for compound 2l

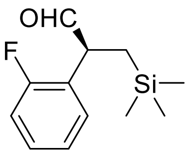

2m

Data File E:\DATA\YHL\YHL-5-204\YHL-5-204 2016-10-23 15-17-32\092-0501.D
Sample Name: YC-4-159-2-RAC

===

Acq. Operator : SYSTEM Seq. Line : 5
Acq. Instrument : 1260HPLC-VWD Location : Vial 92
Injection Date : 10/23/2016 5:06:20 PM Inj : 1
Inj Volume : 2.000 µl
Acq. Method : E:\DATA\YHL\YHL-5-204\YHL-5-204 2016-10-23 15-17-32\VWD-AD(1-6)-97-3-0.5ML-2UL-210-30MIN.M
Last changed : 10/23/2016 3:50:50 PM by SYSTEM
Analysis Method : E:\DATA\YHL\YHL-5-204\YHL-5-204 2016-10-23 15-17-32\VWD-AD(1-6)-97-3-0.5ML-2UL-210-30MIN.M (Sequence Method)
Last changed : 10/23/2016 6:56:11 PM by SYSTEM
(modified after loading)
Additional Info : Peak(s) manually integrated

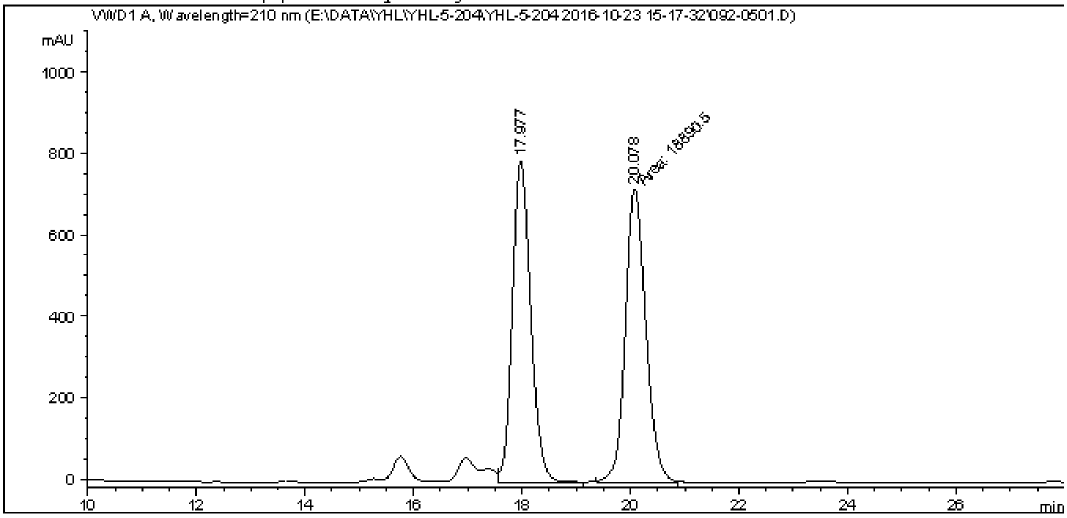

===

Area Percent Report

===

Sorted By : Signal
Multiplier : 1.0000
Dilution : 1.0000
Do not use Multiplier & Dilution Factor with ISTDs

Signal 1: VWD1 A, Wavelength=210 nm

Peak #	RetTime [min]	Type	Width [min]	Area [mAU*s]	Height [mAU]	Area %
1	17.977	VB	0.3625	1.85080e4	789.39410	49.4886
2	20.078	MM	0.4376	1.88905e4	719.49689	50.5114

Totals : 3.73986e4 1508.89099

===

*** End of Report ***

```
Data File E:\DATA\YHL\YHL-5-204\YHL-5-204 2016-10-23 15-17-32\093-0601.D
Sample Name: YC-4-159-2

    =====================================================================
    Acq. Operator   : SYSTEM                         Seq. Line :   6
    Acq. Instrument : 1260HPLC-VWD                    Location : Vial 93
    Injection Date  : 10/23/2016 5:37:04 PM                Inj :   1
                                                    Inj Volume : 2.000 µl
    Acq. Method     : E:\DATA\YHL\YHL-5-204\YHL-5-204 2016-10-23 15-17-32\VWD-AD(1-6)-97-3-0.
                      5ML-2UL-210-30MIN.M
    Last changed    : 10/23/2016 3:50:50 PM by SYSTEM
    Analysis Method : E:\DATA\YHL\YHL-5-204\YHL-5-204 2016-10-23 15-17-32\VWD-AD(1-6)-97-3-0.
                      5ML-2UL-210-30MIN.M (Sequence Method)
    Last changed    : 10/23/2016 6:54:59 PM by SYSTEM
                      (modified after loading)
    Additional Info : Peak(s) manually integrated
```

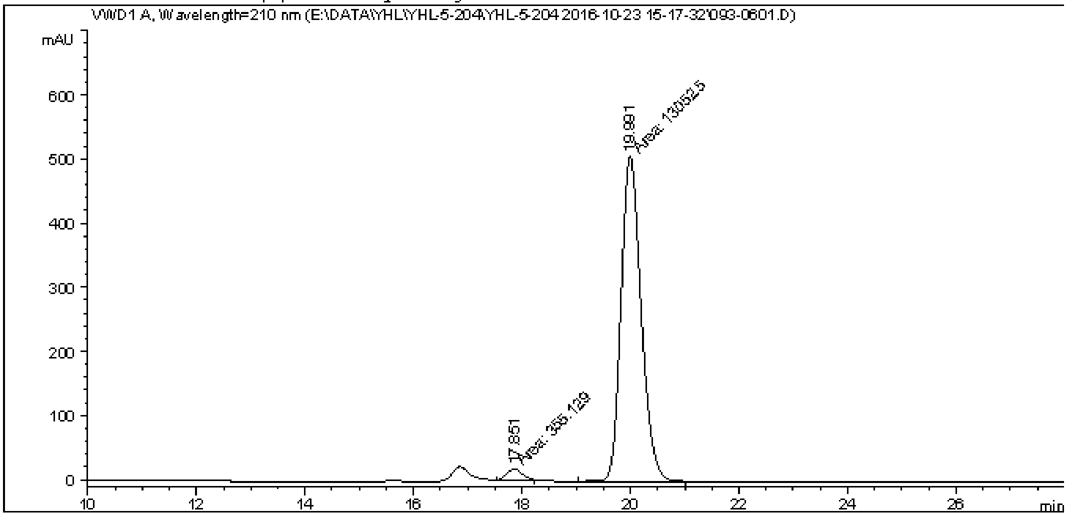


```
    =====================================================================
                         Area Percent Report
    =====================================================================

    Sorted By             :      Signal
    Multiplier            :      1.0000
    Dilution              :      1.0000
    Do not use Multiplier & Dilution Factor with ISTDs


    Signal 1: VWD1 A, Wavelength=210 nm

    Peak RetTime Type  Width     Area      Height     Area
      #   [min]        [min]   [mAU*s]     [mAU]        %
    ----|-------|----|-------|----------|----------|--------|
       1  17.851 MM    0.3430  355.12869   17.25850   2.6487
       2  19.991 MM    0.4297 1.30525e4   506.24280  97.3513

    Totals :                   1.34076e4   523.50130


    =====================================================================
                          *** End of Report ***


1260HPLC-VWD 10/23/2016 6:55:04 PM SYSTEM                          Page 1 of 1
```

Supplementary Figure 100. HPLC spectra for compound 2m

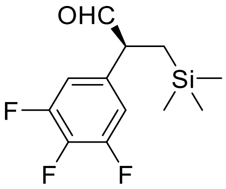

2n

```
Data File E:\DATA\YC\YC-4-170\YC-4-170 2016-11-05 12-07-02\015-0701.D
Sample Name: YC-4-170-3-RAC

    =====================================================================
    Acq. Operator   : SYSTEM                        Seq. Line :   7
    Acq. Instrument : 1260HPLC-VWD                   Location : Vial 15
    Injection Date  : 11/5/2016 3:15:29 PM                Inj :   1
                                                   Inj Volume : 2.000 µl
    Acq. Method     : E:\DATA\YC\YC-4-170\YC-4-170 2016-11-05 12-07-02\VWD-AD(1-6)-97-3-0.5ML-
                      2UL-210-30MIN.M
    Last changed    : 11/5/2016 12:31:11 PM by SYSTEM
    Analysis Method : E:\DATA\YC\YC-4-170\YC-4-170 2016-11-05 12-07-02\VWD-AD(1-6)-97-3-0.5ML-
                      2UL-210-30MIN.M (Sequence Method)
    Last changed    : 11/5/2016 4:12:07 PM by SYSTEM
                      (modified after loading)
    Additional Info : Peak(s) manually integrated
```

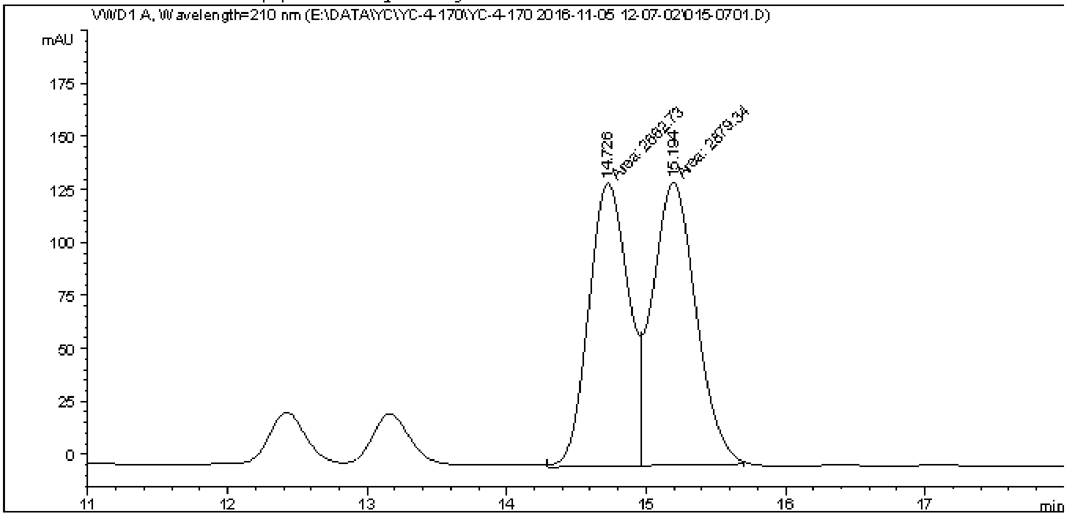


```
    =====================================================================
                             Area Percent Report
    =====================================================================

    Sorted By             :      Signal
    Multiplier            :      1.0000
    Dilution              :      1.0000
    Do not use Multiplier & Dilution Factor with ISTDs


    Signal 1: VWD1 A, Wavelength=210 nm

    Peak RetTime Type  Width     Area      Height     Area
      #   [min]        [min]   [mAU*s]     [mAU]       %
    ----|-------|----|-------|----------|----------|--------|
       1  14.726 MF    0.3325 2662.72729  133.48756  48.0457
       2  15.194 FM    0.3600 2879.34326  133.31422  51.9543

    Totals :                  5542.07056  266.80179


    =====================================================================
                             *** End of Report ***
```

Data File E:\DATA\YC\YC-4-170\YC-4-170 2016-11-05 12-07-02\016-0801.D
Sample Name: YC-4-170-3

===

Acq. Operator : SYSTEM Seq. Line : 8
Acq. Instrument : 1260HPLC-VWD Location : Vial 16
Injection Date : 11/5/2016 3:46:12 PM Inj : 1
Inj Volume : 2.000 µl
Acq. Method : E:\DATA\YC\YC-4-170\YC-4-170 2016-11-05 12-07-02\VWD-AD(1-6)-97-3-0.5ML-2UL-210-30MIN.M
Last changed : 11/5/2016 4:07:26 PM by SYSTEM
(modified after loading)
Analysis Method : E:\DATA\YC\YC-4-170\YC-4-170 2016-11-05 12-07-02\VWD-AD(1-6)-97-3-0.5ML-2UL-210-30MIN.M (Sequence Method)
Last changed : 11/5/2016 4:17:07 PM by SYSTEM
(modified after loading)
Additional Info : Peak(s) manually integrated

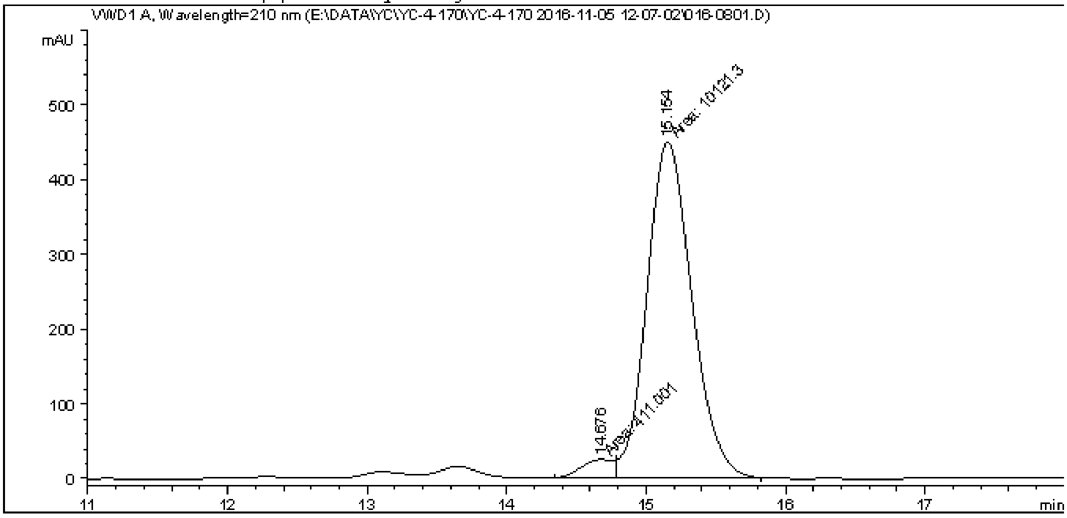

===

Area Percent Report

===

Sorted By : Signal
Multiplier : 1.0000
Dilution : 1.0000
Do not use Multiplier & Dilution Factor with ISTDs

Signal 1: VWD1 A, Wavelength=210 nm

Peak #	RetTime [min]	Type	Width [min]	Area [mAU*s]	Height [mAU]	Area %
1	14.676	MF	0.2736	411.00061	25.04113	3.9023
2	15.154	FM	0.3745	1.01213e4	450.37656	96.0977

Totals : 1.05323e4 475.41768

===

*** End of Report ***

1260HPLC-VWD 11/5/2016 4:18:14 PM SYSTEM Page 1 of 1

Supplementary Figure 101. HPLC spectra for compound 2n

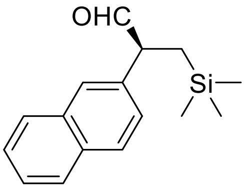

2o

Data File E:\DATA\YC\YC-4-170\YC-4-170 2016-11-05 12-07-02\011-0201.D
Sample Name: YC-4-170-1-RAC

```
=====================================================================
Acq. Operator   : SYSTEM                          Seq. Line :   2
Acq. Instrument : 1260HPLC-VWD                     Location : Vial 11
Injection Date  : 11/5/2016 12:18:37 PM                 Inj :   1
                                                Inj Volume : 4.000 µl
Acq. Method     : E:\DATA\YC\YC-4-170\YC-4-170 2016-11-05 12-07-02\VWD-AD(1-6)-99-1-1.0ML-
                  210NM-40MIN.M
Last changed    : 11/5/2016 12:32:46 PM by SYSTEM
                  (modified after loading)
Analysis Method : E:\DATA\YC\YC-4-170\YC-4-170 2016-11-05 12-07-02\VWD-AD(1-6)-99-1-1.0ML-
                  210NM-40MIN.M (Sequence Method)
Last changed    : 11/5/2016 2:59:58 PM by SYSTEM
                  (modified after loading)
Additional Info : Peak(s) manually integrated
```

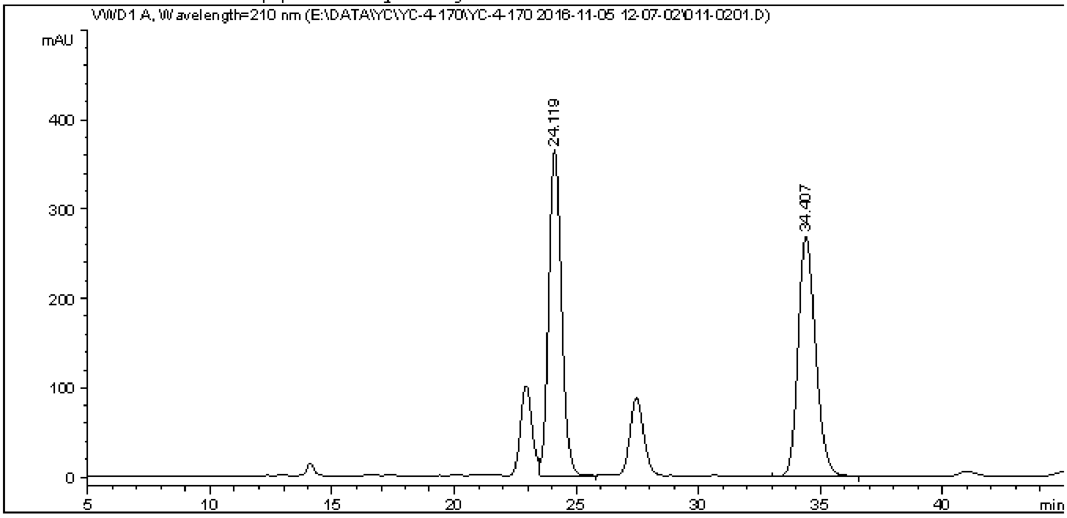


```
=====================================================================
                         Area Percent Report
=====================================================================

Sorted By             :      Signal
Multiplier            :      1.0000
Dilution              :      1.0000
Do not use Multiplier & Dilution Factor with ISTDs


Signal 1: VWD1 A, Wavelength=210 nm
```

Peak #	RetTime [min]	Type	Width [min]	Area [mAU*s]	Height [mAU]	Area %
1	24.119	VB	0.5655	1.32595e4	363.57300	49.8551
2	34.407	BB	0.7637	1.33366e4	268.38843	50.1449

Totals : 2.65960e4 631.96143

```
=====================================================================
                          *** End of Report ***
```

Data File E:\DATA\YC\YC-4-170\YC-4-170 2016-11-05 12-07-02\012-0301.D
Sample Name: YC-4-170-1

===

Acq. Operator : SYSTEM　　Seq. Line : 3
Acq. Instrument : 1260HPLC-VWD　　Location : Vial 12
Injection Date : 11/5/2016 1:09:20 PM　　Inj : 1
Inj Volume : 4.000 µl
Acq. Method : E:\DATA\YC\YC-4-170\YC-4-170 2016-11-05 12-07-02\VWD-AD(1-6)-99-1-1.0ML-210NM-40MIN.M
Last changed : 11/5/2016 1:57:19 PM by SYSTEM
(modified after loading)
Analysis Method : E:\DATA\YC\YC-4-170\YC-4-170 2016-11-05 12-07-02\VWD-AD(1-6)-99-1-1.0ML-210NM-40MIN.M (Sequence Method)
Last changed : 11/5/2016 3:00:48 PM by SYSTEM
(modified after loading)
Additional Info : Peak(s) manually integrated

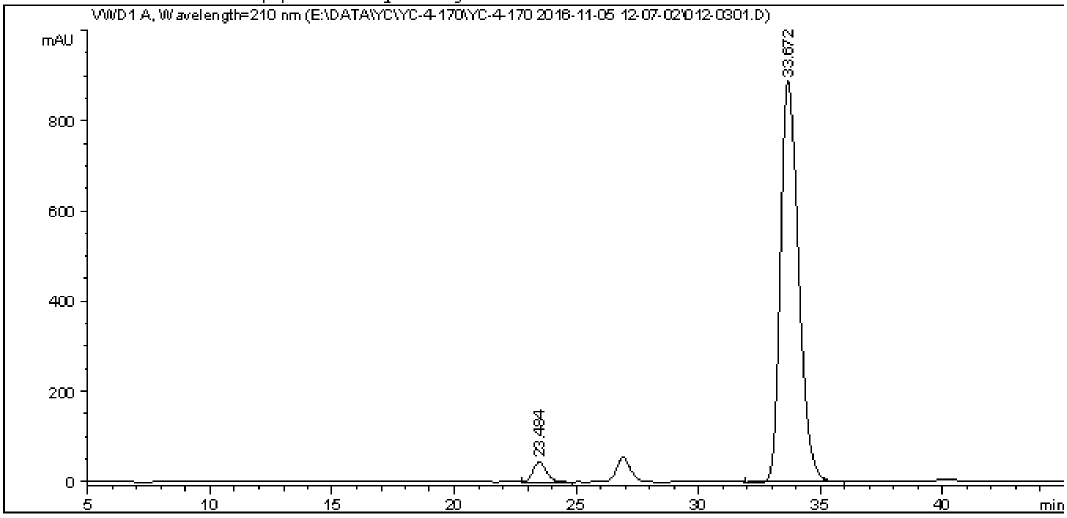

===

Area Percent Report

===

Sorted By : Signal
Multiplier : 1.0000
Dilution : 1.0000
Do not use Multiplier & Dilution Factor with ISTDs

Signal 1: VWD1 A, Wavelength=210 nm

Peak #	RetTime [min]	Type	Width [min]	Area [mAU*s]	Height [mAU]	Area %
1	23.484	VB	0.5697	1665.63098	44.70560	3.5052
2	33.672	BB	0.8100	4.58529e4	888.57343	96.4948

Totals : 4.75185e4 933.27903

===

*** End of Report ***

1260HPLC-VWD 11/5/2016 3:00:53 PM SYSTEM　　Page 1 of 1

Supplementary Figure 102. HPLC spectra for compound 2o

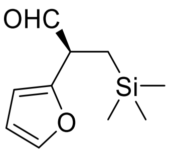

2p

Data File E:\DATA\WQ\WQ-3-53\WQ-3-54-4 2016-10-28 10-59-41\043-1301.D
Sample Name: YC-4-167-2-RAC-1

===

Acq. Operator : SYSTEM Seq. Line : 13
Acq. Instrument : 1260HPLC-VWD Location : Vial 43
Injection Date : 10/28/2016 4:56:09 PM Inj : 1
Inj Volume : 4.000 µl
Acq. Method : E:\DATA\WQ\WQ-3-53\WQ-3-54-4 2016-10-28 10-59-41\VWD-AD(1-6)-99-1-1.0ML-210NM-40MIN.M
Last changed : 10/28/2016 5:17:31 PM by SYSTEM
(modified after loading)
Analysis Method : E:\DATA\WQ\WQ-3-53\WQ-3-54-4 2016-10-28 10-59-41\VWD-AD(1-6)-99-1-1.0ML-210NM-40MIN.M (Sequence Method)
Last changed : 10/28/2016 5:57:17 PM by SYSTEM
(modified after loading)
Additional Info : Peak(s) manually integrated

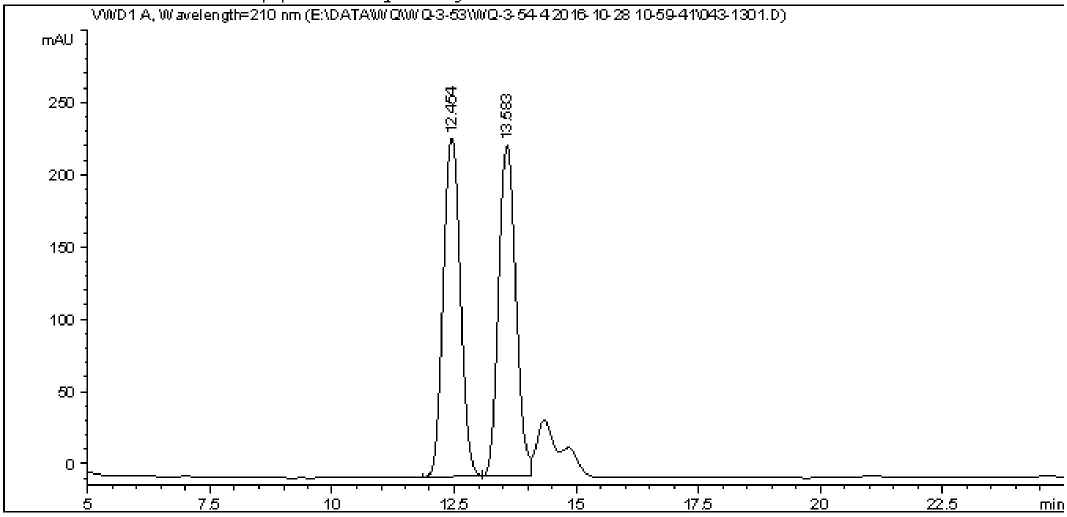

===

Area Percent Report

===

Sorted By : Signal
Multiplier : 1.0000
Dilution : 1.0000
Do not use Multiplier & Dilution Factor with ISTDs

Signal 1: VWD1 A, Wavelength=210 nm

Peak #	RetTime [min]	Type	Width [min]	Area [mAU*s]	Height [mAU]	Area %
1	12.454	BB	0.3914	5717.02148	233.68697	50.3292
2	13.583	BV	0.3918	5642.23779	228.75363	49.6708

Totals : 1.13593e4 462.44060

===

*** End of Report ***

1260HPLC-VWD 10/28/2016 5:57:20 PM SYSTEM Page 1 of 1

```
Data File E:\DATA\WQ\WQ-3-53\WQ-3-54-4 2016-10-28 10-59-41\044-1401.D
Sample Name: YC-4-167-2-1

    =====================================================================
    Acq. Operator   : SYSTEM                          Seq. Line :  14
    Acq. Instrument : 1260HPLC-VWD                     Location : Vial 44
    Injection Date  : 10/28/2016 5:26:55 PM                 Inj :   1
                                                     Inj Volume : 4.000 µl
    Acq. Method     : E:\DATA\WQ\WQ-3-53\WQ-3-54-4 2016-10-28 10-59-41\VWD-AD(1-6)-99-1-1.0ML-
                      210NM-40MIN.M
    Last changed    : 10/28/2016 5:53:08 PM by SYSTEM
                      (modified after loading)
    Analysis Method : E:\DATA\WQ\WQ-3-53\WQ-3-54-4 2016-10-28 10-59-41\VWD-AD(1-6)-99-1-1.0ML-
                      210NM-40MIN.M (Sequence Method)
    Last changed    : 10/28/2016 5:55:57 PM by SYSTEM
                      (modified after loading)
    Additional Info : Peak(s) manually integrated
```

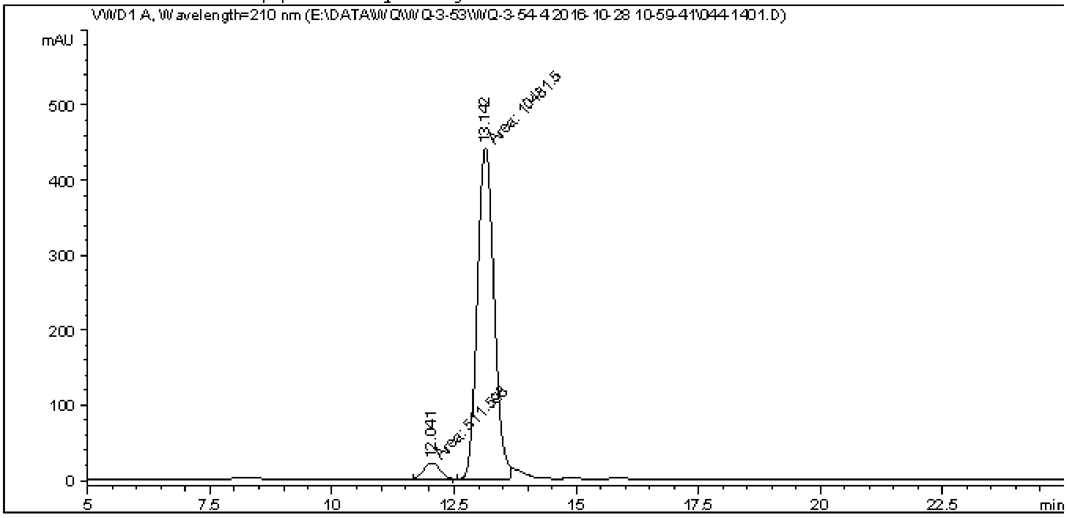


```
    =====================================================================
                             Area Percent Report
    =====================================================================

    Sorted By             :      Signal
    Multiplier            :      1.0000
    Dilution              :      1.0000
    Do not use Multiplier & Dilution Factor with ISTDs


    Signal 1: VWD1 A, Wavelength=210 nm

    Peak RetTime Type  Width     Area      Height     Area
      #  [min]        [min]   [mAU*s]     [mAU]        %
    ----|-------|----|-------|----------|----------|--------|
       1  12.041 MM    0.3942  511.59763   21.63086   4.6538
       2  13.142 MF    0.3963 1.04815e4   440.82993  95.3462

    Totals :                  1.09931e4   462.46078


    =====================================================================
                             *** End of Report ***


1260HPLC-VWD 10/28/2016 5:56:00 PM SYSTEM                          Page 1 of 1
```

Supplementary Figure 103. HPLC spectra for compound 2p

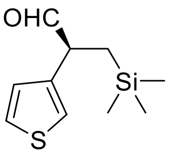

2q

Data File E:\DATA\YC\YC-4-161\YC-4-161 2016-10-24 20-05-36\051-0201.D
Sample Name: yc-4-161-3-rac

```
=====================================================================
Acq. Operator   : SYSTEM                        Seq. Line :   2
Acq. Instrument : 1260HPLC-DAD                   Location : Vial 51
Injection Date  : 10/24/2016 8:17:28 PM               Inj :   1
                                               Inj Volume : 3.000 µl
Acq. Method     : E:\DATA\YC\YC-4-161\YC-4-161 2016-10-24 20-05-36\DAD-OD(1-2)-99-1-1ML-
                  3UL-210NM-40MIN.M
Last changed    : 10/24/2016 8:05:36 PM by SYSTEM
Analysis Method : E:\DATA\YC\YC-4-161\YC-4-161 2016-10-24 20-05-36\DAD-OD(1-2)-99-1-1ML-
                  3UL-210NM-40MIN.M (Sequence Method)
Last changed    : 10/24/2016 9:39:48 PM by SYSTEM
                  (modified after loading)
Additional Info : Peak(s) manually integrated
```

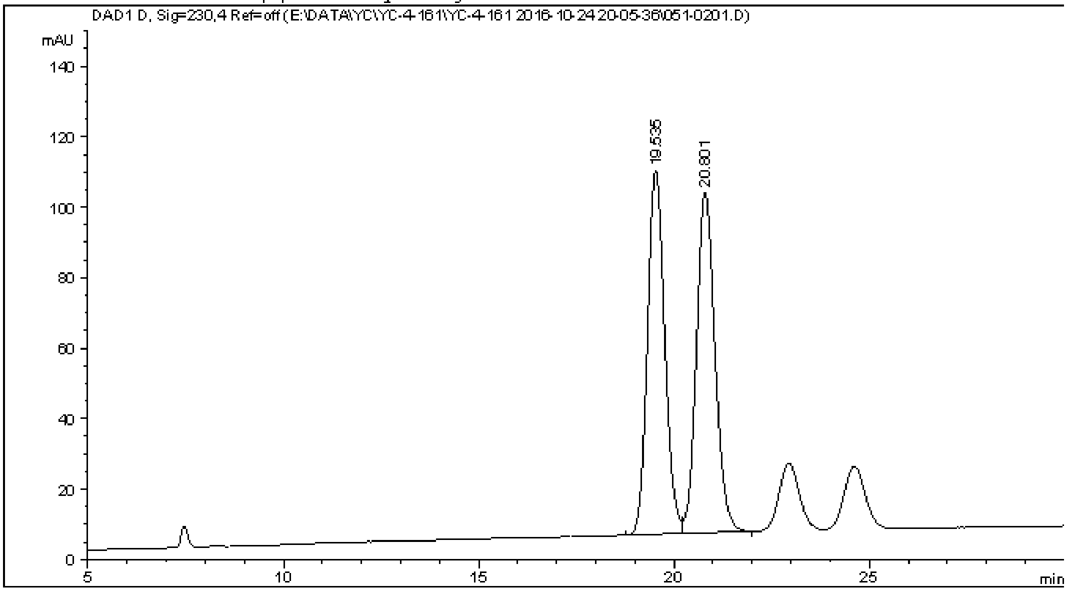


```
=====================================================================
                         Area Percent Report
=====================================================================

Sorted By             :      Signal
Multiplier            :      1.0000
Dilution              :      1.0000
Do not use Multiplier & Dilution Factor with ISTDs


Signal 1: DAD1 D, Sig=230,4 Ref=off

Peak RetTime Type  Width     Area      Height     Area
  #   [min]        [min]   [mAU*s]     [mAU]        %
----|-------|----|-------|----------|----------|--------|
   1  19.535 BV    0.4744 3169.05493  103.15356  49.8158
   2  20.801 VB    0.4952 3192.48901   96.68012  50.1842

Totals :                  6361.54395  199.83368


=====================================================================
                          *** End of Report ***
```

1260HPLC-DAD 10/24/2016 9:39:50 PM SYSTEM Page 1 of 1

```
Data File E:\DATA\YC\YC-4-161\YC-4-161 2016-10-24 20-05-36\052-0301.D
Sample Name: yc-4-161-3

=====================================================================
Acq. Operator   : SYSTEM                        Seq. Line :   3
Acq. Instrument : 1260HPLC-DAD                   Location : Vial 52
Injection Date  : 10/24/2016 8:58:21 PM               Inj :   1
                                                Inj Volume : 3.000 µl
Acq. Method     : E:\DATA\YC\YC-4-161\YC-4-161 2016-10-24 20-05-36\DAD-OD(1-2)-99-1-1ML-
                  3UL-210NM-40MIN.M
Last changed    : 10/24/2016 9:31:46 PM by SYSTEM
                  (modified after loading)
Analysis Method : E:\DATA\YC\YC-4-161\YC-4-161 2016-10-24 20-05-36\DAD-OD(1-2)-99-1-1ML-
                  3UL-210NM-40MIN.M (Sequence Method)
Last changed    : 10/24/2016 9:37:48 PM by SYSTEM
                  (modified after loading)
Additional Info : Peak(s) manually integrated
```

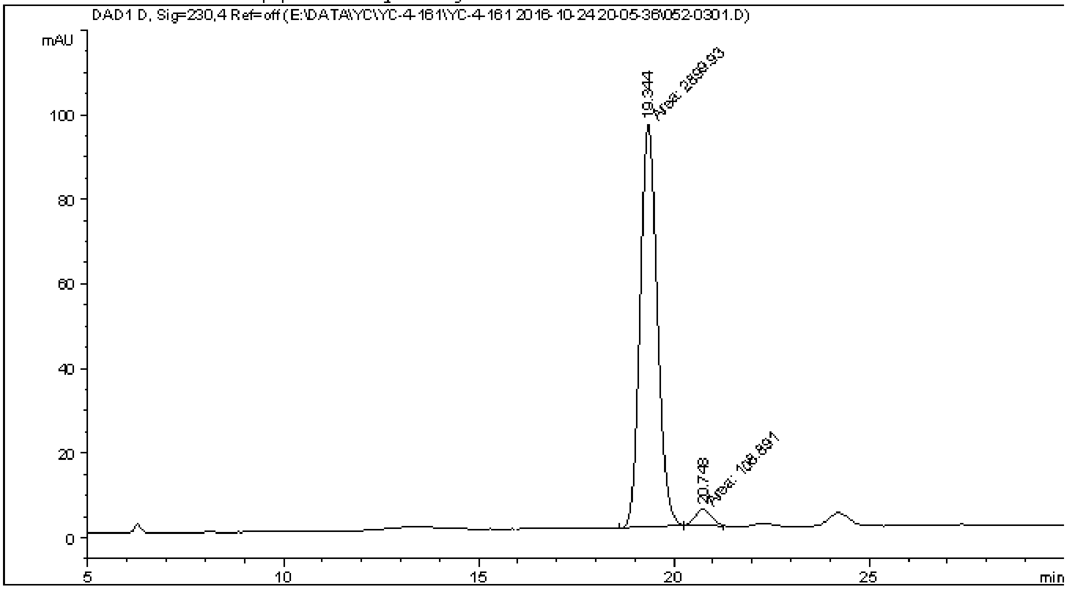


```
=====================================================================
                        Area Percent Report
=====================================================================

Sorted By             :      Signal
Multiplier            :      1.0000
Dilution              :      1.0000
Do not use Multiplier & Dilution Factor with ISTDs


Signal 1: DAD1 D, Sig=230,4 Ref=off

Peak RetTime Type  Width     Area      Height     Area
  #   [min]        [min]   [mAU*s]     [mAU]        %
----|-------|----|-------|----------|----------|--------|
   1  19.344 MM    0.5077 2899.92969   95.19570  96.3809
   2  20.748 MM    0.4825  108.89140    3.76144   3.6191

Totals :                  3008.82108   98.95714

=====================================================================
```


Supplementary Figure 104. HPLC spectra for compound 2q

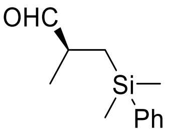

2r

Data File E:\DATA\WQ\WQ-3-53\WQ-3-54-4 2016-10-28 10-59-41\045-1101.D
Sample Name: YC-4-167-1-RAC

===

Acq. Operator : SYSTEM Seq. Line : 11
Acq. Instrument : 1260HPLC-VWD Location : Vial 45
Injection Date : 10/28/2016 4:14:33 PM Inj : 1
Inj Volume : 3.000 µl
Acq. Method : E:\DATA\WQ\WQ-3-53\WQ-3-54-4 2016-10-28 10-59-41\VWD-AD(1-6)-99-1-1.5ML-3UL-210NM-40MIN.M
Last changed : 10/28/2016 4:27:33 PM by SYSTEM
(modified after loading)
Analysis Method : E:\DATA\WQ\WQ-3-53\WQ-3-54-4 2016-10-28 10-59-41\VWD-AD(1-6)-99-1-1.5ML-3UL-210NM-40MIN.M (Sequence Method)
Last changed : 10/28/2016 5:28:12 PM by SYSTEM
(modified after loading)
Additional Info : Peak(s) manually integrated

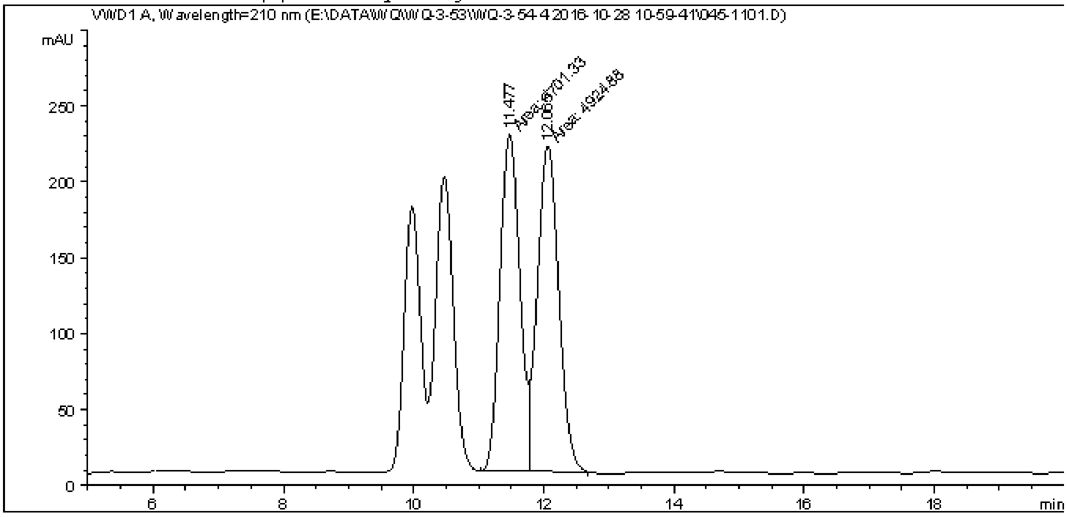

===

Area Percent Report

===

Sorted By : Signal
Multiplier : 1.0000
Dilution : 1.0000
Do not use Multiplier & Dilution Factor with ISTDs

Signal 1: VWD1 A, Wavelength=210 nm

Peak #	RetTime [min]	Type	Width [min]	Area [mAU*s]	Height [mAU]	Area %
1	11.477	MF	0.3528	4701.32715	222.11121	48.8388
2	12.065	FM	0.3826	4924.88037	214.55661	51.1612

Totals : 9626.20752 436.66782

===

*** End of Report ***

1260HPLC-VWD 10/28/2016 5:29:43 PM SYSTEM Page 1 of 1

Data File E:\DATA\WQ\WQ-3-53\WQ-3-54-4 2016-10-28 10-59-41\046-1201.D
Sample Name: YC-4-167-1

```
=====================================================================
Acq. Operator   : SYSTEM                          Seq. Line :  12
Acq. Instrument : 1260HPLC-VWD                     Location : Vial 46
Injection Date  : 10/28/2016 4:35:20 PM                 Inj :   1
                                                Inj Volume : 3.000 µl
Acq. Method     : E:\DATA\WQ\WQ-3-53\WQ-3-54-4 2016-10-28 10-59-41\VWD-AD(1-6)-99-1-1.5ML-
                  3UL-210NM-40MIN.M
Last changed    : 10/28/2016 4:27:33 PM by SYSTEM
Analysis Method : E:\DATA\WQ\WQ-3-53\WQ-3-54-4 2016-10-28 10-59-41\VWD-AD(1-6)-99-1-1.5ML-
                  3UL-210NM-40MIN.M (Sequence Method)
Last changed    : 10/28/2016 5:31:39 PM by SYSTEM
                  (modified after loading)
Additional Info : Peak(s) manually integrated
```

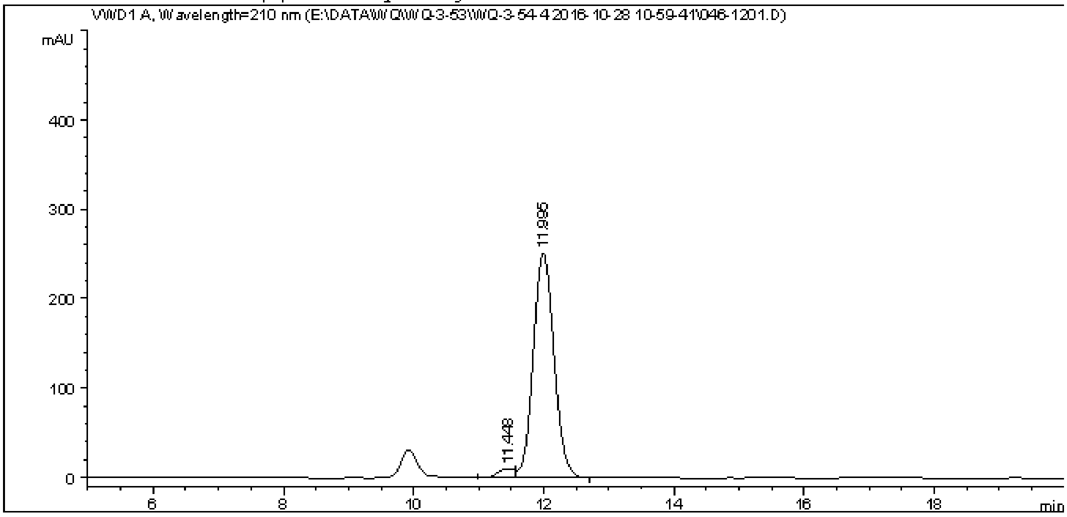


```
=====================================================================
                        Area Percent Report
=====================================================================

Sorted By           :      Signal
Multiplier          :      1.0000
Dilution            :      1.0000
Do not use Multiplier & Dilution Factor with ISTDs


Signal 1: VWD1 A, Wavelength=210 nm
```

Peak #	RetTime [min]	Type	Width [min]	Area [mAU*s]	Height [mAU]	Area %
1	11.448	BV	0.2843	181.72940	9.98778	3.1546
2	11.995	VB	0.3481	5578.96875	251.25012	96.8454

Totals : 5760.69815 261.23791

```
=====================================================================
                        *** End of Report ***
```

1260HPLC-VWD 10/28/2016 5:34:09 PM SYSTEM   Page 1 of 1

Supplementary Figure 105. HPLC spectra for compound 2r

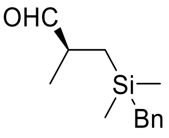

2s

```
Data File E:\DATA\YC\YC-4-177-OD\YC-4-177-OD 2016-11-18 17-10-52\011-0201.D
Sample Name: YC-4-177-1-RAC

=====================================================================
Acq. Operator   : SYSTEM                          Seq. Line :   2
Acq. Instrument : 1260HPLC-DAD                     Location : Vial 11
Injection Date  : 11/18/2016 5:22:44 PM                 Inj :   1
                                                Inj Volume : 3.000 µl
Acq. Method     : E:\DATA\YC\YC-4-177-OD\YC-4-177-OD 2016-11-18 17-10-52\DAD-OD(1-2)-99-1-
                  1ML-3UL-210NM-40MIN.M
Last changed    : 11/18/2016 6:00:32 PM by SYSTEM
                  (modified after loading)
Analysis Method : E:\DATA\YC\YC-4-177-OD\YC-4-177-OD 2016-11-18 17-10-52\DAD-OD(1-2)-99-1-
                  1ML-3UL-210NM-40MIN.M (Sequence Method)
Last changed    : 11/18/2016 10:06:49 PM by SYSTEM
                  (modified after loading)
Additional Info : Peak(s) manually integrated
```

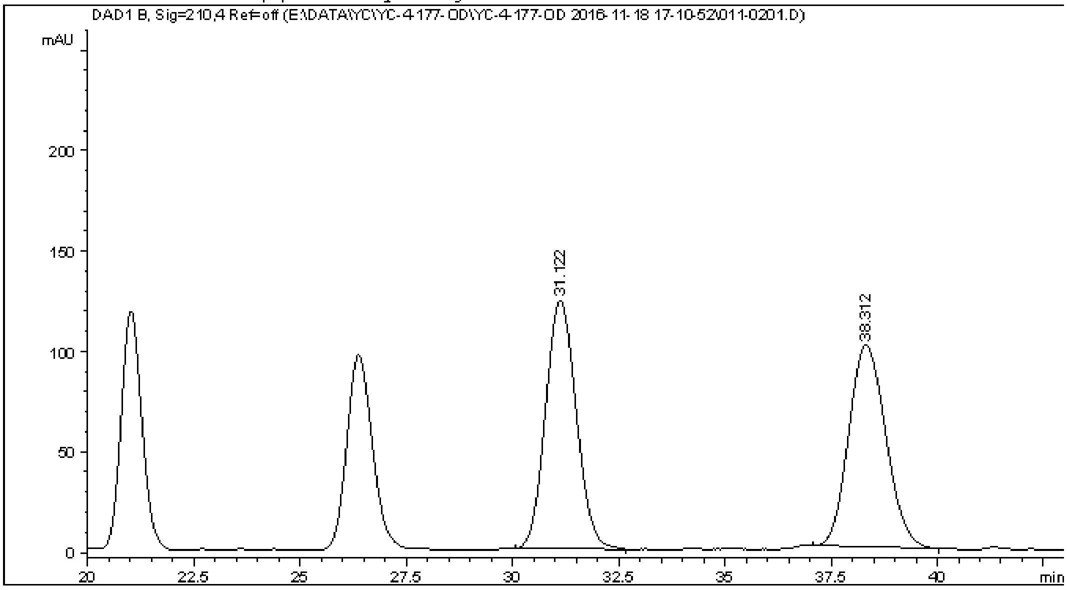


```
=====================================================================
                         Area Percent Report
=====================================================================

Sorted By             :      Signal
Multiplier            :      1.0000
Dilution              :      1.0000
Do not use Multiplier & Dilution Factor with ISTDs


Signal 1: DAD1 B, Sig=210,4 Ref=off

Peak RetTime Type  Width     Area      Height     Area
  #   [min]        [min]   [mAU*s]     [mAU]       %
----|-------|----|-------|----------|----------|--------|
   1  31.122 BB    0.6021 6230.96582  123.61324  50.4983
   2  38.312 BB    0.7173 6108.00586  100.18985  49.5017

Totals :                  1.23390e4   223.80309


=====================================================================
```

1260HPLC-DAD 11/18/2016 10:06:54 PM SYSTEM Page 1 of 1

Data File E:\DATA\YC\YC-4-177-OD\YC-4-177-OD 2016-11-18 17-10-52\012-0301.D
Sample Name: YC-4-177-1

```
=====================================================================
Acq. Operator   : SYSTEM                          Seq. Line :   3
Acq. Instrument : 1260HPLC-DAD                     Location : Vial 12
Injection Date  : 11/18/2016 6:23:37 PM                 Inj :   1
                                                Inj Volume : 3.000 µl
Acq. Method     : E:\DATA\YC\YC-4-177-OD\YC-4-177-OD 2016-11-18 17-10-52\DAD-OD(1-2)-99-1-
                  1ML-3UL-210NM-40MIN.M
Last changed    : 11/18/2016 6:00:32 PM by SYSTEM
Analysis Method : E:\DATA\YC\YC-4-177-OD\YC-4-177-OD 2016-11-18 17-10-52\DAD-OD(1-2)-99-1-
                  1ML-3UL-210NM-40MIN.M (Sequence Method)
Last changed    : 11/18/2016 10:05:45 PM by SYSTEM
                  (modified after loading)
Additional Info : Peak(s) manually integrated
```

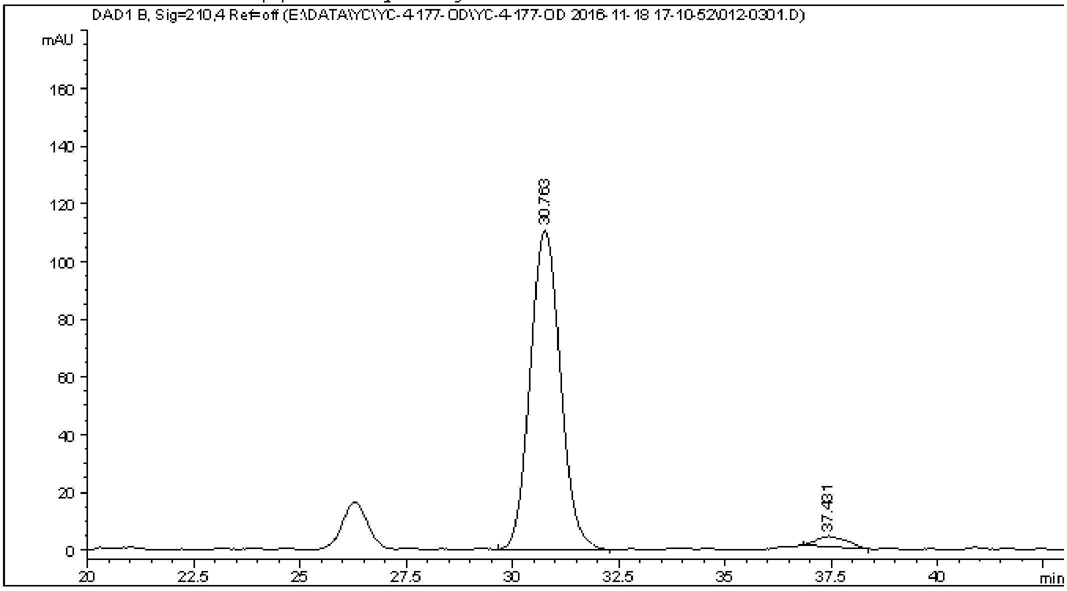

===
Area Percent Report
===

Sorted By : Signal
Multiplier : 1.0000
Dilution : 1.0000
Do not use Multiplier & Dilution Factor with ISTDs

Signal 1: DAD1 B, Sig=210,4 Ref=off

Peak #	RetTime [min]	Type	Width [min]	Area [mAU*s]	Height [mAU]	Area %
1	30.763	BB	0.6154	5473.32031	110.59863	96.8820
2	37.431	BB	0.5873	176.15318	3.52336	3.1180

Totals : 5649.47350 114.12200

===
*** End of Report ***

1260HPLC-DAD 11/18/2016 10:05:48 PM SYSTEM Page 1 of 1

Supplementary Figure 106. HPLC spectra for compound 2s

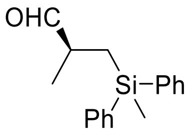

2t

Data File E:\DATA\YC\YC-4-177-AD\YC-4-177-AD 2016-11-18 17-14-44\021-0201.D
Sample Name: YC-4-177-4-RAC

===

```
Acq. Operator   : SYSTEM                          Seq. Line :   2
Acq. Instrument : 1260HPLC-VWD                     Location : Vial 21
Injection Date  : 11/18/2016 5:26:18 PM                 Inj :   1
                                                 Inj Volume : 3.000 µl
Acq. Method     : E:\DATA\YC\YC-4-177-AD\YC-4-177-AD 2016-11-18 17-14-44\VWD-AD(1-6)-99-1-
                  1.5ML-3UL-210NM-40MIN.M
Last changed    : 11/18/2016 5:14:45 PM by SYSTEM
Analysis Method : E:\DATA\YC\YC-4-177-AD\YC-4-177-AD 2016-11-18 17-14-44\VWD-AD(1-6)-99-1-
                  1.5ML-3UL-210NM-40MIN.M (Sequence Method)
Last changed    : 11/18/2016 9:56:43 PM by SYSTEM
                  (modified after loading)
Additional Info : Peak(s) manually integrated
```

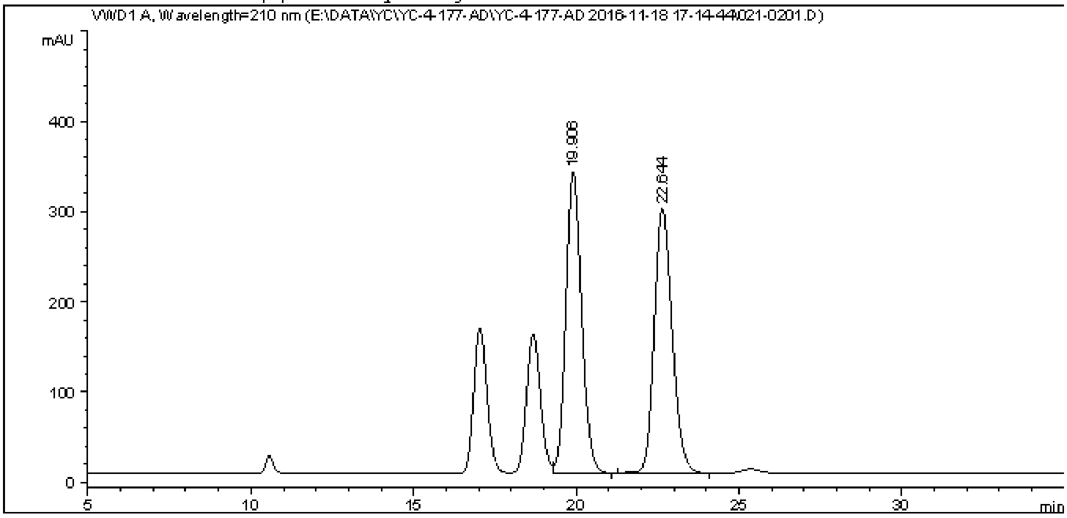

===
Area Percent Report
===

```
Sorted By             :      Signal
Multiplier            :      1.0000
Dilution              :      1.0000
Do not use Multiplier & Dilution Factor with ISTDs
```

Signal 1: VWD1 A, Wavelength=210 nm

Peak #	RetTime [min]	Type	Width [min]	Area [mAU*s]	Height [mAU]	Area %
1	19.906	VB	0.5166	1.11986e4	333.60880	49.8252
2	22.644	BB	0.5947	1.12772e4	293.22806	50.1748

Totals : 2.24758e4 626.83685

===
*** End of Report ***

```
Data File E:\DATA\YC\YC-4-177-AD\YC-4-177-AD 2016-11-18 17-14-44\022-0301.D
Sample Name: YC-4-177-4

    =====================================================================
    Acq. Operator   : SYSTEM                          Seq. Line :   3
    Acq. Instrument : 1260HPLC-VWD                     Location : Vial 22
    Injection Date  : 11/18/2016 6:07:02 PM                 Inj :   1
                                                     Inj Volume : 3.000 µl
    Acq. Method     : E:\DATA\YC\YC-4-177-AD\YC-4-177-AD 2016-11-18 17-14-44\VWD-AD(1-6)-99-1-
                      1.5ML-3UL-210NM-40MIN.M
    Last changed    : 11/18/2016 5:14:45 PM by SYSTEM
    Analysis Method : E:\DATA\YC\YC-4-177-AD\YC-4-177-AD 2016-11-18 17-14-44\VWD-AD(1-6)-99-1-
                      1.5ML-3UL-210NM-40MIN.M (Sequence Method)
    Last changed    : 11/18/2016 9:57:36 PM by SYSTEM
                      (modified after loading)
    Additional Info : Peak(s) manually integrated
```

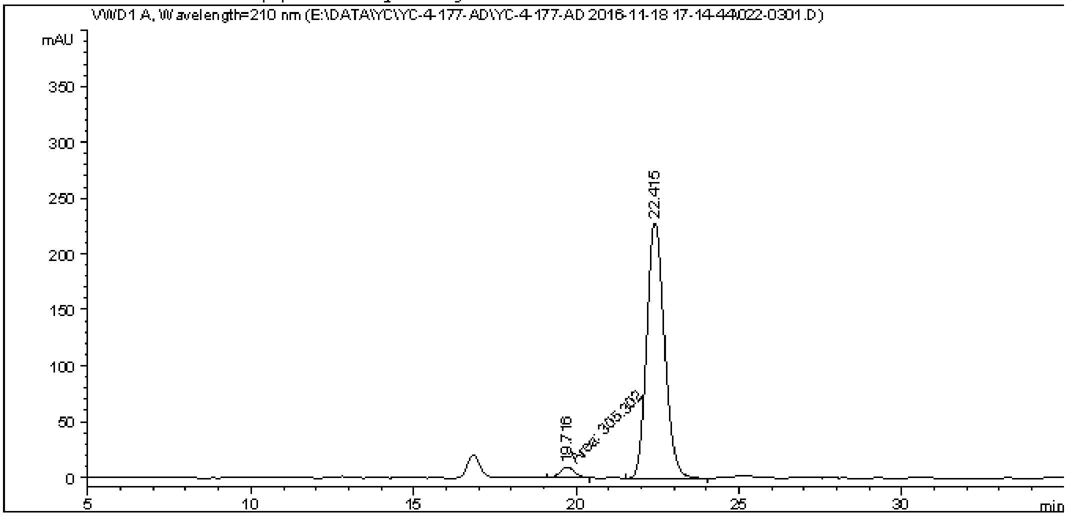


```
    =====================================================================
                             Area Percent Report
    =====================================================================

    Sorted By             :      Signal
    Multiplier            :      1.0000
    Dilution              :      1.0000
    Do not use Multiplier & Dilution Factor with ISTDs


    Signal 1: VWD1 A, Wavelength=210 nm

    Peak RetTime Type  Width     Area      Height     Area
      #   [min]        [min]   [mAU*s]     [mAU]        %
    ----|-------|----|-------|----------|----------|--------|
       1  19.716 MM    0.5457  305.30219    9.32463   3.3914
       2  22.415 BB    0.5871 8696.96582  228.96463  96.6086

    Totals :                  9002.26801  238.28926


    =====================================================================
                              *** End of Report ***
```

1260HPLC-VWD 11/18/2016 9:57:41 PM SYSTEM   Page 1 of 1

Supplementary Figure 107. HPLC spectra for compound 2t

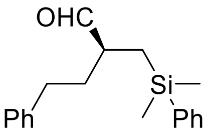

2u

Data File E:\DATA\HZY\SUANGAN\4F-EERAC-161219 2016-12-19 11-42-18\034-1001.D
Sample Name: YC-4-190-RAC-2

```
=====================================================================
Acq. Operator   : SYSTEM                          Seq. Line :  10
Acq. Instrument : 1260HPLC-DAD                     Location : Vial 34
Injection Date  : 12/19/2016 4:17:58 PM                 Inj :   1
                                                 Inj Volume : 3.000 µl
Acq. Method     : E:\DATA\HZY\SUANGAN\4F-EERAC-161219 2016-12-19 11-42-18\DAD-OD(1-2)-99-1
                  -3UL-1ML-210NM-80MIN.M
Last changed    : 12/19/2016 5:36:08 PM by SYSTEM
                  (modified after loading)
Analysis Method : E:\DATA\HZY\SUANGAN\4F-EERAC-161219 2016-12-19 11-42-18\DAD-OD(1-2)-99-1
                  -3UL-1ML-210NM-80MIN.M (Sequence Method)
Last changed    : 12/19/2016 7:33:34 PM by SYSTEM
                  (modified after loading)
Additional Info : Peak(s) manually integrated
```

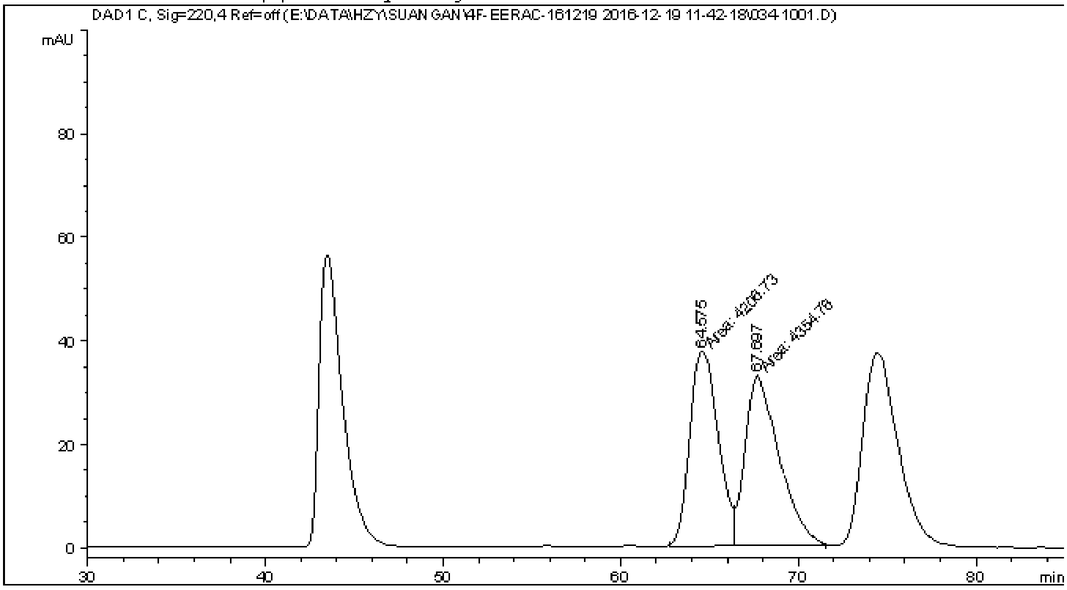


```
=====================================================================
                         Area Percent Report
=====================================================================

Sorted By             :      Signal
Multiplier            :      1.0000
Dilution              :      1.0000
Do not use Multiplier & Dilution Factor with ISTDs


Signal 1: DAD1 C, Sig=220,4 Ref=off

Peak RetTime Type  Width     Area      Height     Area
  #   [min]        [min]   [mAU*s]     [mAU]       %
----|-------|----|-------|----------|----------|--------|
   1  64.575 MF    1.8684 4206.73438   37.52433  49.1355
   2  67.697 FM    2.2133 4354.75684   32.79295  50.8645

Totals :                  8561.49121   70.31728


=====================================================================
```

1260HPLC-DAD 12/19/2016 7:33:38 PM SYSTEM   Page 1 of 1

Data File E:\DATA\HZY\SUANGAN\4F-EERAC-161219 2016-12-19 11-42-18\035-1101.D
Sample Name: YC-4-190-2

```
=====================================================================
Acq. Operator   : SYSTEM                        Seq. Line :  11
Acq. Instrument : 1260HPLC-DAD                   Location : Vial 35
Injection Date  : 12/19/2016 5:43:53 PM               Inj :  1
                                               Inj Volume : 5.000 µl
Acq. Method     : E:\DATA\HZY\SUANGAN\4F-EERAC-161219 2016-12-19 11-42-18\DAD-OD(1-2)-99-1
                  -3UL-1ML-210NM-80MIN.M
Last changed    : 12/19/2016 5:36:08 PM by SYSTEM
Analysis Method : E:\DATA\HZY\SUANGAN\4F-EERAC-161219 2016-12-19 11-42-18\DAD-OD(1-2)-99-1
                  -3UL-1ML-210NM-80MIN.M (Sequence Method)
Last changed    : 12/19/2016 7:32:22 PM by SYSTEM
                  (modified after loading)
Additional Info : Peak(s) manually integrated
```

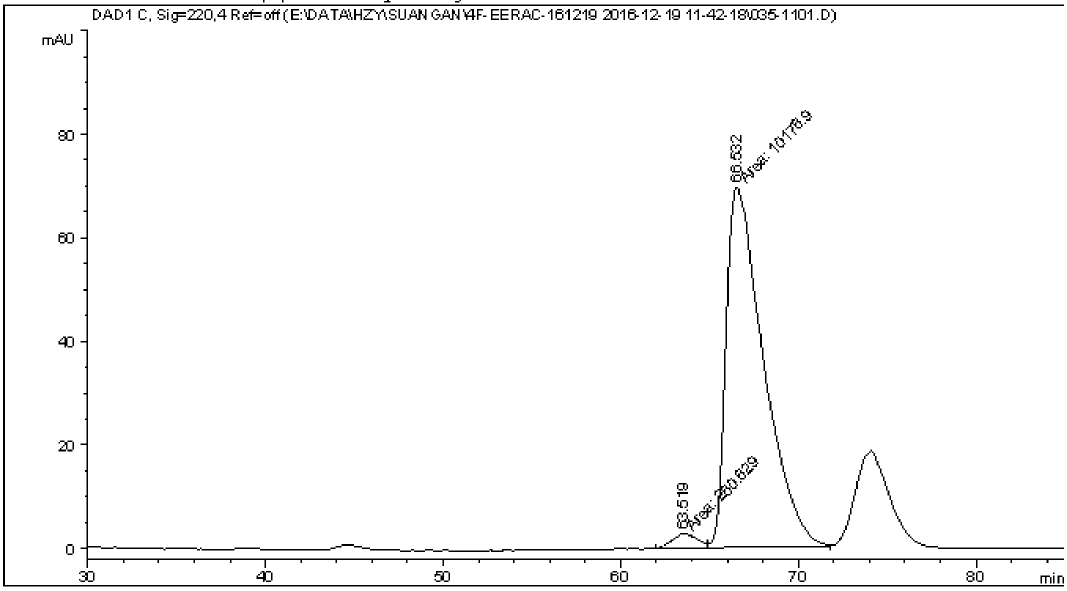


```
=====================================================================
                         Area Percent Report
=====================================================================

Sorted By             :      Signal
Multiplier            :      1.0000
Dilution              :      1.0000
Do not use Multiplier & Dilution Factor with ISTDs


Signal 1: DAD1 C, Sig=220,4 Ref=off

Peak RetTime Type  Width     Area      Height     Area
  #   [min]        [min]   [mAU*s]     [mAU]        %
----|-------|----|-------|----------|----------|--------|
   1  63.519 MF    1.6253  260.62927    2.67259   2.4970
   2  66.532 FM    2.4358 1.01769e4    69.63472  97.5030

Totals :                  1.04375e4    72.30731


=====================================================================
                          *** End of Report ***
```

1260HPLC-DAD 12/19/2016 7:32:25 PM SYSTEM Page 1 of 1

Supplementary Figure 108. HPLC spectra for compound 2u

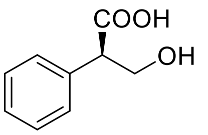

(*R*)-Tropic acid, **5**

Data File E:\DATA\GWC\GWC16-12\YC-4-188 2016-12-11 12-55-58\041-0401.D
Sample Name: YC-4-188-RAC

===

Acq. Operator : SYSTEM Seq. Line : 4
Acq. Instrument : 1260HPLC-DAD Location : Vial 41
Injection Date : 12/11/2016 2:09:45 PM Inj : 1
Inj Volume : 3.000 µl
Acq. Method : E:\DATA\GWC\GWC16-12\YC-4-188 2016-12-11 12-55-58\DAD-AD(1-2)-95-5-1.0ML-3UL-210NM-35MIN.M
Last changed : 12/11/2016 1:03:40 PM by SYSTEM
Analysis Method : E:\DATA\GWC\GWC16-12\YC-4-188 2016-12-11 12-55-58\DAD-AD(1-2)-95-5-1.0ML-3UL-210NM-35MIN.M (Sequence Method)
Last changed : 12/11/2016 4:12:08 PM by SYSTEM
(modified after loading)
Additional Info : Peak(s) manually integrated

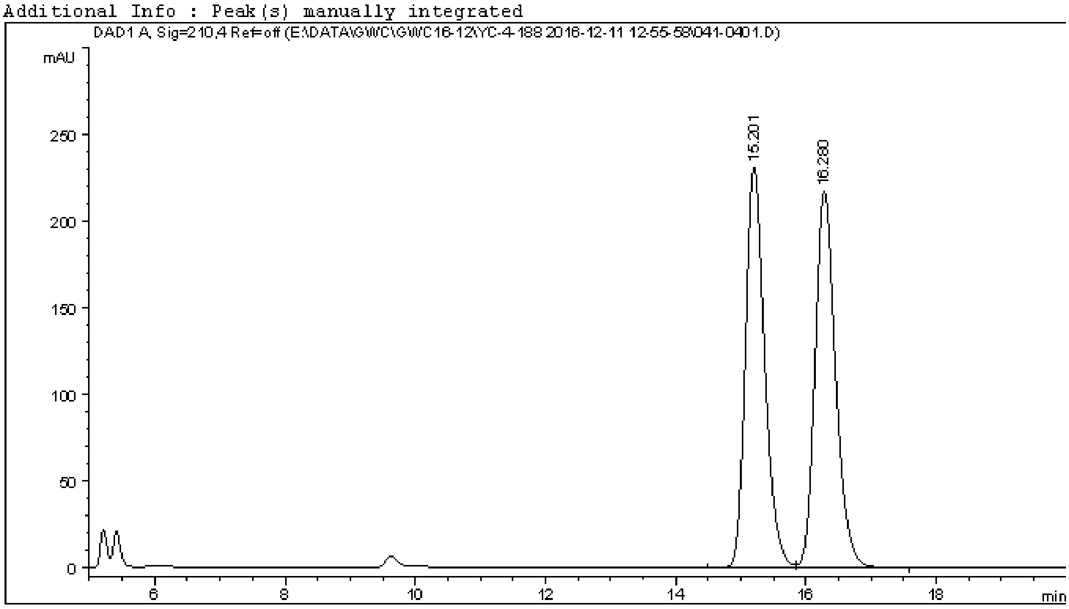

===

Area Percent Report

===

Sorted By : Signal
Multiplier : 1.0000
Dilution : 1.0000
Do not use Multiplier & Dilution Factor with ISTDs

Signal 1: DAD1 A, Sig=210,4 Ref=off

Peak #	RetTime [min]	Type	Width [min]	Area [mAU*s]	Height [mAU]	Area %
1	15.201	BV	0.3131	4749.20605	231.68581	49.9124
2	16.280	VB	0.3339	4765.86963	217.24519	50.0876

Totals : 9515.07568 448.93100

===

*** End of Report ***

1260HPLC-DAD 12/11/2016 4:12:13 PM SYSTEM Page 1 of 1

```
Data File E:\DATA\GWC\GWC16-12\YC-4-188 2016-12-11 12-55-58\042-0501.D
Sample Name: YC-4-188-2

=====================================================================
Acq. Operator   : SYSTEM                          Seq. Line :   5
Acq. Instrument : 1260HPLC-DAD                     Location : Vial 42
Injection Date  : 12/11/2016 2:45:39 PM                 Inj :   1
                                                 Inj Volume : 3.000 µl
Acq. Method     : E:\DATA\GWC\GWC16-12\YC-4-188 2016-12-11 12-55-58\DAD-AD(1-2)-95-5-1.0ML
                  -3UL-210NM-35MIN.M
Last changed    : 12/11/2016 1:03:40 PM by SYSTEM
Analysis Method : E:\DATA\GWC\GWC16-12\YC-4-188 2016-12-11 12-55-58\DAD-AD(1-2)-95-5-1.0ML
                  -3UL-210NM-35MIN.M (Sequence Method)
Last changed    : 12/11/2016 4:11:01 PM by SYSTEM
                  (modified after loading)
Additional Info : Peak(s) manually integrated
```

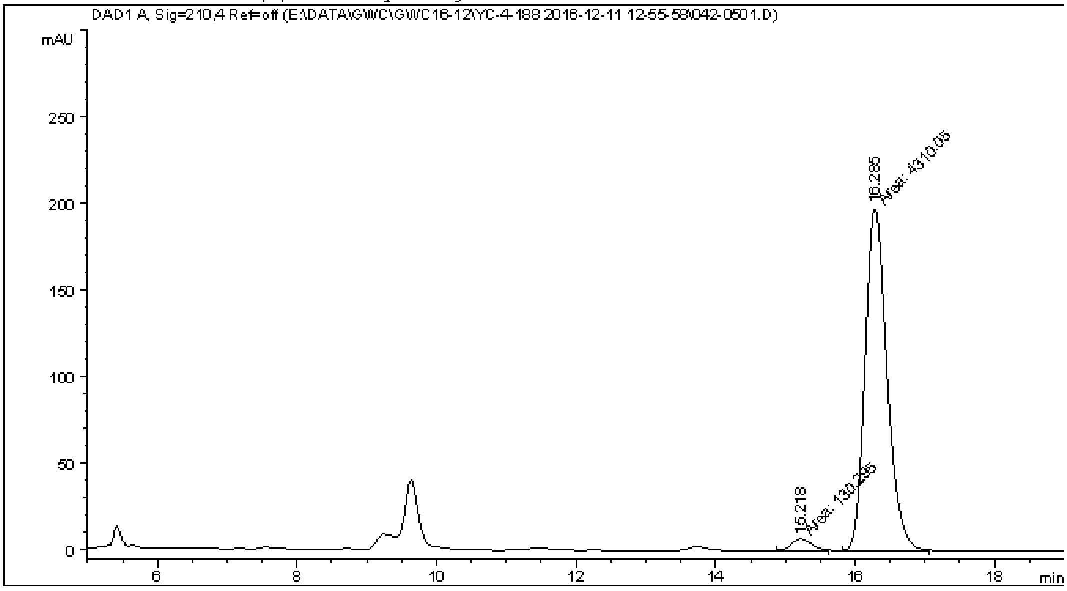


```
=====================================================================
                         Area Percent Report
=====================================================================

Sorted By             :      Signal
Multiplier            :      1.0000
Dilution              :      1.0000
Do not use Multiplier & Dilution Factor with ISTDs


Signal 1: DAD1 A, Sig=210,4 Ref=off

Peak RetTime Type  Width     Area      Height     Area
  #   [min]        [min]   [mAU*s]     [mAU]        %
----|-------|----|-------|----------|----------|--------|
   1  15.218 MM    0.3307  130.29491    6.56642   2.9343
   2  16.285 MM    0.3632 4310.04688  197.76315  97.0657

Totals :                  4440.34178  204.32958


=====================================================================
                          *** End of Report ***

1260HPLC-DAD 12/11/2016 4:11:05 PM SYSTEM                      Page 1 of 1
```

Supplementary Figure 109. HPLC spectra for compound 5

Supplementary References

1. Sun, F.; Gu, Z. Decarboxylative Alkynyl Termination of Palladium-Catalyzed Catellani Reaction: A Facile Synthesis of α-Alkynyl Anilines via *Ortho* C–H Amination and Alkynylation. *Org. Lett.*, **17**, 2222-2225 (2015).
2. Nishihara, Y.; Saito, D.; Tanemura, K.; Noyori, S.; Takagi, K. Regio- and Stereoselective Synthesisof Multisubstituted Vinylsilanes via Zirconacycles. *Org. Lett.* **11**, 3546-3549 (2009).
3. Sheshenev, A. E.; Baird, M. S.; Bolesov, I. G.; Shashkov, A. S. Stereo- and Regiocontrol in Ene-dimerisation and Trimerisation of 1-trimethylsilyl-3-phenylcyclopropene. *Tetrahedron.* ***65***, 10552-10564 (2009).
4. Kubota, K.; Yamamoto, E.; Ito, H. Regio□ and Enantioselective Monoborylation of Alkenylsilanes Catalyzed by an Electron□Donating Chiral Phosphine–Copper(I) Complex. *Adv. Synth. Catal.* **355**, 3527-3531 (2013).
5. Zhang, X. W.; Cao, B. N.; Yu, S. C.; Zhang, X. M. Rhodium□Catalyzed Asymmetric Hydroformylation of *N*□Allylamides: Highly Enantioselective Approach to β^2□Amino Aldehydes. *Angew. Chem. Int. Ed.* **49**, 4047-4050 (2010).
6. Klomp, D.; Peters, J. A.; Hanefeld, U. Enzymatic Kinetic Resolution of Tropic Acid. *Tetrahedron: Asymmetry.* **16**, 3892-3896 (2005).
7. Zhao, Y.; Truhlar, D. G. Density Functionals with Broad Applicability in Chemistry. *Acc. Chem. Res.* **41**, 157-167 (2008).
8. Ditchfield, R.; Hehre, W. J.; Pople, J. A. Self□Consistent Molecular□Orbital Methods. IX. An Extended Gaussian□Type Basis for Molecular□Orbital Studies of Organic Molecules. *J. Chem. Phys.* **54**, 724-728 (1971).
9. Hehre, W. J.; Ditchfield, R.; Pople, J. A. Self-consistent Molecular Orbital Methods. XII. Further Extensions of Gaussian-type Basis Sets for Use in Molecular Orbital Studies of Organic Molecules. *J. Chem. Phys.* **56**, 2257-2261 (1972).
10. Hariharan, P. C.; Pople, J. A. The Influence of Polarization Functions on Molecular Orbital Hydrogenation Energies. *Theoret. chim. Acta.* **28**, 213-222 (1973).
11. Marenich, A. V.; Cramer, C. J.; Truhlar, D. G. Universal Solvation Model Based on Solute Electron Density and on a Continuum Model of the Solvent Defined by the Bulk Dielectric Constant and Atomic Surface Tensions. *J. Phys. Chem. B.* **113**, 6378-6396 (2009).
12. Becke, A. D. Density-functional Thermochemistry. III. The Role of Exact Exchange. *J. Chem. Phys.* **98**, 5648-5652 (1993).
13. Lee, C.; Yang, W.; Parr, R. G. Development of the Colle-Salvetti Correlation-energy Formula into a Functional of the Electron Density. *Phys. Rev. B.* **37**, 785-789 (1988).
14. Vosko, S. H.; Wilk, L.; Nusair, M. Accurate Spin-dependent Electron Liquid Correlation Energies for Local Spin Density Calculations: A Critical Analysis. *Can. J. Phys.* **58**, 1200-1211 (1980).
15. Grimme, S.; Antony, J.; Ehrlich, S.; Krieg, H. A Consistent and Accurate ab Initio-parametrization of Density Functional Dispersion Correction (DFT-D) for the 94 Elements H-Pu. *J. Chem. Phys.* **132**, 154104-154119 (2010).
16. Gaussian 09.; Revision D.01.; Frisch, M. J.; Trucks, G. W.; Schlegel, H. B.; Scuseria, G. E.; Robb, M. A.; Cheeseman, J. R.; Scalmani, G.; Barone, V.; Mennucci, B.; G. Petersson, A.; Nakatsuji, H.; Caricato, M.; Li, X.; Hratchian, H. P.; Izmaylov, A. F.; Bloino, J.; Zheng, G.;

Sonnenberg, J. L.; Hada, M.; Ehara, M.; Toyota, K.; Fukuda, R.; Hasegawa, J.; Ishida, M.; Nakajima, T.; Honda, Y.; Kitao, O.; Nakai, H.; Vreven, T.; Montgomery, J. A.; Peralta, J. E.; Ogliaro, F.; Bearpark, M.; Heyd, J. J.; Brothers, E.; Kudin, K. N.; Staroverov, V. N.; Kobayashi, R.; Normand, J.; Raghavachari, K.; Rendell, A.; Burant, J. C.; Iyengar, S. S.; Tomasi, J.; Cossi, M.; Rega, N.; Millam, J. M.; Klene, M.; Knox, J. E.; Cross, J. B.; Bakken, V.; Adamo, C.; Jaramillo, J.; Gomperts, R.; Stratmann, R. E.; Yazyev, O.; Austin, A. J.; Cammi, R.; Pomelli, C.; Ochterski, J. W.; Martin, R. L.; Morokuma, K.; Zakrzewski, V. G.; Voth, G. A.; Salvador, P.; Dannenberg, J. J.; Dapprich, S.; Daniels, A. D.; Farkas, Ö.

17. Legault, C. Y. CYL View, version 1.0 b; Universite de Sherbrooke, Sherbrooke, Quebec, Canada, http://www.cylview.org (2009).
