# Supplementary material for: Silicon-oriented regio- and enantioselective rhodium-catalyzed hydroformylation
Source: Nat Commun. 2018 May 23;9:2045. doi: 10.1038/s41467-018-04277-7 (PMC5966446; doi:10.1038/s41467-018-04277-7)
Supplement: Supplementary file 2 — Description of Additional Supplementary Files [file 41467_2018_4277_MOESM2_ESM.pdf]

Description of Additional Supplementary Files

File Name: Supplementary Data 1
Description: Cartesian coordinates of all stationary points optimized at M06/6-31G* level.
